# Design, synthesis, and docking analysis of benzene sulfonamide-[1,4]benzoxazepine hybrids as acetylcholinesterase and butyrylcholinesterase inhibitors

**DOI:** 10.1039/d6ra08312a

**Published:** 2026-11-04

**Authors:** Almeqdad Y. Habashneh, Muhammad Ashram, Wafa Hourani, Mahmoud A. Al-Sha'er, Murad A. AlDamen

**Affiliations:** a Department of Chemistry, The University of Jordan Amman 11942 Jordan a.habashneh@ju.edu.jo; b Department of Chemistry, Mutah University P.O. Box 7 Al-Karak 61710 Jordan; c Department of Pharmaceutical Sciences, Faculty of Pharmacy, Philadelphia University Amman 19392 Jordan; d Department of Pharmaceutical Sciences, Zarqa University P.O. Box 13111 Zarqa 2000 Jordan

## Abstract

A series of novel hybrid [1,4]benzoxazepine-based sulfonamide derivatives 9–12a–j were synthesized and evaluated for their inhibitory activity against acetylcholinesterase (AChE) and butyrylcholinesterase (BChE). Compounds 9a and 9b exhibited potent dual cholinesterase inhibition, with IC_50_ values of 9.04 and 8.61 µM against AChE and 3.08 and 4.64 µM against BChE, respectively. Compound 12j emerged as the most potent BChE inhibitor of the series (IC_50_ = 1.06 ± 0.11 µM), displaying pronounced BChE preference (SI = 10.37), whereas 9d showed the highest BChE selectivity (BChE IC_50_ = 4.18 ± 0.56 µM; SI = 14.94). Molecular docking studies revealed plausible binding modes of the active compounds within the AChE and BChE active sites, involving π–π stacking, hydrogen bonding, and hydrophobic interactions. The SAR analysis indicated that the effects of nitro and halogen substituents were scaffold- and position-dependent, with selected nitro-substituted derivatives showing enhanced cholinesterase inhibition, whereas brominated analogs exhibited a pronounced preference toward BChE. Integration of the biological and computational data identified compounds 9a, 9b, 9d, 12b, 12j, and 12j as promising candidates for further investigation. Among these, 12j represents the potency lead for BChE inhibition, whereas 9d provides the most pronounced BChE selectivity. ADMET predictions further suggested more favorable physicochemical and pharmacokinetic profiles for selected benzoxazepine-based derivatives, although potential toxicity liabilities were predicted for 12j. Overall, these findings support the [1,4]benzoxazepine–sulfonamide scaffold as a promising starting point for developing BChE-preferring cholinesterase inhibitors.

## Abstract 2

Compound 12j was evaluated for its inhibitory activity against acetylcholinesterase (AChE) and butyrylcholinesterase (BChE), showing preferential inhibition of BChE over AChE with selectivity index = 10.37.

## Graphical abstract

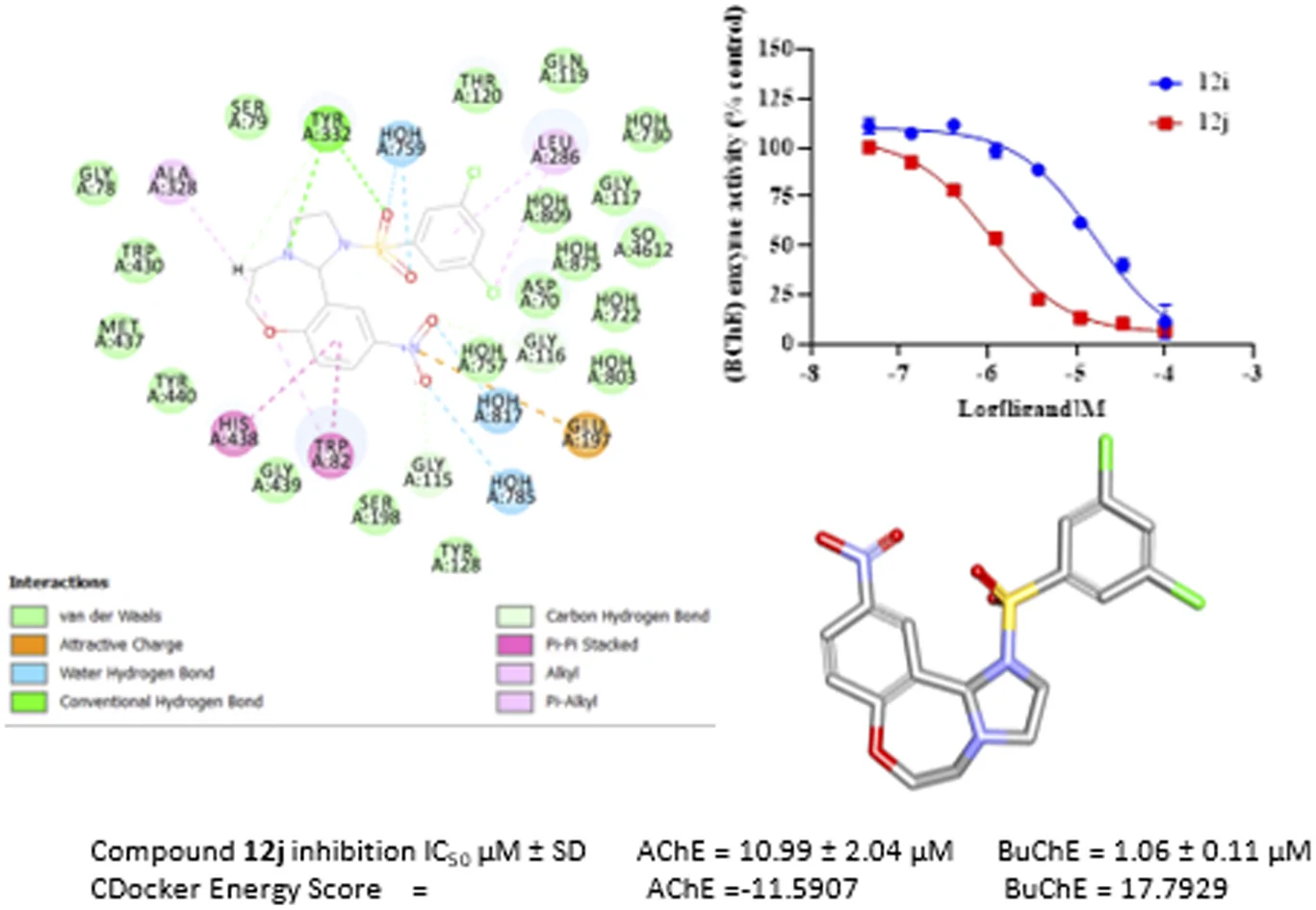

## 1. Introduction

Alzheimer's disease (AD) is the most common neurodegenerative disease, which is manifested by memory loss and weakened motor skills. Although available treatments can improve symptoms or slow disease progression, no treatment completely halts or cures AD. Furthermore, the actual cause of AD remains unclear. However, strong evidence indicates that AD is associated with reduced acetylcholine levels and altered phospholipid metabolism.^1,2^ Cholinesterase enzymes (ChEs) such as acetylcholinesterase (AChE) and butyrylcholinesterase (BChE) catalyze the hydrolysis of the neurotransmitter acetylcholine into choline and acetic acid.^3^ Cholinesterase inhibitors reduce acetylcholine hydrolysis, thereby increasing acetylcholine availability and enhancing cholinergic neurotransmission in patients with AD.^4^ Approved pharmacological treatments for AD include the cholinesterase inhibitors tacrine,^5^ donepezil,^6^ rivastigmine,^7^ and galantamine,^8^ as well as the NMDA receptor antagonist memantine^9^ (Fig. 1).

**Fig. 1:**
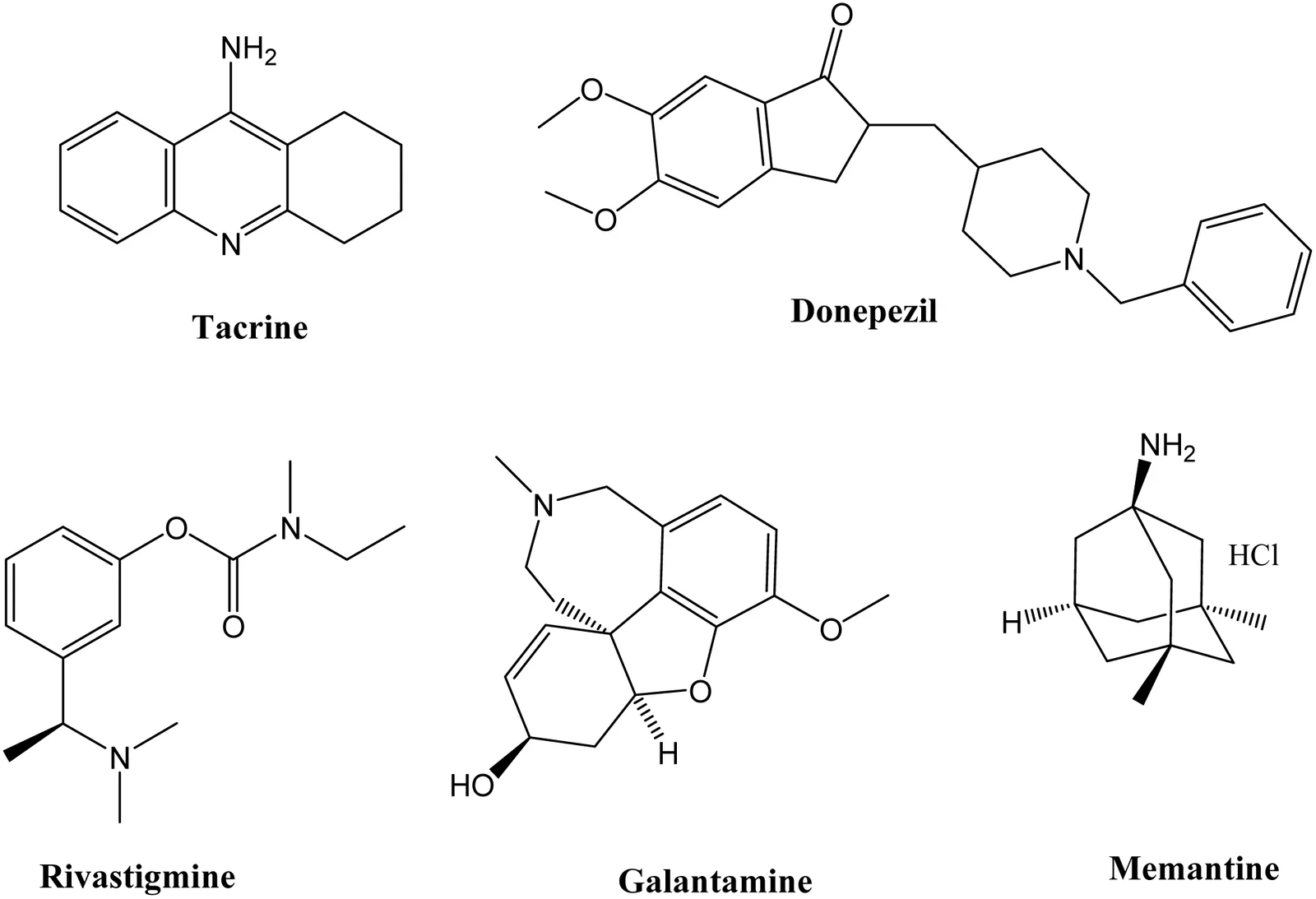
Representative pharmacological treatments for AD include cholinesterase inhibitors and the NMDA receptor antagonist memantine.

However, these drugs can only improve symptoms for most patients, but do not address the etiology of AD. Therefore, extensive research has focused on synthesizing heterocyclic derivatives with strong ChE-inhibitory activity. For instance, as shown in Fig. 2, the indole–thiadiazole-based compound 1 exhibited high potency, with an AChE IC_50_ of 0.17 ± 0.05 µM and a BChE IC_50_ of 0.30 ± 0.1 µM.^10^ Coumarin-3-carboxamide with *N*-benzylpiperidine 2 selectively inhibited AChE with IC_50_ = 0.3 µM.^11^ The synthetic flavonoid derivative 3 has potent anti-AChE activity with an AChE IC_50_ of 0.13 ± 0.002 µM and a BChE IC_50_ of 4.82 ± 0.32 µM.^12^

**Fig. 2:**
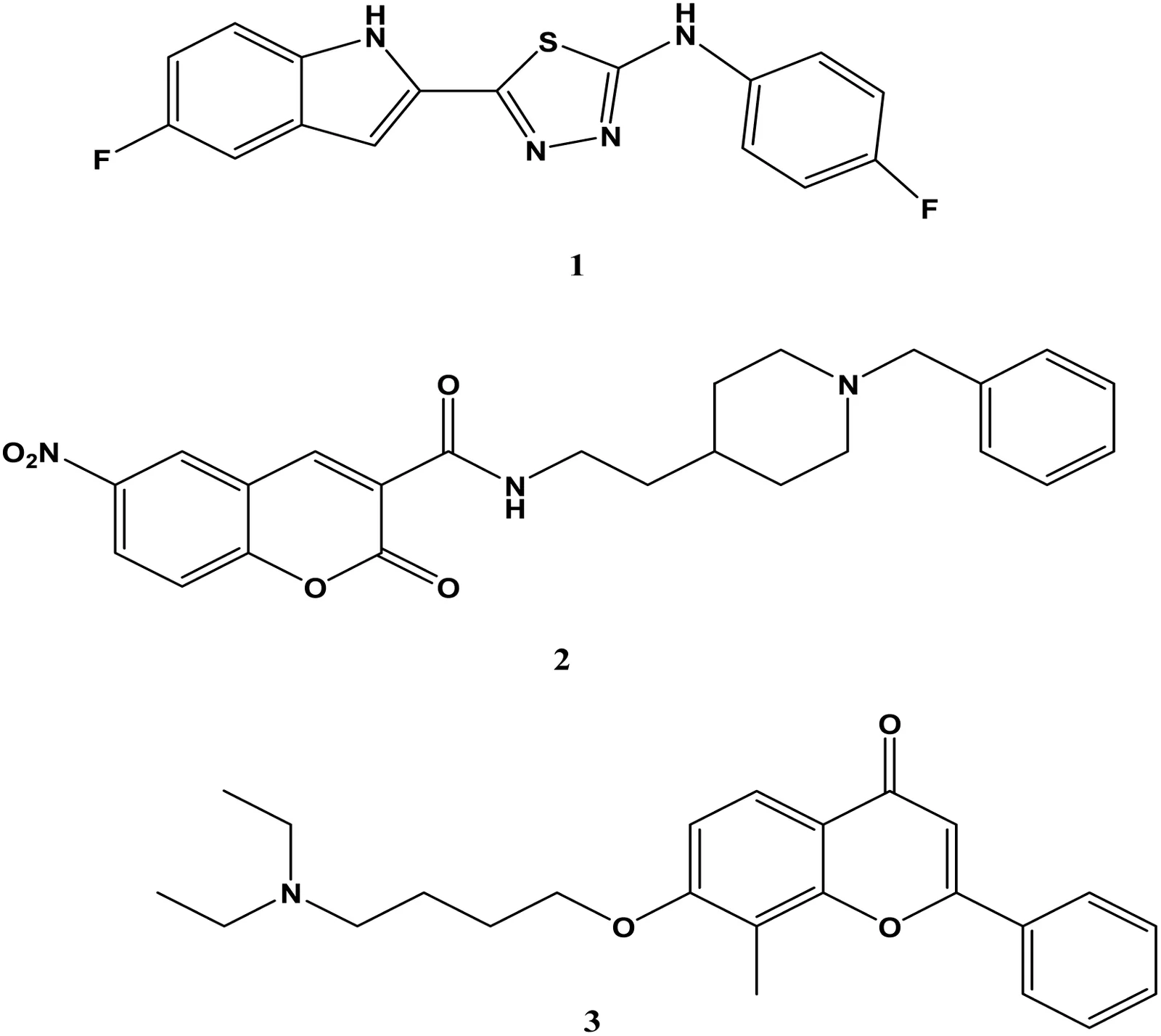
Some examples of compounds exhibit high potency as ChE inhibitors.

Oxazepines are among the privileged nitrogen- and oxygen-containing heterocyclic templates in medicinal chemistry. In particular, the [1,4]benzoxazepine system consists of a benzene ring fused to an oxazepine ring that contains nitrogen and oxygen atoms at the 1,4-positions. The aromatic and heterocyclic components of benzoxazepines can contribute to molecular recognition within the binding sites of AChE and BChE. When the crystal structure of AChE was revealed, it was quite noticeable that the highly asymmetric charge distribution and the constriction midway down the binding site, bearing the quaternary group produced by the juxtaposition of F330 and Y121 in the binding site;^13^ in addition to W279 and Y70 at the top of the binding site, with a narrower diameter (6.4 Å) of AChE. This can play an essential role in π–cation interactions within the AChE cavity, from the top to the active site at the bottom, and in subsequent interactions along the binding site. Therefore, AChE could serve as ‘hosts’ for quaternary ligands or other cations containing aromatic groups. The presence of both aromatic benzene and oxazepine rings in benzoxazepines can facilitate π–cation interactions with AChE and, consequently, lower its concentration.^14^ However, the literature presents few examples on this subject. Derivatives of the Pyrazolo[1,5-*d*][1,4]benzoxazepin-5(6*H*)-one scaffold were evaluated for their *in vitro* inhibition of AChE and BChE, showing greater selectivity for BChE. The dihalogenated and 6-ethyl-substituted benzene derivatives were the most potent, with IC_50_ values of 2.95 and 2.04 µM, respectively.^15,16^ In the meantime, [1,4]benzoxazepine derivatives did not exhibit significant neuroprotective activity. However, the fused oxazoline-[1,4]benzoxazepine scaffold inhibited AChE by 81.6% at a concentration of 50 µg mL^−1^, whereas galantamine caused 87.7% inhibition of the enzyme activity.^17^

Sulfonamides are among the most targeted and extensively researched moieties and are considered essential pharmacophores in medicinal chemistry because of their versatile applications in biological and pharmacological fields.^18^ Sulfonamide-based compounds are particularly known as dual AChE inhibitors. In the literature, some have shown nanomolar inhibitory potency against AChE, such as pyrazoline-sulfonamides,^19^ polyfluoro-substituted pyrazoline sulfonamide derivatives,^20^ pyrrolidine-benzenesulfonamides,^21^ thiazole-sulfonamide derivatives,^22^ and *N*-phenylsulfonamides.^23^ Diaryltriazene-substituted sulfonamides showed excellent percentage inhibition against BChE (98.47%).^24^ Sulfonamides incorporating ketene *N*,*S*-acetal bioisosteres specifically target AChE expression and show a better inhibition profile than the standard drugs acetazolamide and tacrine,^25^ as do sulfonamide-bearing phenolic Schiff base derivatives,^26^ benzenesulfonamide derivatives incorporating 1,3,5-triazine structural motifs,^27^ and 1,2,3-triazole-sulfonamides.^28^ Sulfonamides have been found to form hydrogen bonds and interact with nonpolar environments within proteins. For example, the oxygen atoms in sulfonamide groups can form weak hydrogen bonds with the hydroxyl group of Tyr70 or the unionized form of Asp72.^29^ On the other hand, Trp286 interacts with the aromatic ring of benzenesulfonamide *via* π–π stacking and with the tosylate methyl group through π–alkyl interactions.^30–33^ Considering the promising effects of benzenesulfonamides and 1,4-benzoxazepine moieties reported in previous studies, and as part of our ongoing research, this article presents the design and synthesis of a new series of hybrid [1,4]-benzoxazepine-based sulfonamides (9a–12j) as inhibitors of AChE and BChE. These novel compounds were biologically evaluated *in vitro* for their inhibitory activity against AChE and BChE. A theoretical SAR analysis was conducted to support the experimental inhibitory activity data. Additionally, molecular docking studies were performed to elucidate the molecular-level inhibition mechanism and to determine the binding modes of the newly synthesized conjugates within the active sites of AChE and BChE.

## 2. Results and discussion

### 2.1 Chemistry

[1,4]Benzoxazepine-based sulfonamide hybrid derivatives 9–12a–j were synthesized straightforwardly by attaching various benzenesulfonyl groups to the nucleophilic nitrogen atom of [1,4]benzoxazepines *via* a nucleophilic substitution reaction. This was accomplished by reacting the precursors (4–7) with substituted sulfonyl chlorides (8a–j) in the presence of sodium hydride and anhydrous DMF at 0 °C, affording the target compounds in fair yields (Scheme 1).

**Scheme 1:**
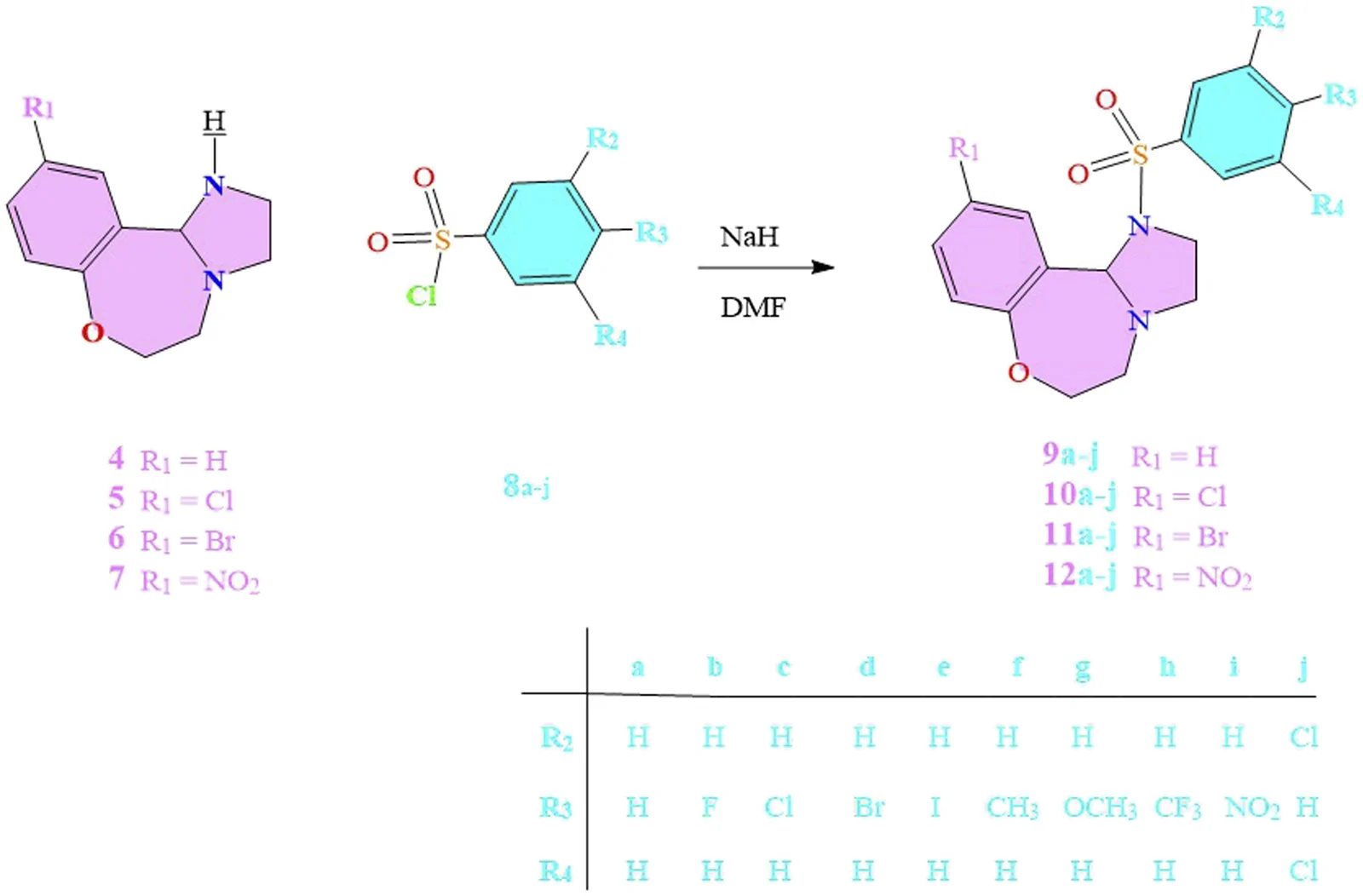
Synthesis of 1,4-benzoxazepine-based sulfonamide compounds 9–12a–j.

The structures of the desired [1,4]-benzoxazepine-based sulfonamide compounds (9–12a–j) were fully characterized by ^1^H and ^13^C NMR spectroscopy, as well as high-resolution mass spectrometry (HRMS). The mass spectra displayed molecular ion peaks consistent with the expected molecular weights, and the HRMS data were in good agreement with the calculated values. The spectral data, detailed in the “Experimental section and SI”, further support the proposed structures. All ^1^H NMR spectra exhibited a singlet signal at approximately *δ* 5.20 ppm, corresponding to the proton attached to the tertiary carbon atom of the (N–CH–N) segment that is located at the fused region of azepine and imidazoline rings. This carbon atom also resonated at around *δ* 78.3 ppm in its ^13^C NMR spectrum. On the other hand, all methylene protons associated with the 1,4-oxazepane and imidazolidine fused rings appeared as multiplets in the *δ* 2.0–4.8 ppm range, due to geminal and vicinal proton–proton coupling. The chemical shifts of their corresponding carbon atoms were observed at *δ* 47.6, 52.8, 54.2, and 71.3 ppm in the ^13^C NMR spectra. The disappearance of the singlet signal corresponding to the amino proton of the imidazoline ring in precursors 4–7, which appeared at *δ* 2.4 ppm, confirms the formation of the target [1,4]benzoxazepine-based sulfonamide derivatives 9–12a–j.

### 2.2 Discussion of the crystal structure of compound 11f

The compound 11f crystallizes in the monoclinic space group *P*2_1_/*n*. The asymmetric unit contains one molecule of C_18_H_19_BrN_2_O_3_S, as illustrated in Fig. 3. The crystal structure of 11f (C_18_H_19_BrN_2_O_3_S) features a highly functionalized framework centered around a sulfonamide linkage. The molecular geometry is defined by a central sulfur atom (S1) adopting a distorted tetrahedral coordination, characterized by short S=O double bonds *d*_S1–O2_ = 1.431(2) Å; *d*_S1–O3_ = 1.432(2) Å) and a notable expansion of the O2–S1–O3 angle to 118.0(4)° due to lone-pair repulsions. The aromatic portion is marked by a bromine atom at the C1 position with a bond distance of 1.900(3) Å. At the same time, the heterocyclic core displays an N1–C9–N2 angle of 123.2(4)°, indicating a wide connectivity between the nitrogen centers. The molecule's non-planar conformation is governed by the sp^3^-hybridized O1 atom, which links the brominated aryl ring to the rest of the scaffold through an O1–C7 bond of 1.451(4) Å and an O1–C4 bond of 1.374(3) Å. Torsion angle analysis reveals significant twisting, such as the C12–S1–N2–C9 angle of −70.4(2)°, which prevents a fully conjugated system but optimizes solid-state packing. All bond distances and angles are shown in Table 1 and agree with those in other.^34^

**Fig. 3:**
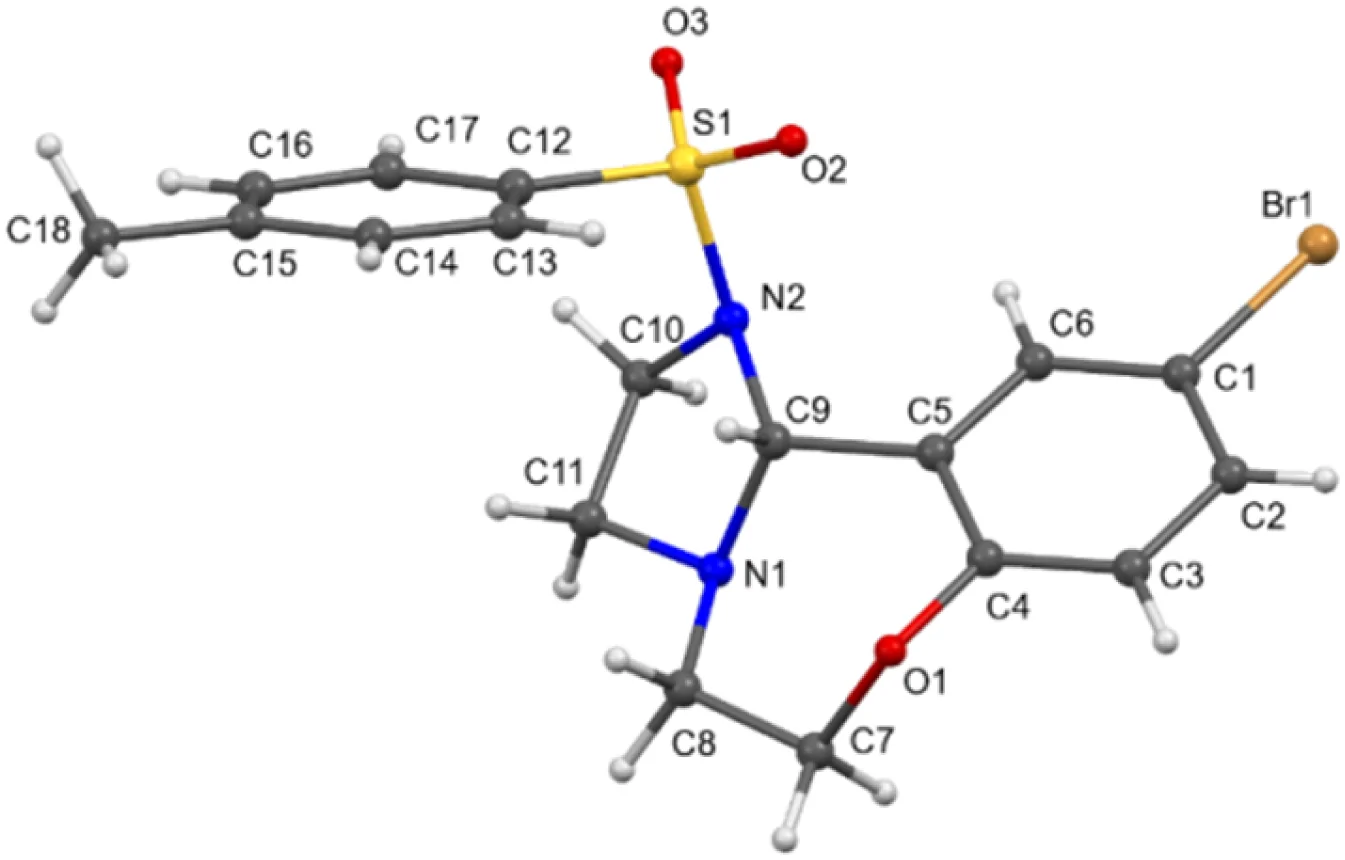
Labeled thermal ellipsoid (35%) of 11f, only hydrogens are not labeled for clarity purposes.

Some proper bond distances (Å) and angles (°) of 11f

**Table:** 

Bond	Distance (Å)	Bond	Distance (Å)
Br1–C1	1.900(3)	S1–O3	1.432(2)
S1–N2	1.633(3)	S1–O2	1.431(2)
S1–C12	1.764(3)	O1–C4	1.374(3)
O1–C7	1.451(4)	C9–N2	1.474(3)
C9–C5	1.513(4)	C9–N1	1.468(4)
N2–C10	1.490(4)	C15–C18	1.509(5)

**Table:** 

Angle	Degree (°)	Angle	Degree (°)
O2–S1–O3	118.0(4)	C4–O1–C7	118.5(4)
O3–S1–N2	124.4(4)	N1–C9–N2	123.2(4)
O2–S1–N2	121.8(4)	C9–N2–S1	120.2(4)
O3–S1–C12	123.4(4)	C10–N2–S1	122.7(5)
N2–S1–C12	120.2(3)	C2–C1–Br1	115.3(4)

This specific stereochemical arrangement, including the puckering of the heterocyclic ring (where N2–C10–C11 is 102.7(3)° and N1–C11–C10 is 102.1(3)°), facilitates a robust intermolecular network of C–H⋯O and C–H⋯Br interactions. A dense network of non-covalent interactions governs the supramolecular assembly and stabilizes the lattice. Hirshfeld surface analysis reveals that these interactions are dominated by C–H⋯O hydrogen bonds (accounting for 21.8% of the surface) and C–H⋯Br halogen contacts (12.5%) with C⋯Br distance of 3.596 Å, which appears as intense red spots on the normalized contact distance *d*_norm_ map (Fig. 4). This type of interaction is well-known in literature and shown in other systems.^35^

**Fig. 4:**
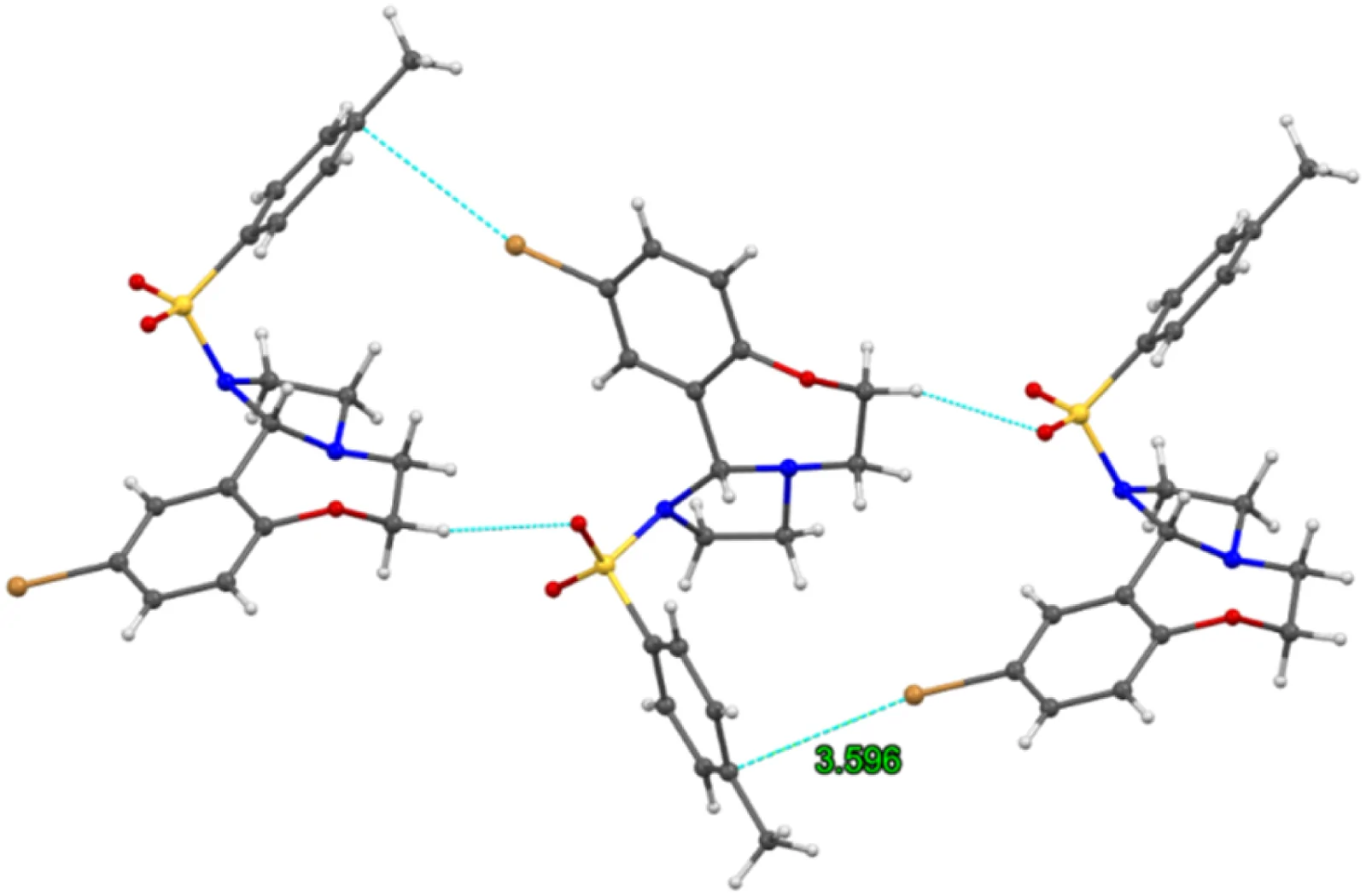
The meaningful intermolecular interactions in compound 11f.

### 2.3 Biological activity

#### 2.3.1 Anticholinesterase activity

Cholinesterase inhibitors are essential in the pharmaceutical management of neurodegenerative illnesses, such as AD; yet, the search for compounds with improved potency and selectivity continues. The development of dual-acting inhibitors that target both AChE and BChE is a promising approach to enhance clinical outcomes, particularly in later stages of the illness, when BChE activity is higher.

Cholinesterase inhibitors are essential in the pharmaceutical management of neurodegenerative illnesses, such as AD; yet, the search for compounds with improved potency and selectivity continues. Developing dual-acting inhibitors that target both AChE and BChE is a promising approach to improve clinical outcomes, particularly in later stages of the illness, when BChE activity is higher.

AChE and equine serum BChE are considered good model enzymes due to their structural homology with the human AChE and BChE isoforms, as well as their availability and cost-effectiveness.^36^

In this study, the [1,4]Benzoxazepine-based sulfonamide hybrid derivatives 9–12a–j were screened for their potential to affect AChE and BChE function using Ellman's spectrophotometric method.^16,37^ Their inhibitory activity values are reported in Table 2. Structure–activity relationship (SAR) analysis indicated that specific substituents on both the benzenesulfonyl and benzoxazepine moieties significantly modulated ChE inhibition.

**Table 2:** *In vitro* inhibition and selectivity of AChE and BChE of synthesized compounds **9–12a–j**. Data represent the mean ± SEM of 3 independent experiments performed in triplicate

No.	R_1_	R_2_	R_3_	R_4_	AChE (mean ± SEM)	BChE (mean ± SEM)	(SI)
9a	H	H	H	H	89 ± 0.60	91 ± 0.68	0.98
9b	H	H	F	H	81 ± 1.25	82 ± 0.74	0.99
9c	H	H	Cl	H	56 ± 2.20	82 ± 1.58	0.68
9d	H	H	Br	H	87 ± 2.19	100 ± 1.33	0.87
9e	H	H	I	H	40 ± 0.43	58 ± 0.38	0.69
9f	H	H	CH_3_	H	79 ± 4.56	97 ± 0.52	0.81
9g	H	H	OCH_3_	H	58 ± 1.89	87 ± 0.36	0.64
9h	H	H	CF_3_	H	43 ± 1.28	67 ± 0.43	0.64
9i	H	H	NO_2_	H	50 ± 0.60	57 ± 1.37	0.88
9j	H	Cl	H	Cl	72 ± 0.49	95 ± 0.29	0.76
10a	Cl	H	H	H	61 ± 0.92	82 ± 0.51	0.74
10b	Cl	H	F	H	71 ± 0.66	80 ± 0.33	0.89
10c	Cl	H	Cl	H	71 ± 1.31	91 ± 0.33	0.78
10d	Cl	H	Br	H	60 ± 2.10	75 ± 0.89	0.8
10e	Cl	H	I	H	56 ± 7.82	83 ± 0.95	0.67
10f	Cl	H	CH_3_	H	54 ± 5.33	82 ± 0.39	0.66
10g	Cl	H	OCH_3_	H	53 ± 6.82	96 ± 0.60	0.55
10h	Cl	H	CF_3_	H	46 ± 2.81	56 ± 1.10	0.82
10i	Cl	H	NO_2_	H	60 ± 6.46	52 ± 1.38	1.15
10j	Cl	Cl	H	Cl	57 ± 3.92	80 ± 0.64	0.71
11a	Br	H	H	H	73 ± 3.23	36 ± 1.87	2.03
11b	Br	H	F	H	26 ± 2.41	90 ± 0.88	0.29
11c	Br	H	Cl	H	3 ± 0.45	78 ± 0.71	0.038
11d	Br	H	Br	H	6 ± 1.44	87 ± 0.9	0.069
11e	Br	H	I	H	2 ± 3.93	87 ± 0.13	0.023
11f	Br	H	CH_3_	H	47 ± 0.99	83 ± 1.42	0.57
11g	Br	H	OCH_3_	H	3 ± 3.46	78 ± 0.65	0.038
11h	Br	H	CF_3_	H	2 ± 1.11	38 ± 0.97	0.05
11i	Br	H	NO_2_	H	69 ± 0.52	59 ± 2.06	1.17
11j	Br	Cl	H	Cl	17 ± 3.49	90 ± 1.96	0.189
12a	NO_2_	H	H	H	67 ± 1.12	74 ± 1.38	0.91
12b	NO_2_	H	F	H	81 ± 1.18	91 ± 0.85	0.89
12c	NO_2_	H	Cl	H	74 ± 1.10	96 ± 0.77	0.77
12d	NO_2_	H	Br	H	66 ± 1.86	64 ± 1.81	1.03
12e	NO_2_	H	I	H	70 ± 1.03	81 ± 1.44	0.86
12f	NO_2_	H	CH_3_	H	10 ± 2.52	94 ± 1.55	0.106
12g	NO_2_	H	OCH_3_	H	67 ± 1.26	92 ± 1.09	0.73
12h	NO_2_	H	CF_3_	H	76 ± 0.08	95 ± 0.72	0.8
12i	NO_2_	H	NO_2_	H	90 ± 1.37	80 ± 1.53	1.13
12j	NO_2_	Cl	H	Cl	84 ± 0.68	98 ± 0.51	0.857
Tacrine					95 ± 0.64	100 ± 0.67	0.95

The tested benzoxazepine-based sulfonamide compounds 9–12a–j inhibited both AChE and BChE, with inhibition values ranging from 2% to 90% for AChE and from 36% to 100% for BChE. Interestingly, most of the targeted benzoxazepine-based sulfonamides 9–12a–j showed higher BChE inhibition than AChE at the screening concentration, suggesting this scaffold may prefer BChE. The BChE inhibition results showed that all derivatives exhibited activity above 70%, except derivatives 9e, 9h, 9i, 10h, 10i, 11a, 11h, 11i, and 12d. In contrast, the inhibitory results against AChE indicated that all derivatives 9a–12j showed activity below 70%, except for compounds 9a, 9b, 9d, 9f, 9j, 10b, 10c, 11a, 12b, 12c, 12e, 12h, 12i, and 12j. Among these, compounds 9a, 9d, 12i, and 12j showed the highest preliminary AChE inhibition at the screening concentration. It is worth noting that the introduction of strongly electron-withdrawing groups, such as trifluoromethyl (CF_3_) or nitro (NO_2_), at the para-position of the benzenesulfonyl moiety, as observed in compounds 9h, 10h, 11h, 9i, 10i, and 11i, was generally associated with a reduction in inhibitory activity against both AChE and BChE. This observation differs from previous reports^38,39^ in which the incorporation of such substituents was associated with enhanced cholinesterase inhibitory activity. The different activity trends in the present series suggest that the effect of an electron-withdrawing substituent depends strongly on its molecular environment, including the chemical scaffold and its position within the molecule. Thus, rather than exerting an inherently activity-enhancing effect, CF_3_ and NO_2_ substituents may influence cholinesterase inhibition differently depending on their position and the inhibitor's surrounding structural features. These findings highlight the importance of considering the overall molecular context when interpreting substituent effects in cholinesterase inhibitor design.

In the current study, however, the decreased activity suggests that, within this scaffold, electron-withdrawing groups may negatively influence the overall binding conformation or interfere with key active-site interactions. For instance, these groups might alter the orientation of the benzenesulfonyl moiety, disrupt hydrogen bonding, or increase steric hindrance, thereby reducing the compound's ability to fit efficiently into the enzyme's catalytic site. Interestingly, compounds 12h and 12i were exceptions to this trend, as they contain an additional strongly electron-withdrawing nitro group on the benzene ring of the benzoxazepine core. Unlike the other derivatives, these compounds exhibited notable dual inhibitory activity against both AChE and BChE. This suggests that the position and context of electron-withdrawing group placement play a critical role in modulating activity. In 12h and 12i, the nitro group on the benzoxazepine scaffold may enhance π–π stacking or other favorable interactions within the enzyme's peripheral or catalytic sites, thereby compensating for the reduced activity conferred by the substituted benzenesulfonyl group. This finding is consistent with previous reports.^39^

Notably, the unsubstituted derivative 9a of both the sulfonated and benzoxazepine cores exhibited dual inhibition of both enzymes, with inhibition potencies approaching 90%. This performance exceeded that of other derivatives bearing electron-donating or electron-withdrawing substituents on the two benzene rings.

Introducing either electron-donating or electron-withdrawing substituents on the benzenesulfonyl group or the azepine core of bromobenzoxazepine-based derivatives 11a–j, except for 11a and 11i, dramatically reduced their inhibitory potency against AChE. In conclusion, our work clarifies a key design idea for ChE inhibitors: substituent effects are context-dependent. The unsubstituted molecular scaffold provided a favorable baseline for dual inhibition in this series, whereas electron-withdrawing groups may be strategically positioned to modulate activity. These findings challenge simple structure–activity theories and improve inhibitor development. Future research will focus on crystallographic studies to clarify the precise binding mechanisms of lead compounds within the active sites of AChE and BChE. This would validate the proposed interactions and steric conflicts, aiding in structure-based optimization. Furthermore, evaluating the most effective dual inhibitors in cellular and in *vivo* models of neurodegeneration is essential to translate these molecular findings into a feasible treatment strategy. This research produces a series of novel chemical entities and lays a conceptual groundwork for their subsequent development.

The compounds selected for IC_50_ determination (9a, 9b, 9d, 12b, **12i**, and 12j) were chosen based on their high inhibition (>80%) of AChE and/or BChE in the preliminary screening assay (Table 3) and their distinct inhibitory profiles. The selected compounds were derivatives with substantial cholinesterase inhibition and varying degrees of BChE preference, allowing quantitative assessment of their potency and selectivity.

**Table 3:** IC_50_ values for AChE and BChE inhibition (µM ± SEM) with selectivity index (SI = IC_50_ (AChE)/IC_50_ (BChE)

No.	AChE IC_50_ (µM) ± SEM	BChE IC_50_ (µM) ± SEM	Selectivity index (SI)
Tacrine	0.0367 ± 0.0044	0.0210 ± 0.0037	1.75
9a	9.04 ± 0.66	3.08 ± 0.37	2.94
9b	8.61 ± 1.88	4.64 ± 0.48	1.86
9d	62.45 ± 16.0	4.18 ± 0.56	14.94
12b	35.06 ± 5.70	14.98 ± 2.66	2.34
12i	64.03 ± 17.1	16.01 ± 3.15	4
12j	10.99 ± 2.04	1.06 ± 0.11	10.37

Analysis of the IC_50_ values revealed that compounds 9a and 9b exhibited comparable inhibitory potency against both AChE and BChE, with AChE IC_50_ values of 9.04 and 8.61 µM and BChE IC_50_ values of 3.08 and 4.64 µM, respectively. These findings suggest that the unsubstituted and *para*-fluoro-substituted benzenesulfonyl moieties can inhibit both enzymes. In contrast, compound 9d, bearing a *para*-bromo substituent, showed markedly reduced AChE potency (IC_50_ = 62.45 µM) while retaining substantial BChE inhibitory activity (IC_50_ = 4.18 µM), yielding the highest BChE selectivity index (SI = 14.94) among the evaluated compounds. This observation suggests that the bulkier bromine substituent may fit better within the BChE active site, although further structural evidence is needed to confirm this hypothesis.

Among the nitro-substituted derivatives, 12j emerged as the most potent BChE inhibitor (IC_50_ = 1.06 µM), while retaining measurable AChE inhibitory activity (IC_50_ = 10.99 µM), resulting in pronounced BChE preference (SI = 10.37). Collectively, these findings indicate that substituent effects on cholinesterase inhibition depend on their nature, position, and surrounding molecular framework. Whereas the unsubstituted and *para*-fluoro-substituted derivatives 9a and 9b showed appreciable inhibition of both enzymes, certain bulkier or nitro-substituted derivatives, particularly 9d and 12j, showed greater BChE preference (Fig. 5–7).

**Fig. 5:**
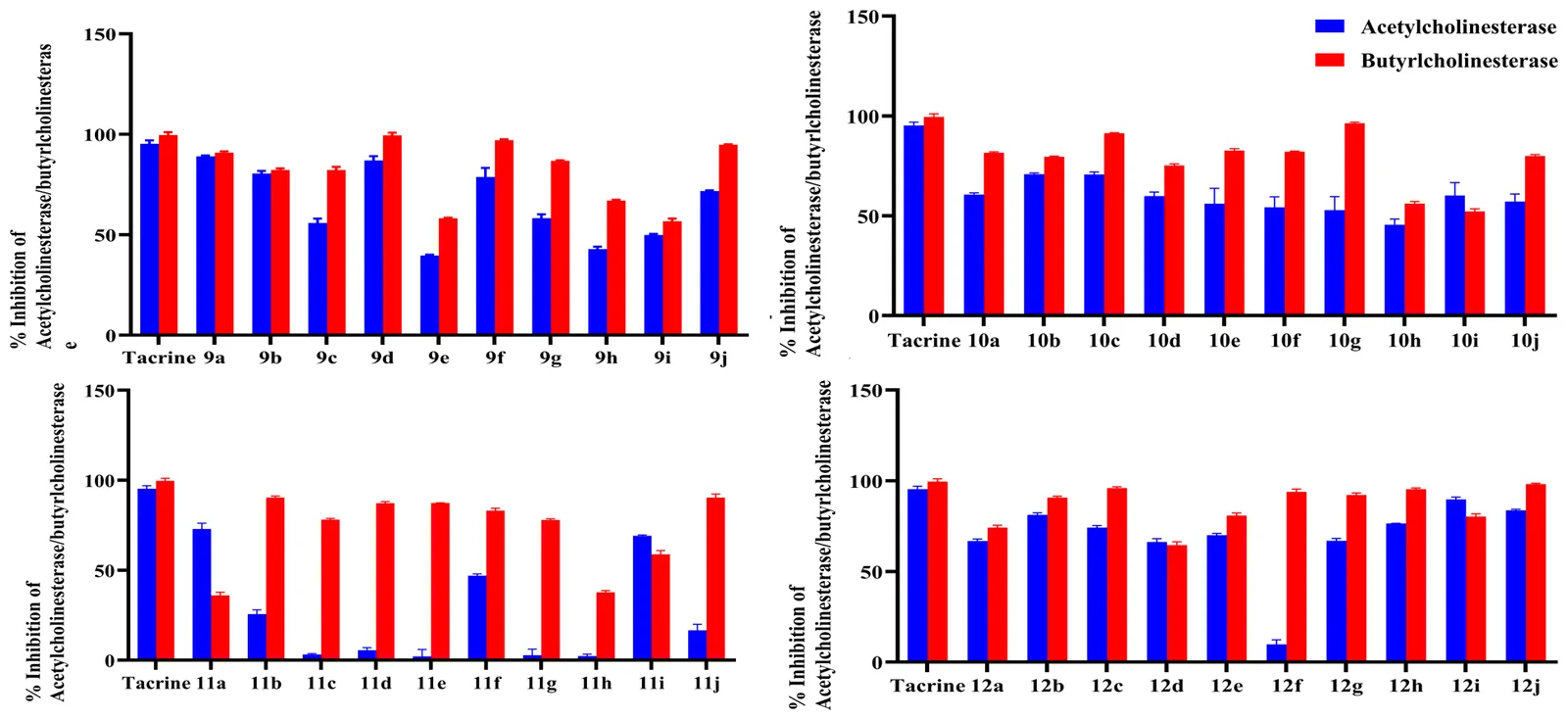
% inhibition of synthetic compounds against AChE and BChE. Data represent the mean ± SEM of 3 independent experiments performed in triplicate.

**Fig. 6:**
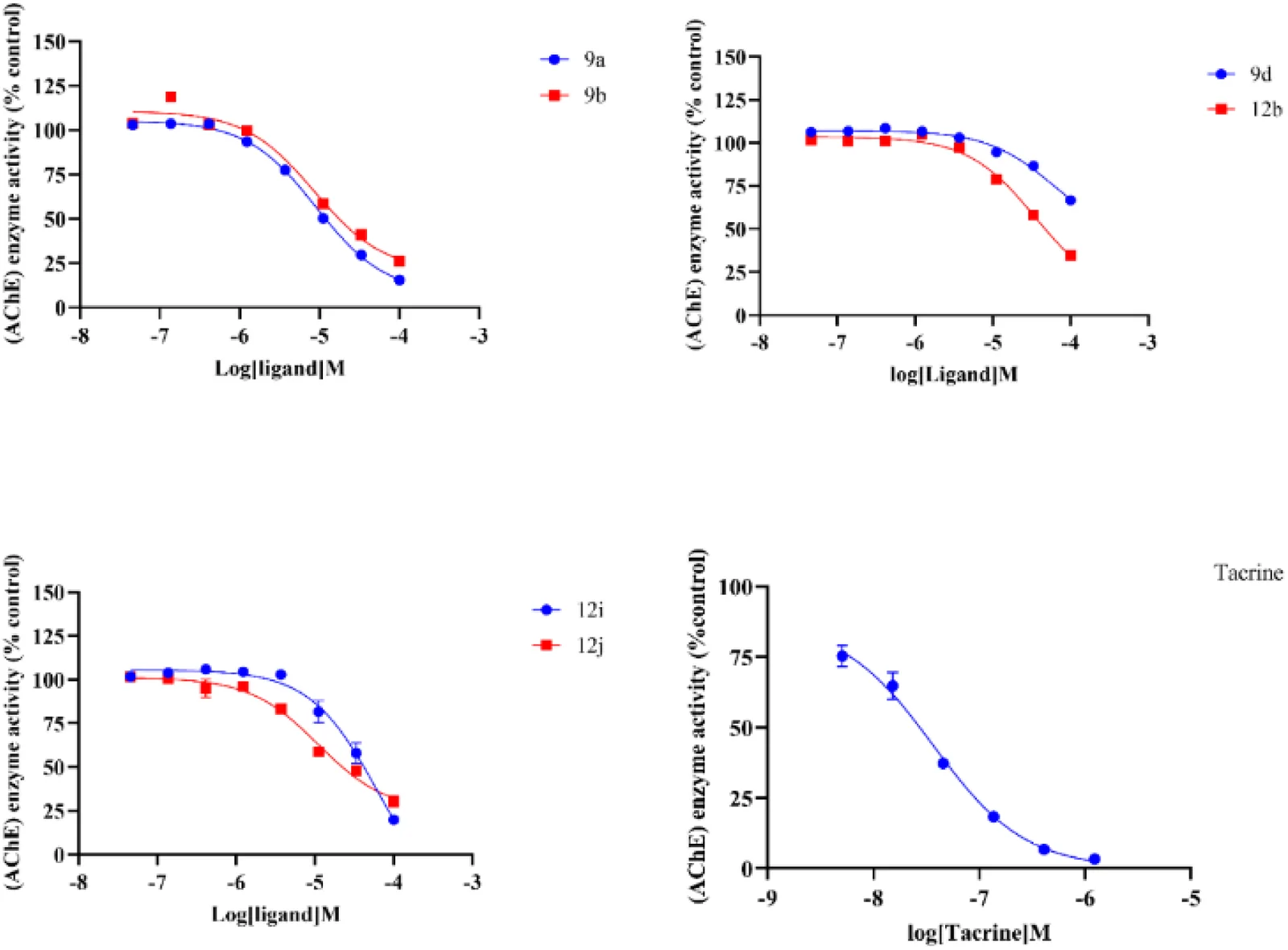
Concentration–response curves of selected compounds against AChE. IC_50_ values were determined by nonlinear regression analysis with a three-parameter logistic model. Data represent the mean ± SEM of 3 independent experiments performed in triplicate.

**Fig. 7:**
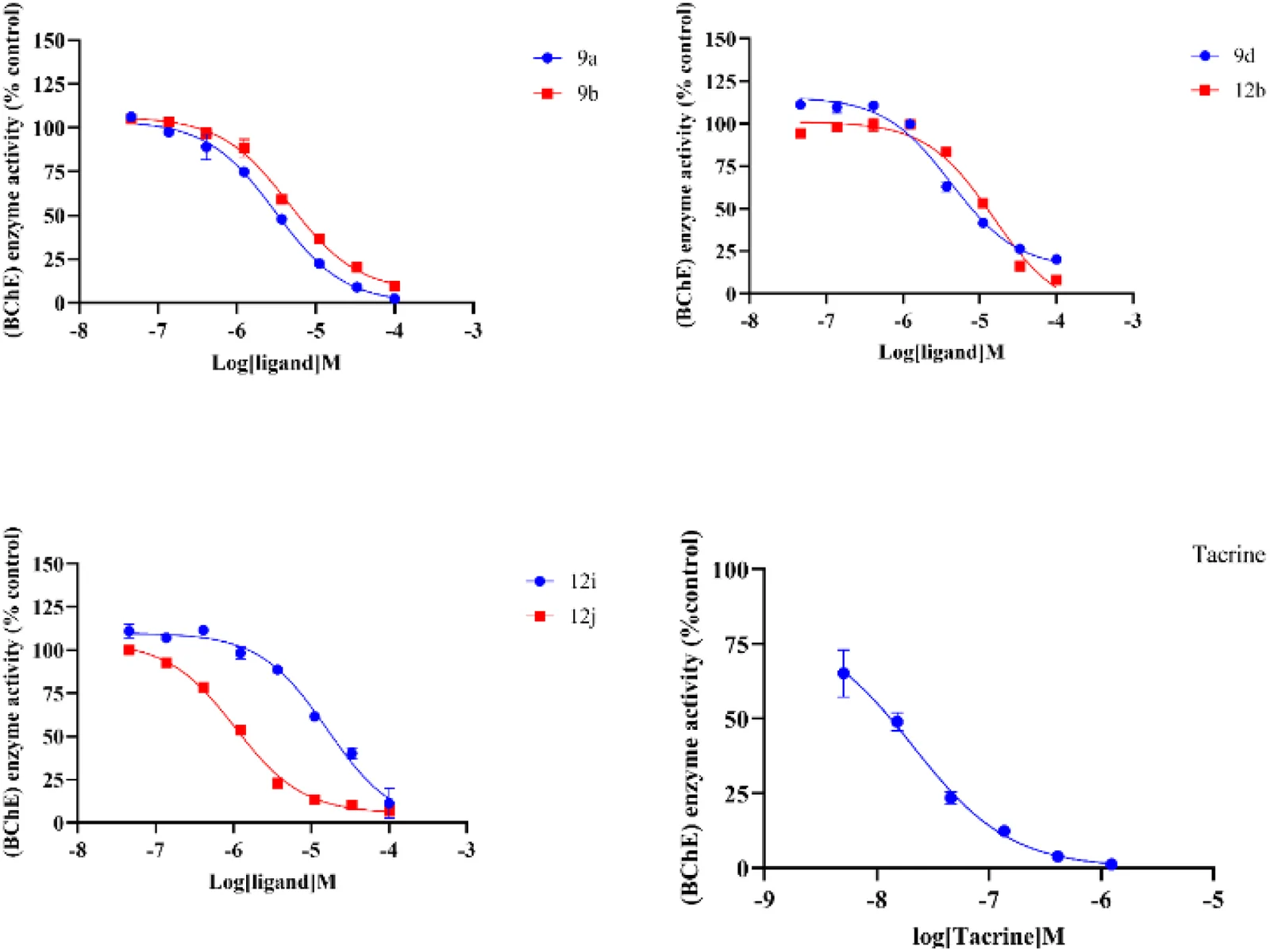
Concentration–response curves of selected compounds against BChE. IC_50_ values were determined by nonlinear regression analysis with a three-parameter logistic model. Data represent the mean ± SEM of 3 independent experiments performed in triplicate.

#### 2.3.2 Biological evaluation and Structure–Activity relationship (SAR)

The synthesized [1,4]benzoxazepine-based sulfonamide hybrid derivatives 9–12a–j exhibited a diverse spectrum of inhibitory activity against AChE and BChE, with selectivity strongly modulated by substituent type and position (R_1_–R_4_). Tacrine showed the highest inhibition toward both targets (AChE: 95%, BChE: 100%) and served as a positive reference. The unsubstituted parent scaffold exhibited pronounced dual ChE inhibition, with compound 9a showing high activity against both AChE (89%) and BChE (91%), indicating a favorable baseline fit within the active sites of both enzymes. Introducing halogen substituents at the R_3_ position selectively enhanced BChE inhibition, as shown by the bromo analog 9d, which achieved complete BChE inhibition (100%) while largely retaining AChE activity (87%). This enhancement is consistent with improved steric and hydrophobic complementarity within the broader BChE active site. In contrast, incorporating strongly electron-withdrawing groups such as NO_2_ or CF_3_ markedly reduced inhibitory activity toward both enzymes, suggesting diminished electronic compatibility and weaker interactions with key catalytic residues. Methoxy substitution partially restored BChE activity while reducing AChE inhibition, highlighting the distinct electronic and steric requirements governing enzyme selectivity.

Introduction of a chloro substituent at R_1_ generally preserved moderate to high BChE inhibition (75–96%) but was accompanied by a noticeable decrease in AChE activity (46–71%), indicating that chloro substitution disfavors optimal accommodation within the narrower AChE site. Among these analogs, methoxy substitution at R_3_ afforded the most balanced inhibition profile, yielding high BChE potency (96%) and moderate AChE inhibition (53%), supporting a role for electron-donating groups in partially compensating for steric or electronic penalties imposed by the chloro scaffold. Conversely, nitro substitution again led to reduced inhibition of both enzymes, reinforcing the unfavorable impact of strongly electron-withdrawing substituents in this context. Replacement of R_1_ with bromine produced a marked shift toward BChE selectivity. Several brominated derivatives demonstrated strong BChE inhibition (78–90%) while exhibiting minimal AChE activity (<20%), consistent with steric exclusion from the more restrictive AChE active site. Notably, the iodo-substituted analog showed near-complete loss of AChE inhibition (2%) yet retained high BChE potency (87%), underscoring the ability of bulky halogens to exploit the spatial tolerance of the BChE site. Only a limited number of brominated analogs maintained moderate AChE inhibition, suggesting that precise substituent orientation and electronic balance are required to overcome steric constraints in AChE binding. Overall, this scaffold showed the highest BChE selectivity among the evaluated compounds. In contrast, nitro substitution at R_1_ generated several highly active derivatives in the preliminary screening assay. Selected analogs achieved high inhibition of both AChE (84–90%) and BChE (80–98%). These results suggest that the nitro group may favorably modulate electronic distribution, potentially enhancing π–π stacking and stabilizing interactions within both catalytic pockets. Nevertheless, the poor AChE inhibition observed for certain analogs despite strong BChE activity indicates that steric alignment and conformational positioning remain critical determinants for effective engagement of the AChE active site. Taken together, the SAR trends indicate that certain bulky halogen substituents, particularly bromine in selected scaffolds, favored BChE inhibition, although this effect was strongly scaffold-dependent. Electron-donating substituents generally support dual or BChE-favored inhibition depending on the core scaffold, whereas strongly electron-withdrawing groups tend to compromise AChE activity, with notable scaffold-dependent exceptions. These findings highlight the importance of balancing steric demand and electronic complementarity to achieve either selective BChE inhibition or potent dual ChE activity. Evaluation across all compound series reveals a clear SAR, in which unsubstituted and nitro-substituted cores afford balanced dual ChE inhibition. In contrast, halogenated scaffolds, particularly brominated analogs 11a–j, exhibit a pronounced bias toward BChE inhibition, consistent with steric discrimination between the AChE and BChE catalytic sites. Notably, the observed IC_50_ values showed some correspondence with selected docking observations; however, the relationship was inconsistent across the series, and the docking results are interpreted as supportive rather than as quantitative measures of inhibitory potency. Compounds such as 9a and 9b showed stable π–π stacking and hydrogen-bonding interactions with key residues, supporting their balanced dual inhibitory activity. In contrast, compound 9d, which showed high BChE selectivity, displayed enhanced hydrophobic interactions within the larger BChE binding pocket, consistent with its lower IC_50_ value against BChE. Similarly, compound 12j showed a high BChE LibDock score (147.32), which provides some structural support for its potent BChE inhibition. The activity trends are regarded as preliminary observations rather than definitive SAR rules. Further optimization with a wider range of electron-donating, electron-withdrawing, sterically demanding, and hydrophobic substituents is needed to define the structural requirements for enhanced cholinesterase inhibition.

### 2.4 Molecular modeling

#### 2.4.1 Molecular docking studies

Molecular docking studies were performed using the CDocker and LibDock algorithms against human AChE (PDB ID: 4EY7) and BChE (PDB ID: 7QHD), as previously reported.^40,41^ Higher LibDock scores and more negative CDocker interaction energies were interpreted as indicative of enhanced ligand–enzyme complementarity and increased binding stability. Redocking of the co-crystalized ligand is performed to validate the docking process, and the RMSD is calculated to be less than 2.0 Å (0.36 Å and 1.57 Å, respectively), supporting the ability of the docking procedures to reproduce the experimentally observed binding poses.

Across the evaluated compounds, several structure–binding trends were observed. Substituents that increase hydrophobic character, including bromine and methyl groups, were associated with favorable predicted interactions in selected derivatives and, in some cases, with appreciable cholinesterase inhibition. Similarly, selected nitro-containing derivatives of the 12-series exhibited favorable docking scores and interaction energies, consistent with their experimentally observed inhibitory activity. Brominated derivatives also showed favorable predicted interactions within the BChE binding site in several cases, which may reflect the larger BChE active site accommodating bulkier substituents. However, this proposed steric contribution remains a structural hypothesis and requires experimental structural validation.

Overall, the docking results provided a structural rationale for selected experimental SAR observations, including the greater BChE preference observed for compounds such as 9d and 12j. Although compounds with favorable LibDock scores and/or more negative CDocker interaction energies often showed appreciable enzymatic inhibition, the present data do not establish a quantitative correlation between docking scores and experimental potency. Therefore, the docking results support the proposed binding modes rather than directly measuring binding affinity or stability.

Among compounds showing relatively stronger AChE inhibition, selected derivatives such as 10i and 11i exhibited favorable values for at least one docking metric against AChE. Compound 10i showed a notably negative CDOCKER interaction energy (−24.07) and an AChE LibDock score of 134.51, supporting a favorable predicted interaction with AChE. Compound 11i also showed a negative CDOCKER interaction energy (−17.00) and an AChE LibDock score of 131.18.

Compounds showing relatively balanced inhibition of both ChE targets did not always display concordant docking results for both enzymes. For example, 9b showed a high AChE LibDock score (134.12) and near-equipotent preliminary inhibition of AChE and BChE (81% and 82%, respectively); however, Table 4 reports BChE CDOCKER and LibDock values of 18.71 and 128.34, respectively, indicating a less favorable CDOCKER interaction energy than for its AChE complex. Similarly, 12d showed appreciable inhibition of both enzymes (66% AChE and 64% BChE), but its BChE CDOCKER interaction energy was positive (+17.05), despite a high BChE LibDock score (138.55). Several compounds showed relatively weak cholinesterase inhibition accompanied by poor docking results. For example, 9e exhibited relatively low inhibition of both AChE and BChE (40% and 58%, respectively) and had zero values for both docking metrics against both enzymes. Compounds 10f and 10j similarly showed zero AChE docking value, while 11a showed weak BChE inhibition (36%) and zero BChE LibDock score. Conversely, compounds such as 11c, 11e, 11g, 11h, and 12f showed low AChE inhibition despite moderate-to-favorable docking values, demonstrating that docking scores alone do not fully explain the observed enzymatic activity.

**Table 4:** Docking scores using LibDock and CDocker against AChE and BChE

No.	4EY7-CDocker	4EY7-LibDock	7QHD-CDocker	7QQHD-LibDock
9a	13.4749	133.653	17.7516	121.84
9b	−10.4331	134.121	18.711	128.341
9c	3.40138	129.866	0	0
9d	6.66992	132.027	19.8059	120.299
9e	0	0	0	0
9f	6.66814	132.631	20.7245	129.221
9g	15.7193	0	0	0
9h	0	158.077	18.0969	143.207
9i	10.0021	148.634	0	0
9j	2.20728	121.339	20.7476	0
10a	6.41965	123.157	18.1698	122.274
10b	0	0	19.3326	0
10c	0	0	0	0
10d	−15.0072	97.7169	21.4808	135.834
10e	0	0	0	0
10f	0	0	21.49	0
10g	−21.8433	131.868	18.8849	131.607
10h	6.30528	143.869	20.5624	0
10i	−24.0654	134.506	17.855	135.941
10j	0	0	23.8212	0
11a	0	0	19.5067	0
11b	−35.6721	100.637	15.9907	137.016
11c	−22.3531	96.4743	16.5927	136.079
11d	0	97.8473	17.7358	0
11e	−16.901	87.092	20.7648	134.598
11f	−19.4125	83.6418	21.0686	0
11g	1.06798	132.806	19.7295	136.398
11h	−3.15644	138.813	22.2116	0
11i	−16.9991	131.18	18.6841	130.807
11j	−23.0827	109.603	19.4054	0
12a	−15.3489	135.663	16.9982	141.127
12b	−29.6972	140.101	13.8545	140.023
12c	−29.6658	138.653	15.715	130.308
12d	−26.7327	135.349	17.0467	138.554
12e	−24.1732	94.9166	17.2042	140.48
12f	−24.0892	140.066	15.9391	140.371
12g	3.72705	0	16.06	140.154
12h	−27.6356	110.196	17.6819	139.434
12i	−31.5136	97.8109	14.3251	151.134
12j	−11.5907	99.3771	17.7929	147.317
4ey7	30.5949	177.15	9.08511	151.55

#### 2.4.2 Comparative discussion: binding mode of compound 12i with ChE

Molecular docking analysis (Fig. 8 and 9) was performed to provide a structural interpretation of the experimental cholinesterase inhibition data. Representative interaction profiles for compounds 9a, 9b, 9d, 12b, 12i, and 12j are shown. The selected compounds adopted plausible binding orientations within the active-site cavities of AChE and BChE, involving combinations of aromatic, hydrophobic, electrostatic, and hydrogen-bonding interactions.

**Fig. 8:**
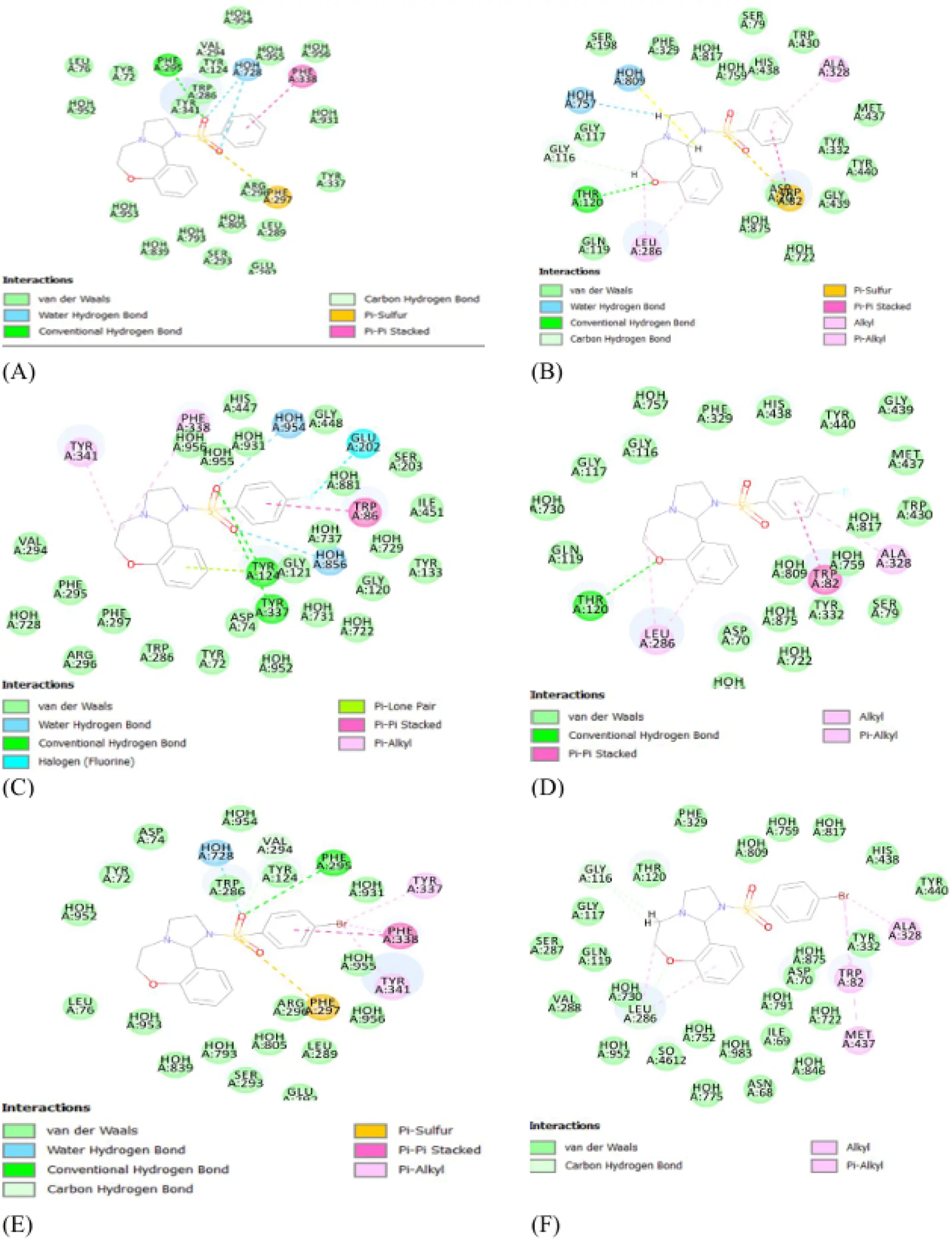
(A) Two-dimensional interaction profile of docked compound 9a with AChE (PDB ID: 4EY7, resolution: 2.35 Å). (B) Two-dimensional interaction profile of docked compound 9a into the binding site of BChE (PDB ID: 7QHD, resolution: 2.04 Å). (C) Two-dimensional interaction profile of docked compound 9b with AChE (PDB ID: 4EY7, resolution: 2.35 Å). (D) Two-dimensional interaction profile of docked compound 9b with BChE (PDB ID: 7QHD, resolution: 2.04 Å). (E) Two-dimensional interaction profile of docked compound 9d with AChE (PDB ID: 4EY7, resolution: 2.35 Å). (F) Two-dimensional interaction profile of docked compound 9d with BChE (PDB ID: 7QHD, resolution: 2.04 Å).

**Fig. 9:**
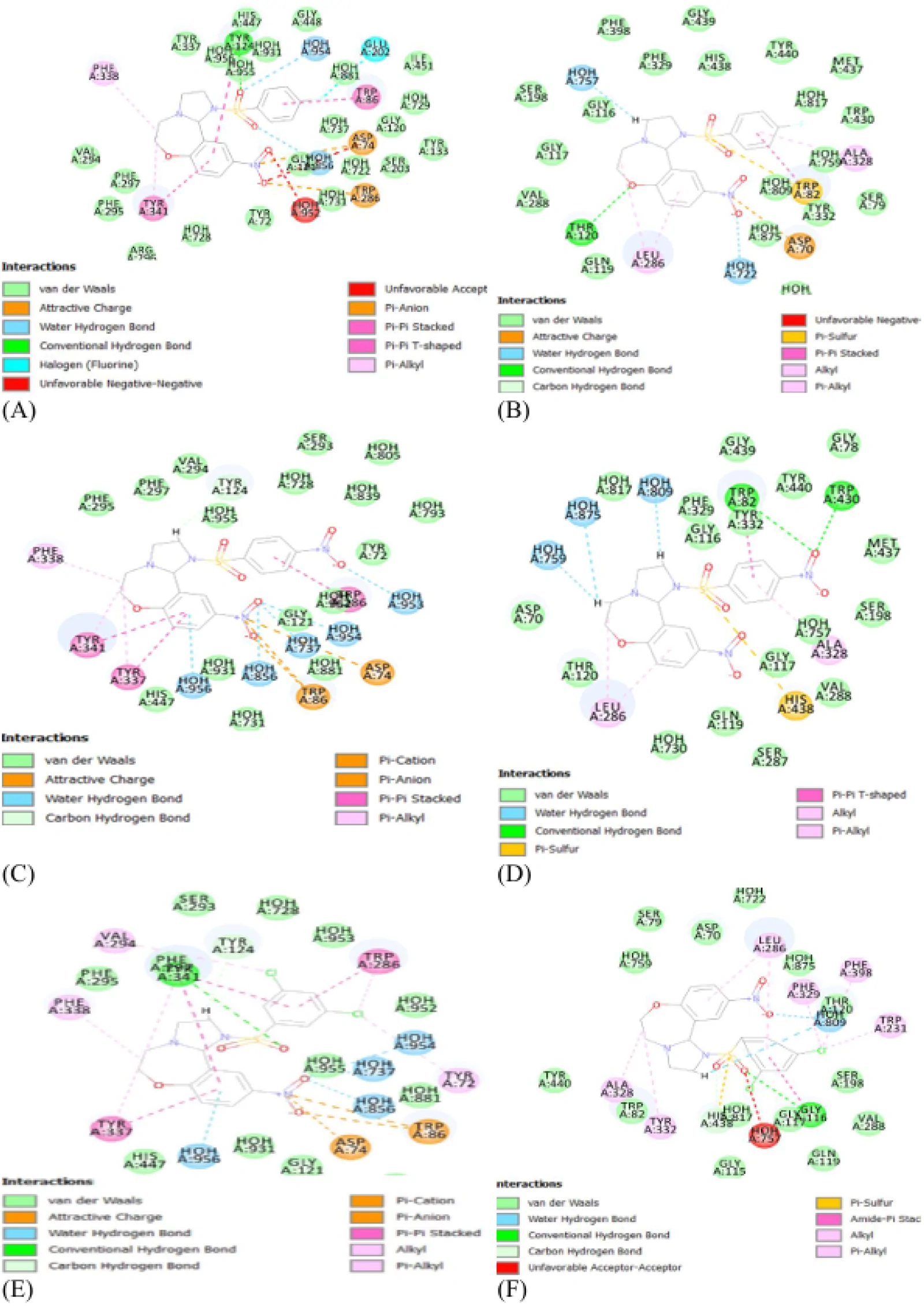
(A) Two-dimensional interaction profile of docked compound 12b with AChE (PDB ID: 4EY7, resolution: 2.35 Å). (B) Two-dimensional interaction profile of docked compound 12b into the binding site of BChE (PDB ID: 7QHD, resolution: 2.04 Å). (C) Two-dimensional interaction profile of docked compound 12i with AChE (PDB ID: 4EY7, resolution: 2.35 Å). (D) Two-dimensional interaction profile of docked compound 12i with BChE (PDB ID: 7QHD, resolution: 2.04 Å). (E) Two-dimensional interaction profile of docked compound 12j with AChE (PDB ID: 4EY7, resolution: 2.35 Å). (F) Two-dimensional interaction profile of docked compound 12j with BChE (PDB ID: 7QHD, resolution: 2.04 Å).

In AChE, the representative docked ligands were accommodated within the active site through combinations of aromatic and hydrophobic contacts. Residues such as Trp86, Phe297, Phe338, Tyr337, and Tyr341 contributed to ligand recognition through aromatic interactions. Hydrogen-bonding interactions were also observed in selected poses, including contacts involving Tyr124 and water-mediated interactions to stabilize the ligand. In BChE, the larger binding cavity permitted combinations of aromatic, hydrophobic, and hydrogen-bonding contacts involving residues such as Trp82, Thr120, Ala328, Leu286, Tyr332, Trp430, His438, and Met437 in representative complexes. Within the 9-series, compound 9b showed a clear π–π interaction involving Trp86 in AChE, together with additional hydrogen-bonding and hydrophobic contacts.

Compound 9d exhibited a favorable AChE interaction pattern involving Phe338 and additional aromatic/hydrophobic contacts. These observations suggest that substituent-dependent changes in aromatic and hydrophobic interactions may contribute to differences in recognition within the cholinesterase binding sites. The proposed BChE binding contribution of 9d is consistent with its experimentally observed BChE preference.

The selected 12-series representatives showed diverse interaction patterns. Compound 12b displayed multiple aromatic, hydrogen-bonding, and electrostatic contacts in representative AChE and BChE complexes. Compound 12i exhibited a dense AChE interaction network involving aromatic residues and water-mediated contacts, while its BChE complex showed multiple aromatic, hydrogen-bonding, and hydrophobic interactions. These observations indicate that the nitro-substituted scaffold can adopt different interaction patterns depending on the enzyme environment.

Compound 12j also displayed a diversified interaction profile. In AChE, the dichloro-substituted derivative engaged several aromatic and hydrophobic residues, together with additional electrostatic and hydrogen-bonding contacts in the representative pose. In BChE, 12j showed multiple interactions involving Trp82, Phe329, His438, and surrounding residues, including an amide–π interaction. Its experimentally determined BChE IC_50_ of 1.06 ± 0.11 µM indicates substantial BChE activity that comply with the docking BChE LibDock score (147.32).

Overall, the docking analysis provides a plausible structural rationale for selected experimental SAR observations, particularly the BChE preference of compounds such as 9d and 12j. The 12-series, especially 12i and 12j, displayed diverse interactions in the representative complexes.

Fig. 10 and Table 5 summarize the predicted ADMET properties of the most active compounds (9a, 9b, 9d, 12b, 12i, and 12j), providing important insights into their pharmacokinetic behavior and safety profiles. Correlating docking and biological activity with ADMET properties reveals a clearer picture. Although the dichlorobenzenesulfonamide analog 12j showed strong enzymatic inhibition and extensive binding interactions, its higher lipophilicity (*A* log *P* = 3.71), low daphnia EC_50_ (0.0273 mg L^−1^), and relatively low rat LD_50_ (235.2 mg kg^−1^) indicate a higher predicted toxicity risk. Similarly, compound 12i, despite favorable docking interactions, showed reduced intestinal absorption (level 1) and poor BBB permeability (level 4), likely because its high polar surface area (PSA = 135.88 Å^2^) exceeds the optimal threshold for CNS-active drugs. In contrast, among the benzoxazepine-based benzenesulfonamide analogs 9a–j, compounds 9a, 9b, and 9d demonstrated a more balanced profile. These compounds showed consistent binding interactions, particularly conserved π–π stacking with key tryptophan residues, and exhibited favorable ADMET characteristics, including good intestinal absorption (level 0), moderate-to-high BBB penetration (levels 1–2), and significantly lower predicted toxicity (higher EC_50_ and LD_50_ values). For instance, bromo-substituted benzenesulfonamide analogue 9d showed strong selectivity and the highest predicted safety margin (LD_50_ = 1100 mg kg^−1^), highlighting the beneficial role of bromine substitution in improving both binding and pharmacokinetic properties.

**Fig. 10:**
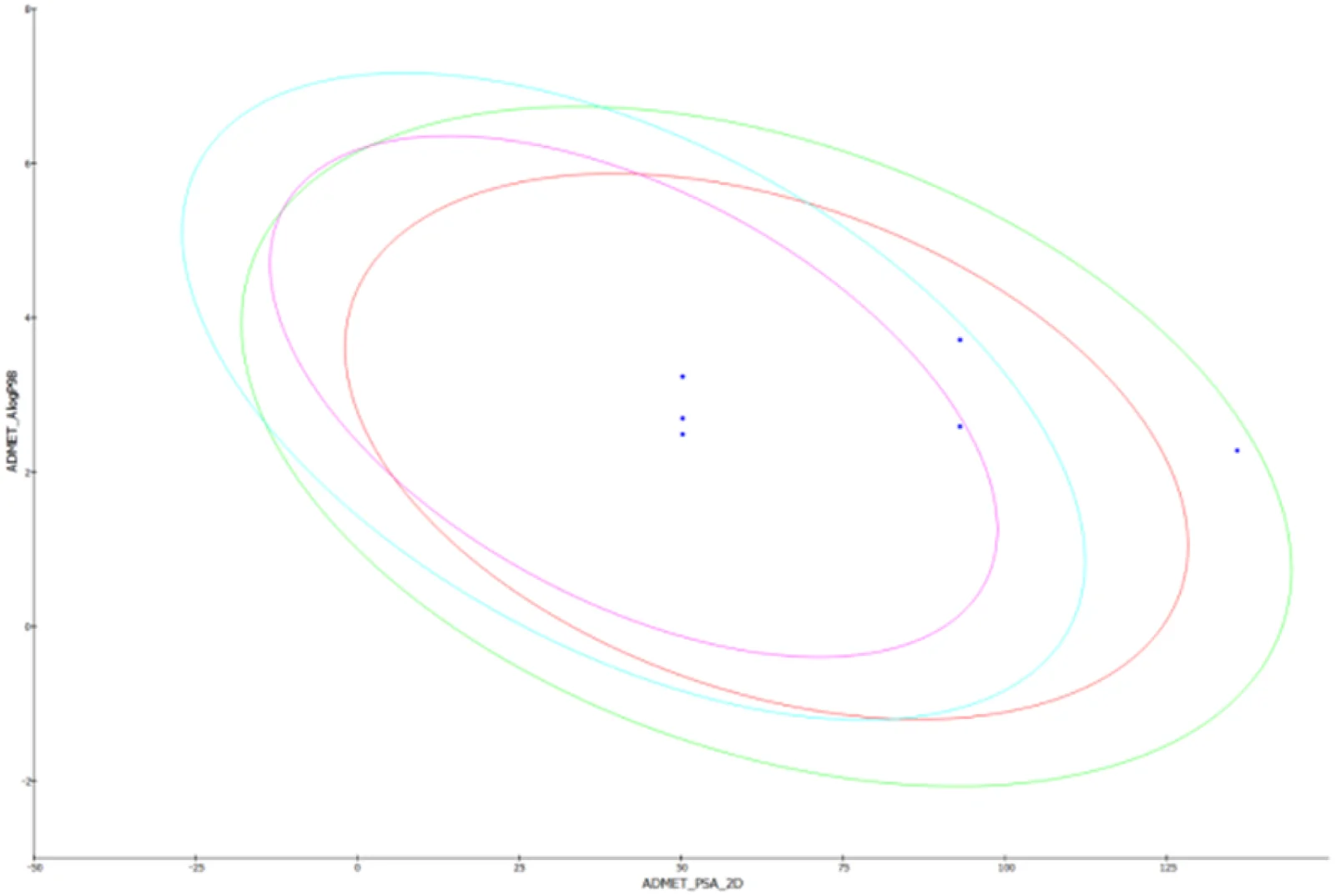
ADMET properties for the best active compounds 9a, 9b, 9d, and 12b, 12i, 12j.

**Table 5:** ADMET properties of the best active compounds

No.	ADMET solubility level^a^	ADMET BBB level^b^	ADMET absorption level^b^	*A* log P98	ADMET PSA 2D	Daphnia EC_50_ (mg L^−1^)^c^	Rat LD_50_ (mg kg^−1^)^d^
9a	2	2	0	2.489	50.236	20.7	948
9b	2	2	0	2.695	50.236	1.9	867.7
9d	f2	1	0	3.238	50.236	2.3	1100
12b	2	3	0	2.589	93.059	0.355	356.3
12i	2	4	1	2.278	135.882	1.1	328.7
12j	2	2	0	3.712	93.059	0.0273	235.2

a Notes: 0 (extremely low soluble), 1 (very low), 2 (low), 3 (moderate), 4 (optimum), 5 (too soluble).

b 0 (Very high), 1 (High), 2 (Medium), 3 (Low). 4 (very low), 5 (negligible).

c EC50; effective concentration that kills 50% of daphnia.

d LD_50_: lethal dose that kills 50% of rats.

Importantly, CNS drug development requires an optimal balance between lipophilicity, polarity, and safety. In this regard, the benzoxazepine-based benzenesulfonamide derivatives 9a–j, with moderate lipophilicity (*A* log *P* ≈ 2.5–3.2) and low PSA (∼50 Å^2^), are generally compatible with physicochemical ranges often considered favorable for CNS drug development, although actual brain exposure requires experimental confirmation. In contrast, the nitrobenzoxazepine-based benzenesulfonamide analogs 12a–j, while structurally more interactive at the molecular level, appear to exhibit increased polarity or toxicity, which may limit their translational potential.

This analysis highlights that biological potency alone is insufficient for lead selection, and compounds must be evaluated holistically. While 12j may represent a potent scaffold from a binding perspective, the benzoxazepine-based benzenesulfonamide derivatives 9a–j, particularly the unsubstituted 9a, fluoro-9b, and bromo-9d analogs, emerge as comparatively more promising lead candidates, offering a more favorable balance among enzymatic inhibition, pharmacokinetics, and predicted safety profiles. These findings emphasize the importance of incorporating ADMET considerations early in the drug design process to guide the development of effective and clinically viable cholinesterase inhibitors.

## 3. Experimental

### 3.1 Chemicals and instruments

Chromatographic separations were performed on silica gel columns (60–120 mesh, Fluka). Reaction progress was monitored by thin-layer chromatography (TLC) on silica gel sheets, with visualization under UV light when applicable. Melting points were measured using a Stuart Scientific melting point apparatus with open capillary tubes and are reported without correction. ^1^H NMR and ^13^C NMR spectra were recorded on a Bruker DPX-500 spectrometer at 500 MHz, using tetramethylsilane (TMS) as the internal standard. Chemical shift values (*δ*) are reported in parts per million (ppm), and ^1^H–^1^H coupling constants (*J*) are given in Hertz (Hz). High-resolution mass spectra (HRMS) were acquired in positive ion mode using electrospray ionization (ESI) on a Bruker APEX-4 (7 tesla) instrument. Samples were dissolved in acetonitrile, diluted with a spray solution (methanol–water 1 : 1, v/v, containing 0.1% formic acid), and introduced using a syringe pump at 2 µL min^−1^. External calibration was performed using an arginine cluster over a mass range of *m*/*z* 175–871.

### 3.2 General procedure for synthesis and characterization of compounds (9–12a–j)

To a stirred solution of a [1,4]benzoxazepine derivatives, 4–7 (0.95 mmol) in 5 mL of anhydrous DMF, sodium hydride (0.04 g, 1.43 mmol) was added at 0 °C. The reaction mixture was stirred at 0 °C for 15 minutes, then sulfonyl chloride derivatives (1.05 mmol) were added in portions. The reaction mixture was then stirred at room temperature for 2–3 hours. Cooled water was gradually added to the reaction mixture, followed by 1–2 mL of 10% aqueous NaOH, resulting in the formation of a precipitate. The precipitate was filtered and washed thoroughly with water. After drying, the solid was washed with about 5 mL of either diethyl ether or methanol.

#### 3.2.1 1-(Phenylsulfonyl)-1,2,3,5,6,11*b*-hexahydrobenzo[*f*]imidazo[1,2-*d*][1,4]oxazepine (9a)

The yellow solid was washed with about 5 mL of diethyl ether (77% yield), mp 120–122 °C. ^1^H NMR (500 Hz, CDCl_3_): *δ* = 1.99 (dd, *J* = 6.1, 9.9 Hz, 1H), 2.71 (dd, *J* = 2.7, 12.3 Hz, 1H), 2.82 (m, 1H), 2.89 (m, 1H), 3.59 (m, 1H), 3.74–3.80 (m, 1H), 4.30 (dt, *J* = 3.0, 12.3 Hz, 1H), 5.22 (s, 1H), 7.02 (dd, *J* = 1.0, 7.9 Hz, 1H), 7.15 (dt, *J* = 1.0, 7.5 Hz, 1H), 7.24 (dt, *J* = 1.0, 7.5 Hz, 1H), 7.59 (t, *J* = 7.5 Hz, 1H 2H), 7.66 (t, *J* = 7.5 Hz, 1H), 7.71 (d, *J* = 1.0, 7.1 Hz, 1H) 7.92 (dd, *J* = 1.0, 7.1 Hz, 2H). ^13^C NMR, (125 Hz, CDCl_3_): *δ* = 47.6, 53.3, 55.2, 71.7, 78.6, 121.2, 124.3, 126.3, 128.1, 129.4, 129.5, 133.3, 133.5, 137.5, 157.3. HRMS (ESI) *m*/*z*: calcd for [M + H] C_17_H_19_N_2_O_3_S mass 331.1116 found 331.1111.

#### 3.2.2 1-((4-Fluorophenyl)sulfonyl)-1,2,3,5,6,11*b*-hexahydrobenzo[*f*]imidazo[1,2-*d*][1,4]oxazepine (9b)

The colorless solid was washed with about 5 mL of methanol (82% yield), mp 139–140 °C. ^1^H NMR (500 Hz, CDCl_3_): *δ* = 2.09 (m, 1H), 2.75 (t, *J* = 10.7 Hz, 1H), 2.85 (m, 1H), 2.91 (d, *J* = 12.4 Hz, 1H), 3.61 (m, 1H), 3.72 (m, 1H), 3.79 (d, *J* = 10.7 Hz, 1H), 4.32 (d, *J* = 12.0 Hz, 1H), 5.22 (s, 1H), 7.01 (d, *J* = 7.5 Hz, 1H), 7.15 (t, *J* = 6.9 Hz, 1H), 7.22 (m, 3H), 7.67 (d, *J* = 7.0 Hz, 1H), 7.94 (m, 2H), ^13^C NMR, (125 Hz, CDCl_3_): *δ* = 47.6, 53.2, 55.0, 71.6, 78.6, 116.8 (d, ^3^*J*_C–F_ = 22.4 Hz), 121.2, 124.3, 126.3, 129.5, 130.8 (d, ^4^*J*_C–F_ = 9.32 Hz), 132.9, 133.7, 157.2, 165.7 (d, ^1^*J*_C–F_ = 255.8 Hz). HRMS (ESI) *m*/*z*: calcd for [M + H] C_17_H_18_N_2_O_3_SF, mass 349.1018; found 349.10167.

#### 3.2.3 1-((4-Chlorophenyl)sulfonyl)-1,2,3,5,6,11*b*-hexahydrobenzo[*f*]imidazo[1,2-*d*][1,4]oxazepine (9c)

The light yellow solid was washed with about 5 mL of diethylether (74% yield), mp 140–142 °C. ^1^H NMR (500 Hz, CDCl_3_): *δ* = 2.11 (m, 1H), 2.75 (m, 1H), 2.84 (m, 1H), 2.91 (d, *J* = 10.8 Hz, 1H), 3.60 (m, 1H), 3.70 (m, 1H), 3.79 (m, 1H), 4.32 (d, *J* = 9.8 Hz, 1H), 5.22 (s, 1H), 7.02 (d, *J* = 7.8 Hz, 1H), 7.15 (t, 1H), 7.25 (t, 1H), 7.56 (d, *J* = 8.3 Hz, 2H) 7.66 (d, *J* = 7.4 Hz, 1H), 7.85 (d, *J* = 8.3 Hz, 2H), ^13^C NMR (125 Hz, CDCl_3_): *δ* = 47.5, 53.1, 54.8, 71.5, 78.4, 121.1, 123.4, 124.2, 126.2, 129.4, 129.7, 132.7, 135.9, 139.9, 157.0. HRMS (ESI) *m*/*z*: calcd For [M + H] C_17_H_18_N_2_O_3_SCl mass 365.07262 found 365.07212.

#### 3.2.4 1-((4-Bromophenyl)sulfonyl)-1,2,3,5,6,11*b*-hexahydrobenzo[*f*]imidazo[1,2-*d*][1,4]oxazepine (9d)

The colorless solid was washed with about 5 mL of methanol (76% yield), mp 140–141 °C. ^1^H NMR (500 Hz, CDCl_3_): *δ* = 2.12 (dd, *J* = 8.6, 15.1 Hz, 1H), 2.76 (t, *J* = 10.8 Hz, 1H), 2.84 (m, 1H), 2.91 (d, *J* = 12.7 Hz, 1H), 3.58–3.63 (m, 1H), 3.69–3.72 (m, 1H), 3.79 (t, *J* = 10.8 Hz, 1H), 4.32 (d, *J* = 12.2 Hz, 1H), 5.22 (s, 1H), 7.02 (d, *J* = 7.8 Hz, 1H), 7.13 (t, *J* = 7.4 Hz,1H), 7.23 (t, *J* = 7.4 Hz, 1H), 7.66 (d, *J* = 7.4 Hz, 1H), 7.73 (d, *J* = 8.3 Hz, 2H), 7.78 (d, *J* = 8.3 Hz, 2H), ^13^C NMR, (125 Hz, CDCl_3_): *δ* = 47.5, 53.1, 54.8, 71.5, 78.4, 121.1, 123.4, 124.2, 126.2, 128.5, 129.4, 129.5, 132.7, 136.5, 157.0. HRMS (ESI) *m*/*z*: calcd For [M + H] C_17_H_18_N_2_O_3_SBr mass 409.02212 found 409.02160.

#### 3.2.5 1-((4-Iodophenyl)sulfonyl)-1,2,3,5,6,11*b*-hexahydrobenzo[*f*]imidazo[1,2-*d*][1,4]oxazepine (9e)

The colorless solid was washed with about 5 mL of methanol (68% yield), mp 163–164 °C. ^1^H NMR (500 Hz, CDCl_3_): *δ* = 2.24 (m, 1H), 2.78–2.85 (m, 3H), 3.70 ((m, 1H), 3.75 (m, 1H), 3.84 (t, *J* = 10.1, 1H), 4.41 (d, *J* = 11.8 Hz, 1H), 5.4 (s, 1H), 7.42–7.45 (m, 3H), 7.73–7.74 (m, 3H), 7.80 (d, *J* = 7.8 Hz, 2H), 7.86 (d, *J* = 7.2 Hz, 1H), 8.05 (s, 1H), ^13^C NMR, (125 Hz, CDCl_3_): *δ* = 47.6, 53.0, 54.1, 72.2, 78.3, 117.8, 125.2, 125.7, 126.5, 126.8, 128.4, 128.5, 129.5, 130.6, 132.7, 136.5, 154.6. HRMS (ESI) *m*/*z*: calcd For [M + H] C_17_H_18_N_2_O_3_SI mass 457.0083 found 457.00773.

#### 3.2.6 1-Tosyl-1,2,3,5,6,11*b*-hexahydrobenzo[*f*]imidazo[1,2-*d*][1,4]oxazepine (9f)

The colorless solid was washed with about 5 mL of diethyl ether (67% yield), mp 128–130 °C. ^1^H NMR (500 Hz, CDCl_3_): *δ* = 2.12 (dd, *J* = 8.9, 15.2 Hz, 1H), 2.47 (s, CH_3_-, 3H), 2.74 (t, *J* = 10.7 Hz, 1H), 2.83 (m, 1H), 2.87 (d, *J* = 12.9 Hz, 1H), 3.54 (m, 1H), 3.72–3.78 (m, 2H), 4.30 (d, *J* = 12.3 Hz, 2H), 5.18 (s, 1H), 6.88 (d, *J* = 8.4 Hz, 1H), 7.32 (d, *J* = 7.1 Hz, 1H), 7.38 (d, *J* = 7.8 Hz, 2H), 7.72 (m, 1H), 7.75 (s, 1H), 7.79 (d, *J* = 7.8 Hz, 2H), ^13^C NMR, (125 Hz, CDCl_3_): *δ* = 21.6, 47.4, 53.3, 54.9, 71.8, 78.0, 117.1, 122.9, 128.0, 129.2, 130.1, 132.0, 134.4, 135.5, 144.4, 156.4. HRMS (ESI) *m*/*z*: calcd For [M + H] C_18_H_21_N_2_O_3_S mass 345.1268 found 345.1267.

#### 3.2.7 1-((4-Methoxyphenyl)sulfonyl)-1,2,3,5,6,11*b*-hexahydrobenzo[*f*]imidazo[1,2-*d*][1,4]oxazepine (9g)

The light yellow solid was washed with about 5 mL of methanol (79% yield), mp 148–149 °C. ^1^H NMR (500 Hz, CDCl_3_): *δ* = 2.07 (dd, *J* = 8.9, 15.3 Hz, 1H), 2.71 (t, *J* = 10.8 Hz, 1H), 2.83 (m, 1H), 2.92 (d, *J* = 12.8 Hz, 1H) 3.58 (m, 1H), 3.72 (m, 1H), 3.78 (m, 1H), 4.01 (s, 3H), 4.34 (d, *J* = 12.3 Hz, 1H), 5.18 (s, 1H), 7.01–7.05 (m, 2H), 7.17 (t, *J* = 7.4 Hz, 1H), 7.23 (t, *J* = 7.4 Hz, 1H), 7.73 (d, *J* = 7.3 Hz, 1H) 7.85 (d, *J* = 8.5 Hz, 2H), ^13^C NMR (125 Hz, CDCl_3_): *δ* = 47.6, 53.5, 55.3, 55.8, 71.8, 78.7, 114.6, 121.1, 124.3, 126.5, 129.2, 129.3, 130.3, 133.5, 157.4, 163.6. HRMS (ESI) *m*/*z*: calcd For [M + H] C_18_H_21_N_2_O_4_S mass 361.12212 found 361.12165.

#### 3.2.8 1-((4-(Trifluoromethyl)phenyl)sulfonyl)-1,2,3,5,6,11*b*-hexahydrobenzo[*f*]imidazo[1,2-*d*][1,4]oxazepine (9h)

The pale-yellow solid was washed with about 5 mL of diethyl ether (77% yield), mp 128–129 °C. ^1^H NMR (500 Hz, CDCl_3_): *δ* = 2.14 (m, 1H), 2.77 (m, 1H), 2.81–2.87 (m, 2H), 3.64 (m, 1H), 3.72 (m, 1H), 3.79 (t, *J* = 10.1 Hz, 1H), 4.32 (d, *J* = 11.0 Hz, 1H), 5.28 (s, 1H), 7.02 (d, *J* = 7.5 Hz, 1H), 7.13 (t, *J* = 6.9 Hz, 1H), 7.23 (t, *J* = 6.9 Hz, 1H), 7.63 (d, *J* = 7.0 Hz, 1H), 7.85 (d, *J* = 7.5 Hz, 2H), 8.05 (d, *J* = 7.5 Hz, 2H), ^13^C NMR, (125 Hz, CDCl_3_): *δ* = 47.5, 53.0, 54.6, 71.3, 78.7, 121.1, 124.2, 126.1, 126.5, 128.5, 129.5, 132.4, 134.8, 141.0, 156.9. HRMS (ESI) *m*/*z*: calcd For [M + H] C_18_H_18_N_2_O_3_SF_3_ mass 399.0988 found 399.08847.

#### 3.2.9 1-((4-Nitrophenyl)sulfonyl)-1,2,3,5,6,11*b*-hexahydrobenzo[*f*]imidazo[1,2-*d*][1,4]oxazepine (9i)

The light-yellow solid was washed with about 5 mL of diethyl ether (77% yield), mp 147–148 °C. ^1^H NMR (500 Hz, CDCl_3_): *δ* = 2.21 (m, 1H), 2.75–2.83 (m, 3H), 3.68–3.78 (m, 3H) 4.31 (m, 1H), 5.32 (s, 1H), 7.02 (d, *J* = 7.3 Hz, 1H), 7.16 (t, *J* = 6.7 Hz, 1H), 7.24 (t, *J* = 6.7 Hz, 1H), 7.59 (d, *J* = 6.7 Hz, 1H), 8.10 (d, *J* = 7.6 Hz, 2H), 8.42 (d, *J* = 7.6 Hz, 2H), ^13^C NMR, (125 Hz, CDCl_3_): *δ* = 47.6, 52.8, 54.2, 71.3, 78.3, 121.0, 124.2, 124.5, 126.1, 129.2, 129.7, 131.8, 143.1, 150.5, 156.7. HRMS (ESI) *m*/*z*: calcd For [M + H] C_17_H_18_N_3_O_5_S mass 376.09672 found 376.09617.

#### 3.2.10 1-((3,5-Dichlorophenyl)sulfonyl)-1,2,3,5,6,11*b*-hexahydrobenzo[*f*]imidazo[1,2-*d*][1,4]oxazepine (9j)

The colorless solid was washed with about 5 mL of methanol (94% yield), mp 118–119 °C. ^1^H NMR (500 Hz, CDCl_3_): *δ* = 2.74 (m, 1H), 2.99 (m, 3H), 3.66 (m, 1H), 3.87 (m, 1H), 4.05 (m, 1H), 4.32 (m, 1H), 5.70 (s, 1H), 6.95 (d, *J* = 7.6 Hz, 1H), 6.99 (t, *J* = 7.2 Hz,1H), 7.19 (t, *J* = 7.1 Hz, 1H), 7.29 (d, *J* = 7.1 Hz, 1H), 7.45 (d, *J* = 8.1 Hz, 1H), 7.50 (d, *J* = 8.1 Hz, 1H), 7.96 (bs, 1H). ^13^C NMR, (125 Hz, CDCl_3_): *δ* = 47.8, 53.5, 54.1, 71.5, 78.6, 121.3, 123.9, 126.0, 129.7, 130.4, 132.1, 133.4, 133.8, 138.4, 157.0. HRMS (ESI) *m*/*z*: calcd For [M + H] C_17_H_17_N_2_O_3_SCl_2_, mass 399.03372 found 399.03314.

#### 3.2.11 10-Chloro-1-(phenylsulfonyl)-1,2,3,5,6,11*b*-hexahydrobenzo[*f*]imidazo[1,2-*d*][1,4]oxazepine (10a)

The colorless solid was washed with about 5 mL of methanol (78% yield), mp 150–152 °C. ^1^H NMR (500 Hz, CDCl_3_): *δ* = 2.24 (dd, *J* = 8.8, 15.4 Hz, 1H), 2.71 (t, *J* = 10.9 Hz, 1H), 2.83 (m, 2H), 2.89 (d, *J* = 12.7 Hz, 1H), 3.56 (m, 1H), 3.71–3.78 (m, 2H), 4.29 (d, *J* = 12.2 Hz, 1H), 5.18 (s, 1H), 6.94 (d, *J* = 8.3 Hz, 1H), 7.18 (d, *J* = 8.3 Hz, 1H), 7.52–7.63 (m, 3H), 7.67 (t, *J* = 8.3 Hz, 1H) 7.91 (d, *J* = 7.4 Hz, 2H). ^13^C NMR, (125 Hz, CDCl_3_): *δ* = 47.6, 53.4, 55.0, 71.9, 78.2, 122.7, 126.4, 128.1, 129.2, 129.6, 133.6, 135.2, 137.4, 155.9. HRMS (ESI) *m*/*z*: calcd For [M + H] C_17_H_18_ClN_2_O_3_S 365.0726 found 365.07212.

#### 3.2.12 10-Chloro-1-((4-fluorophenyl)sulfonyl)-1,2,3,5,6,11*b*-hexahydrobenzo[*f*]imidazo[1,2-*d*][1,4]oxazepine (10b)

The colorless solid was washed with about 5 mL of diethyl ether (84% yield), mp 128–129 °C. ^1^H NMR (500 Hz, CDCl_3_): *δ* = 2.13 (m, 1H), 2.78 (t, *J* = 10.9 Hz, 1H), 2.86–2.92 (m, 2H), 3.58 (m, 1H), 3.73–3.77 (m, 2H), 4.31 (d, *J* = 12.0 Hz, 1H), 5.18 (s, 1H), 6.95 (d, *J* = 8.2 Hz, 1H), 7.23 (d, *J* = 8.2 Hz, 1H), 7.30 (d, *J* = 7.5 Hz, 2H), 7.58 (bs, 1H), 7.93 (d, *J* = 7.9 Hz, 2H). ^13^C NMR, (125 Hz, CDCl_3_): *δ* = 47.5, 53.2, 54.7, 717, 78.0, 116.7 (d, ^3^*J*_C–F_ = 22.3 Hz), 122.6, 126.3, 129.2, 129.5, 130.7 (d, ^4^*J*_C–F_ = 9.3 Hz), 133.5, 134.7, 155.7, 165.6 (d, ^1^*J*_C–F_ = 254.8 Hz). HRMS (ESI) *m*/*z*: calcd For [M + H] C_17_H_17_ClN_2_O_3_SF 383.06322 found 383.06270.

#### 3.2.13 10-Chloro-1-((4-chlorophenyl)sulfonyl)-1,2,3,5,6,11*b*-hexahydrobenzo[*f*]imidazo[1,2-*d*][1,4]oxazepine (10c)

The pale yellow solid was washed with about 5 mL of diethyl ether (71% yield), mp 140–142 °C. ^1^H NMR (500 Hz, CDCl_3_): *δ* = 2.14 (m, 1H), 2.72–2.79 (m, 1H), 2.85–2.89 (m, 2H), 3.58 (m, 1H), 3.73–3.77 (m, 2H), 4.30 (d, *J* = 12.4 Hz, 1H), 5.18 (s, 1H), 6.95 (d, *J* = 8.7 Hz, 1H), 7.19 (d, *J* = 8.7 Hz, 1H), 7.57 (m, 3H), 7.85 (d, *J* = 8.7 Hz, 2H), ^13^C NMR (125 Hz, CDCl_3_): *δ* = 47.5, 53.2, 54.6, 71.7, 78.0, 122.6, 126.3, 129.2, 129.4, 129.5, 129.8, 134.6, 135.9, 140.2, 155.6. HRMS (ESI) *m*/*z*: calcd For [M + H] C_17_H_17_Cl_2_N_2_O_3_S 399.03362 found 399.03314.

#### 3.2.14 1-((4-Bromophenyl)sulfonyl)-10-chloro-1,2,3,5,6,11*b*-hexahydrobenzo[*f*]imidazo[1,2-*d*][1,4]oxazepine (10d)

The colorless solid was washed with about 5 mL of methanol (67% yield), mp 154–155 °C. ^1^H NMR (500 Hz, CDCl_3_): *δ* = 2.15 (m, 1H), 2.77 (t, *J* = 10.7 Hz, 1H), 2.83 (m, 1H), 2.89 (d, *J* = 12.7 Hz, 1H), 3.8 (m, 1H), 3.73–3.78 (m, 2H), 4.31 (d, *J* = 11.5 Hz, 1H), 5.19 (s, 1H), 6.95 (d, *J* = 7.8 Hz, 1H), 7.19 (d, *J* = 7.8 Hz, 1H), 7.56 (s, 1H), 7.74–7.76 (m, 4H), ^13^C NMR (125 Hz, CDCl_3_): *δ* = 47.5, 53.2, 54.6, 71.7, 78.0, 122.6, 126.3, 128.7, 129.1, 129.4, 132.7, 134.6, 136.4, 155.6. HRMS (ESI) *m*/*z*: calcd For [M + H] C_17_H_11_ClN_2_O_3_SBr 442.98322 found 442.9826.

#### 3.2.15 10-Chloro-1-((4-iodophenyl)sulfonyl)-1,2,3,5,6,11*b*-hexahydrobenzo[*f*]imidazo[1,2-*d*][1,4]oxazepine (10e)

The colorless solid was washed with about 5 mL of diethyl ether (77% yield), mp 159–161 °C. ^1^H NMR (500 Hz, CDCl_3_) *δ* = 2.13 (m, 1H), 2.76 (m, 1H), 2.84–2.94 (m, 2H), 3.56 (m, 1H), 3.72–3.77 (m, 2H), 4.30 (d, *J* = 11.0 Hz, 1H), 5.18 (s, 1H), 6.94 (d, *J* = 7.4 Hz, 1H), 7.17 (d, *J* = 7.8 Hz, 1H), 7.56 (s, 1H), 7.61 (d, *J* = 6.5 Hz, 2H) 7.94 (d, *J* = 6.5 Hz, 2H). ^13^C NMR (125 Hz, CDCl_3_): *δ* = 47.6, 53.3, 54.7, 71.8, 78.0, 101.2, 122.7, 126.4, 129.2, 129.3, 129.4, 134.5, 137.1, 138.8, 155.7. HRMS (ESI) *m*/*z*: calcd For [M^+^Na] C_17_H_16_ClN_2_O_3_SINa 512.9512 found 512.9507.

#### 3.2.16 10-Chloro-1-tosyl-1,2,3,5,6,11*b*-hexahydrobenzo[*f*]imidazo[1,2-*d*][1,4]oxazepine (10f)

The light-yellow solid was washed with about 5 mL of methanol (73% yield), mp 148–149 °C. ^1^H NMR (500 Hz, CDCl_3_): *δ* = 2.05 (dd, *J* = 9.0, 15.4 Hz, 1H), 2.46 (s, CH_3_-, 3H), 2.72 (t, *J* = 10.7 Hz, 1H), 2.82 (m, 2H), 2.90 (d, *J* = 12.8 Hz, 1H), 3.54 (m, 1H), 3.71–3.76 (m, 2H), 4.29 (d, *J* = 12.2 Hz, 1H), 5.17 (s, 1H), 6.94 (d, *J* = 8.3 Hz, 1H), 7.17 (d, *J* = 6.9 Hz, 1H), 7.37 (d, *J* = 7.5 Hz, 2H), 7.63 (s, 1H), 7.79 (d, *J* = 7.7 Hz, 2H). ^13^C NMR (125 Hz, CDCl_3_): *δ* = 21.8, 47.6, 53.4, 55.0, 71.9, 78.2, 122.6, 126.5, 128.2, 129.1, 129.6, 130.2, 134.5, 135.3, 144.6, 155.9. HRMS (ESI) *m*/*z*: calcd For [M + H] C_18_H_20_ClN_2_O_3_S 379.08822 found 379.0878.

#### 3.2.17 10-Chloro-1-((4-methoxyphenyl)sulfonyl)-1,2,3,5,6,11*b*-hexahydrobenzo[*f*]imidazo[1,2-*d*][1,4]oxazepine (10g)

The light-yellow solid was washed with about 5 mL of methanol (81% yield), mp 156–158 °C. ^1^H NMR (500 Hz, CDCl_3_): *δ* = 2.07 (dd, *J* = 9.0, 15.4 Hz, 1H), 2.74 (t, *J* = 10.8 Hz, 1H), 2.83 (m, 2H), 2.89 (d, *J* = 12.7 Hz, 1H), 3.54 (m, 1H), 3.72–3.76 (m, 2H), 3.90 (s, CH_3_O-, 3H), 4.29 (d, *J* = 12.3 Hz, 1H), 5.18 (s, 1H), 6.94 (d, *J* = 8.4 Hz, 1H), 7.04 (d, *J* = 8.5 Hz, 2H), 7.17 (d, *J* = 7.0 Hz, 1H), 7.63 (s, 1H), 7.84 (d, *J* = 8.5 Hz, 2H). ^13^C NMR (125 Hz, CDCl_3_): *δ* = 47.6, 53.5, 55.1, 55.8, 71.9, 78.2, 114.7, 122.6, 126.5, 129.1, 129.6, 130.3, 135.4, 156.0, 163.7. HRMS (ESI) *m*/*z*: calcd For [M + H] C_18_H_20_ClN_2_O_4_S 395.08322 found 395.08268.

#### 3.2.18 10-Chloro-1-((4-(trifluoromethyl)phenyl)sulfonyl)-1,2,3,5,6,11*b*-hexahydrobenzo[*f*]imidazo[1,2-*d*][1,4]oxazepine (10h)

The colorless solid was washed with about 5 mL of diethyl ether (64% yield), mp 130–131 °C. ^1^H NMR (500 Hz, CDCl_3_): *δ* = 2.24 (dd, *J* = 8.5, 15.3 Hz, 1H), 2.77 (t, *J* = 11.2 Hz, 1H), 2.86–2.90 (m, 2H), 2.89 (d, *J* = 12.7 Hz, 1H), 3.60 (m, 1H), 3.70–3.78 (m, 2H), 4.31 (d, *J* = 12.2 Hz, 1H), 5.25 (s, 1H), 6.95 (d, *J* = 8.4 Hz, 1H), 7.19 (d, *J* = 8.4 Hz, 1H), 7.48 (bs, 1H), 7.86 (d, *J* = 7.9 Hz, 2H) 8.04 (d, *J* = 7.9 Hz, 2H). ^13^C NMR (125 Hz, CDCl_3_): *δ* = 47.6, 53.2, 54.6, 71.7, 78.0, 122.8, 124.2, 126.3, 126.8, 129.0, 129.6, 134.4, 140.1, 155.7. HRMS (ESI) *m*/*z*: calcd For [M + H] C_18_H_17_ClN_2_O_3_SF3 433.0600 found 433.05950.

#### 3.2.19 10-Chloro-1-((4-nitrophenyl)sulfonyl)-1,2,3,5,6,11*b*-hexahydrobenzo[*f*]imidazo[1,2-*d*][1,4]oxazepine (10i)

The light-yellow solid was washed with about 5 mL of methanol (74% yield), mp 152–154 °C. ^1^H NMR (500 Hz, CDCl_3_): *δ* = 2.24 (m, 1H), 2.78 (t, *J* = 10.8 Hz, 1H), 2.82–2.92 (m, 2H), 3.62 (m, 1H), 3.75–3.79 (m, 2H), 4.31 (d, *J* = 11.6 Hz, 1H), 5.28 (s, 1H), 6.96 (d, *J* = 8.0 Hz, 1H), 7.20 (d, *J* = 8.0 Hz, 1H), 7.46 (s, 1H), 8.09 (d, *J* = 7.7 Hz, 2H) 8.43 (d, *J* = 7.7 Hz, 2H). ^13^C NMR (125 Hz, CDCl_3_): *δ* = 47.6, 53.0, 54.3, 71.6, 78.0, 122.8, 124.7, 126.3, 129.3, 129.5, 133.9, 143.3, 150.7, 155.5. HRMS (ESI) *m*/*z*: calcd For [M + H] C_17_H_17_ClN_3_O_5_S 410.0577 found 410.0572.

#### 3.2.20 10-Chloro-1-((3,5-*d*ichlorophenyl)sulfonyl)-1,2,3,5,6,11*b*-hexahydrobenzo[*f*]imidazo[1,2-*d*][1,4]oxazepine (10j)

The light-yellow solid was washed with about 5 mL of methanol (74% yield), mp 131–132 °C. ^1^H NMR (500 Hz, CDCl_3_): *δ* = 2.73 (m, 1H), 2.91–3.16 (m, 3H), 3.62 (m, 1H), 3.80 (t, *J* = 9.7 Hz, 1H), 4.14 (m, 1H), 4.35 (d, *J* = 11.5 Hz, 1H), 5.62 (s, 1H), 6.90 (d, *J* = 7.6 Hz, 1H), 7.11 (m, 2H), 7.48 (d, *J* = 7.6 Hz, 1H), 7.53 (d, *J* = 8.1 Hz, 1H), 7.98 (s, 1H), ^13^C NMR (125 Hz, CDCl_3_): *δ* = 48.0, 53.7, 54.2, 71.8, 78.0, 122.7, 125.6, 129.1(d, *J* = 10.0 Hz), 130.3, 131.8, 133.4 (d, *J* = 8.6 Hz), 133.9 (d, *J* = 13.3 Hz), 138.3, 155.6. HRMS (ESI) *m*/*z*: calcd For [M + H] C_17_H_16_Cl_3_N_2_O_3_S 432.9947 found 432.9942.

#### 3.2.21 10-Bromo-1-(phenylsulfonyl)-1,2,3,5,6,11*b*-hexahydrobenzo[*f*]imidazo[1,2-*d*][1,4]oxazepane (11a)

The light-yellow solid was washed with about 5 mL of methanol (84% yield), mp 140–142 °C. ^1^H NMR (500 Hz, CDCl_3_): *δ* = 2.04 (m, 1H), 2.72 (t, *J* = 10.8 Hz, 1H), 2.82 (t, *J* = 10.7 Hz, 1H), 2.89 (dt, *J* = 3.0, 12.3 Hz, 1H), 3.56 (m, 2H), 3.77–3.80 (m, 2H), 4.29 (dt, *J* = 3.0, 12.3 Hz, 1H), 5.20 (s, 1H), 6.89 (d, *J* = 8.4 Hz, 1H), 7.30 (dd, *J* = 2.4, 8.4 Hz, 1H), 7.63 (t, *J* = 7.6 Hz, 2H), 7.67 (t, *J* = 7.4 Hz,1H), 7.75 (d, *J* = 2.3 Hz, 1H) 7.91 (d, *J* = 8.4 Hz, 2H). ^13^C NMR (125 Hz, CDCl_3_): *δ* = 47.5, 53.3, 55.1, 71.6, 78.5, 120.9, 124.2, 126.3, 128.1, 129.2, 130.0, 133.3, 134.5, 138.8, 144.2, 157.2. HRMS (ESI) *m*/*z*: calcd For [M + H] C_17_H_18_BrN_2_O_3_S 409.02212 found 409.0216.

#### 3.2.22 10-Bromo-1-((4-fluorophenyl)sulfonyl)-1,2,3,5,6,11*b*-hexahydrobenzo[*f*]imidazo[1,2-*d*][1,4]oxazepane (11b)

The light-yellow solid was washed with about 5 mL of diethyl ether (84% yield), mp 125–126 °C. ^1^H NMR (500 Hz, CDCl_3_): *δ* = 2.15 (m, 1H), 2.72 (t, *J* = 10.9 Hz, 1H), 2.86 (m, 1H), 2.72 (d, *J* = 12.7 Hz, 1H), 3.57 (m, 1H), 3.70–3.72 (m, 2H), 4.31 (d, *J* = 11.9 Hz, 1H), 5.20 (s, 1H), 6.89 (d, *J* = 8.1 Hz, 1H), 7.28 (d, *J* = 8.1 Hz, 1H), 7.34 (d, *J* = 7.9 Hz, 1H), 7.69 (s, 1H), 8.93 (m, 2H). ^13^C NMR (125 Hz, CDCl_3_): *δ* = 47.6, 53.4, 54.8, 71.8, 78.0, 116.8 (d, ^3^*J*_C–F_ = 22.5 Hz), 117.2, 123.2, 129.2, 130.8 (d, ^4^*J*_C–F_ = 9.3 Hz), 132.3, 133.7, 135.1, 156.3, 165.8 (d, ^1^*J*_C–F_ = 256.2 Hz). HRMS (ESI) *m*/*z*: calcd For [M + H] C_17_H_17_BrN_2_O_3_SF 427.01272 found 427.01218.

#### 3.2.23 10-Bromo-1-((4-chlorophenyl)sulfonyl)-1,2,3,5,6,11*b*-hexahydrobenzo[*f*]imidazo[1,2-*d*][1,4]oxazepine (11c)

The light-yellow solid was washed with about 5 mL of diethyl ether (69% yield), mp 148–150 °C. ^1^H NMR (500 Hz, CDCl_3_): *δ* = 2.17 (m, 1H), 2.77 (t, *J* = 10.8 Hz, 1H), 2.86–2.89 (m, 2H), 3.57 (m, 1H), 3.74–3.78 (m, 2H), 4.31 (d, *J* = 12.0 Hz, 1H), 5.20 (s, 1H), 6.89 (d, *J* = 8.3 Hz, 1H), 7.34 (d, *J* = 7.6 Hz, 1H), 7.57 (d, *J* = 7.83 Hz, 2H), 7.68 (s, 1H), 7.81 (d, *J* = 7.83 Hz, 2H), ^13^C NMR, (125 Hz, CDCl_3_): *δ* = 47.5, 53.2, 54.6, 71.6, 77.9, 117.1, 123.0, 129.1, 129.4, 129.8, 132.2, 134.9, 136.0, 140.1, 156.2. HRMS (ESI) *m*/*z*: calcd For [M + H] C_17_H_17_BrN_2_O_3_SCl 442.9832 found 442.9826.

#### 3.2.24 10-Bromo-1-((4-bromophenyl)sulfonyl)-1,2,3,5,6,11*b*-hexahydrobenzo[*f*]imidazo[1,2-*d*][1,4]oxazepine (11d)

The light-yellow solid was washed with about 5 mL of methanol (74% yield), mp 158–160 °C. ^1^H NMR (500 Hz, CDCl_3_): *δ* = 2.17 (m, 1H), 2.77(t, *J* = 10.7, 1H), 2.85–2.91 (m, 2H), 3.57 (q, 1H), 3.73–3.78 (m, 2H), 4.31 (d, *J* = 12.1 Hz, 1H), 5.20 (s, 1H), 6.89 (d, *J* = 8.3 Hz, 1H), 7.34 (d, *J* = 7.5 Hz, 2H), 7.67 (bs, 1H), 7.73 (d, *J* = 7.6 Hz, 2H), 7.77 (d, *J* = 7.7 Hz, 2H), ^13^C NMR (125 Hz, CDCl_3_): *δ* = 47.5, 53.2, 54.6, 71.6, 77.9, 117.1, 123.0, 128.7, 129.1, 129.4, 132.2, 132.7,134.9, 136.5, 156.1. HRMS (ESI) *m*/*z*: calcd For [M + H] C_17_H_17_Br_2_N_2_O_3_S 486.9326 found 486.9321.

#### 3.2.25 10-Bromo-1-((4-iodophenyl)sulfonyl)-1,2,3,5,6,11*b*-hexahydrobenzo[*f*]imidazo[1,2-*d*][1,4]oxazepine (11e)

The light-yellow solid was washed with about 5 mL of diethyl ether (78% yield), mp 156–157 °C. ^1^H NMR (500 Hz, CDCl_3_): *δ* = 2.16 (m, 1H), 2.78 (t, *J* = 10.8, 1H), 2.85–2.91 (m, 2H), 3.56 (m, 1H), 3.73–3.78 (m, 2H), 4.31 (d, *J* = 11.9 Hz, 1H), 5.20 (s, 1H), 6.89 (d, *J* = 8.1 Hz, 1H), 7.34 (d, *J* = 7.7 Hz, 1H),7.61 (d, *J* = 7.5 Hz, 2H), 7.67 (bs, 1H), 7.95 (d, *J* = 7.5 Hz, 2H), ^13^C NMR, (125 Hz, CDCl_3_): *δ* = 47.5, 53.2, 54.6, 71.6, 77.9, 101.1, 117.1, 123.0, 129.1, 129.3, 132.2, 134.9, 137.1, 138.7, 156.2. HRMS (ESI) *m*/*z*: calcd For [M + H] C_17_H_17_BrN_2_O_3_SI 534.9187 found 534.9183.

#### 3.2.26 10-Bromo-1-tosyl-1,2,3,5,6,11*b*-hexahydrobenzo[*f*]imidazo[1,2-*d*][1,4]oxazepine (11f)

The colorless solid was washed with about 5 mL of methanol (77% yield), mp 153–154 °C. ^1^H NMR (500 Hz, CDCl_3_): *δ* = 2.16 (m, 1H), 2.42 (s, CH_3_– 3H), 2.71 (t, *J* = 10.8, 1H), 2.81 (m, 1H), 2.90 (m, 1H), 3.58 (m, 1H), 3.73–3.79 (m, 2H), 4.30 (d, *J* = 11.9 Hz, 1H), 5.21 (s, 1H), 7.01 (d, *J* = 7.3 Hz, 1H), 7.16 (d, *J* = 7.0 Hz, 1H), 7.23 (s, 1H), 7.37 (d, *J* = 7.3 Hz, 2H), 7.72 (d, *J* = 6.8 Hz, 1H), 7.80 (d, *J* = 7.3 Hz, 2H). ^13^C NMR (125 Hz, CDCl_3_): *δ* = 21.7, 47.6, 53.4, 55.2, 71.7, 78.6, 121.1, 124.3, 126.4, 128.2, 129.3, 130.1, 133.4, 134.6, 144.4, 157.3. HRMS (ESI) *m*/*z*: calcd For [M + H] C_18_H_20_BrN_2_O_3_S 423.0377 found 423.0373.

#### 3.2.27 10-Bromo-1-((4-methoxyphenyl)sulfonyl)-1,2,3,5,6,11*b*-hexahydrobenzo[*f*]imidazo[1,2-*d*][1,4]oxazepine (11g)

The light-yellow solid was washed with about 5 mL of diethyl ether (81% yield), mp 140–142 °C. ^1^H NMR (500 Hz, CDCl_3_): *δ* = 2.09 (dd, *J* = 9.0, 15.2 Hz, 1H), 2.74 (t, *J* = 10.8, 1H), 2.84 (m, 1H), 2.91 (d, *J* = 12.9 Hz, 1H), 3.54 (m, 1H), 3.72–3.76 (m, 2H), 3.89 (s, 3H, CH_3_O-), 4.30 (d, *J* = 12.3 Hz, 1H), 5.16 (s, 1H), 6.88 (d, *J* = 8.4 Hz, 1H), 7.04 (d, *J* = 8.5 Hz, 2H), 7.32 (d, *J* = 7.9 Hz, 1H), 7.75 (bs, 1H), 7.83 (d, *J* = 8.5 Hz, 2H), ^13^C NMR (125 Hz, CDCl_3_): *δ* = 47.6, 53.4, 55.1, 55.8, 71.9, 78.1, 114.7, 117.2, 123.1, 129.1, 129.3, 130.2, 132.2, 135.7, 156.5, 163.7. HRMS (ESI) *m*/*z*: calcd For [M + H] C_18_H_20_BrN_2_O_4_S 439.0327 found 438.2104.

#### 3.2.28 10-Bromo-1-((4-(trifluoromethyl)phenyl)sulfonyl)-1,2,3,5,6,11*b*-hexahydrobenzo[*f*]imidazo[1,2-*d*][1,4]oxazepine (11h)

The light-yellow solid was washed with about 5 mL of diethyl ether (83% yield), mp 128–129 °C. ^1^H NMR (500 Hz, CDCl_3_): *δ* = 2.18 (m, 1H), C 2.87–2.90 (m, 2H), 3.60 (m, 1H), 3.76–3.78 (m, 2H), 4.31 (d, *J* = 11.5 Hz, 1H), 5.26 (s, 1H), 6.9 (d, *J* = 7.9 Hz, 1H), 7.34 (d, *J* = 7.7 Hz, 2H), 7.63 (bs, 1H), 7.86 (d, *J* = 7.1 Hz, 2H), 8.04 (d, *J* = 7.1 Hz, 2H), ^13^C NMR (125 Hz, CDCl_3_): *δ* = 47.6, 53.3, 54.5, 71.7, 77.9, 117.2, 123.2, 126.7, 126.7, 128.6, 129.2, 132.5, 134.7, 141.2, 156.2. HRMS (ESI) *m*/*z*: calcd For [M + H] C_18_H_17_BrN_2_O_3_SF3 477.00952 found 477.0899.

#### 3.2.29 10-Bromo-1-((4-nitrophenyl)sulfonyl)-1,2,3,5,6,11*b*-hexahydrobenzo[*f*]imidazo[1,2-*d*][1,4]oxazepine (11i)

The colorless solid was washed with about 5 mL of methanol (71% yield), mp 154–155 °C. ^1^H NMR (500 Hz, CDCl_3_): *δ* = 2.28 (m, 1H), 2.81 (m, 1H), 2.83–2.89 (m, 2H), 3.62 (m, 1H), 3.75–3.79 (m, 2H), 4.32 (d, *J* = 10.8 Hz, 1H), 5.30 (s, 1H), 6.90 (d, *J* = 7.5 Hz, 1H), 7.35 (d, *J* = 6.8 Hz, 1H), 7.56 (s, 1H), 8.09 (d, *J* = 7.0 Hz, 2H), 8.43 (d, *J* = 7.0 Hz, 2H), ^13^C NMR (125 Hz, CDCl_3_): *δ* = 47.6, 53.1, 54.2, 71.6, 78.1, 117.2, 123.3, 124.8, 129.1, 129.1, 132.6, 134.2, 143.4, 150.7, 156.1. HRMS (ESI) *m*/*z*: calcd For [M + H] C_17_H_17_BrN_3_O_5_S 454.0072 found 454.0067.

#### 3.2.30 10-Bromo-1-((3,5-dichlorophenyl)sulfonyl)-1,2,3,5,6,11*b*-hexahydrobenzo[*f*]imidazo[1,2-*d*][1,4]oxazepine (11j)

The pale-yellow solid was washed with about 5 mL of diethyl ether (76% yield), mp 130–131 °C. ^1^H NMR (500 Hz, CDCl_3_): *δ* = 2.74 (m, 1H), 3.01–3.05 (m, 3H), 3.62 (m, 1H), 3.79 (m, 1H), 4.17 (m, 1H), 4.35 (d, *J* = 11.8 Hz, 1H), 5.62 (s, 1H), 6.85 (d, *J* = 8.3 Hz, 1H), 7.20–7.24 (m, 2H), 7.48 (d, *J* = 8.3 Hz, 1H), 7.54 (d, *J* = 8.3 Hz, 1H), 7.96 (s, 1H). ^13^C NMR (125 Hz, CDCl_3_): *δ* = 48.2, 53.9, 54.3, 71.9, 78.1, 116.8, 123.3, 128.7, 130.4, 131.8, 132.3, 133.5, 134.2, 138.5, 156.3. HRMS (ESI) *m*/*z*: calcd for [M + H] C_17_H_16_BrN_2_O_3_SCl_2_ 476.9441 found 476.9437.

#### 3.2.31 10-Nitro-1-(phenylsulfonyl)-1,2,3,5,6,11*b*-hexahydrobenzo[*f*]imidazo[1,2-*d*][1,4]oxazepine (12a)

The yellow solid was washed with about 5 mL of methanol (87% yield), mp 150–152 °C. ^1^H NMR (500 Hz, CDCl_3_): *δ* = 2.24 (dd, *J* = 8.6, 15.3 Hz, 1H), 2.80 (t, *J* = 11.2, 1H), 2.87 (m, 1H), 2.97 (d, *J* = 13.0, 1H), 3.56 (m, 1H), 3.79 (m, 1H), 3.84 (t, *J* = 11.2, 1H) 4.41 (d, *J* = 12.3, 1H), 5.26 (s, 1H), 7.11 (d, *J* = 8.7 Hz, 1H), 7.56–62 (m, 2H), 7.68 (t, *J* = 7.1 Hz, 1H), 7.93 (d, *J* = 7.4 Hz, 2H) 8.12 (d, *J* = 8.5 Hz, 1H), 8.51 (s, 1H). ^13^C NMR (125 Hz, CDCl_3_): *δ* = 47.6, 53.4, 54.5, 72.3, 77.8, 122.3, 122.8, 125.1, 128.1, 129.7, 133.8, 134.5, 137.4, 144.1, 162.6. HRMS (ESI) *m*/*z*: calcd for [M + H] C_17_H_18_N_3_O_5_S 376.09672 found 365.07212.

#### 3.2.32 1-((4-Fluorophenyl)sulfonyl)-10-nitro-1,2,3,5,6,11*b*-hexahydrobenzo[*f*]imidazo[1,2-*d*][1,4]oxazepine (12b)

The light-yellow solid was washed with about 5 mL of methanol (80% yield), mp 142–143 °C. ^1^H NMR (500 Hz, CDCl_3_): *δ* = 2.29 (m, 1H), 2.84–2.91 (m, 2H), 2.99 (d, *J* = 11.4, 1H), 3.58 (m, 1H), 3.77 (m, 1H), 3.88 (t, *J* = 10.1 Hz, 1H), 4.44 (d, *J* = 10.6 Hz, 1H), 5.27 (s, 1H), 7.10 (d, *J* = 8.3 Hz, 1H), 7.27 (d, *J* = 6.5 Hz, 2H), 7.96 (d, *J* = 6.5 Hz, 2H), 8.14 (d, *J* = 8.3 Hz, 1H), 8.49 (bs, 1H), ^13^C NMR (125 Hz, CDCl_3_): *δ* = 47.6, 53.4, 54.3, 72.2, 77.8, 117.0 (d, ^3^*J*_C–F_ = 22.6 Hz), 122.3, 122.8, 125.2, 130.9 (d, ^4^*J*_C–F_ = 9.4 Hz), 133.6, 134.1, 143.1, 162.5, 165.7 (d, ^1^*J*_C–F_ = 254.9 Hz). HRMS (ESI) *m*/*z*: calcd for [M + H] C_17_H_17_N_3_O_5_SF 394.0873 found 394.0868.

#### 3.2.33 1-((4-Chlorophenyl)sulfonyl)-10-nitro-1,2,3,5,6,11*b*-hexahydrobenzo[*f*]imidazo[1,2-*d*][1,4]oxazepine (12c)

The yellow solid was washed with about 5 mL of methanol (84% yield), mp 150–152 °C. ^1^H NMR (500 Hz, CDCl_3_): *δ* = 2.30 (dd, *J* = 8.9, 15.2 Hz, 1H), 2.83–2.92 (m, 2H), 2.96 (d, *J* = 13.3 Hz, 1H), 3.58 (td, *J* = 5.3, 10.3 Hz, 1H), 3.76 (m, 1H), 3.87 (t, *J* = 10.3 Hz, 1H), 4.44 (d, *J* = 12.4 Hz, 1H), 5.27 (s, 1H), 7.13 (d, *J* = 8.7 Hz, 1H), 7.56 (d, *J* = 8.3 Hz, 2H), 7.87 (d, *J* = 8.3 Hz, 2H), 8.14 (dd, *J* = 2.3, 8.7 Hz, 2H), 8.45 (d, *J* = 2.3 Hz, 1H), ^13^C NMR (125 Hz, CDCl_3_): *δ* = 476, 53.4, 54.3, 72.2, 77.8, 122.3, 122.8, 125.3, 129.5, 130.0, 134.0, 136.0, 140.6, 144.1, 162.4. HRMS (ESI) *m*/*z*: calcd For [M + H] C_17_H_17_N_3_O_5_SCl 410.0577 found 410.0572.

#### 3.2.34 1-((4-Bromophenyl)sulfonyl)-10-nitro-1,2,3,5,6,11*b*-hexahydrobenzo[*f*]imidazo[1,2-*d*][1,4]oxazepine (12d)

The yellow solid was washed with about 5 mL of methanol (82% yield), mp 159–161 °C. ^1^H NMR (500 Hz, CDCl_3_): *δ* = 2.29 (dd, *J* = 8.9, 15.2 Hz, 1H), 2.83–2.91 (m, 2H), 2.98 (d, *J* = 13.4 Hz, 1H), 3.58 (td, *J* = 5.3, 10.3 Hz, 1H), 3.75 (m, 1H), 3.87 (t, *J* = 10.3 Hz, 1H), 4.44 (d, *J* = 12.4 Hz, 1H), 5.27 (s, 1H), 7.12 (d, *J* = 7.1 Hz, 1H), 7.75 (d, *J* = 8.4 Hz, 2H), 7.79 (d, *J* = 8.4 Hz, 2H), 8.14 (dd, *J* = 2.3, 8.7 Hz, 2H), 8.47 (d, *J* = 2.3, 1H), ^13^C NMR (125 Hz, CDCl_3_): *δ* = 47.6, 53.3, 54.3, 72.2, 77.8, 122.3, 122.7, 125.3, 129.0, 129.6, 133.0, 134.0, 136.4, 144.1, 162.4. HRMS (ESI) *m*/*z*: calcd For [M + H] C_17_H_17_N_3_O_5_SBr 454.00722 found 454.0767.

#### 3.2.35 1-((4-Iodophenyl)sulfonyl)-10-nitro-1,2,3,5,6,11*b*-hexahydrobenzo[*f*]imidazo[1,2-*d*][1,4]oxazepine (12e)

The dark yellow solid was washed with about 5 mL of diethyl ether (88% yield), mp 166–167 °C. ^1^H NMR (500 Hz, CDCl_3_): *δ* = 2.30 (dd, *J* = 8.8, 15.2 Hz, 1H), 2.84–2.90 (m, 2H), 2.97 (m, 2H), 3.57 (td, *J* = 5.3, 10.5 Hz, 1H), 3.74 (m, 1H), 3.87 (t, *J* = 10.5 Hz, 1H), 4.44 (d, *J* = 12.4 Hz, 1H), 5.27 (s, 1H), 7.12 (d, *J* = 8.7 Hz, 1H), 7.63 (d, *J* = 8.1 Hz, 2H), 7.97 (d, *J* = 8.1 Hz, 2H), 8.14 (dd, *J* = 2.0, 8.7 Hz, 1H), 8.47 (d, *J* = 2.0, 1H), ^13^C NMR (125 Hz, CDCl_3_): *δ* = 47.6, 53.3, 54.3, 72.2, 77.6, 101.5, 122.3, 122.8, 125.3, 129.4, 134.0, 137.1, 139.0, 144.1, 162.4. HRMS (ESI) *m*/*z*: calcd For [M + H] C_17_H_17_N_3_O_5_SI 501.9933 found 501.9928.

#### 3.2.36 10-Nitro-1-tosyl-1,2,3,5,6,11*b*-hexahydrobenzo[*f*]imidazo[1,2-*d*][1,4]oxazepine (12f)

The light-yellow solid was washed with about 5 mL of methanol (88% yield), mp 146–148 °C. ^1^H NMR (500 Hz, CDCl_3_): *δ* = 2.30 (dd, *J* = 8.8, 15.1 Hz, 1H), 2.46 (s, CH_3_-, 3H), 2.77 (t, *J* = 9.0 Hz, 1H), 2.75–2.84 (m, 2H), 3.66 (m, 2H) 3.79 (m, 1H), 3.82 (m, 1H), 4.40 (dt, *J* = 3.1, 12.2, 1H), 5.369 (s, 1H), 7.38 (d, *J* = 8.2 Hz, 2H), 7.42 (t, *J* = 7.2 Hz, 1H), 7.73 (d, *J* = 7.8 Hz, 1H), 7.83 (d, *J* = 8.2 Hz, 2H), 7.87 (d, *J* = 7.8 Hz, 1H), 8.12 (s, 1H), ^13^C NMR (125 Hz, CDCl_3_): *δ* = 21.8, 47.7, 53.4, 54.7, 72.6, 78.6, 117.8, 125.2, 125.8, 126.5, 126.9, 128.2, 128.5, 130.1, 130.8, 134.2, 134.7, 144.4, 155.0. HRMS (ESI) *m*/*z*: calcd For [M + Na] C_18_H_19_N_3_O_5_S 412.0942 found 412.0942.

#### 3.2.37 1-((4-Methoxyphenyl)sulfonyl)-10-nitro-1,2,3,5,6,11*b*-hexahydrobenzo[*f*]imidazo[1,2-*d*][1,4]oxazepine (12g)

The yellow solid was washed with about 5 mL of methanol (80% yield), mp 155–157 °C. ^1^H NMR (500 Hz, CDCl_3_): *δ* = 2.24 (dd, *J* = 9.0, 15.3 Hz, 1H), 2.84 (t, *J* = 9.0 Hz, 1H), 2.89 (m, 1H), 2.99 (d, *J* = 13.1 Hz, 1H), 3.53 (td, *J* = 5.2, 10.7 Hz, 1H), 3.77 (m, 1H), 3.85 (t, *J* = 10.7 Hz, 1H), 3.90 (s, CH_3_O–, 3H), 4.42 (d, *J* = 12.4 Hz, 2H), 5.21 (s, 1H), 7.05 (d, *J* = 8.5 Hz, 2H), 7.11 (d, *J* = 8.7 Hz, 1H), 7.86 (d, *J* = 8.5 Hz, 2H), 8.12 (dd, *J* = 8.7 Hz, 1H), 8.53 (bs, 1H), ^13^C NMR (125 Hz, CDCl_3_): *δ* = 47.6, 53.6, 54.6, 55.9, 72.1, 77.8, 114.8, 122.2, 122.9, 125.1, 129.0, 130.3, 134.7, 144.1, 162.7, 163.8. HRMS (ESI) *m*/*z*: calcd For [M + H] C_18_H_20_N_3_O_6_S 406.10728 found 406.1067.

#### 3.2.38 10-Nitro-1-((4-(trifluoromethyl)phenyl)sulfonyl)-1,2,3,5,6,11*b*-hexahydrobenzo[*f*]imidazo[1,2-*d*][1,4]oxazepine (12h)

The yellow solid was washed with about 5 mL of methanol (77% yield), mp 154–156 °C. ^1^H NMR (500 Hz, CDCl_3_): *δ* = 2.33 (dd, *J* = 8.7, 15.1 Hz, 1H), 2.87–2.91 (m, 2H), 2.98 (d, *J* = 13.2 Hz, 1H), 3.61 (m, 1H), 3.78 (m, 1H), 3.89 (t, *J* = 10.5 Hz, 1H), 4.45 (d, *J* = 12.4 Hz, 1H), 5.33 (s, 1H), 7.13 (d, *J* = 8.7 Hz, 1H), 7.88 (d, *J* = 8.0 Hz, 2H), 8.07 (d, *J* = 8.0 Hz, 2H), 8.15 (dd, *J* = 2.3, 8.7 Hz, 1H), 8.41 (d, *J* = 2.3, 1H), ^13^C NMR (125 Hz, CDCl_3_): *δ* = 47.5, 53.1, 54.0, 72.0, 77.6, 122.3, 122.5, 125.2, 126.7, 126.7, 128.5, 133.5, 140.9, 144.0, 162.2. HRMS (ESI) *m*/*z*: calcd For [M + H] C_18_H_17_N_3_O_5_SF_3_ 444.0841 found 444.0836.

#### 3.2.39 10-Nitro-1-((4-nitrophenyl)sulfonyl)-1,2,3,5,6,11*b*-hexahydrobenzo[*f*]imidazo[1,2-*d*][1,4]oxazepinem (12i)

The yellow solid was washed with about 5 mL of methanol (83% yield), mp 150–152 °C. ^1^H NMR (500 Hz, CDCl_3_): *δ* = 2.38 (dd, *J* = 8.3, 15.1 Hz, 1H), 2.85–2.97 (m, 3H), 3.64 (m, 1H), 3.78 (m, 1H), 3.90 (t, *J* = 10.5 Hz, 1H)), 4.45 (d, *J* = 12.4 Hz, 1H), 5.36 (s, 1H), 7.14 (d, *J* = 8.6 Hz, 1H), 8.11–8.16 (m, 3H), 8.39 (bs, 1H), 8.46 (d, *J* = 8.2, 2H), ^13^C NMR (125 Hz, CDCl_3_): *δ* = 47.7, 53.1, 53.8, 72.1, 77.7, 122.5, 122.6, 124.9, 125.5, 129.4, 133.2, 143.2, 144.1,150.8, 162.2. HRMS (ESI) *m*/*z*: calcd For [M + H] C_17_H_17_N_4_O_7_S 421.0818 found 421.0813.

#### 3.2.40 1-((3,5-Dichlorophenyl)sulfonyl)-10-nitro-1,2,3,5,6,11*b*-hexahydrobenzo[*f*]imidazo[1,2-*d*][1,4]oxazepine (12j)

The yellow solid was washed with about 5 mL of methanol (81% yield), mp 140–141 °C. ^1^H NMR (500 Hz, CDCl_3_): *δ* = 2.83 (dd, *J* = 8.2, 15.0 Hz, 1H), 3.05–3.07 (m, 2H), 3.14 (t, *J* = 9.7 Hz, 1H), 3.64 (m, 1H), 3.92 (t, *J* = 10.6 Hz, 1H), 4.20 (m, 1H), 4.49 (d, *J* = 12.5 Hz, 1H), 5.72 (s, 1H), 7.08 (d, *J* = 8.6 Hz, 1H), 7.47 (d, *J* = 8.0 Hz, 1H), 7.56 (d, *J* = 8.4 Hz, 1H), 7.94 (s, 1H), 8.06 (s, 1H), 8.06 (d, *J* = 8.5, Hz, 1H), ^13^C NMR (125 Hz, CDCl_3_): *δ* = 48.3, 53.7, 53.8, 72.2, 77.7, 121.8, 122.4, 125.2, 130.4, 131.4, 133.2, 133.5, 133.7, 134.1, 138.1, 143.6, 162.3. HRMS (ESI) *m*/*z*: calcd For [M + H] C_17_H_16_N_3_O_5_SCl_2_ 444.0188 found 444.0182.

### 3.3 Cholinesterase inhibitory activity assay

The inhibitory activity of the synthesized compounds against AChE and BChE was evaluated *in vitro* using a slightly modified Ellman's spectrophotometric method. All assays were performed in triplicate on three independent occasions (*n* = 3). AChE (Type VI-S, from Electrophorus electricus), BChE (from equine serum), acetylthiocholine iodide (ATCI), butyrylthiocholine chloride (BTCI), 5,5′-dithiobis(2-nitrobenzoic acid) (DTNB), and tacrine hydrochloride were procured from Sigma-Aldrich. All other chemicals and solvents were of analytical grade. The assay was conducted using two sodium phosphate buffers (0.1 M): Buffer A (pH 8.0), prepared from 39% 0.1 M NaH_2_PO_4_ and 61% 0.1 M Na_2_HPO_4_, and Buffer B (pH 7.0), prepared from 5.3% 0.1 M NaH_2_PO_4_ and 94.7% 0.1 M Na_2_HPO_4_. The DTNB solution (3.96 mM) was prepared in Buffer B containing 44.6 mM sodium bicarbonate. Stock solutions of test compounds and the reference inhibitor, tacrine, were prepared in dimethyl sulfoxide (DMSO), with the final DMSO concentration in all assays maintained at 1% (v/v), a concentration confirmed not to interfere with enzyme activity. For the AChE inhibition assay, reactions were performed in a final volume of 200 µL in 96-well plates. A mixture of 120 µL of Buffer A (pH 8.0), 20 µL of test compound (final concentration 100 µM), and 20 µL of AChE solution (0.28 U mL^−1^ final) was incubated for 15 minutes at 37 °C. After incubation, 20 µL of DTNB solution was added, followed by 20 µL of acetylthiocholine iodide (13.8 mM in H_2_O) to initiate the reaction. The hydrolysis of the substrate was monitored by measuring the formation of the 5-thio-2-nitrobenzoate anion at 412 nm for 10 minutes using a Multiskan SkyHigh microplate spectrophotometer. The BChE inhibition assay followed an identical protocol, substituting BChE (0.1 U mL^−1^ final) as the enzyme and butyryl thiocholine chloride (2.21 mM in H_2_O) as the substrate. In all experiments, appropriate blanks and initial activity controls (enzyme with DMSO, but no inhibitor) were included. Tacrine was used as a positive control at a final concentration of 1 µM. The biochemical assays therefore used AChE from Electrophorus electricus and BChE from equine serum. The percentage inhibition of each enzyme was calculated using the formula: Inhibition (%) = 100 × [(*A*_0_ – *A*_1_)/*A*_0_], where *A*_0_ is the absorbance of the initial activity control, and *A*_1_ is the absorbance in the presence of the test compound or tacrine.

### 3.4 Molecular modelling

#### 3.4.1 Softwares

(1) Biovia Discovery Studio 2022.

(2) ChemDraw Ultra 7.0 version.

(3) Marvin Sketch 21.18 version.

(4) Free trial version of Mestrenova 14.2.3.

(5) Free trial version of GraphPad Prism 9.3.1.

(6) ACD/ChemSketch freeware version 2021.2.0.

(7) Free trial version of SpectraGryph 1.2.

(8) GraphPad Prism 9.3.1 software.

#### 3.4.2 Computational docking

A series of benzoxazepine–benzenesulfonamide conjugates (Appendix V) were synthesized and selected for computational docking and filtering. Synthesis and biological evaluation were performed on the 40 synthetic compounds. Molecular docking studies were performed using the docking module in BIOVIA Discovery Studio 2022 (BIOVIA, San Diego, CA). X-ray crystal structures of human AChE and BChE with bound small-molecule ligands were retrieved from the Protein Data Bank (PDB IDs: 4EY7, resolution 2.35 Å, and 7QHD, resolution 2.04 Å, respectively). The 3D coordinates of AChE were obtained as described by Berman *et al.*^40^ Hydrogen atoms were added to the protein structures using DS 4.5 templates for protein residues. Gasteiger–Marsili charges were assigned to the protein atoms *via* the DS 4.5 module.^41^ The protein structures were used for docking without energy minimization. Explicit water molecules were retained as per the docking conditions, enabling docking simulations in their presence.

Docking settings accounted for ligand flexibility and modeled the receptor as a sphere using the LibDock score and the CDocker algorithm^42^ (CATALYST_4.11 software users, 2005). The binding site for docking was defined using the co-crystallized ligands E20604 (in AChE, PDB ID: 4EY7) and C0I606 (in BChE, PDB ID: 7QHD). The selected hits were docked into the binding site with explicit water molecules using LibDock and CDocker configurations.^43–48^

The LibDock score was used to assess compound fit in the binding pocket using a rigid docking approach, with higher scores indicating better alignment and potential binding affinity. Additionally, CDocker Interaction Energy was used to estimate binding affinity; more negative values indicate stronger binding interactions.

#### 3.4.3 ADMET properties calculation

Absorption, distribution, metabolism, excretion, and toxicity were predicted using BIOVIA Discovery Studio 2022 (Biovia Inc., San Diego, California). The 2D structures of the ligands were drawn using ChemDraw Ultra 7.0 (Cambridge Soft Corp., https://www.cambridgesoft.com, USA).

#### 3.4.4 X-ray diffraction crystallography

The X-ray diffraction data for the compound were collected on an Oxford Xcalibur, Eos four-circle diffractometer. Data collection, cell refinement, and data reduction were performed using the CrysAlisPro software package.^49^ The crystal structure was solved with the SHELXT 2014/5 solution program using dual space methods and refined with the SHELXL 2019/3 refinement package^50^ using full-matrix least-squares minimization on *F*^2^. All aspects of the solution and refinement process were managed within the Olex2 1.5 interface.^51,52^ All non-hydrogen atoms were refined anisotropically. Almost all hydrogen atoms were placed at calculated positions and refined using a riding model. The crystallographic data have been deposited with the Cambridge Crystallographic Data Center (CCDC); the deposition number is [2541520], and the data can be obtained free of charge from the CCDC *via*http://www.ccdc.cam.ac.uk/data_request/cif. The detailed crystallographic data, bond lengths, and bond angles are summarized in the SI (Tables S1 and S2). Finally, all figures were prepared by Mercury CCDC.^53^

## 4. Conclusion

In this study, a series of hybrid 1,4-benzoxazepine-based sulfonamide derivatives 9–12a–j was evaluated using *in vitro* AChE/BChE inhibition assays, molecular docking, and *in silico* ADMET prediction. Several compounds showed appreciable inhibition of both enzymes, with a general preference for BChE. Compound 9d exhibited the highest BChE selectivity (IC_50_ = 4.18 ± 0.56 µM, SI = 14.94), whereas 12j showed the greatest BChE potency (IC_50_ = 1.06 ± 0.11 µM, SI = 10.37). Docking studies against AChE (4EY7) and BChE (7QHD) supported selected SAR observations, including π–π, hydrogen-bonding, and hydrophobic interactions. The SAR indicated that substituent effects were scaffold- and position-dependent, with selected brominated and nitro-substituted derivatives showing enhanced BChE preference or potency. ADMET predictions identified 9a, 9b, and 9d as having comparatively more favorable overall profiles, whereas 12j showed potential toxicity liabilities despite its higher potency. Overall, the 1,4-benzoxazepine–sulfonamide scaffold is a promising starting point for developing BChE-preferring cholinesterase inhibitors, but further structural, pharmacokinetic, toxicity, cellular, and *in vivo* studies are needed to optimize leads.

## Author contributions

Almeqdad Y. Habashneh: funding, characterized compounds, wrote the main manuscript text and revised the main manuscript text. Muhammad Ashram synthesized target compounds, wrote part of the main manuscript text and revised the main manuscript text. Wafa Hourani participated in *vitro* cholinesterase inhibition assays. Mahmoud A. Al-Sha'er conducted *in silico* studies and wrote part of the main manuscript text and revised the main manuscript text. Murad A. AlDamen conducted X-ray analysis. All authors reviewed the manuscript.

## Conflicts of interest

The authors declare no competing interests or any conflicts of interest related to this work.

## Supplementary Material

RA-OLF-D6RA08312A-s001

## Data Availability

CCDC 2541520 contains the supplementary crystallographic data for this paper.^54^ The ^1^H, ^13^C NMR, HRMS, and X-ray for all the synthesized compounds are in the supplementary information (SI) of this article. Supplementary information is available. See DOI: https://doi.org/10.1039/d6ra08312a.
