# Supplementary material for: Design, synthesis, and docking analysis of benzene sulfonamide-[1,4]benzoxazepine hybrids as acetylcholinesterase and butyrylcholinesterase inhibitors
Source: RSC Adv. 2026 Nov 4. Online ahead of print. doi: 10.1039/d6ra08312a (PMC13732728; doi:10.1039/d6ra08312a)
Supplement: Supplementary file 1 [file RA-OLF-D6RA08312A-s001.pdf]

Supplementary Information (SI) for RSC Advances.
This journal is © The Royal Society of Chemistry

# Design, Synthesis, and Docking Analysis of Benzene Sulfonamide- [1,4]Benzoxazepine Hybrids as Acetylcholinesterase and Butyrylcholinesterase Inhibitors

Almeqdad Y. Habashneh[a*], Muhammad Ashram[b] Wafa Hourani[c], Mahmoud A. Al-Sha'er[d], Murad A. AlDamen[a]

[a] Department of Chemistry, The University of Jordan, Amman 11942, Jordan;

[b] Department of Chemistry, Mutah University, P.O. Box 7, 61710 Al-Karak, Jordan;

[c] Department of Pharmaceutical Sciences, Faculty of Pharmacy, Philadelphia University, Amman 19392, Jordan;

[d] Pharmaceutical Pharmacy, Zarqa University, 2000 Zarqa, P.O. Box 13111, Jordan;

* Corresponding author, e-mail: a.habashneh@ju.edu.jo

$^{1}H$, $^{13}C$ NMR and HRMS spectrum for compounds **(9-12a-j) FigS1-S118**

Crystal data and structure refinement for **(11f)**

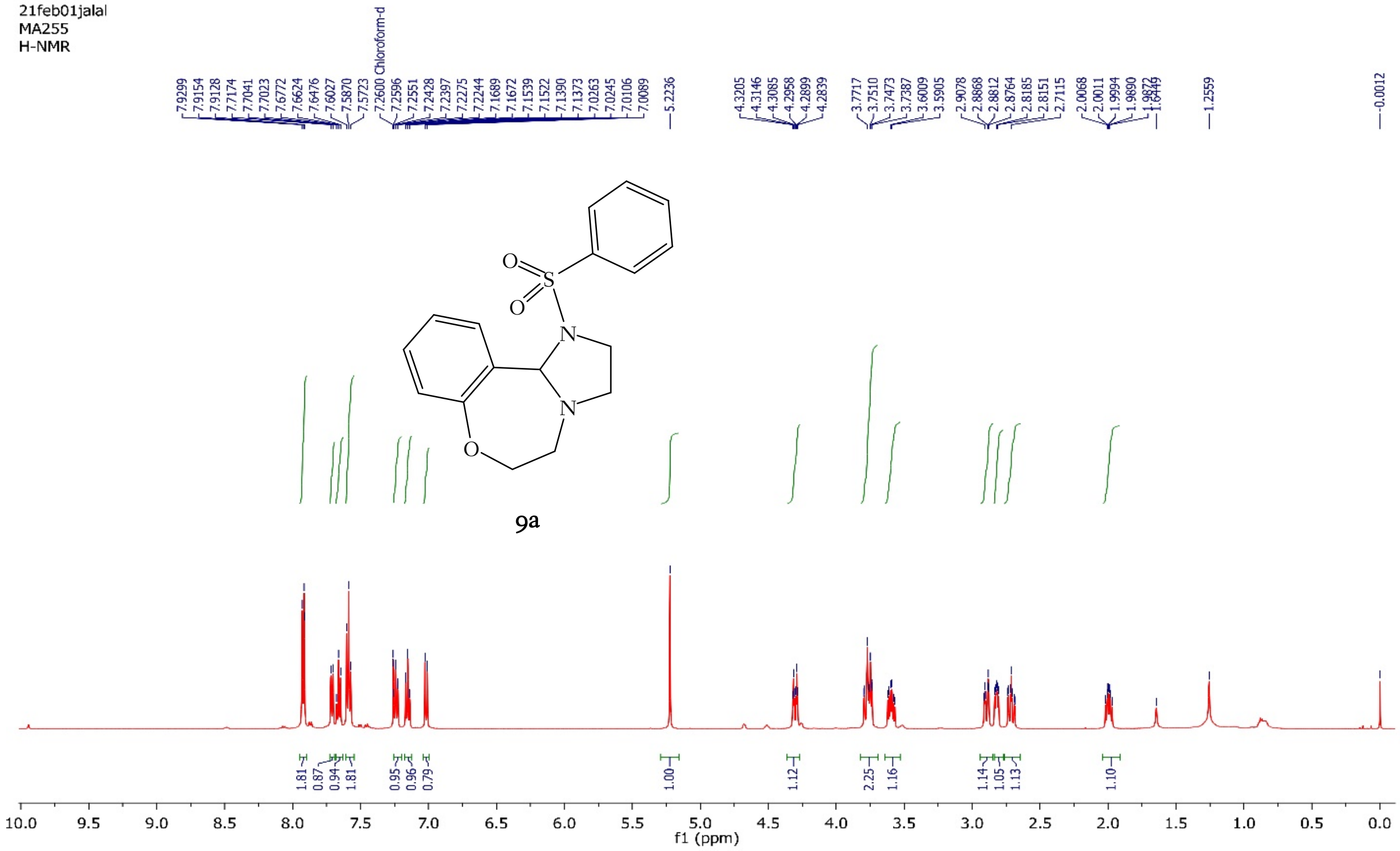


Fig.S1. $^{1}$H NMR spectrum of compound 9a $CDCl_3$

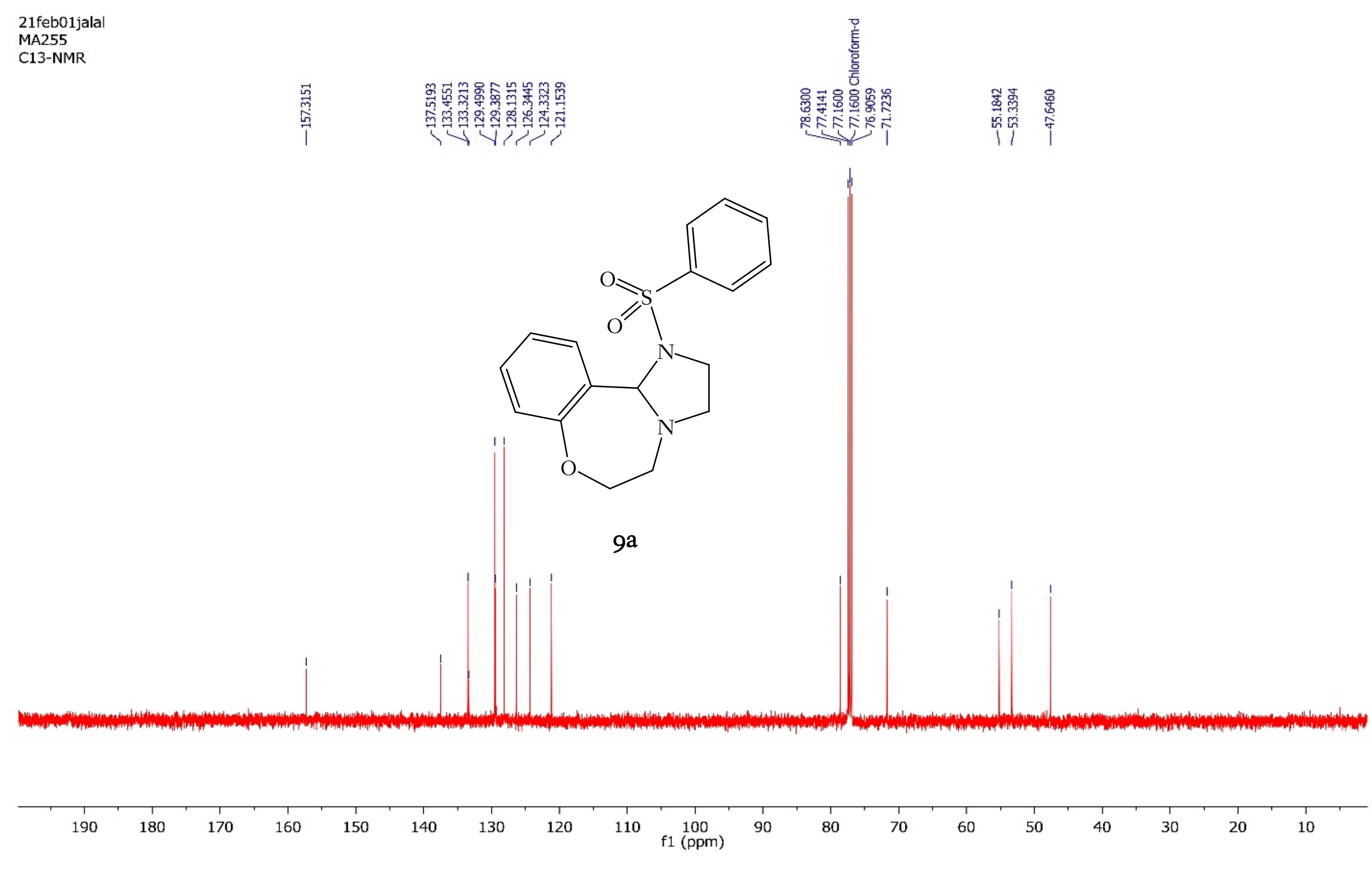

Fig.S2. $^{13}$C NMR spectrum of compound 9a $CDCl_3$

# The University of Jordan
## Faculty of science
## Department of Chemistry

# Mass Spectrum Molecular Formula Report

**Analysis Info**

| | | | |
|---|---|---|---|
| Analysis Name | F:\Data\2022MAR20\Mustafa_000070.d | Acquisition Date | 3/23/2022 3:00:37 PM |
| Method | ESI_pos_20181024 | Operator | Bruker_PC |
| Sample Name | AM255 | Instrument | apex-IV |
| Comment | CHCl3+ACN | | |

**Acquisition Parameter**

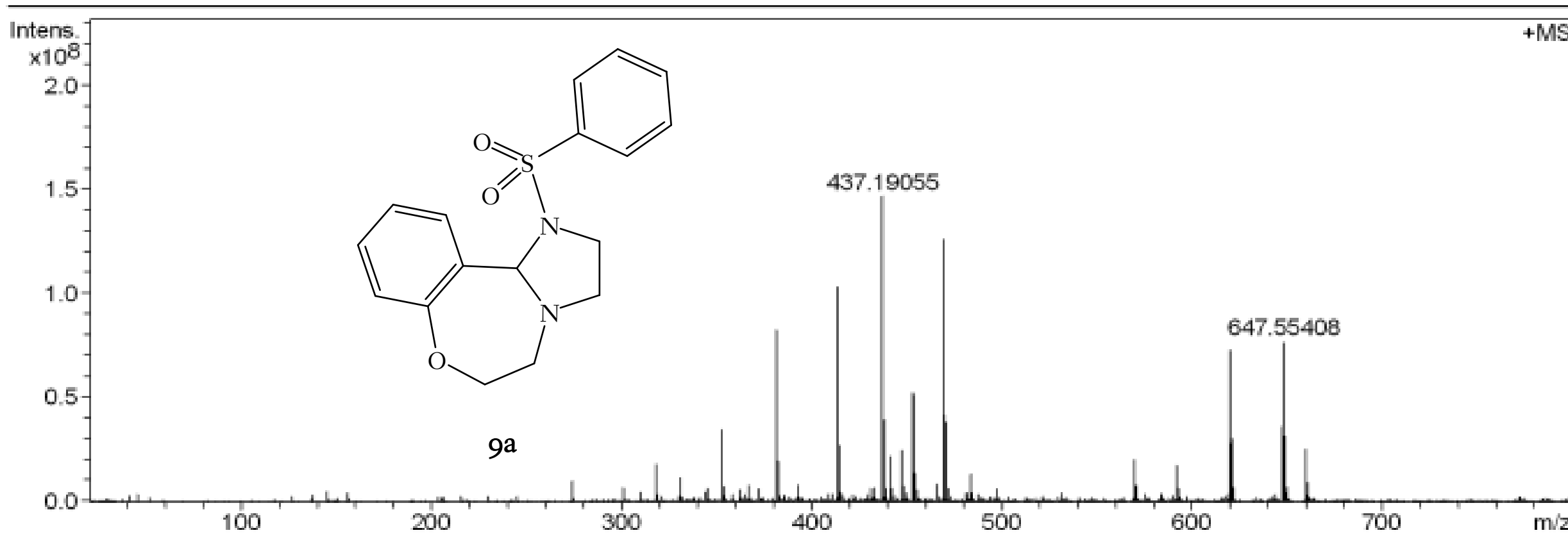


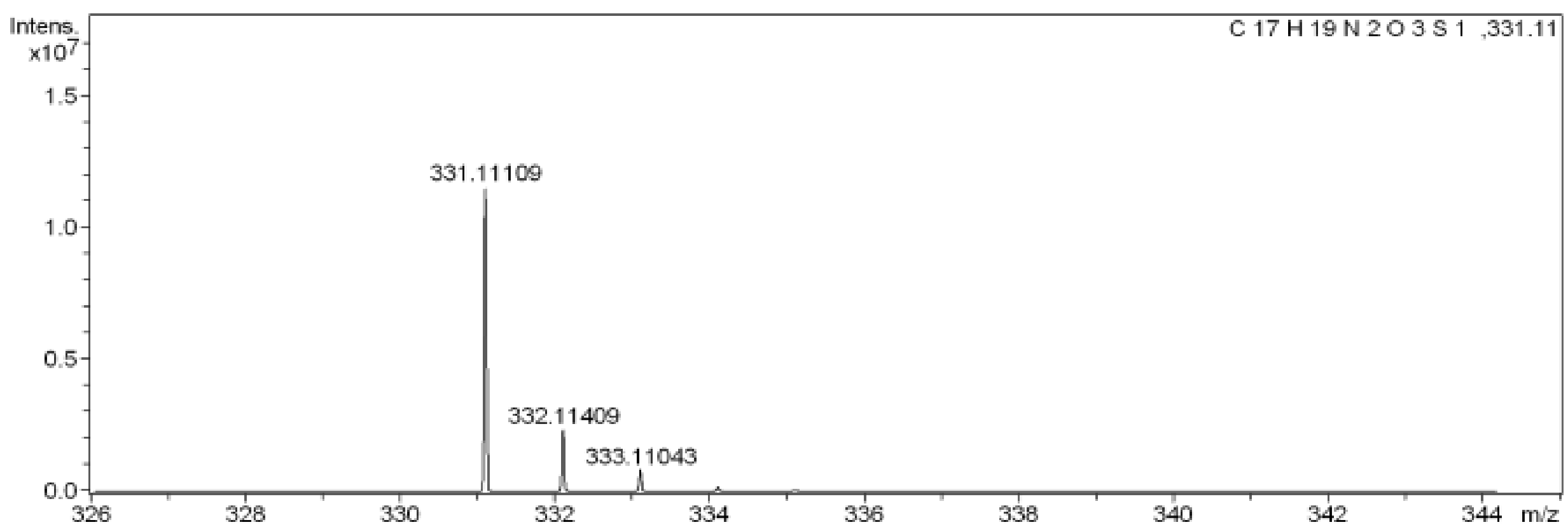


| Sum Formula | Sigma | m/z | Err [ppm] | Mean Err [ppm] | Err [mDa] | rdb | N Rule | e⁻ |
|---|---|---|---|---|---|---|---|---|
| C 17 H 19 N 2 O 3 S 1 | 0.013 | 331.11109 | 0.07 | 0.53 | 0.02 | 9.50 | ok | even |

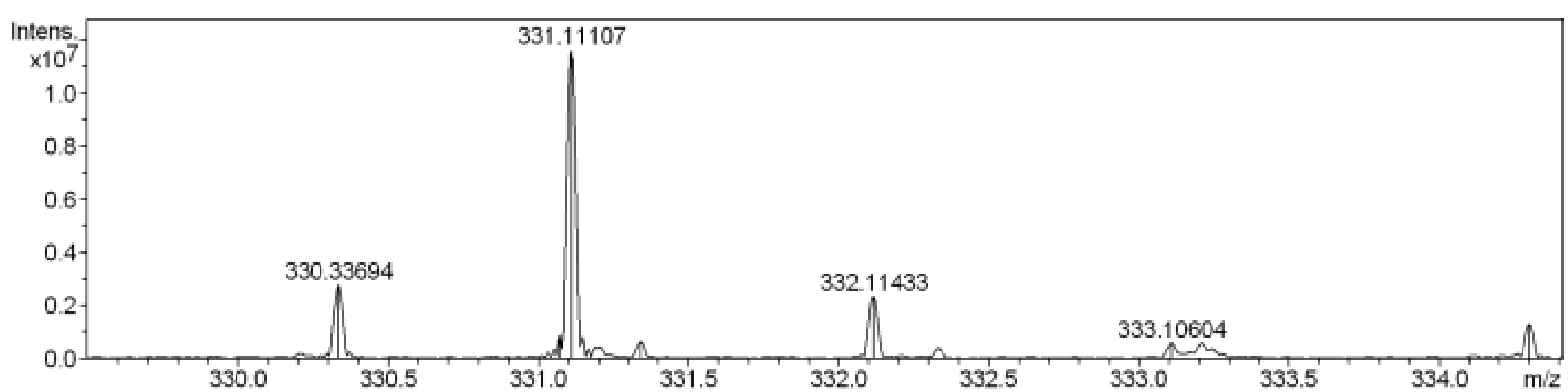

Fig.S3. HRMS spectrum of compound 9a

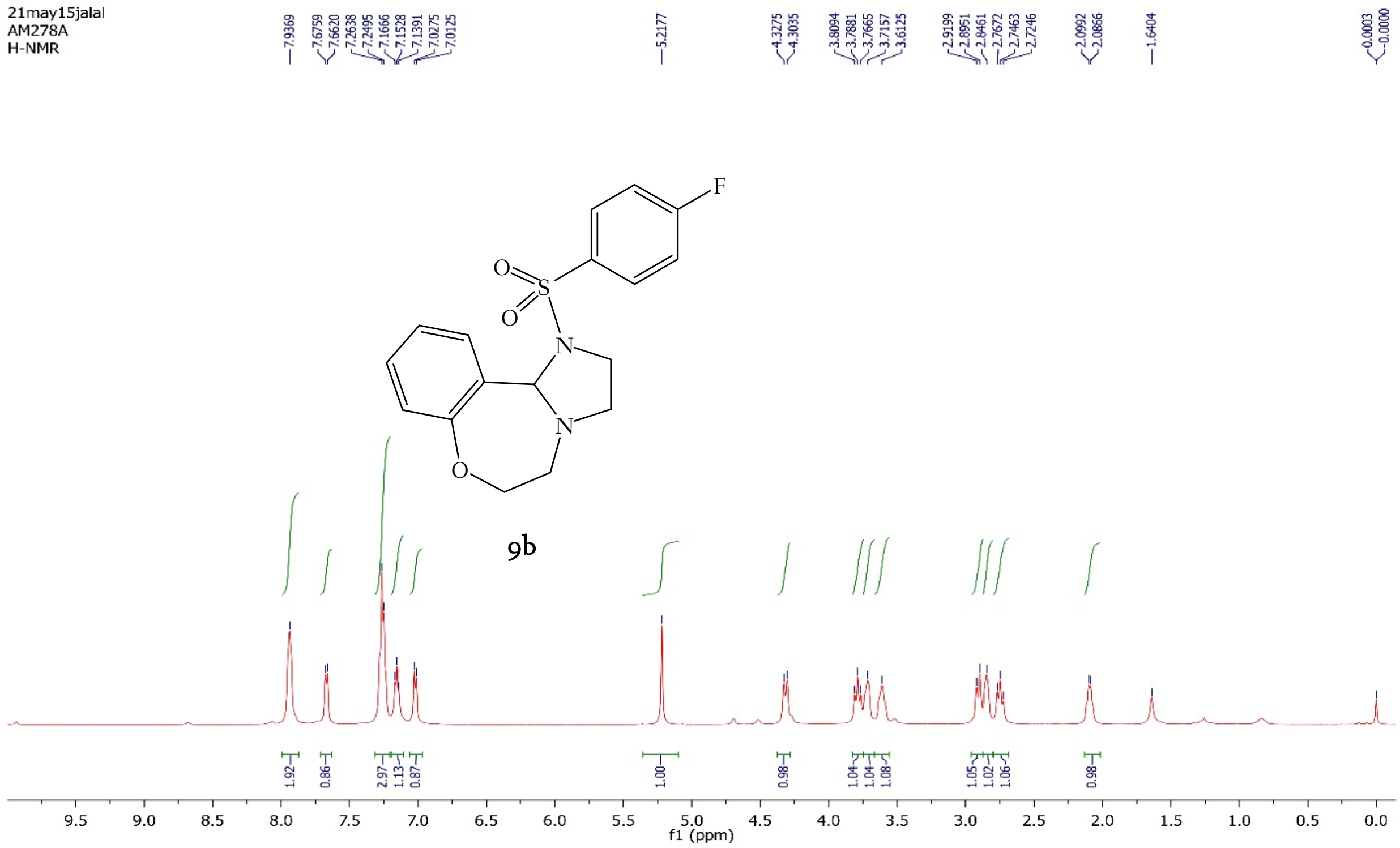


Fig.S4. $^{1}$H NMR spectrum of compound 9b $CDCl_3$

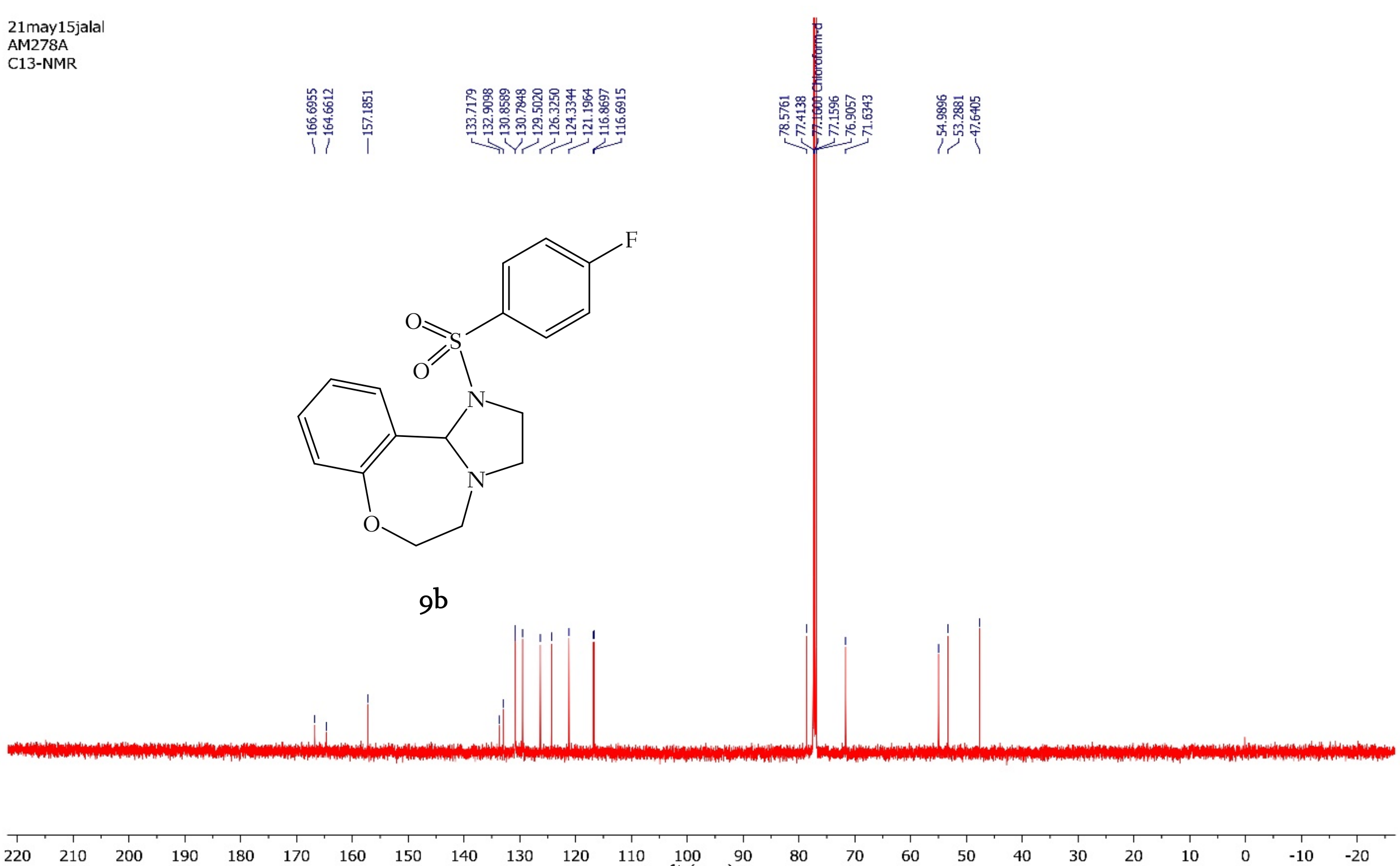

Fig.S5. $^{13}C$ NMR spectrum of compound 9b $CDCl_3$

**The University of Jordan**

**Faculty of science**

**Department of Chemistry**

## Mass Spectrum Molecular Formula Report

**Analysis Info**

| | | | |
|---|---|---|---|
| Analysis Name | F:\Data\2022MAR20\Mustafa_000045.d | Acquisition Date | 3/23/2022 1:48:08 PM |
| Method | ESI_pos_20181024 | Operator | Bruker_PC |
| Sample Name | AM278 | Instrument | apex-IV |
| Comment | CHCl3+ACN | | |

**Acquisition Parameter**

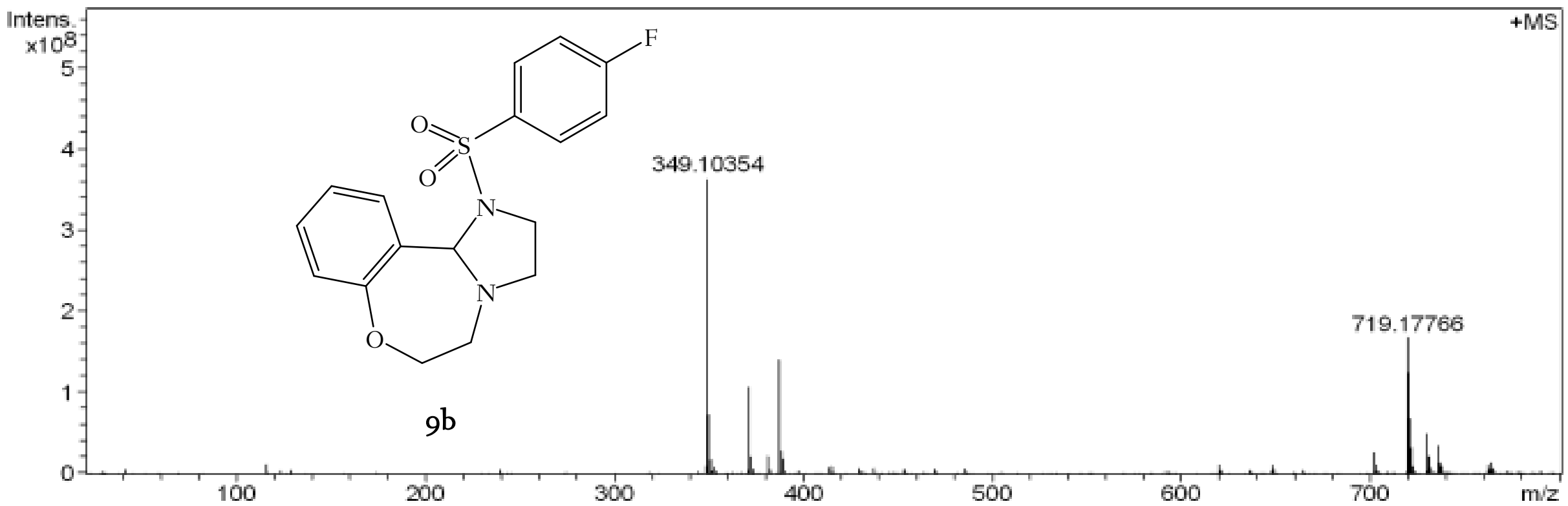


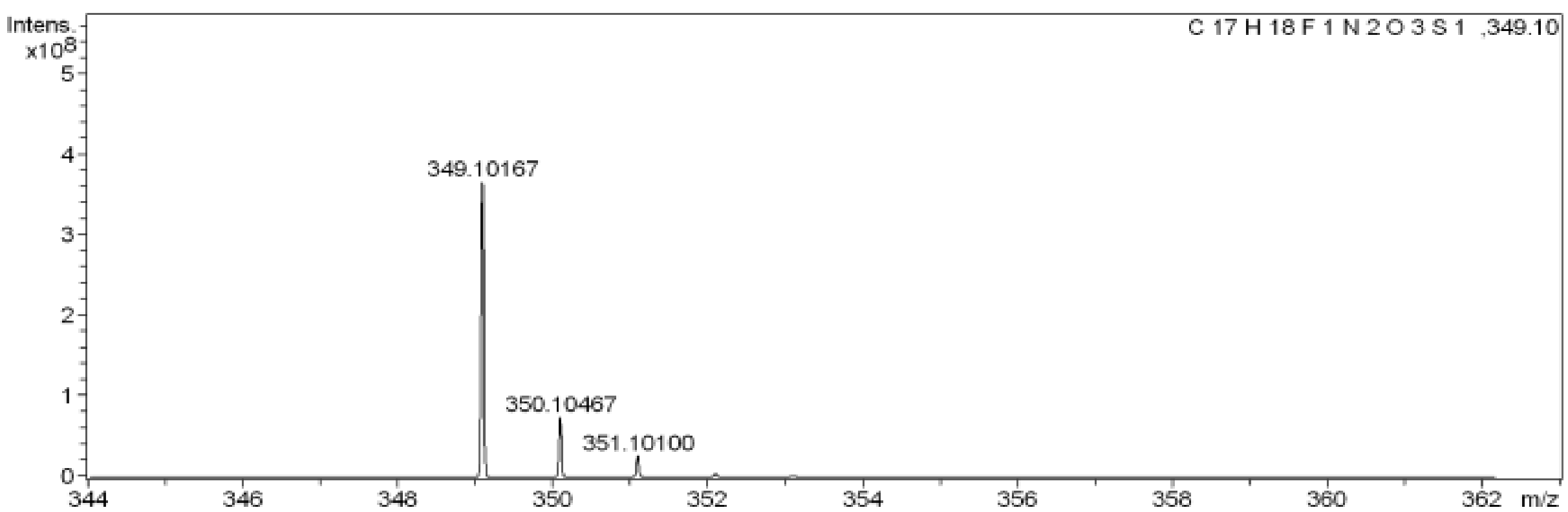


| Sum Formula | Sigma | m/z | Err [ppm] | Mean Err [ppm] | Err [mDa] | rdb | N Rule | e⁻ |
|---|---|---|---|---|---|---|---|---|
| C 17 H 18 F 1 N 2 O 3 S 1 | 0.011 | 349.10167 | -5.37 | -5.05 | -1.88 | 9.50 | ok | even |

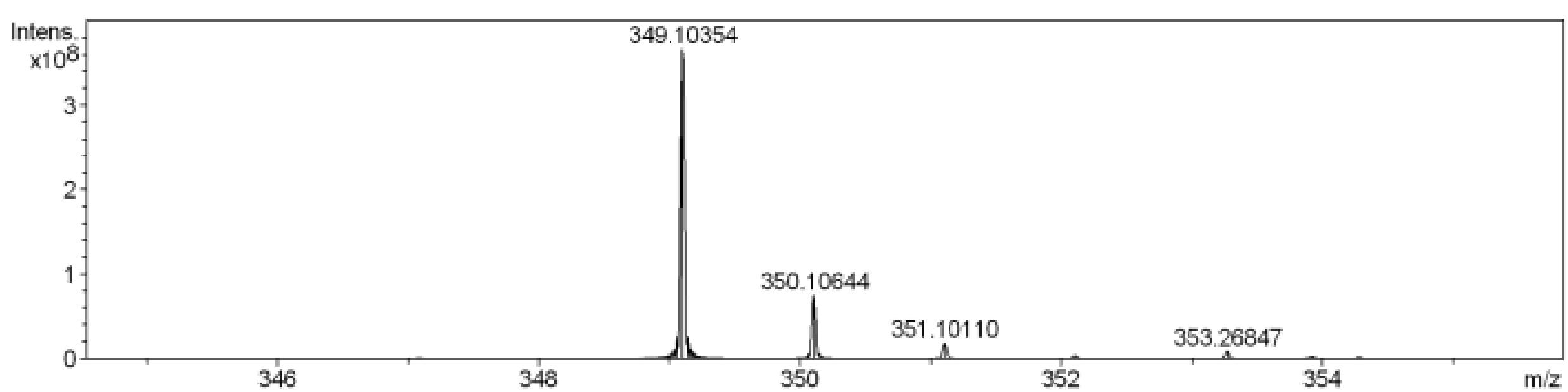


Fig.S6. HRMS spectrum of compound 9b

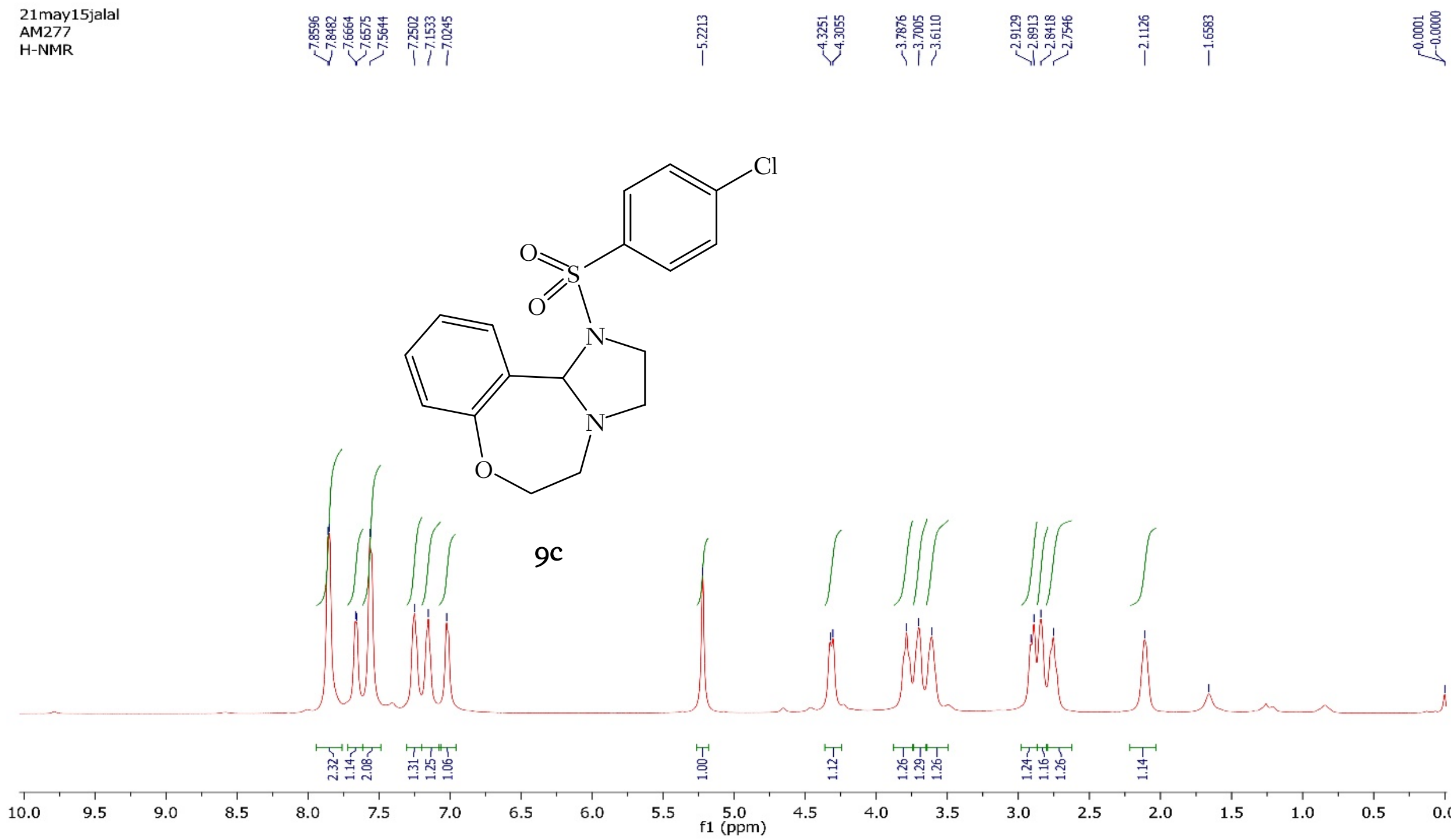


Fig.S7. $^{1}$H NMR spectrum of compound 9c $CDCl_3$

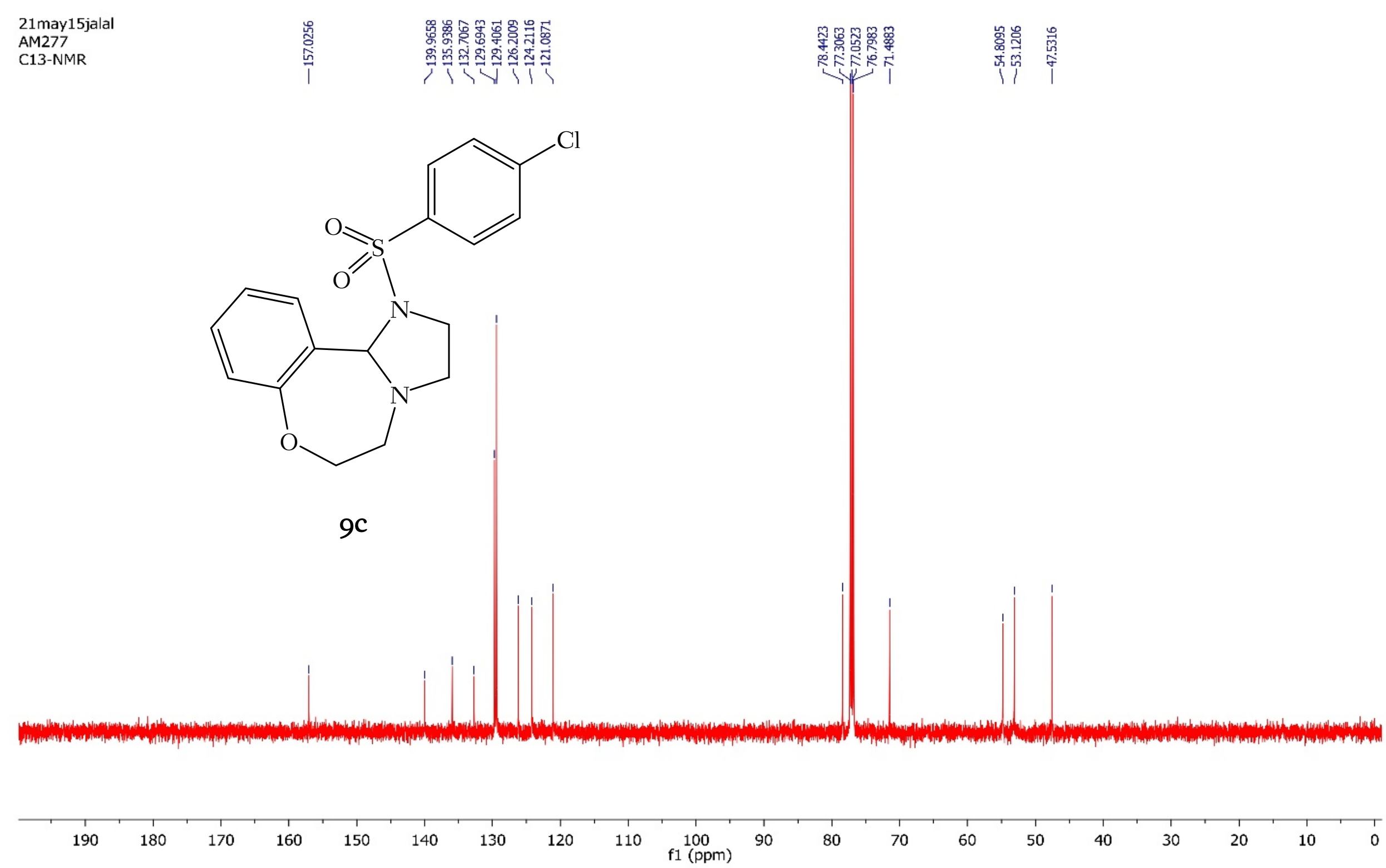

Fig.S8. $^{13}$C NMR spectrum of compound 9c $CDCl_3$

**The University of Jordan**

**Faculty of science**

**Department of Chemistry**

## Mass Spectrum Molecular Formula Report

| | | | |
|---|---|---|---|
| **Analysis Info** | | Acquisition Date | 3/23/2022 2:42:57 PM |
| Analysis Name | F:\Data\2022MAR20\Mustafa_000064.d | | |
| Method | ESI_pos_20181024 | Operator | Bruker_PC |
| Sample Name | AM277 | Instrument | apex-IV |
| Comment | CHCl3+ACN | | |

**Acquisition Parameter**

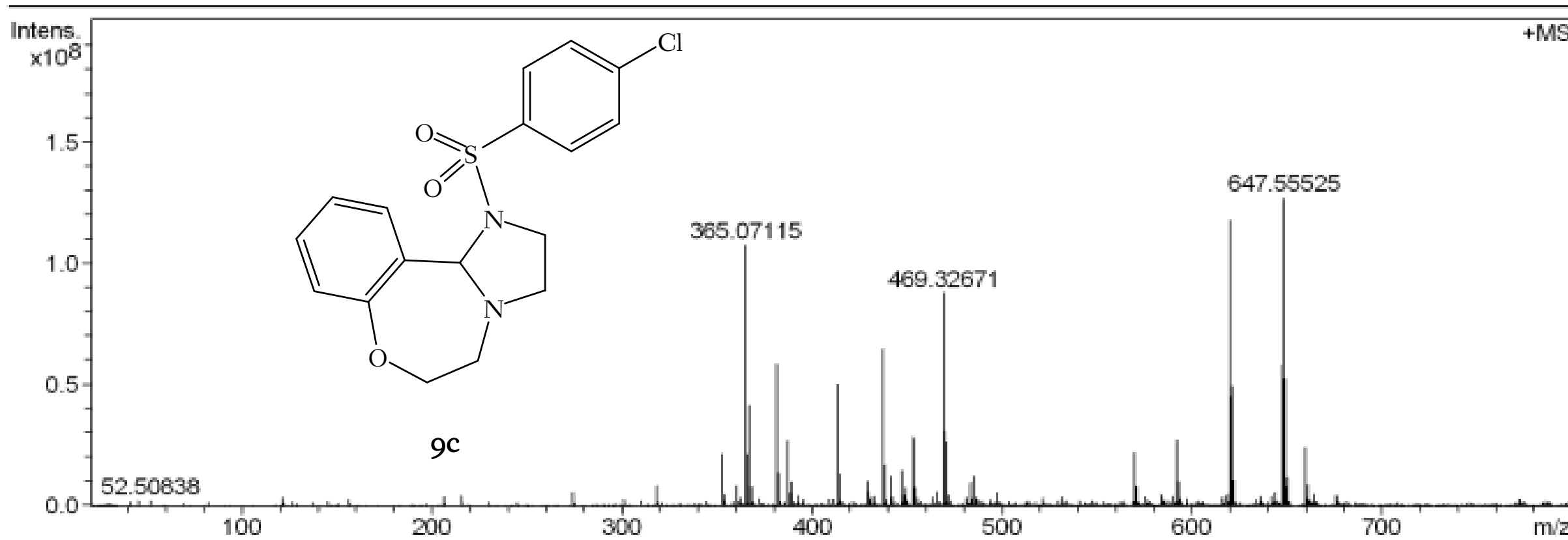


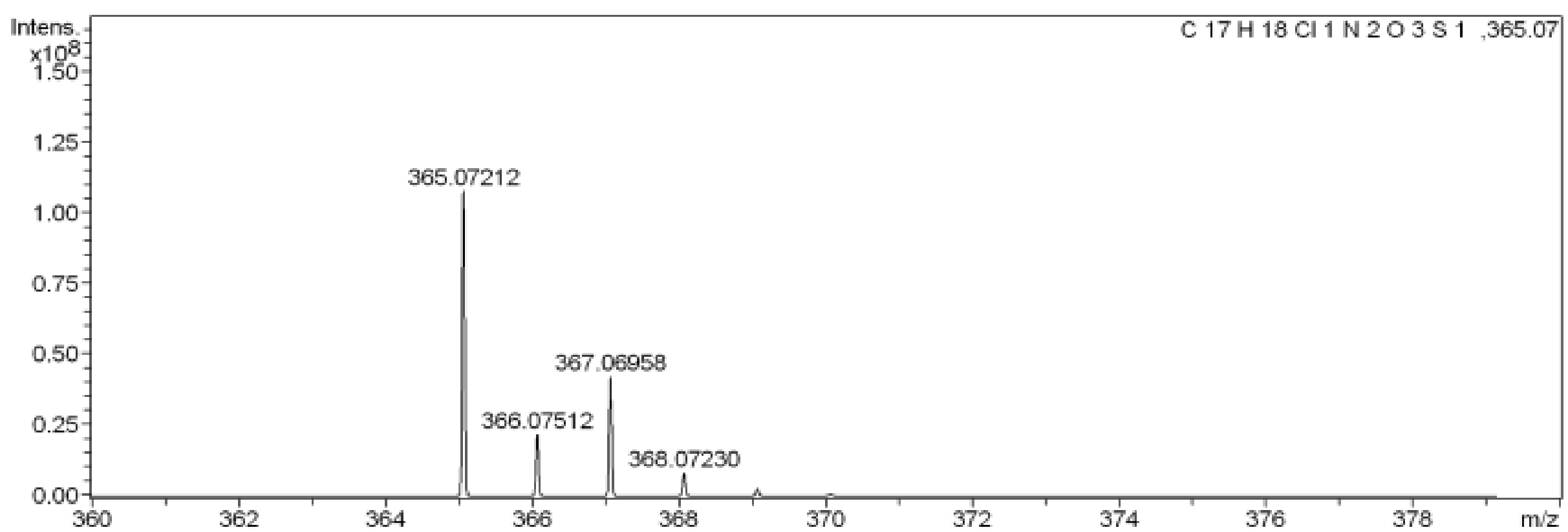


| Sum Formula | Sigma | m/z | Err [ppm] | Mean Err [ppm] | Err [mDa] | rdb | N Rule | e⁻ |
|---|---|---|---|---|---|---|---|---|
| C 17 H 18 Cl 1 N 2 O 3 S 1 | 0.003 | 365.07212 | 2.66 | 1.97 | 0.97 | 9.50 | ok | even |

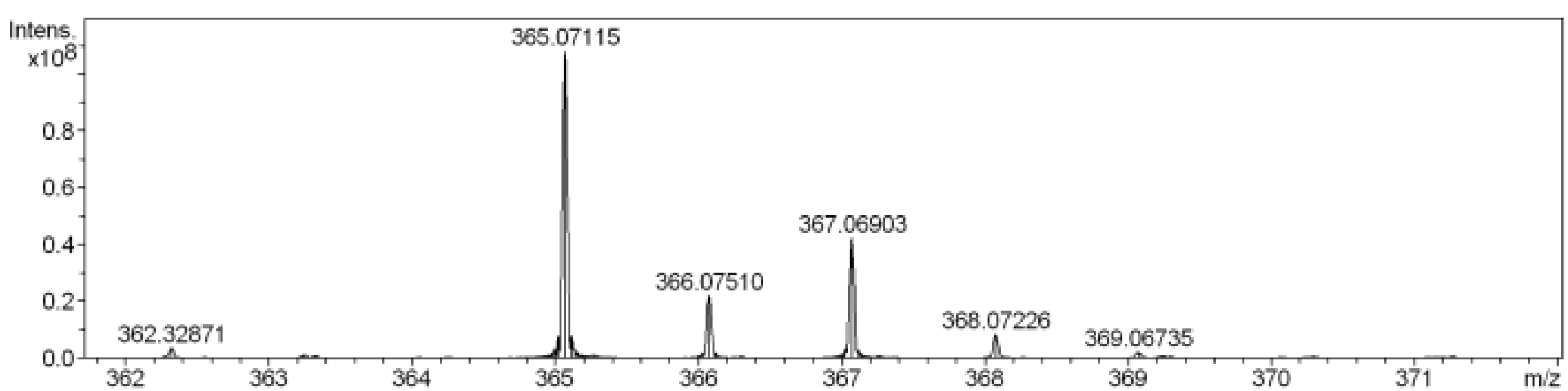

Fig.S9. HRMS spectrum of compound 9c

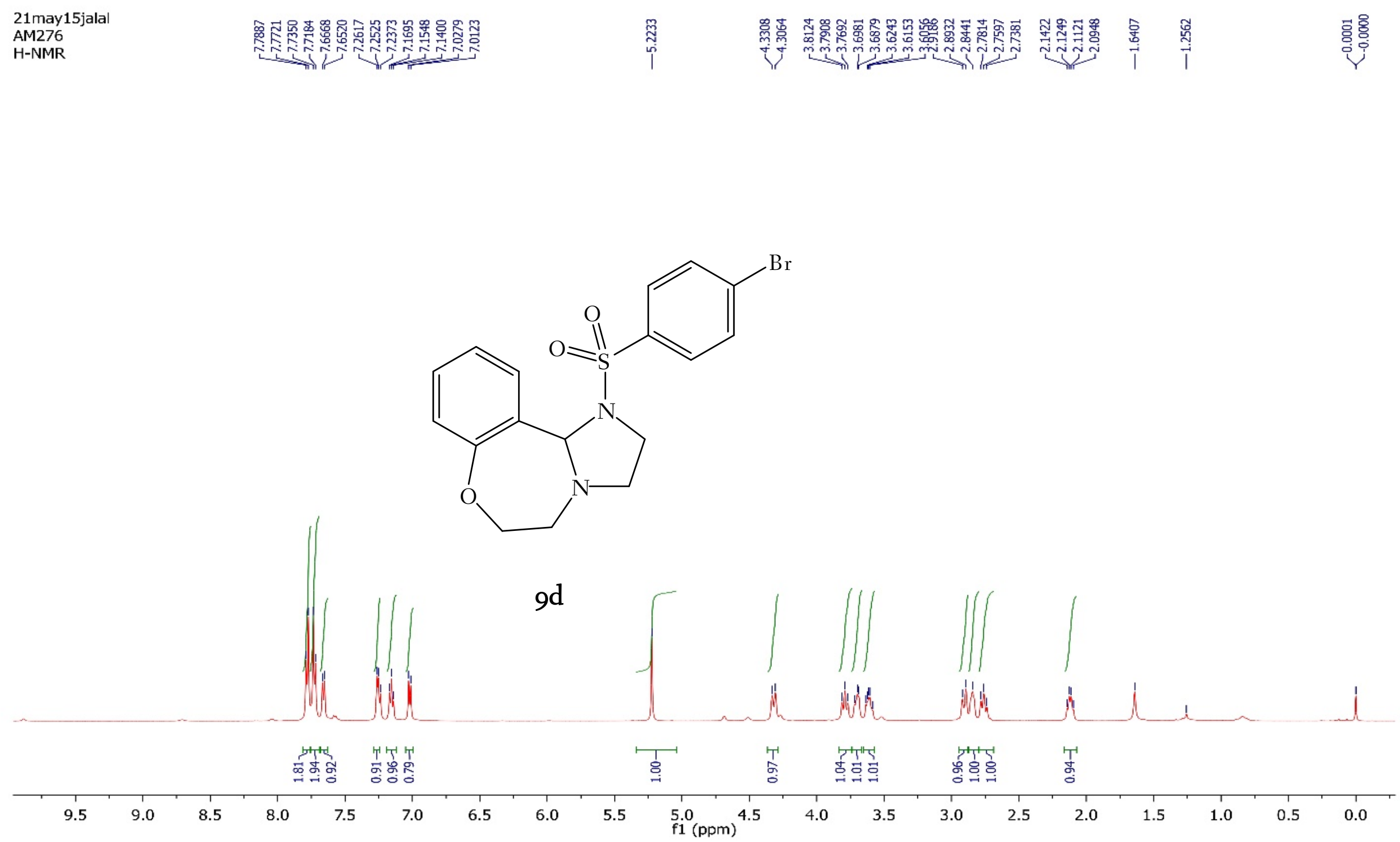


Fig.S10. $^{1}$H NMR spectrum of compound 9d $CDCl_3$

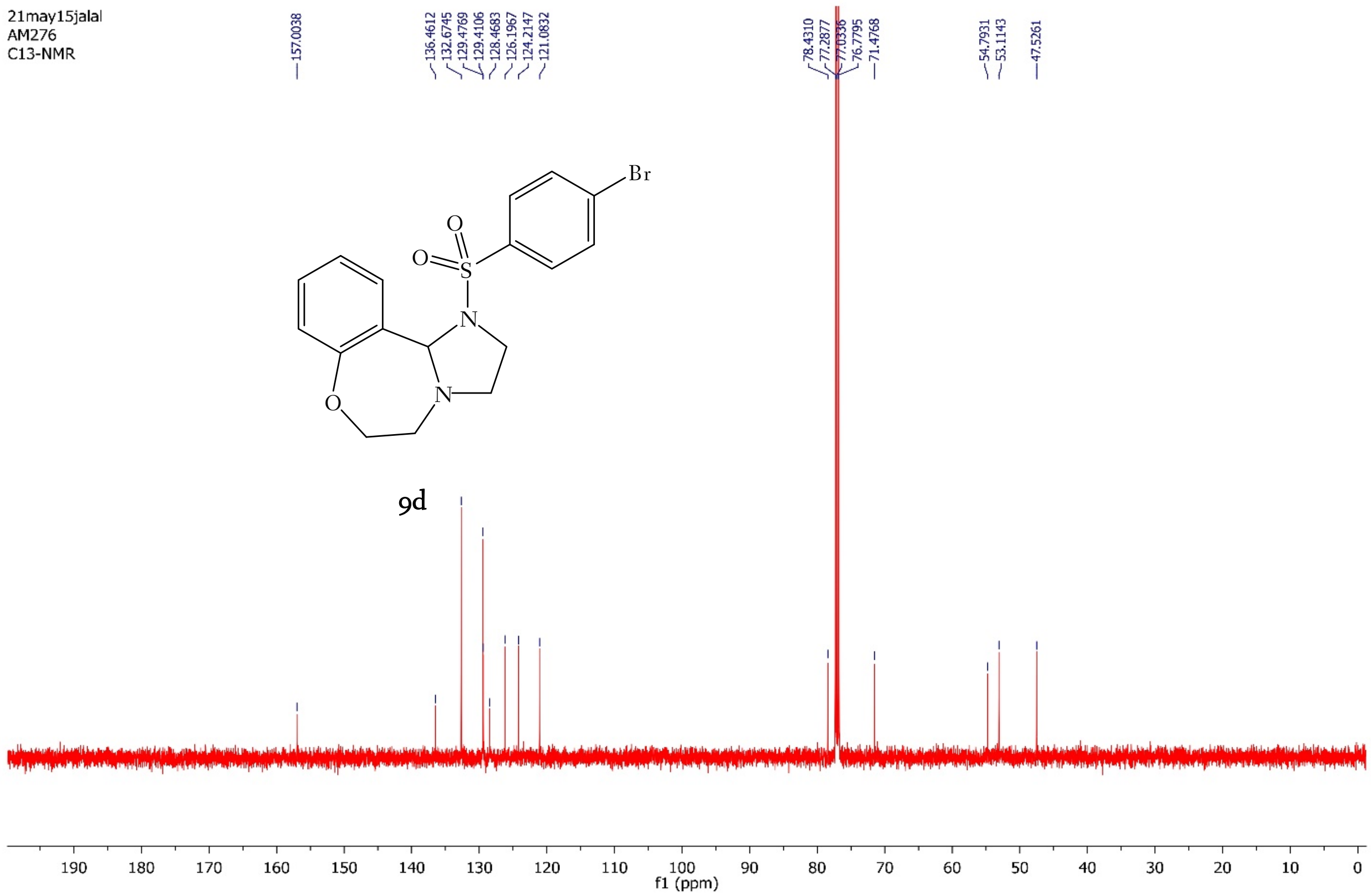


Fig.S11. $^{13}$C NMR spectrum of compound 9d $CDCl_3$

The University of Jordan
Faculty of science
Department of Chemistry

# Mass Spectrum Molecular Formula Report

**Analysis Info**

| | | | |
|---|---|---|---|
| Analysis Name | F:\Data\2022MAR08\Mustafa_000047.d | Acquisition Date | 3/13/2022 2:17:52 PM |
| Method | ESI_pos_20181024 | Operator | Bruker_PC |
| Sample Name | AM276 | Instrument | apex-IV |
| Comment | CHCl3+ACN | | |

**Acquisition Parameter**

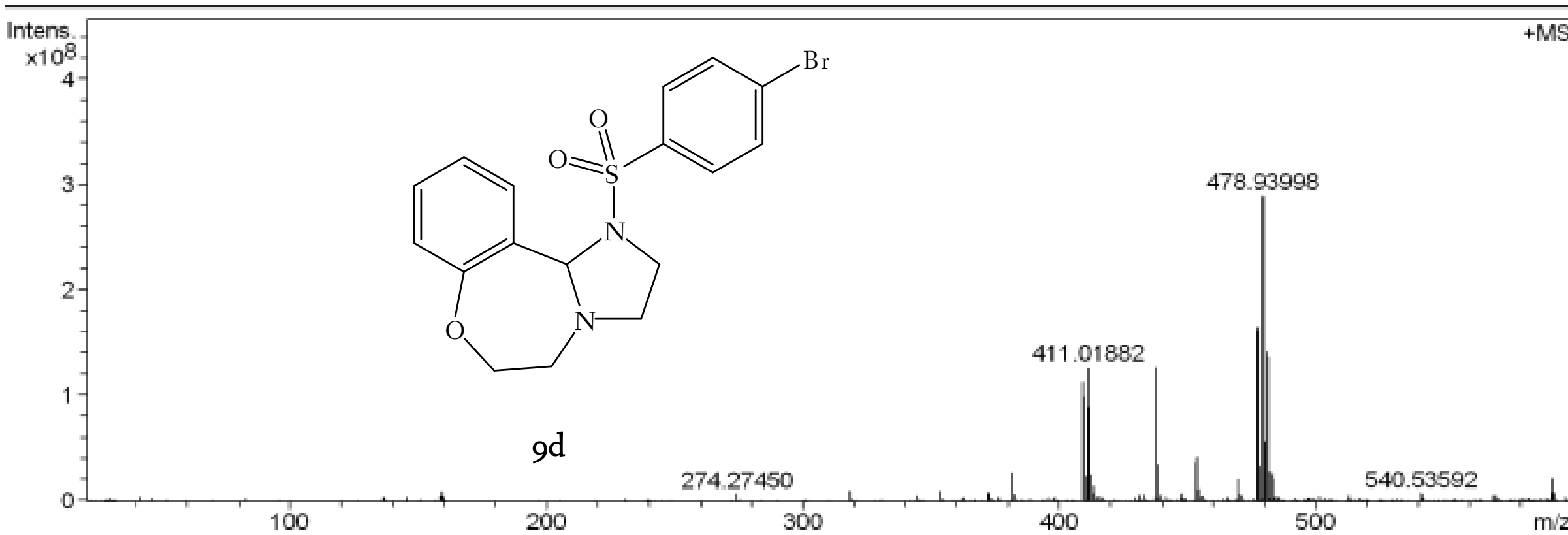


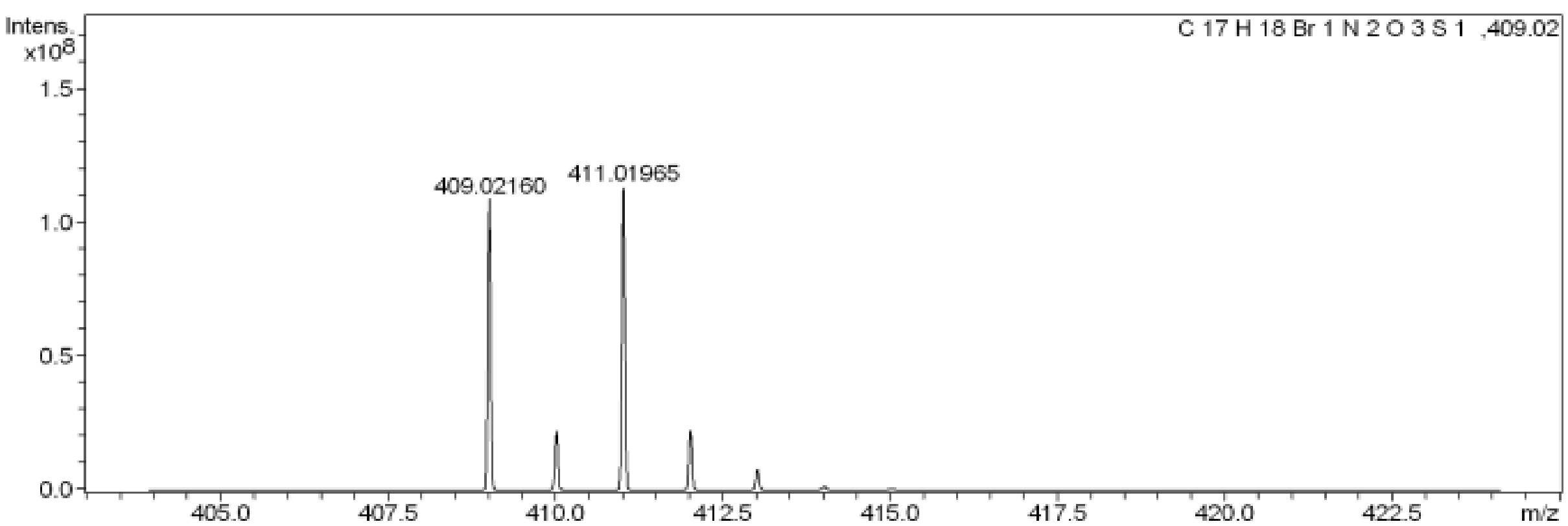


| Sum Formula | Sigma | m/z | Err [ppm] | Mean Err [ppm] | Err [mDa] | rdb | N Rule | e⁻ |
|---|---|---|---|---|---|---|---|---|
| C 17 H 18 Br 1 N 2 O 3 S 1 | 0.028 | 409.02160 | 4.22 | 2.70 | 1.73 | 9.50 | ok | even |

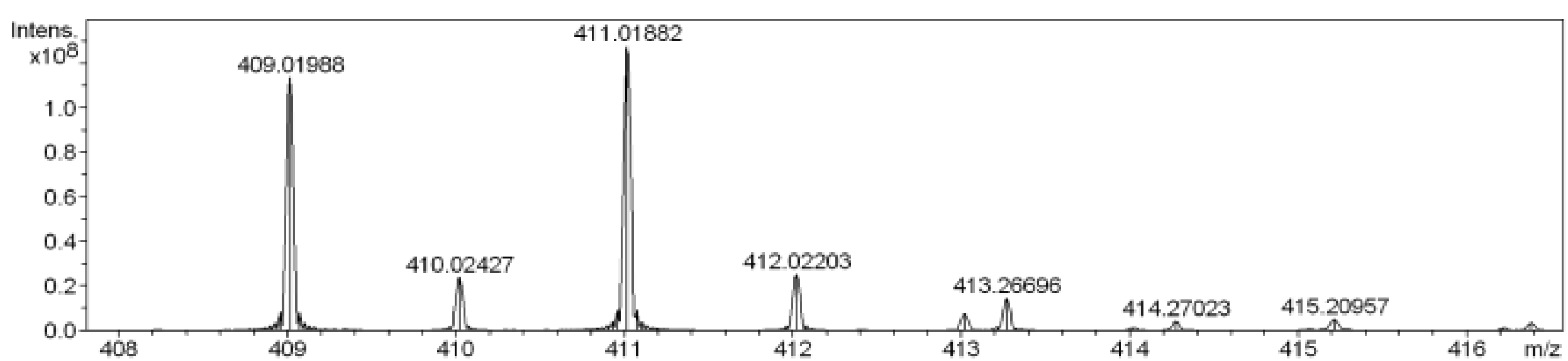


Fig.S12. HRMS spectrum of compound 9d

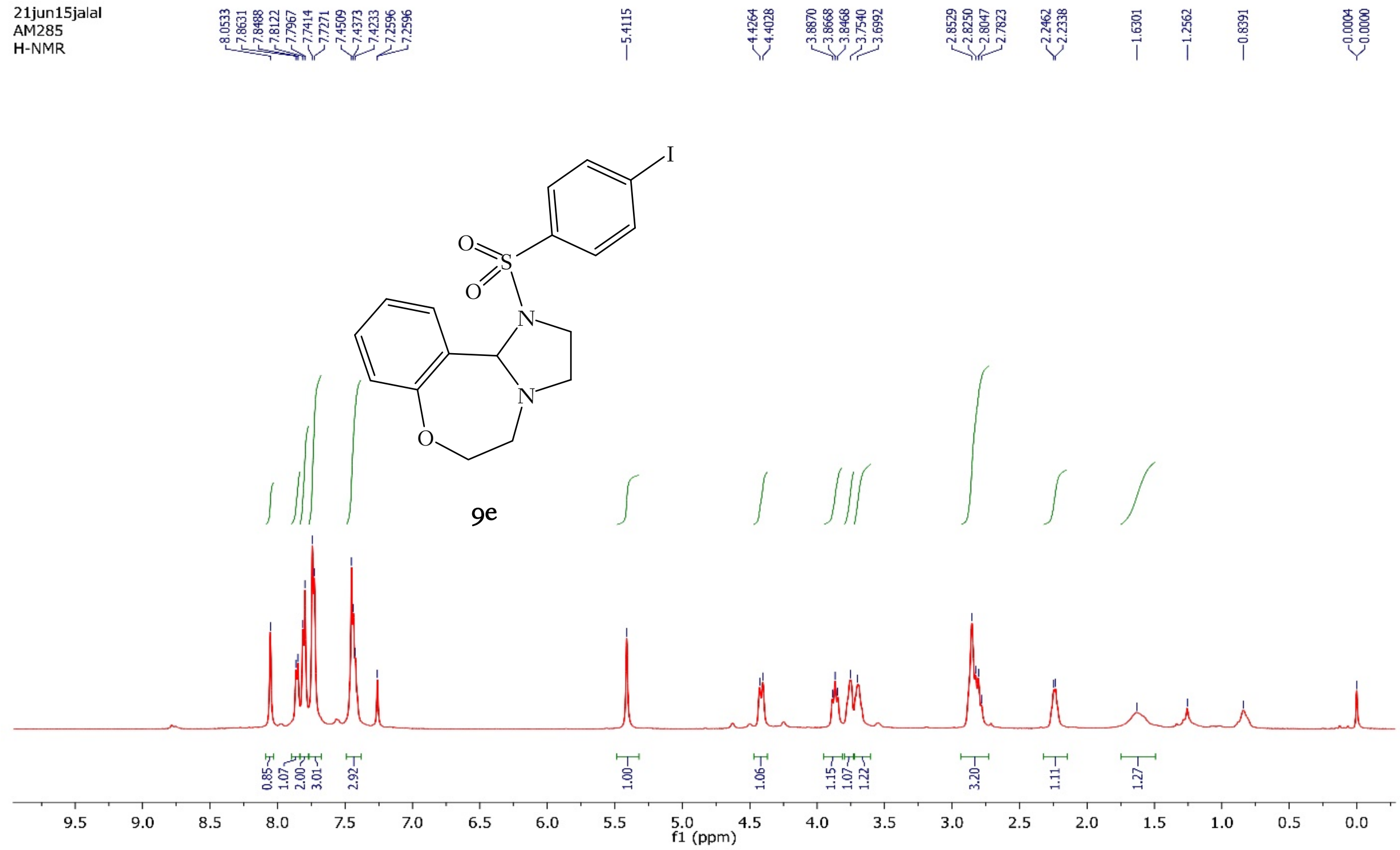

Fig.S13. $^{1}$H NMR spectrum of compound 9e $CDCl_3$

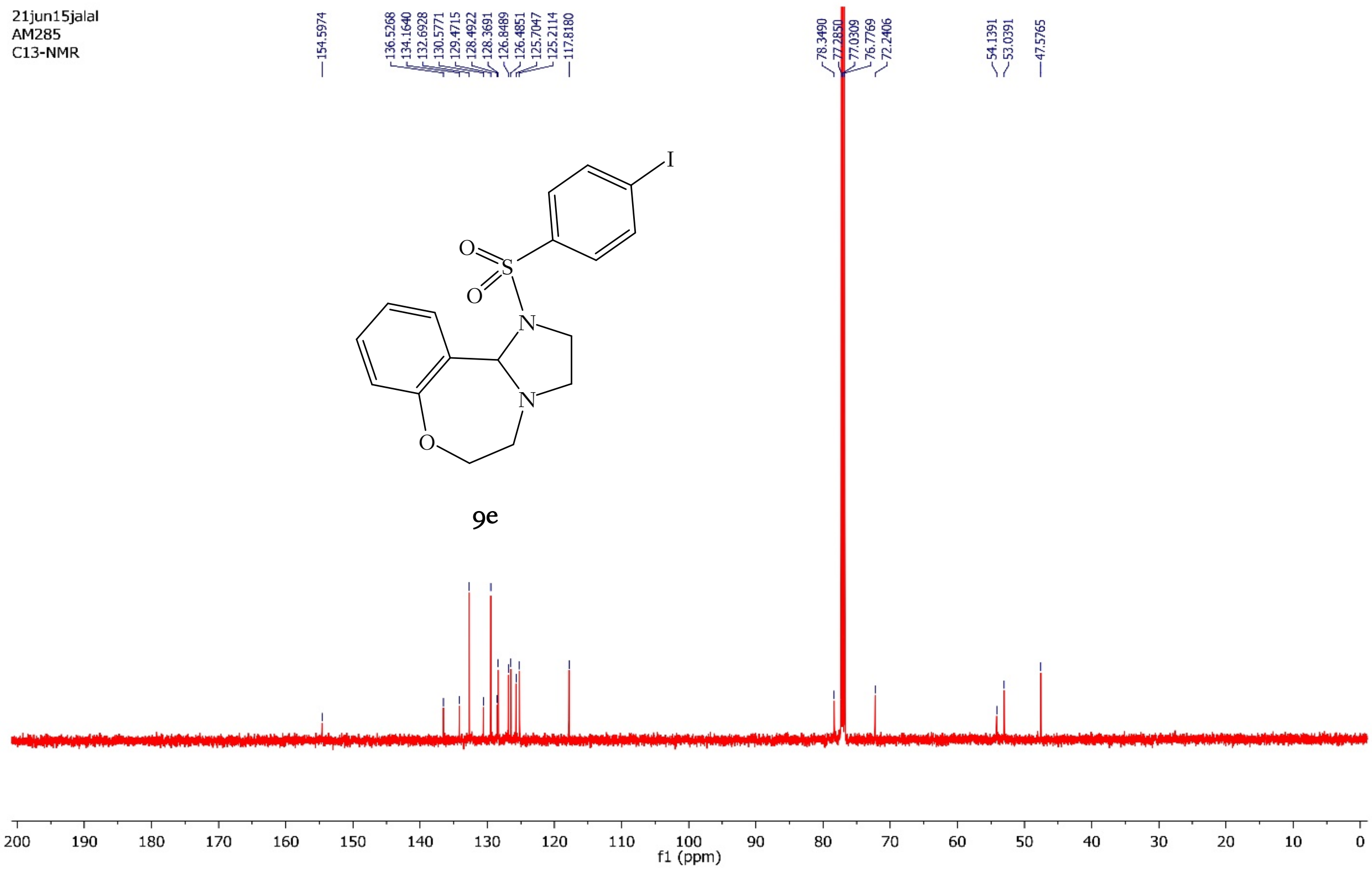


9e

Fig.S14. 13C NMR spectrum of compound 9e $CDCl_3$

**The University of Jordan**

**Faculty of science**

**Department of Chemistry**

# Mass Spectrum Molecular Formula Report

| | | | |
|---|---|---|---|
| **Analysis Info** | | Acquisition Date | 3/13/2022 10:11:04 AM |
| Analysis Name | F:\Data\2022MAR08\Mustafa_000025.d | | |
| Method | ESI_pos_20181024 | Operator | Bruker_PC |
| Sample Name | AM283 | Instrument | apex-IV |
| Comment | CHCl3+ACN | | |

**Acquisition Parameter**

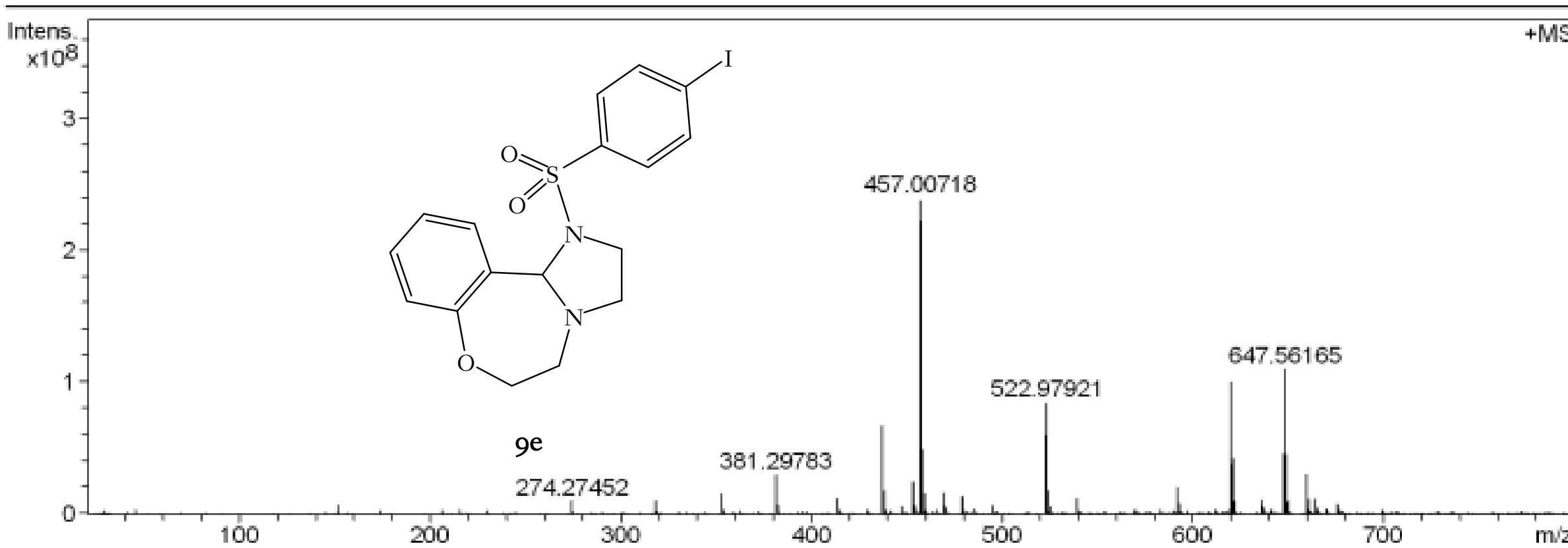


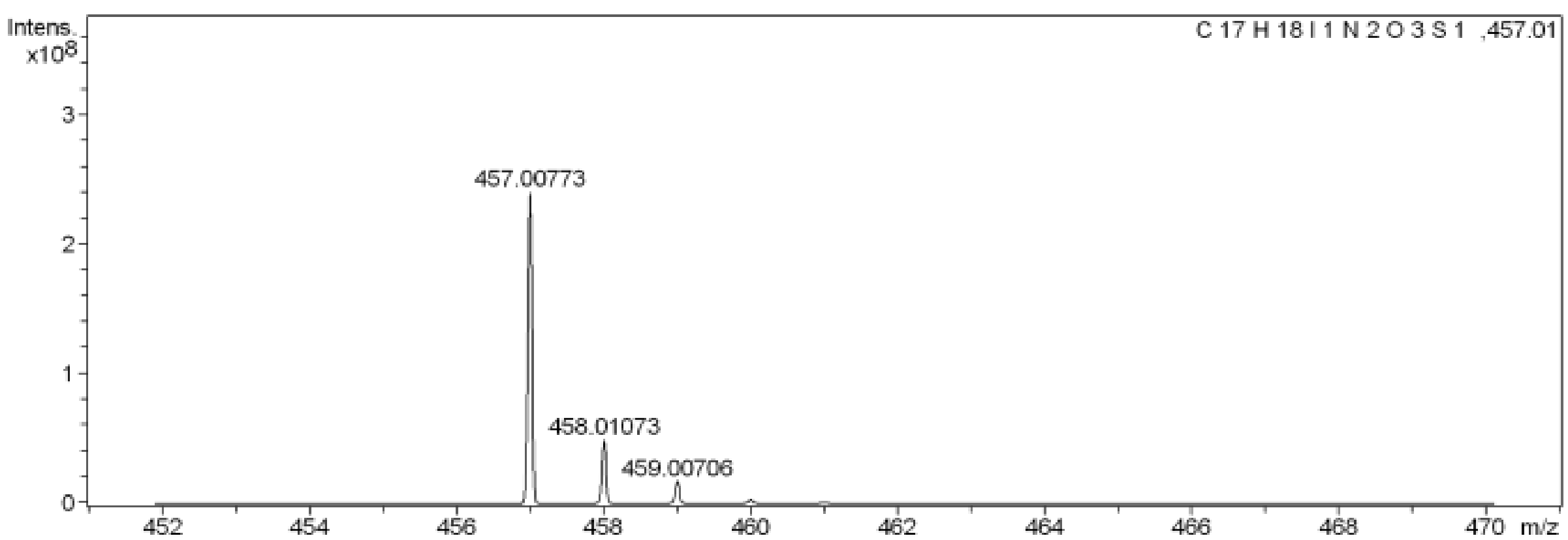


| Sum Formula | Sigma | m/z | Err [ppm] | Mean Err [ppm] | Err [mDa] | rdb | N Rule | e⁻ |
|---|---|---|---|---|---|---|---|---|
| C 17 H 18 I 1 N 2 O 3 S 1 | 0.004 | 457.00773 | 1.20 | 0.78 | 0.55 | 9.50 | ok | even |

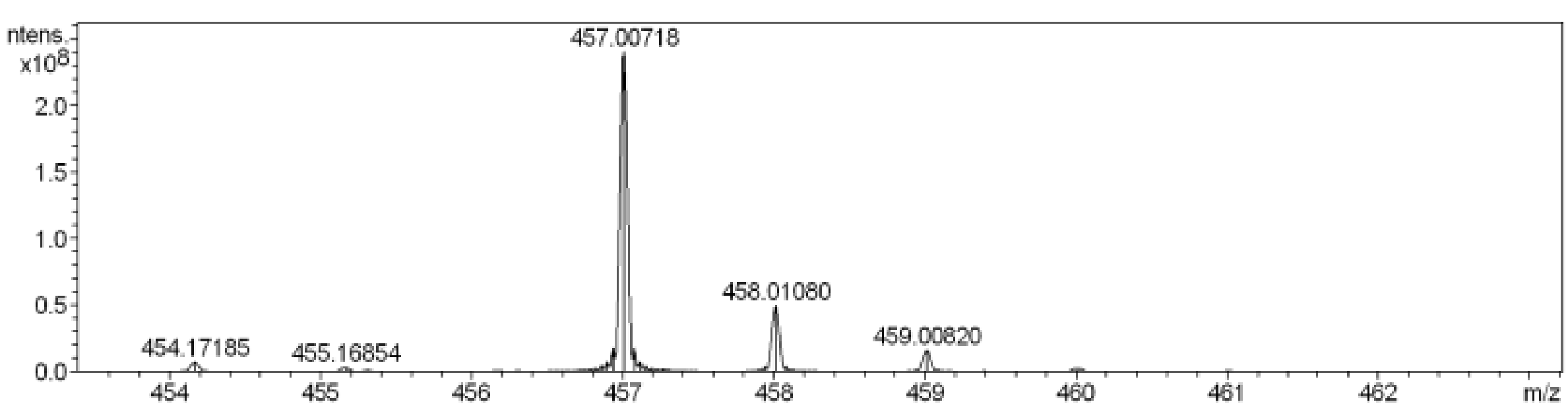


Fig.S15. HRMS spectrum of compound 9e

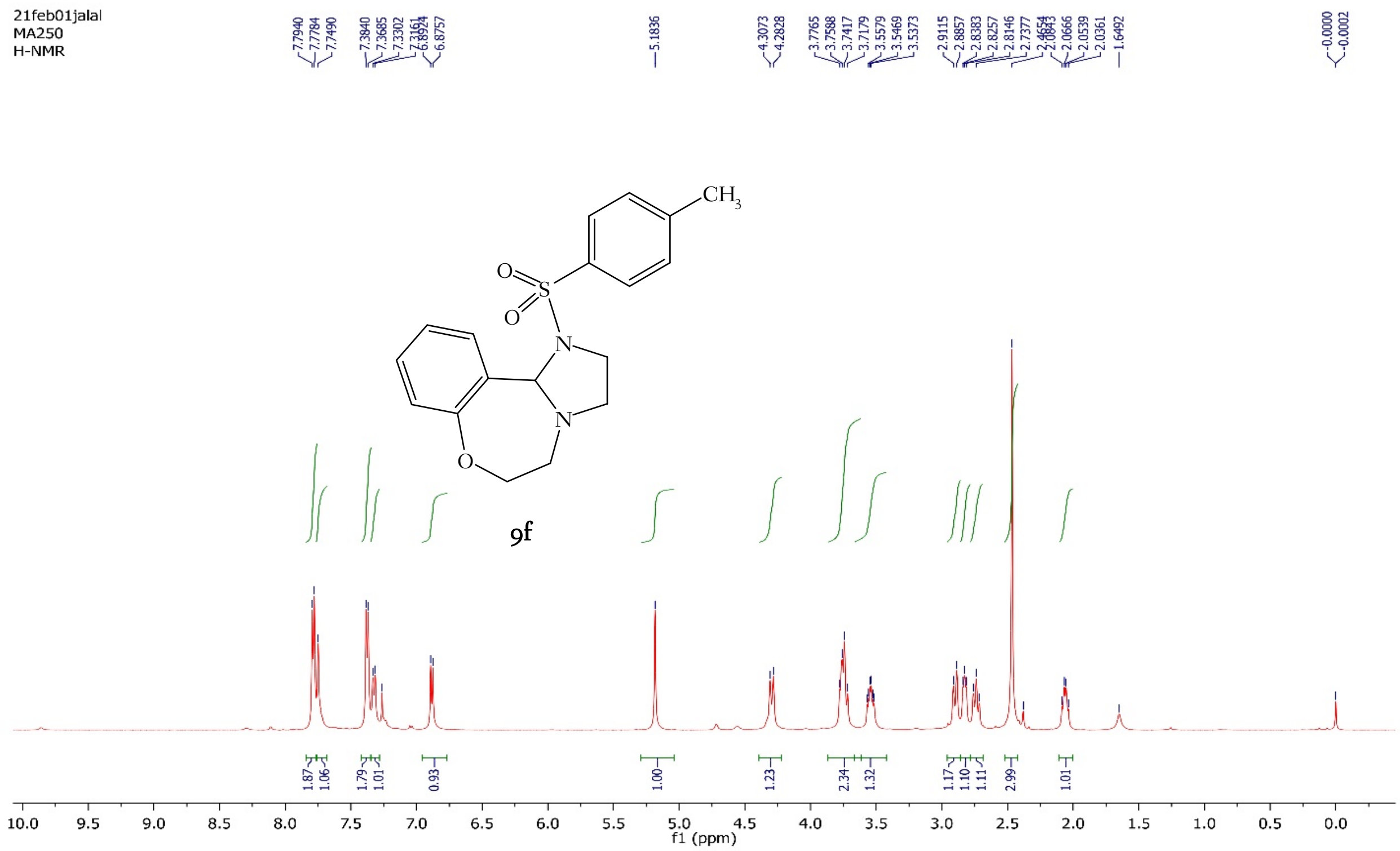


Fig.S16. $^{1}$H NMR spectrum of compound 9f $CDCl_3$

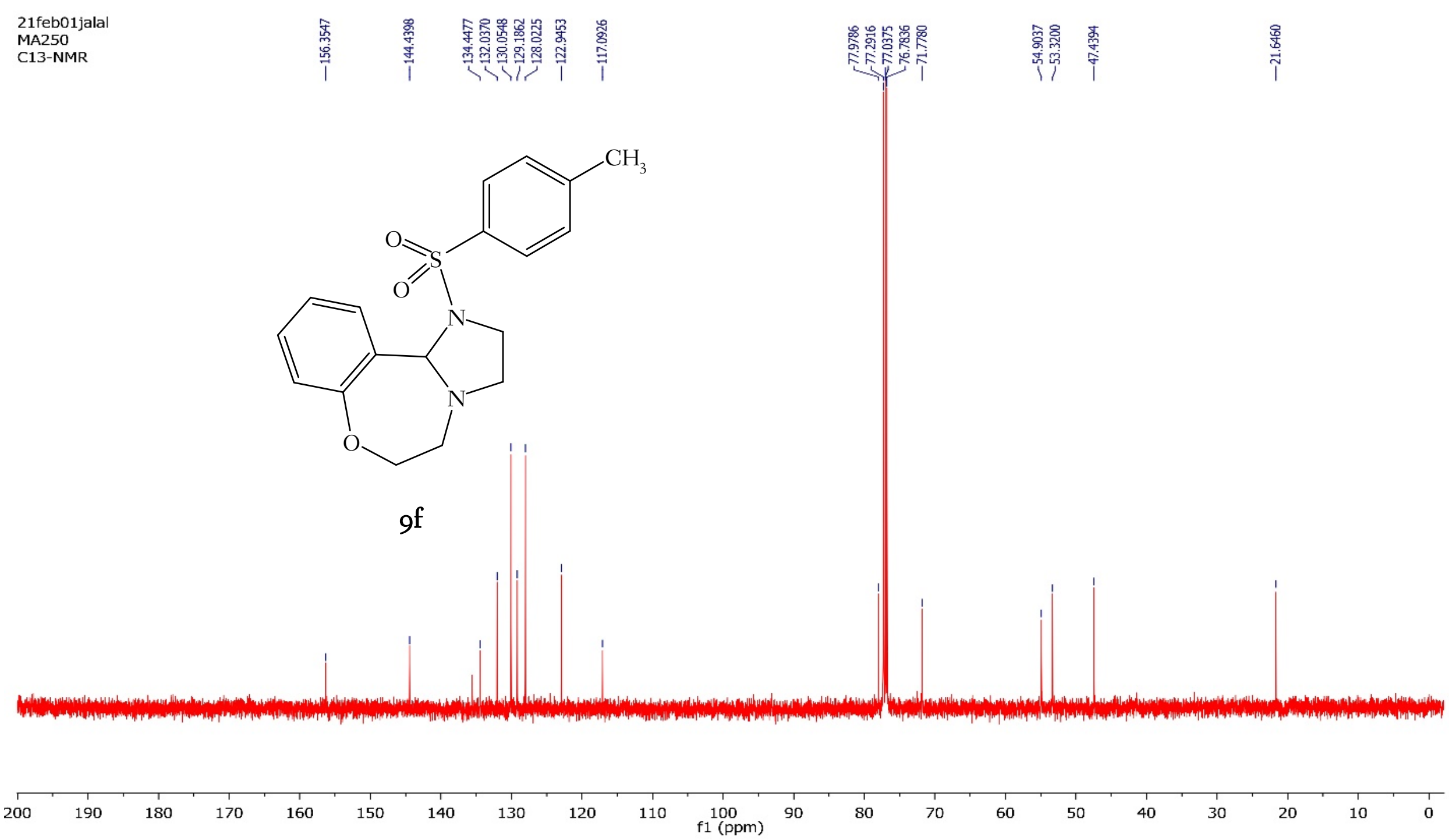


Fig.S17. $^{13}$C NMR spectrum of compound 9f $CDCl_3$

# The University of Jordan
## Faculty of science
## Department of Chemistry

### Mass Spectrum Molecular Formula Report

**Analysis Info**

| | | | |
|---|---|---|---|
| Analysis Name | F:\Data\2022MAR20\Mustafa_000069.d | Acquisition Date | 3/23/2022 2:56:32 PM |
| Method | ESI_pos_20181024 | Operator | Bruker_PC |
| Sample Name | AM250 | Instrument | apex-IV |
| Comment | CHCl3+ACN | | |

**Acquisition Parameter**

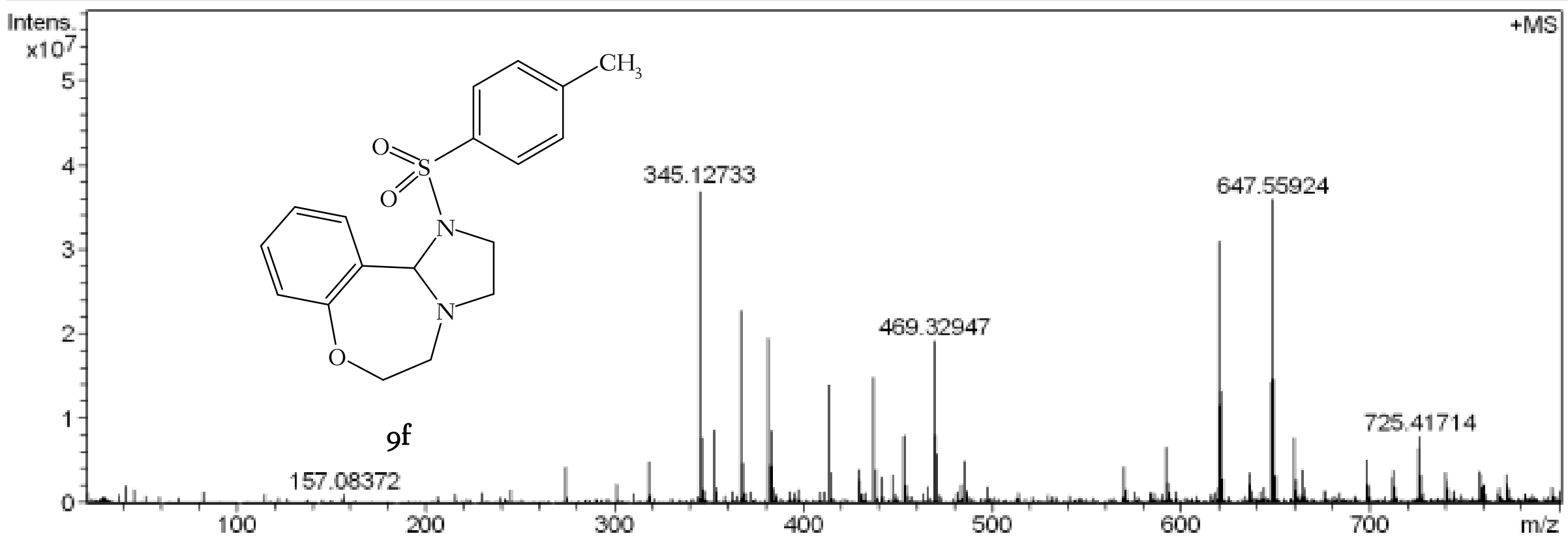


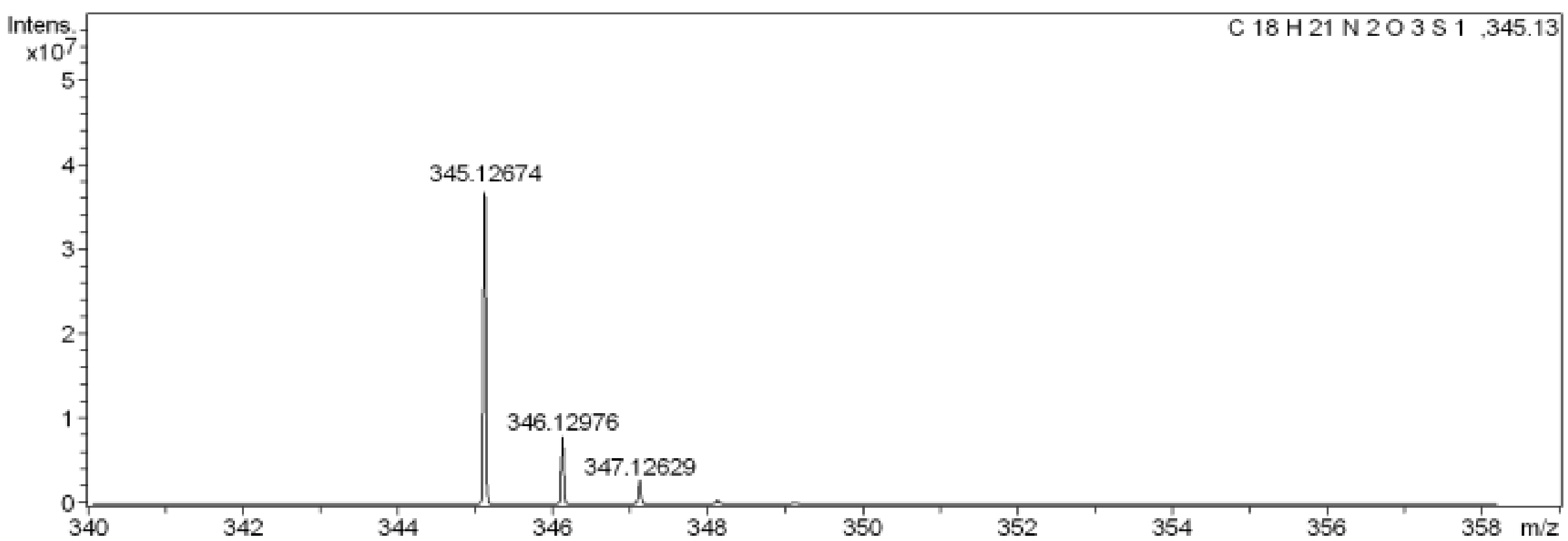


| Sum Formula | Sigma | m/z | Err [ppm] | Mean Err [ppm] | Err [mDa] | rdb | N Rule | e⁻ |
|---|---|---|---|---|---|---|---|---|
| C 18 H 21 N 2 O 3 S 1 | 0.014 | 345.12674 | -1.72 | -1.45 | -0.59 | 9.50 | ok | even |

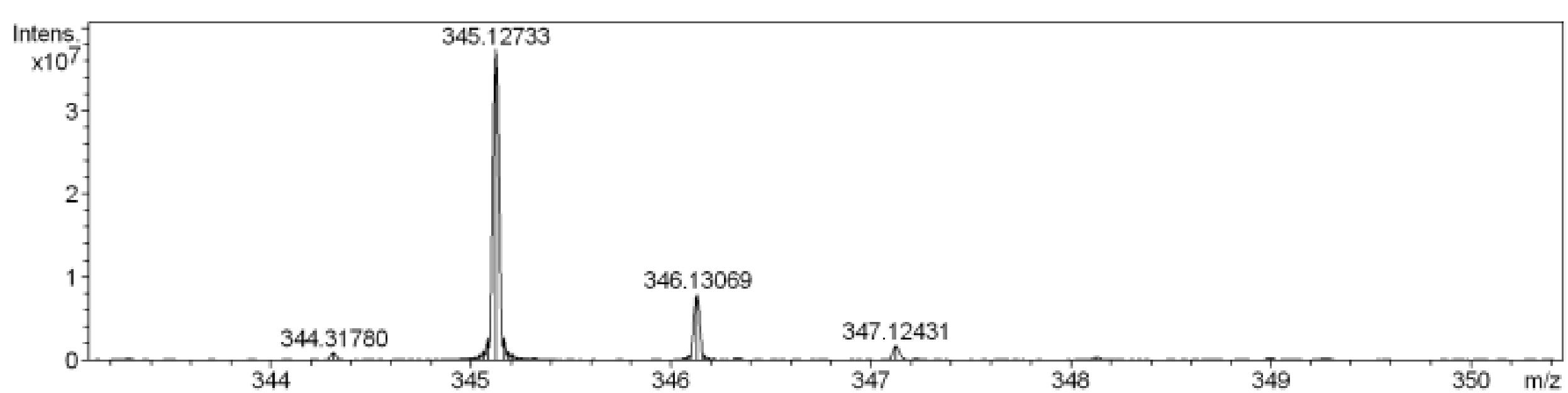


Fig.S18. HRMS spectrum of compound 9f

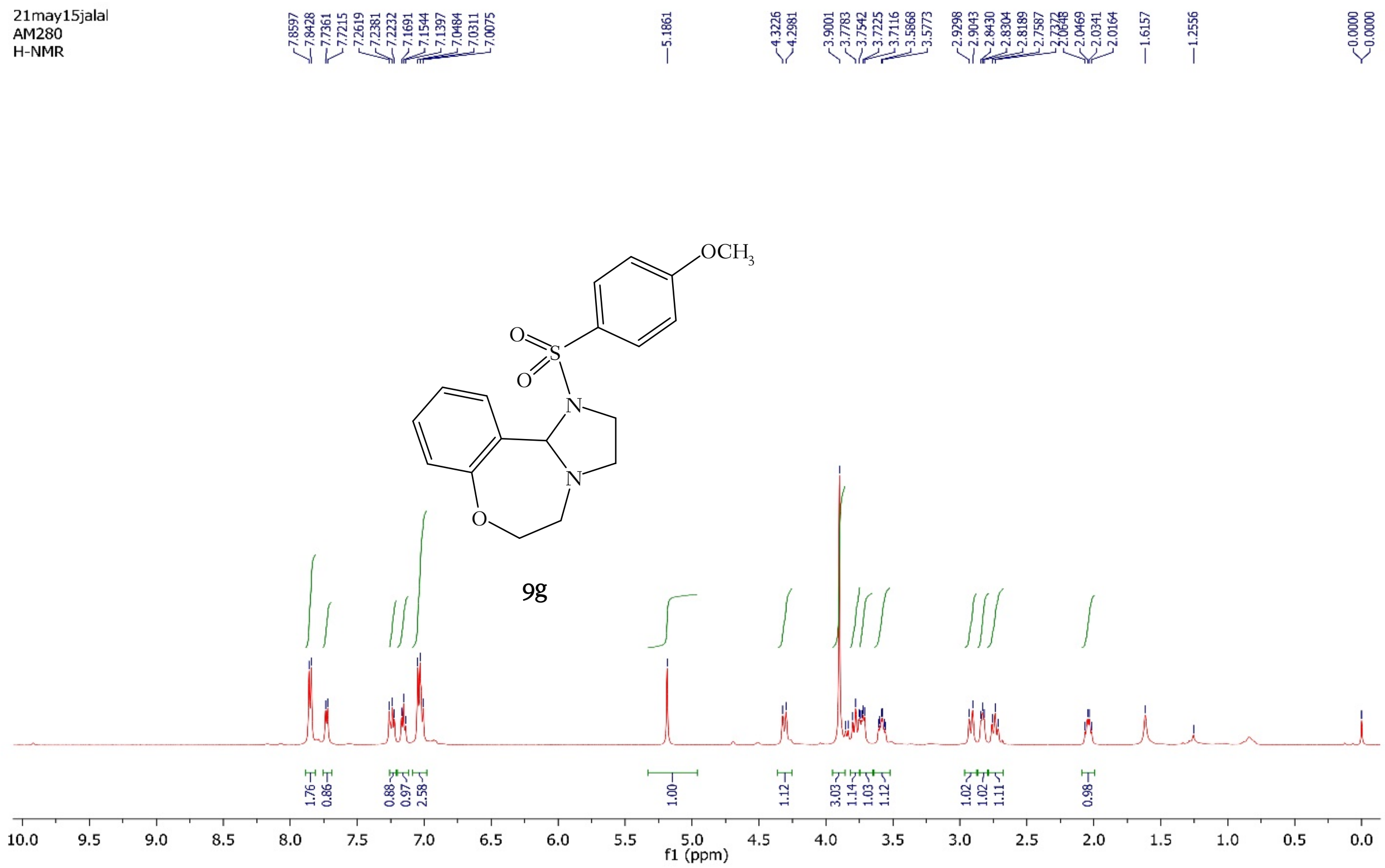

Fig.S19. $^{1}H$ NMR spectrum of compound 9g $CDCl_3$

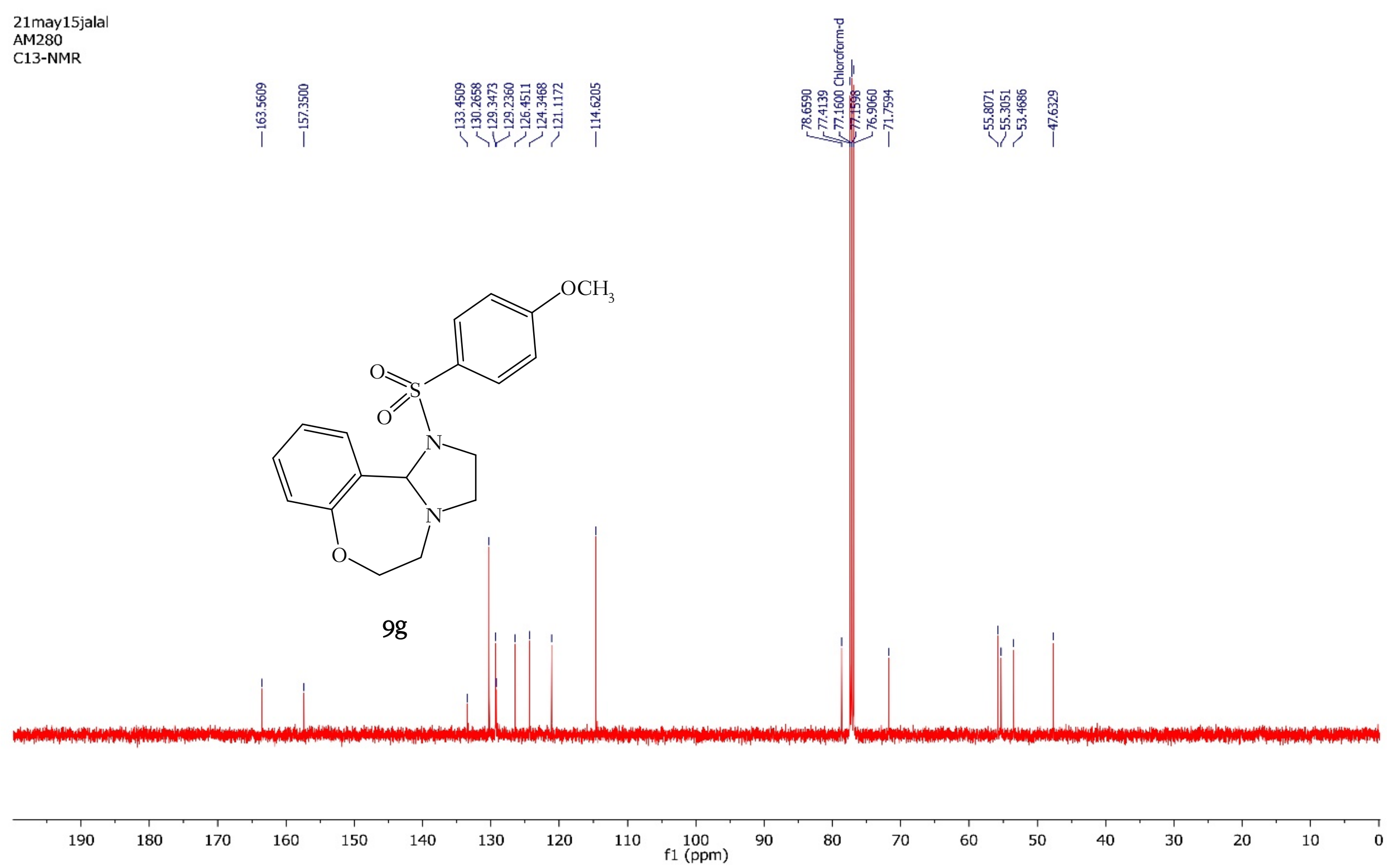

Fig.S20. $^{13}C$ NMR spectrum of compound 9g $CDCl_3$

# The University of Jordan
## Faculty of science
## Department of Chemistry

## Mass Spectrum Molecular Formula Report

**Analysis Info**

| | | | |
|---|---|---|---|
| Analysis Name | F:\Data\2022MAR08\Mustafa_000052.d | Acquisition Date | 3/13/2022 2:36:10 PM |
| Method | ESI_pos_20181024 | Operator | Bruker_PC |
| Sample Name | AM280 | Instrument | apex-IV |
| Comment | CHCl3+ACN | | |

**Acquisition Parameter**

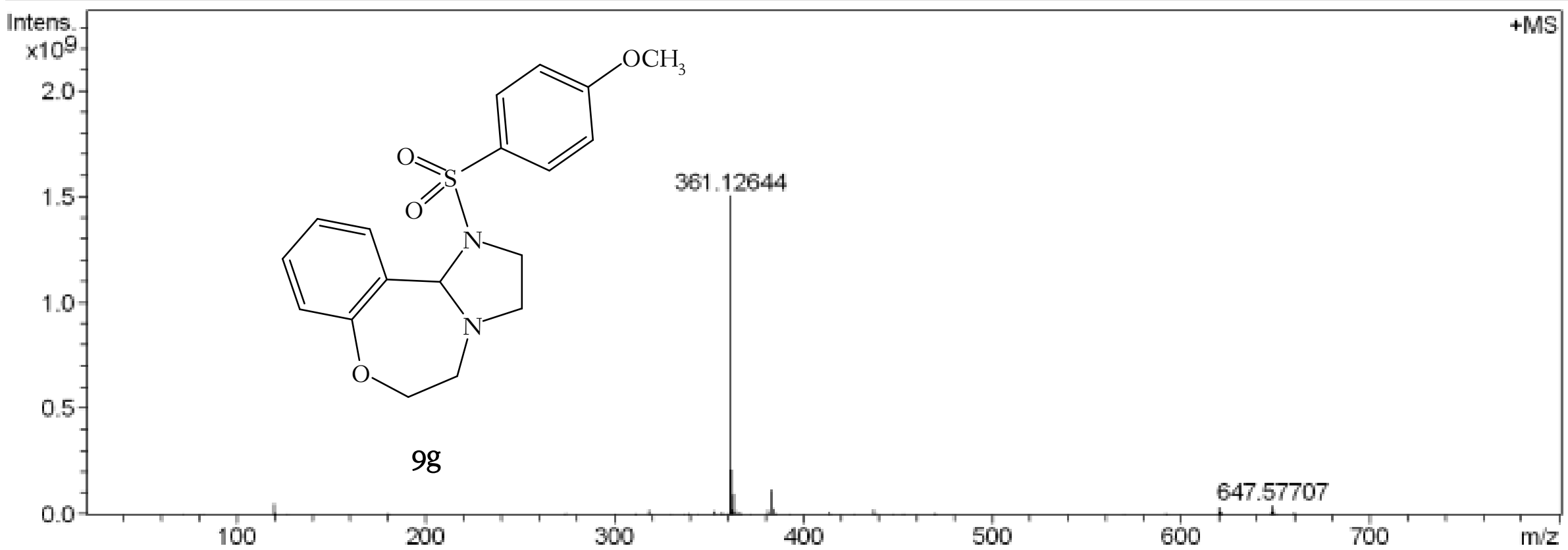


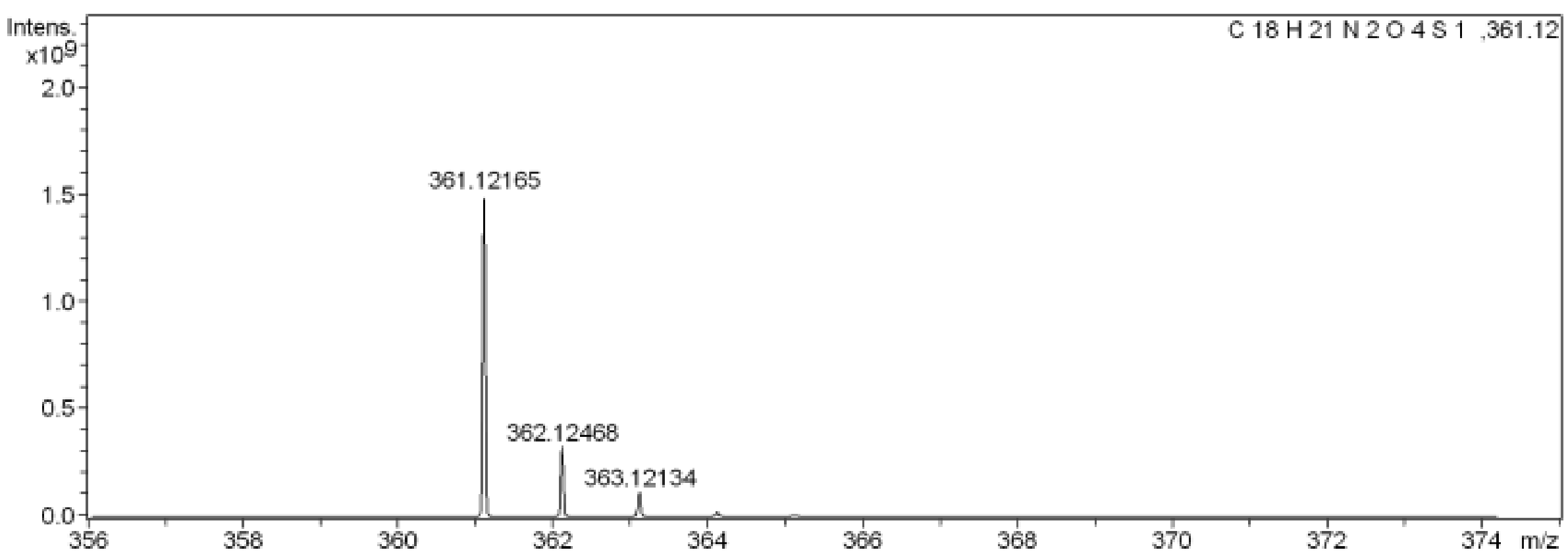


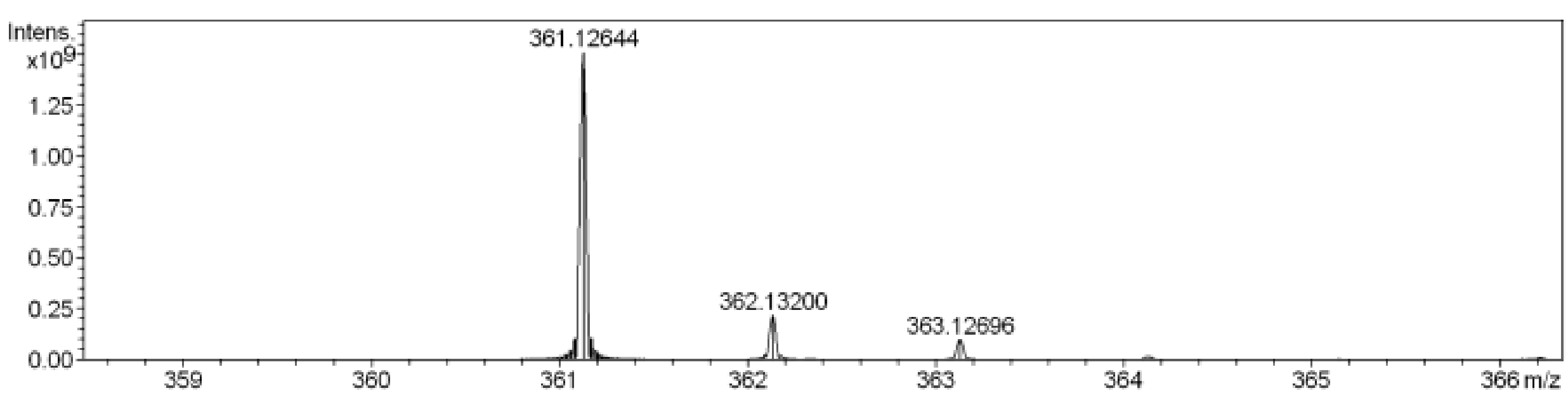


Fig.S21. HRMS spectrum of compound 9g

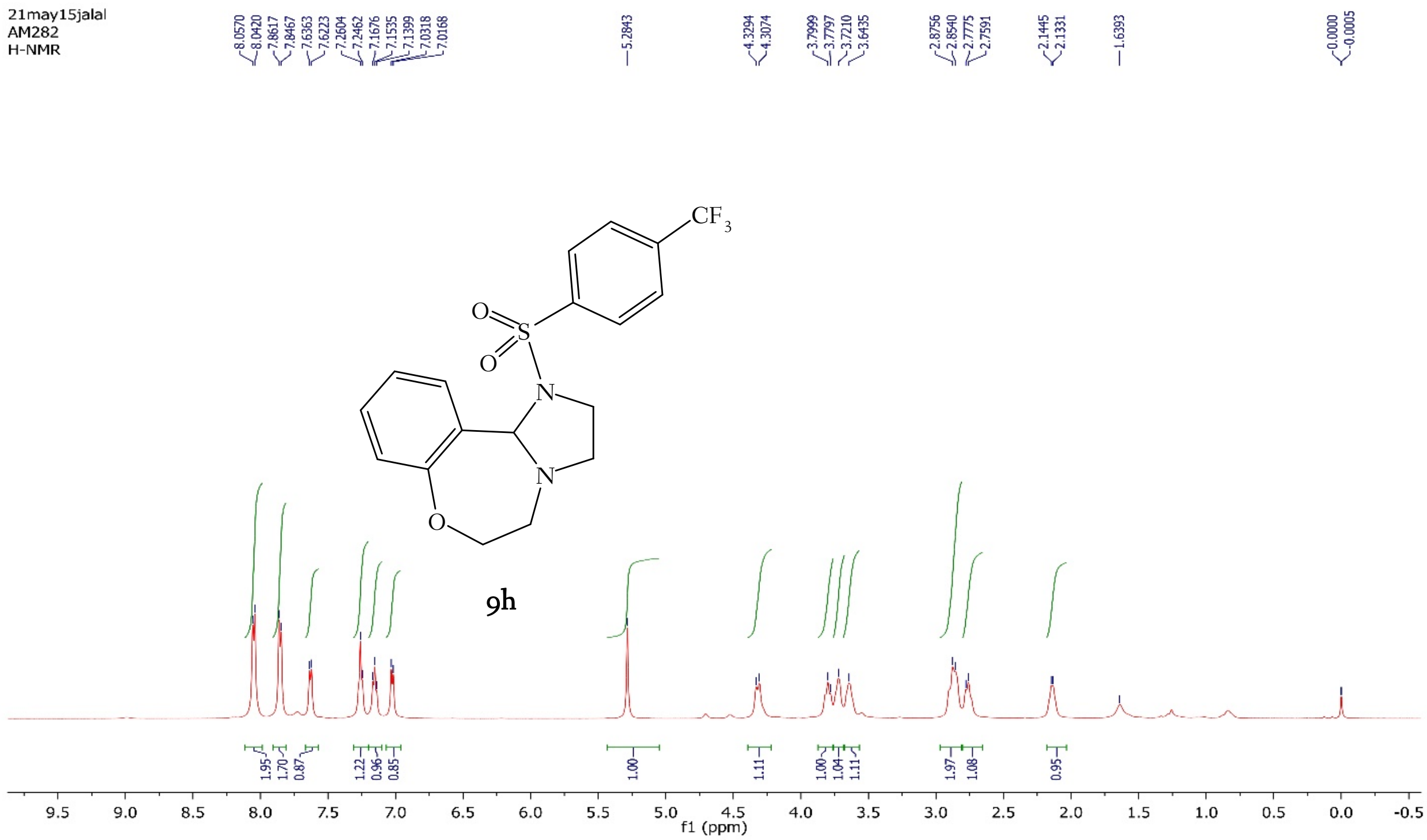


Fig.S22. $^{1}$H NMR spectrum of compound 9h $CDCl_3$

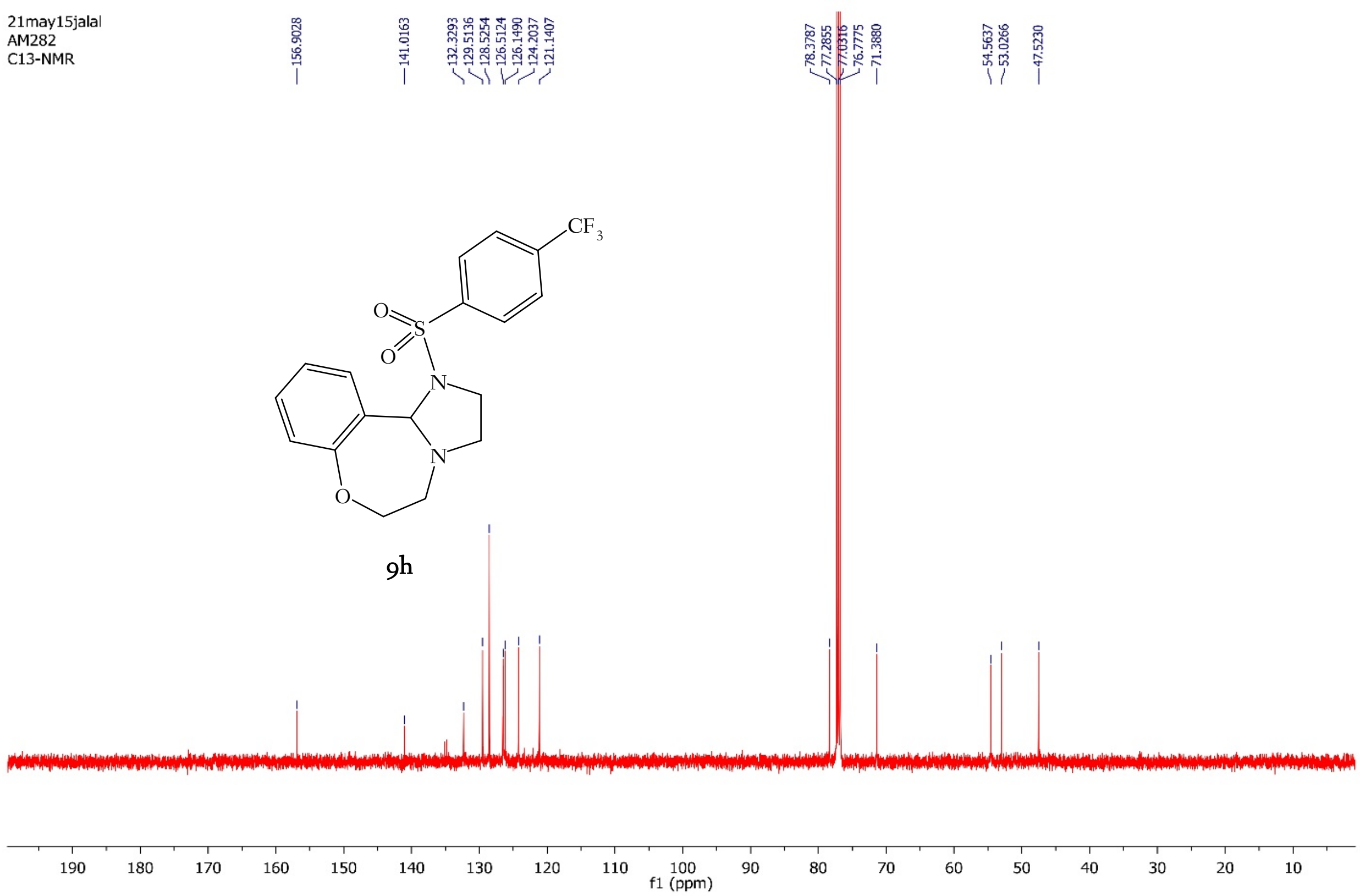


Fig.S23. $^{13}$C NMR spectrum of compound 9h $CDCl_3$

# The University of Jordan
## Faculty of science
## Department of Chemistry

# Mass Spectrum Molecular Formula Report

**Analysis Info**

| | | | |
|---|---|---|---|
| Analysis Name | F:\Data\2022MAR08\Mustafa_000056.d | Acquisition Date | 3/13/2022 2:45:30 PM |
| Method | ESI_pos_20181024 | Operator | Bruker_PC |
| Sample Name | AM282 | Instrument | apex-IV |
| Comment | CHCl3+ACN | | |

**Acquisition Parameter**

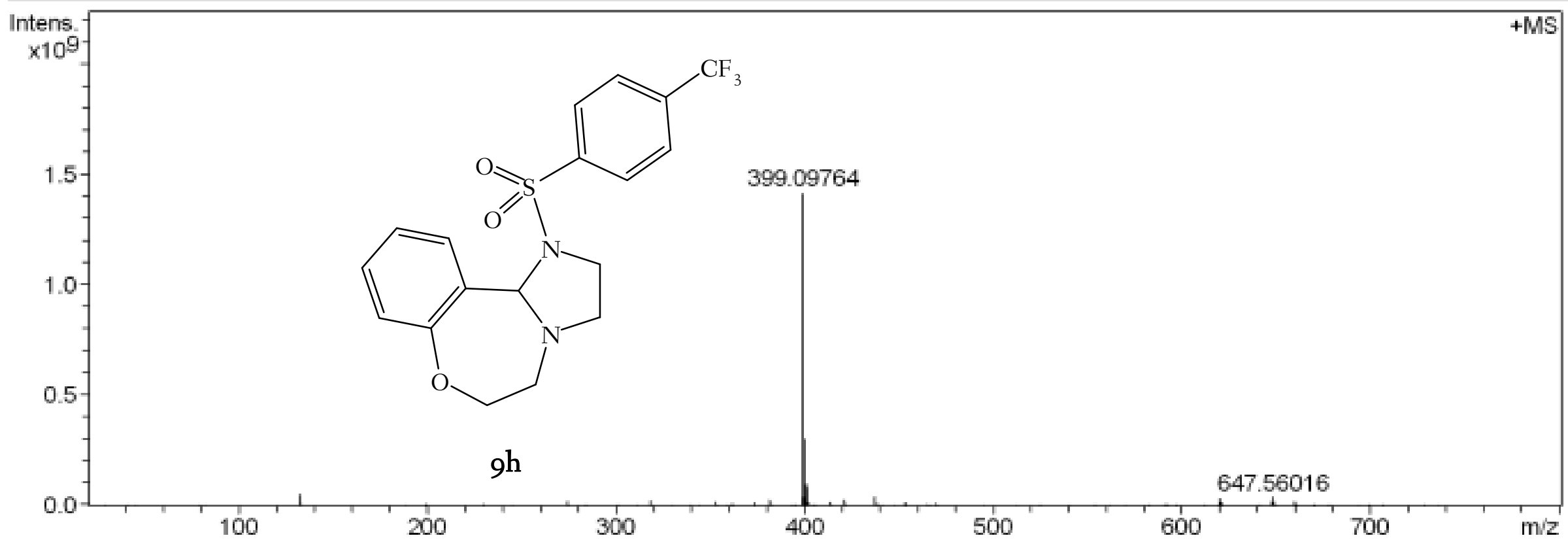


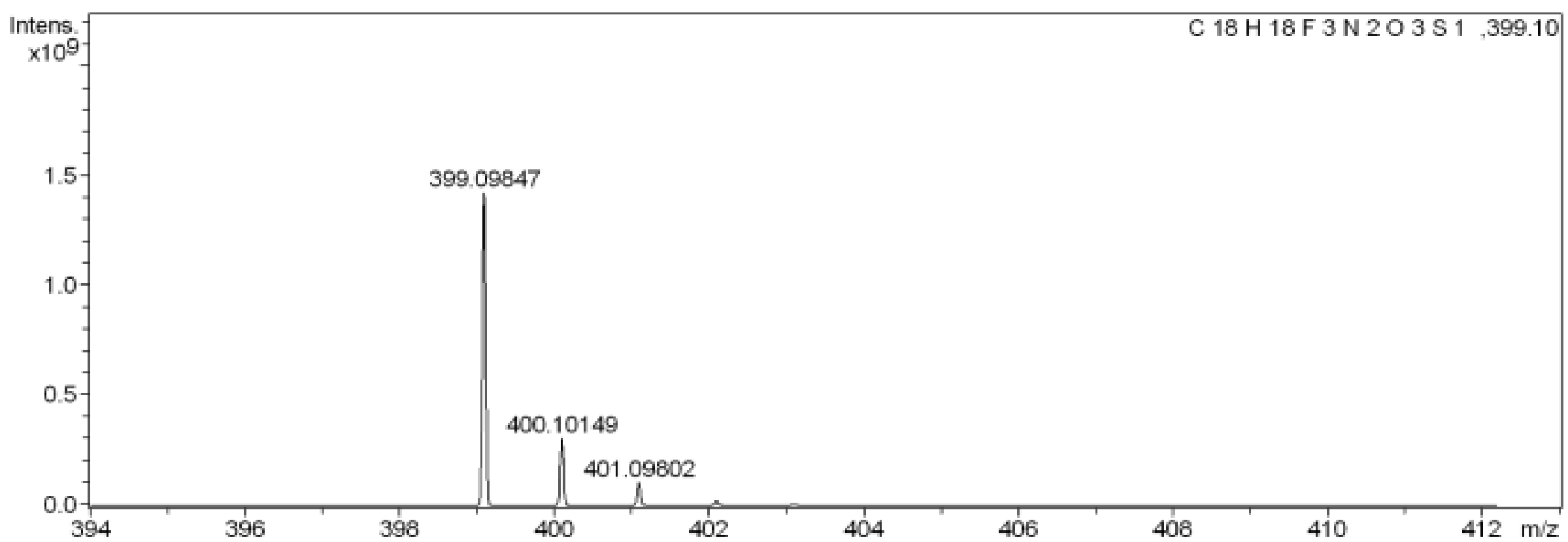


| Sum Formula | Sigma | m/z | Err [ppm] | Mean Err [ppm] | Err [mDa] | rdb | N Rule | e⁻ |
|---|---|---|---|---|---|---|---|---|
| C 18 H 18 F 3 N 2 O 3 S 1 | 0.002 | 399.09847 | 2.10 | 1.43 | 0.84 | 9.50 | ok | even |

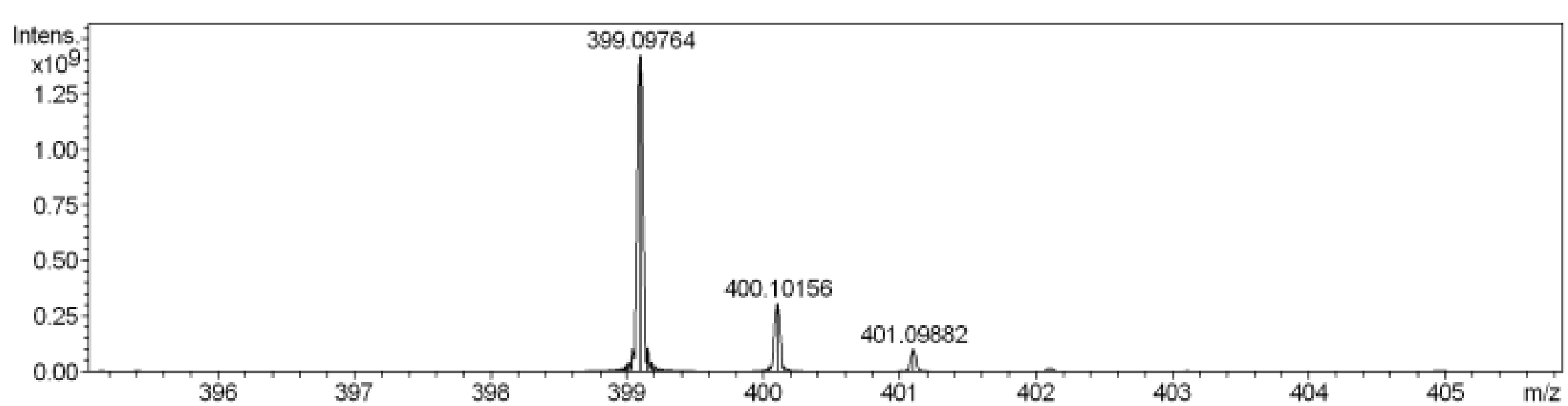


Fig.S24. HRMS spectrum of compound 9h

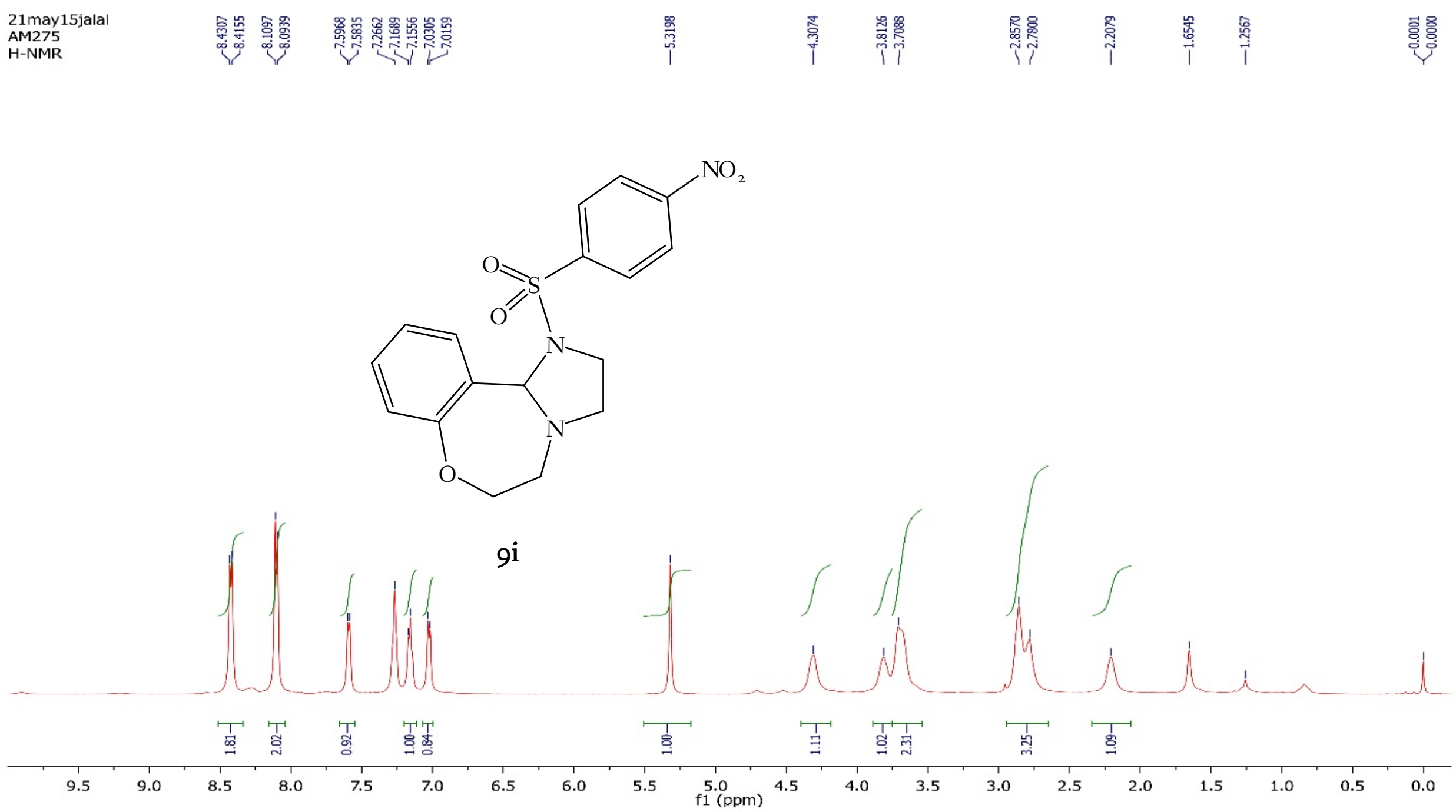


Fig.S25. $^{1}$H NMR spectrum of compound 9i $CDCl_3$

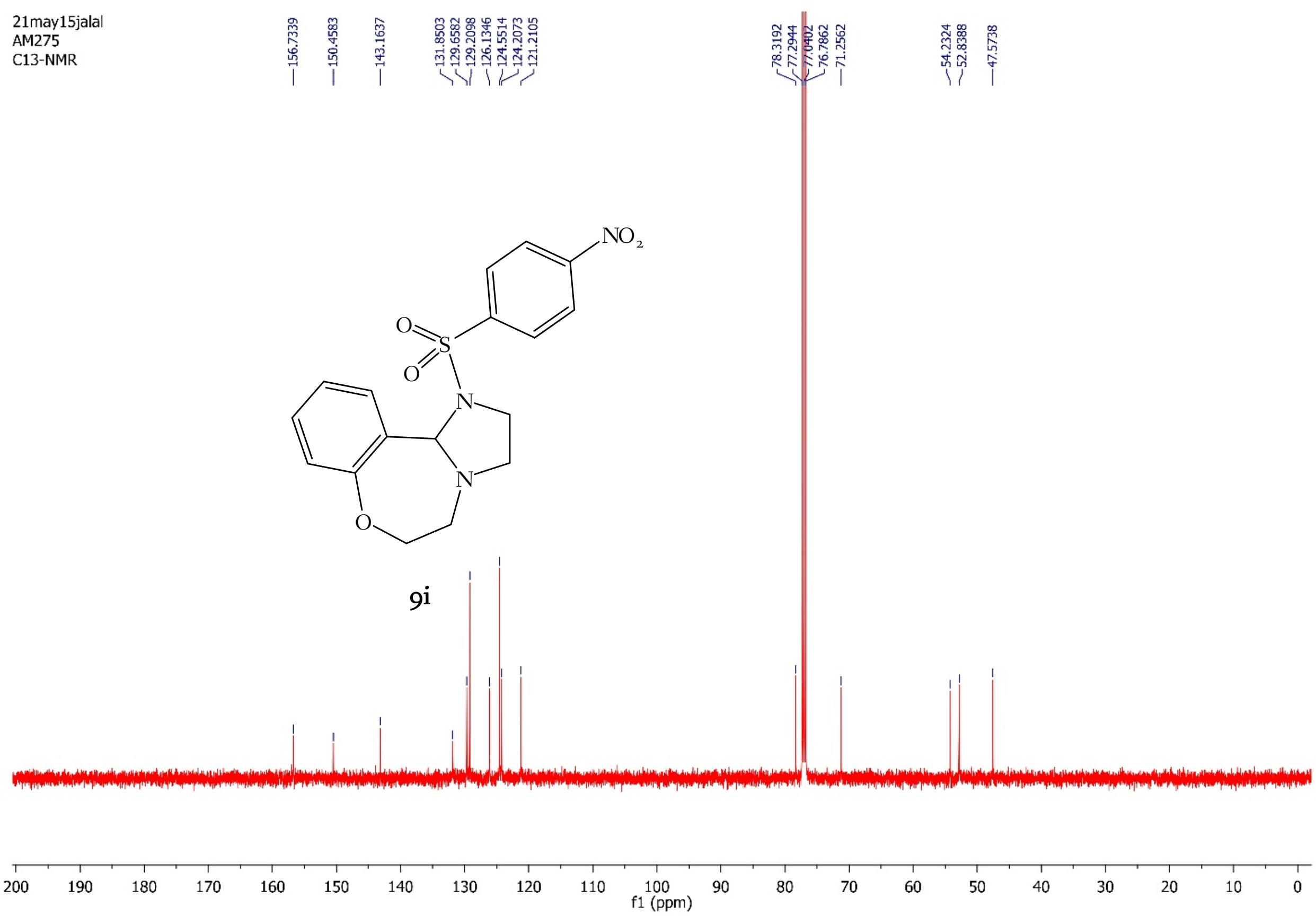


Fig.S26. $^{13}$C NMR spectrum of compound 9i $CDCl_3$

# The University of Jordan
## Faculty of science
## Department of Chemistry

## Mass Spectrum Molecular Formula Report

**Analysis Info**

| | | | |
|---|---|---|---|
| Analysis Name | F:\Data\2022MAR08\Mustafa_000046.d | Acquisition Date | 3/13/2022 2:08:45 PM |
| Method | ESI_pos_20181024 | Operator | Bruker_PC |
| Sample Name | AM275 | Instrument | apex-IV |
| Comment | CHCl3+ACN | | |

**Acquisition Parameter**

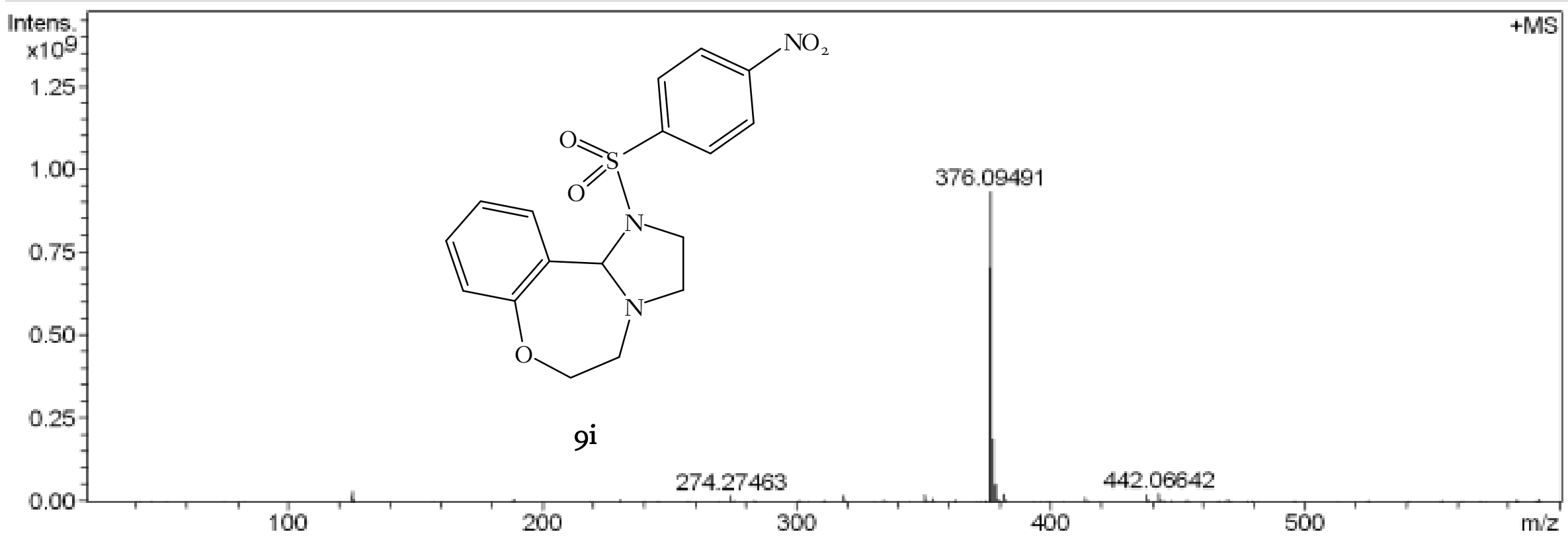


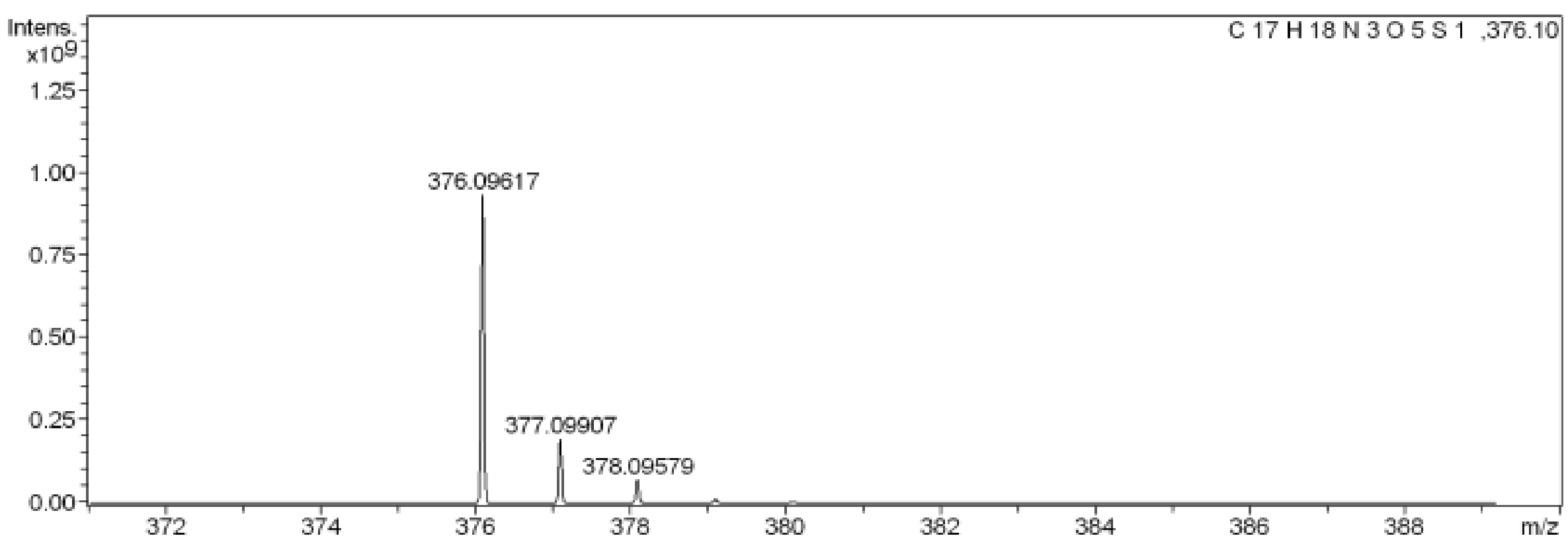


| Sum Formula | Sigma | m/z | Err [ppm] | Mean Err [ppm] | Err [mDa] | rdb | N Rule | e⁻ |
|---|---|---|---|---|---|---|---|---|
| C 17 H 18 N 3 O 5 S 1 | 0.009 | 376.09617 | 3.33 | 3.56 | 1.25 | 10.50 | ok | even |

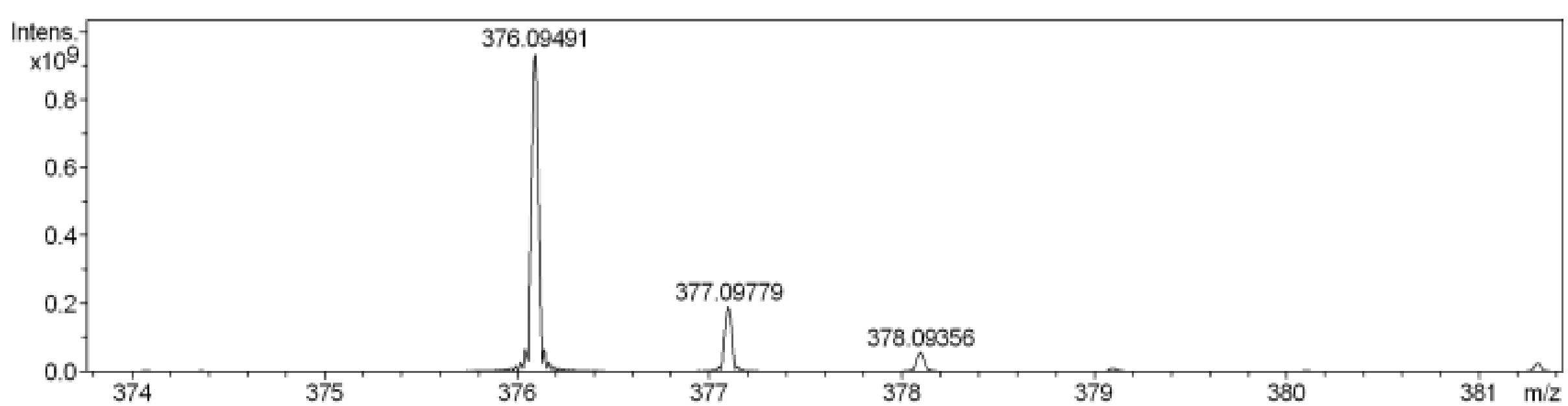


Fig.S27. HRMS spectrum of compound 9i

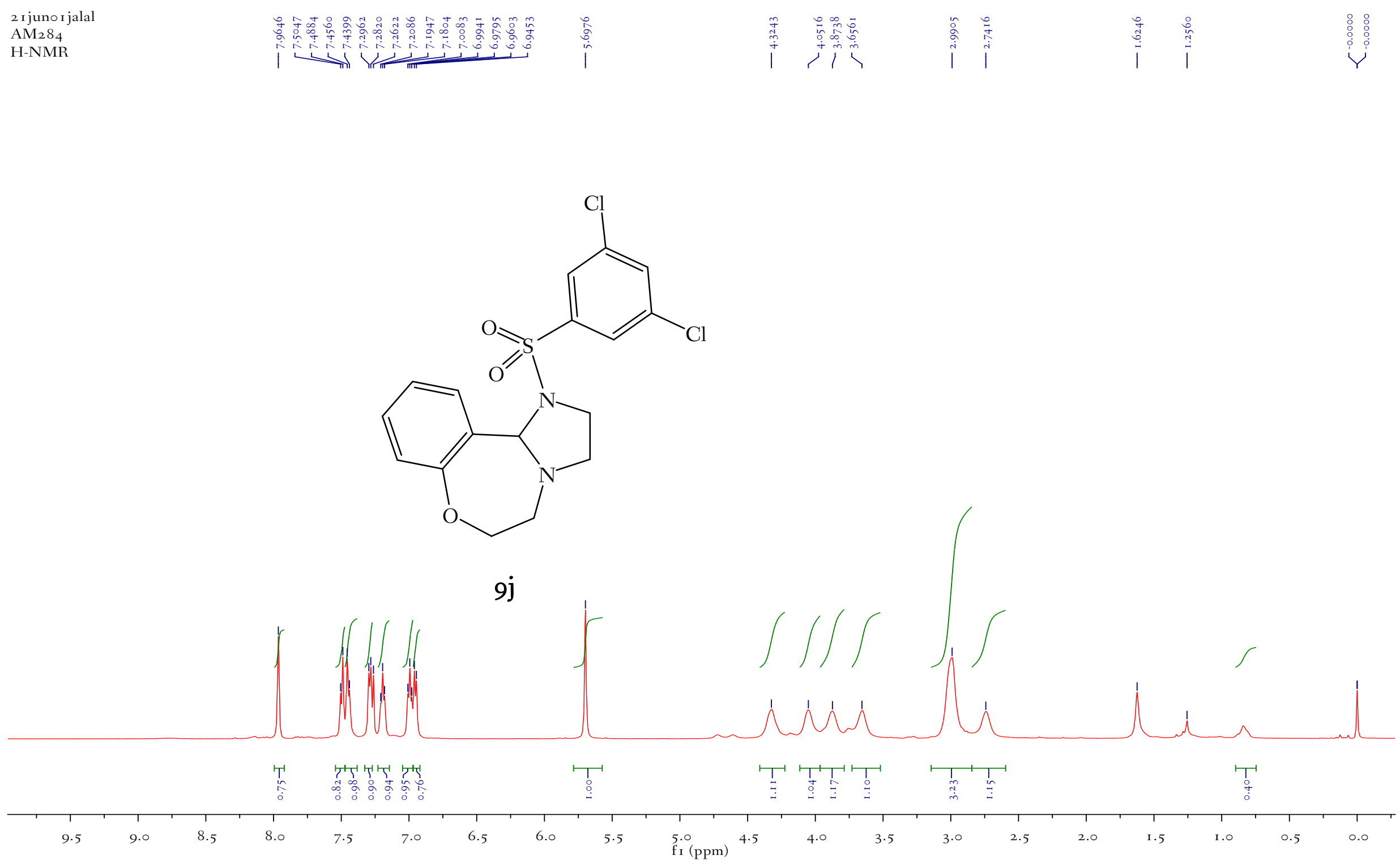


Fig.S28. $^{1}$H NMR spectrum of compound 9j $CDCl_3$

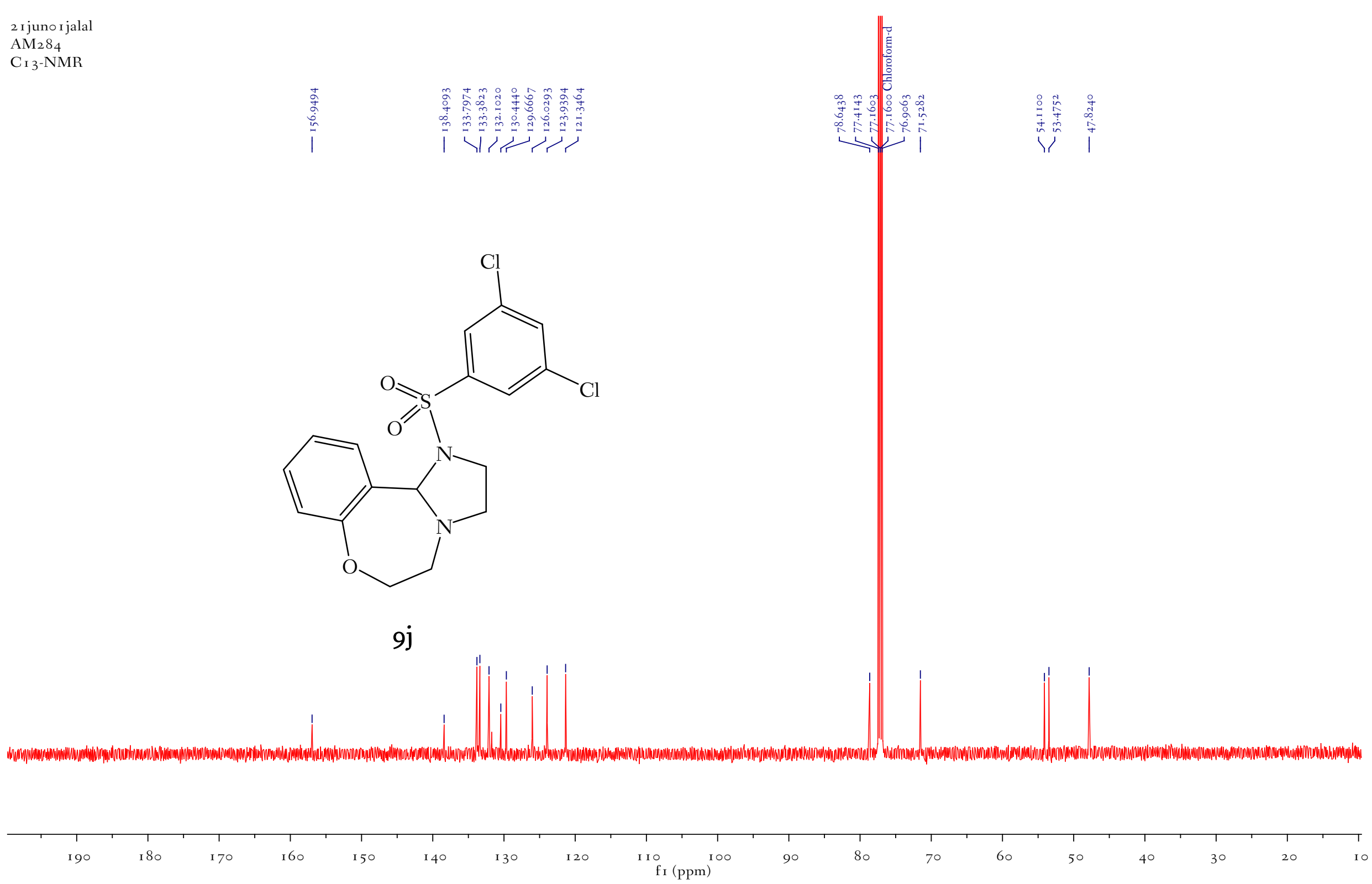


Fig.S29. $^{13}$C NMR spectrum of compound 9j $CDCl_3$

# The University of Jordan
## Faculty of science
## Department of Chemistry

## Mass Spectrum Molecular Formula Report

| | | | |
|---|---|---|---|
| **Analysis Info** | | Acquisition Date | 3/13/2022 10:19:13 AM |
| Analysis Name | F:\Data\2022MAR08\Mustafa_000027.d | | |
| Method | ESI_pos_20181024 | Operator | Bruker_PC |
| Sample Name | AM284 | Instrument | apex-IV |
| Comment | CHCl3+ACN | | |

**Acquisition Parameter**

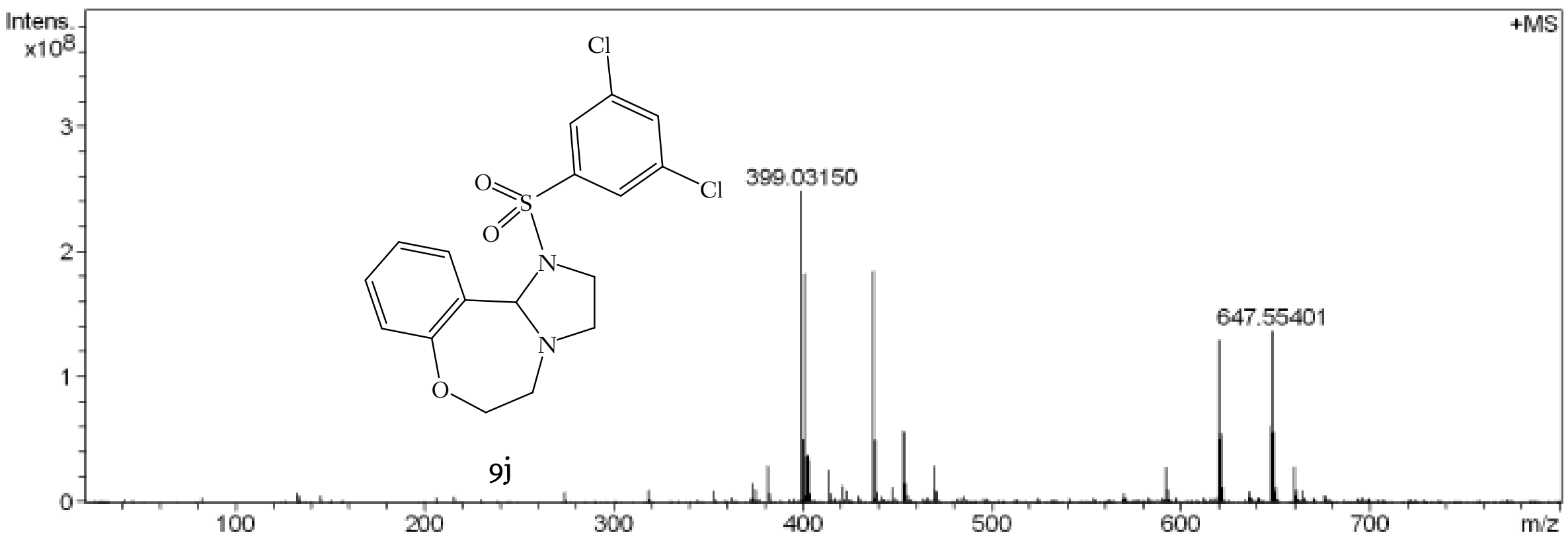


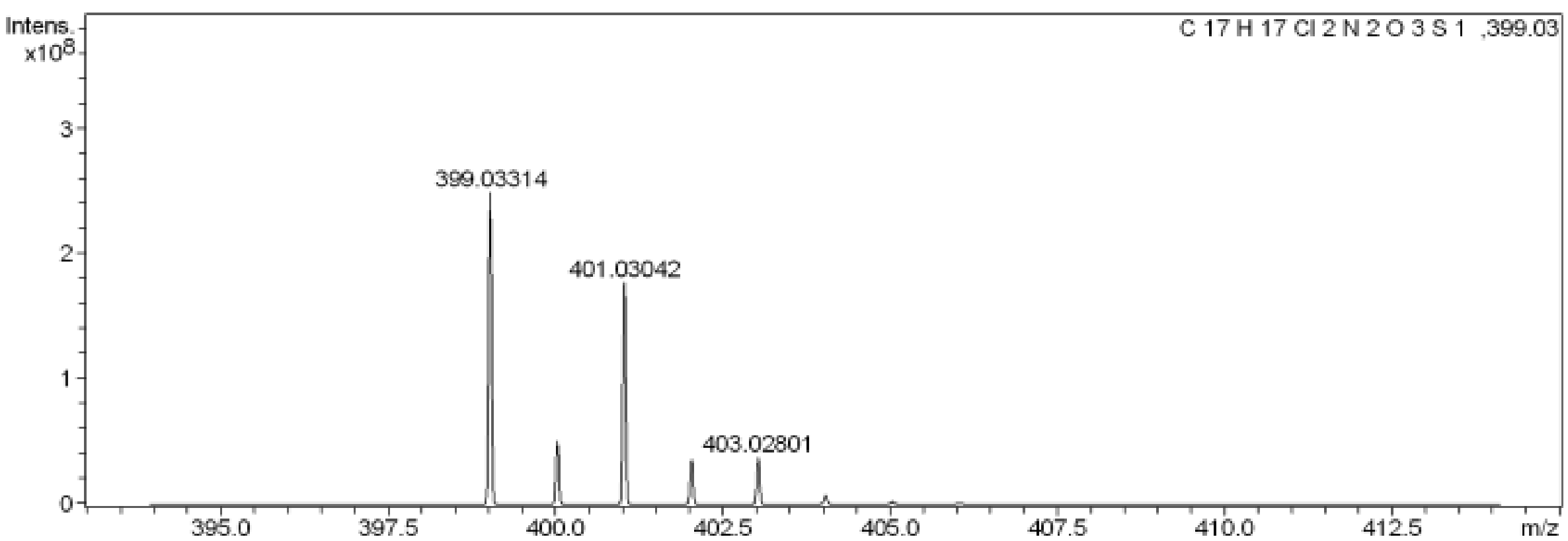


| Sum Formula | Sigma | m/z | Err [ppm] | Mean Err [ppm] | Err [mDa] | rdb | N Rule | e⁻ |
|---|---|---|---|---|---|---|---|---|
| C 17 H 17 Cl 2 N 2 O 3 S 1 | 0.012 | 399.03314 | 4.13 | 3.57 | 1.65 | 9.50 | ok | even |

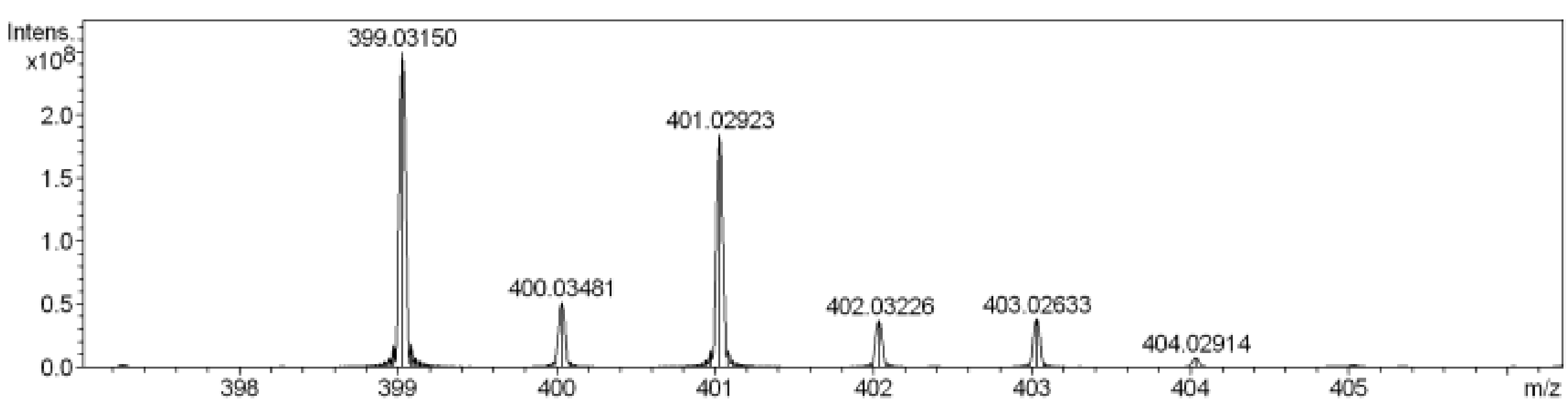


Fig.S30. HRMS spectrum of compound 9j

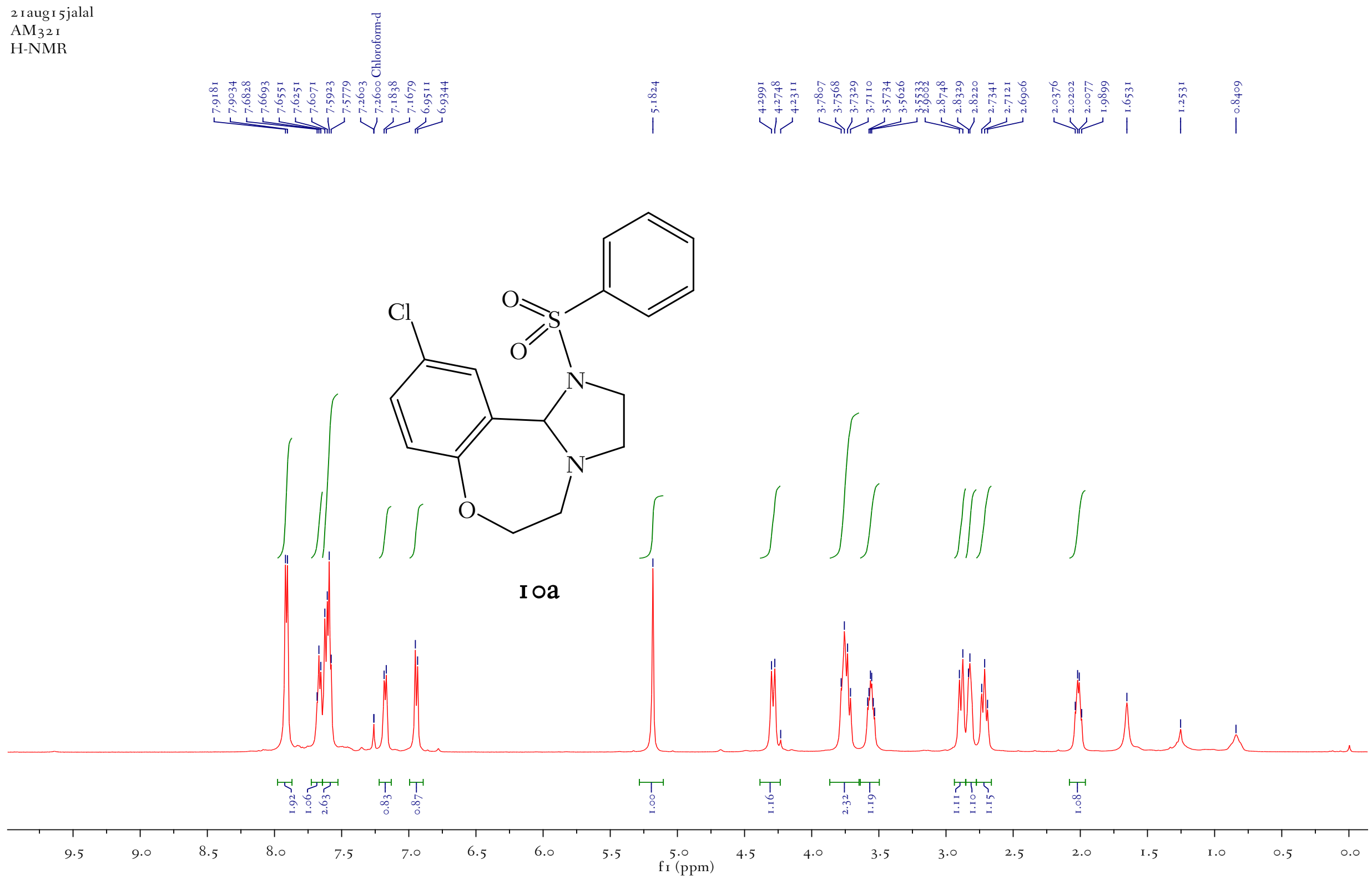


Fig.S31. $^{1}$H NMR spectrum of compound 10a $CDCl_3$

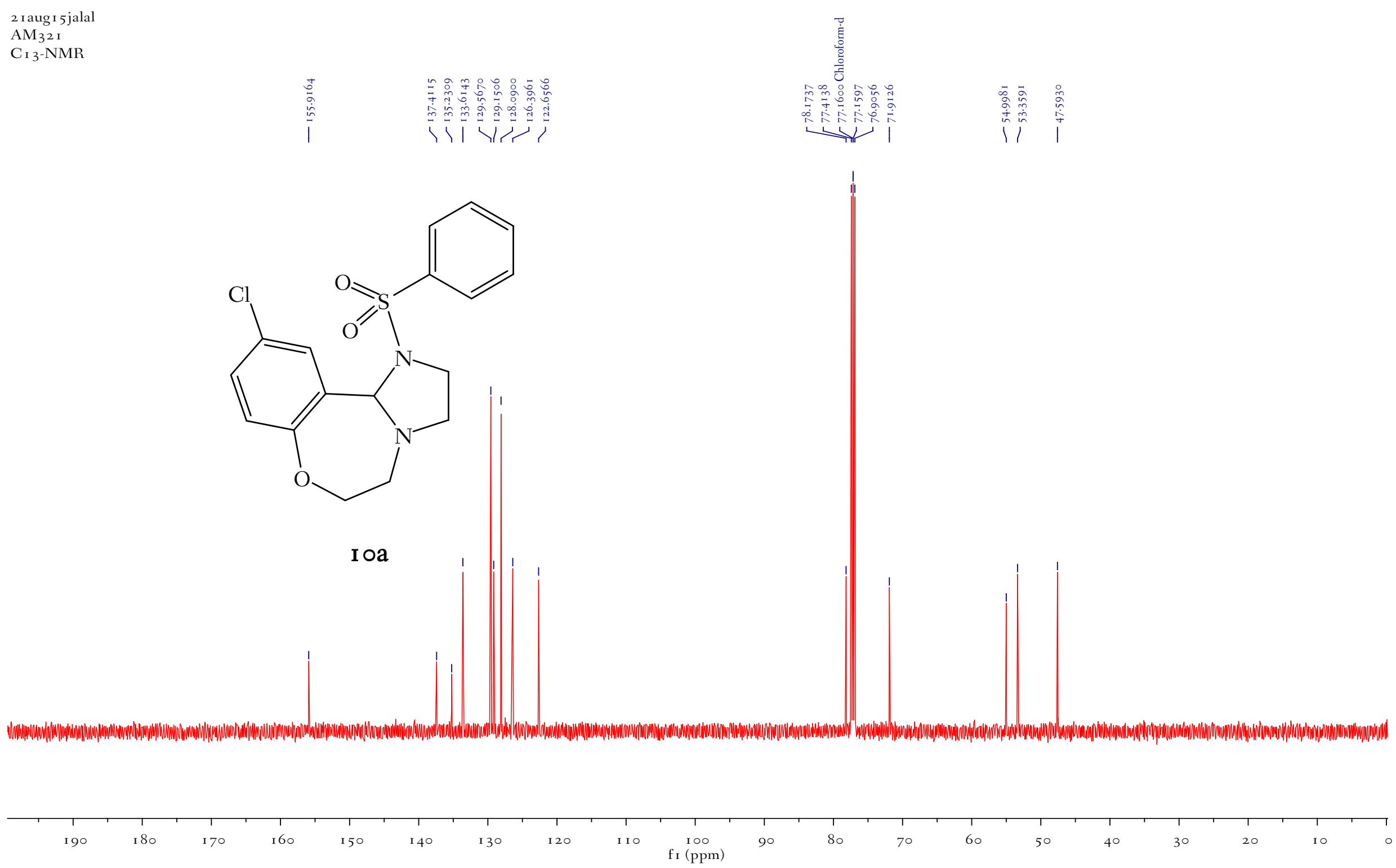


Fig.S32. $^{13}$C NMR spectrum of compound 10a $CDCl_3$

# The University of Jordan
## Faculty of science
## Department of Chemistry

## Mass Spectrum Molecular Formula Report

**Analysis Info**

| | | | |
|---|---|---|---|
| Analysis Name | F:\Data\2022MAR20\Mustafa_000049.d | Acquisition Date | 3/23/2022 2:02:05 PM |
| Method | ESI_pos_20181024 | Operator | Bruker_PC |
| Sample Name | AM321 | Instrument | apex-IV |
| Comment | CHCl3+ACN | | |

**Acquisition Parameter**

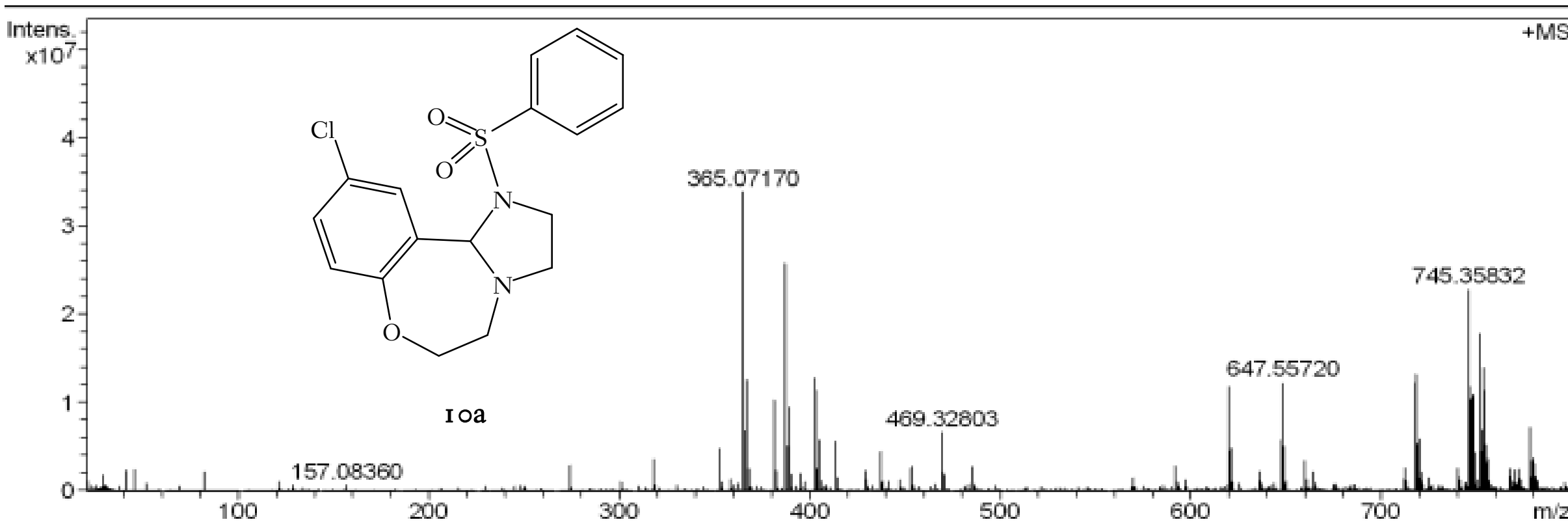


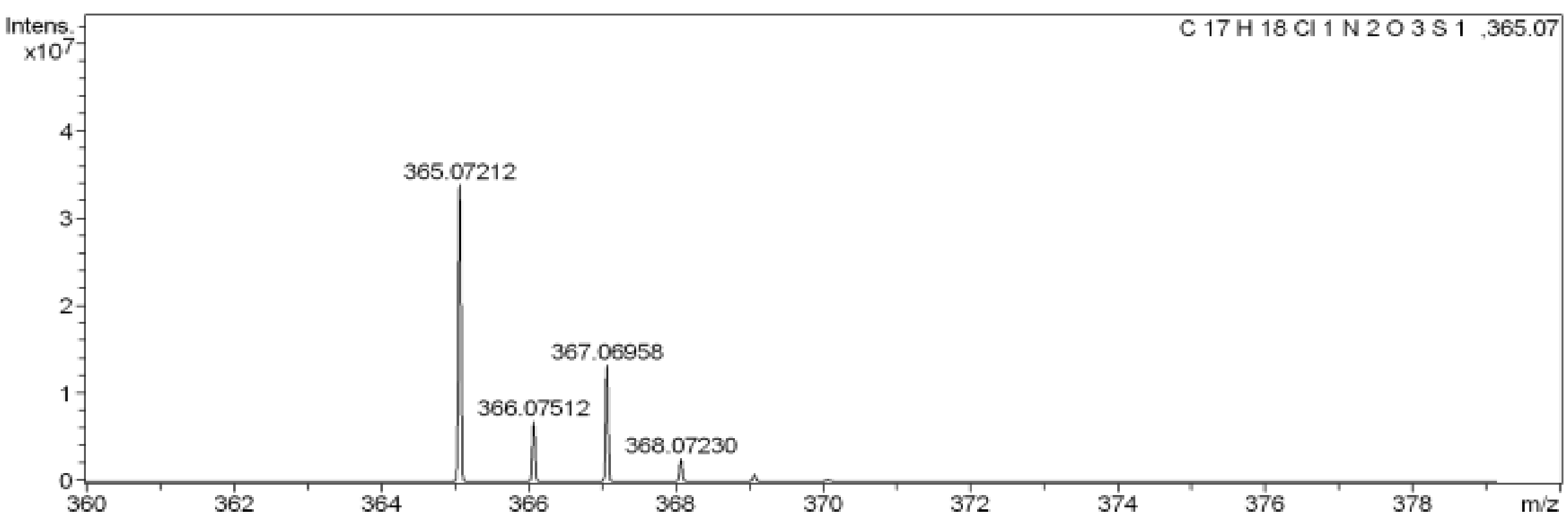


| Sum Formula | Sigma | m/z | Err [ppm] | Mean Err [ppm] | Err [mDa] | rdb | N Rule | $e^-$ |
|---|---|---|---|---|---|---|---|---|
| C 17 H 18 Cl 1 N 2 O 3 S 1 | 0.009 | 365.07212 | 1.14 | 1.36 | 0.42 | 9.50 | ok | even |

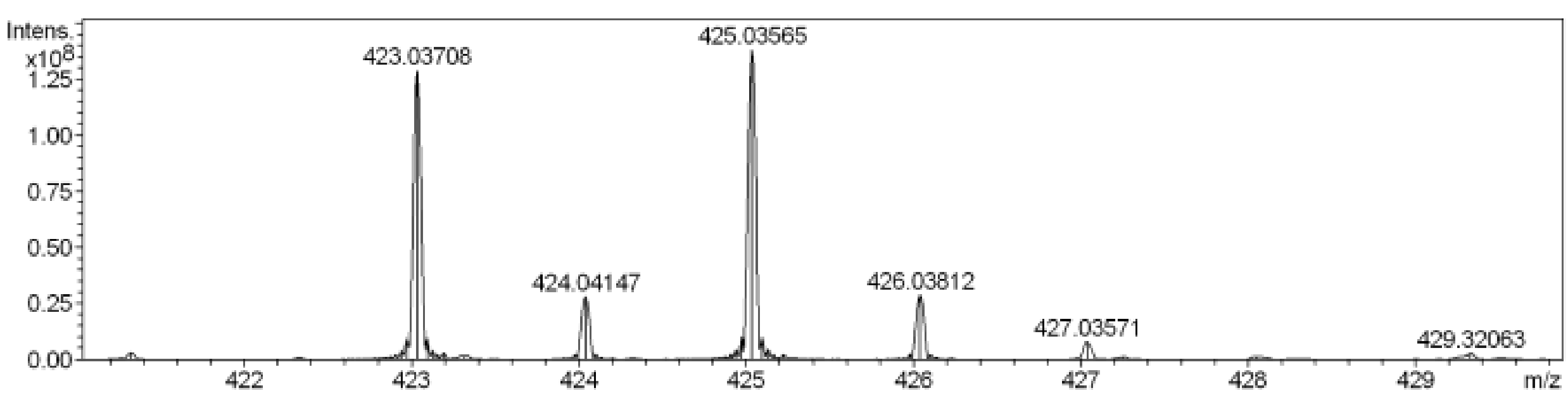


Fig.S33. HRMS spectrum of compound 10a

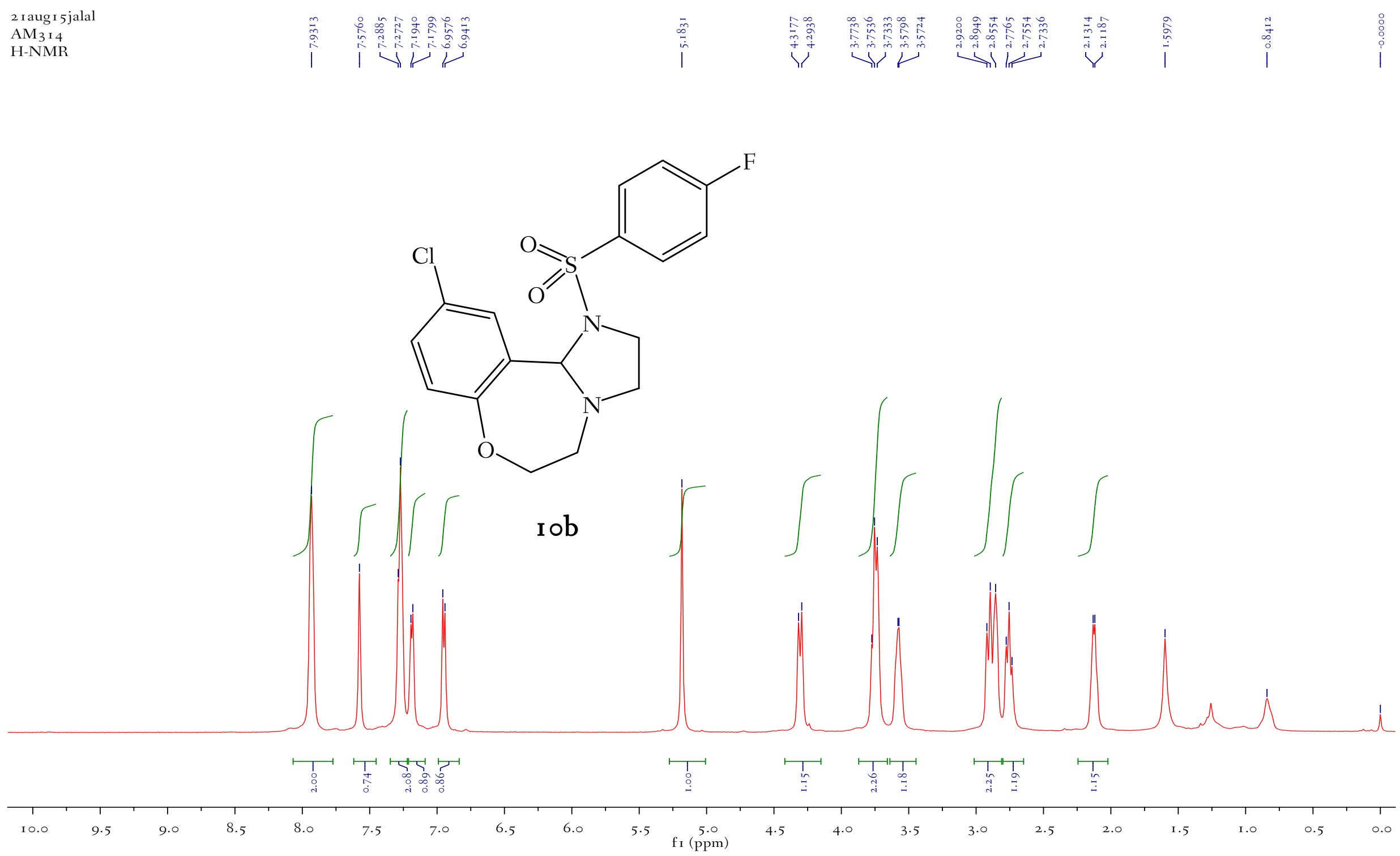

Fig.S34. $^{1}$H NMR spectrum of compound 10b $CDCl_3$

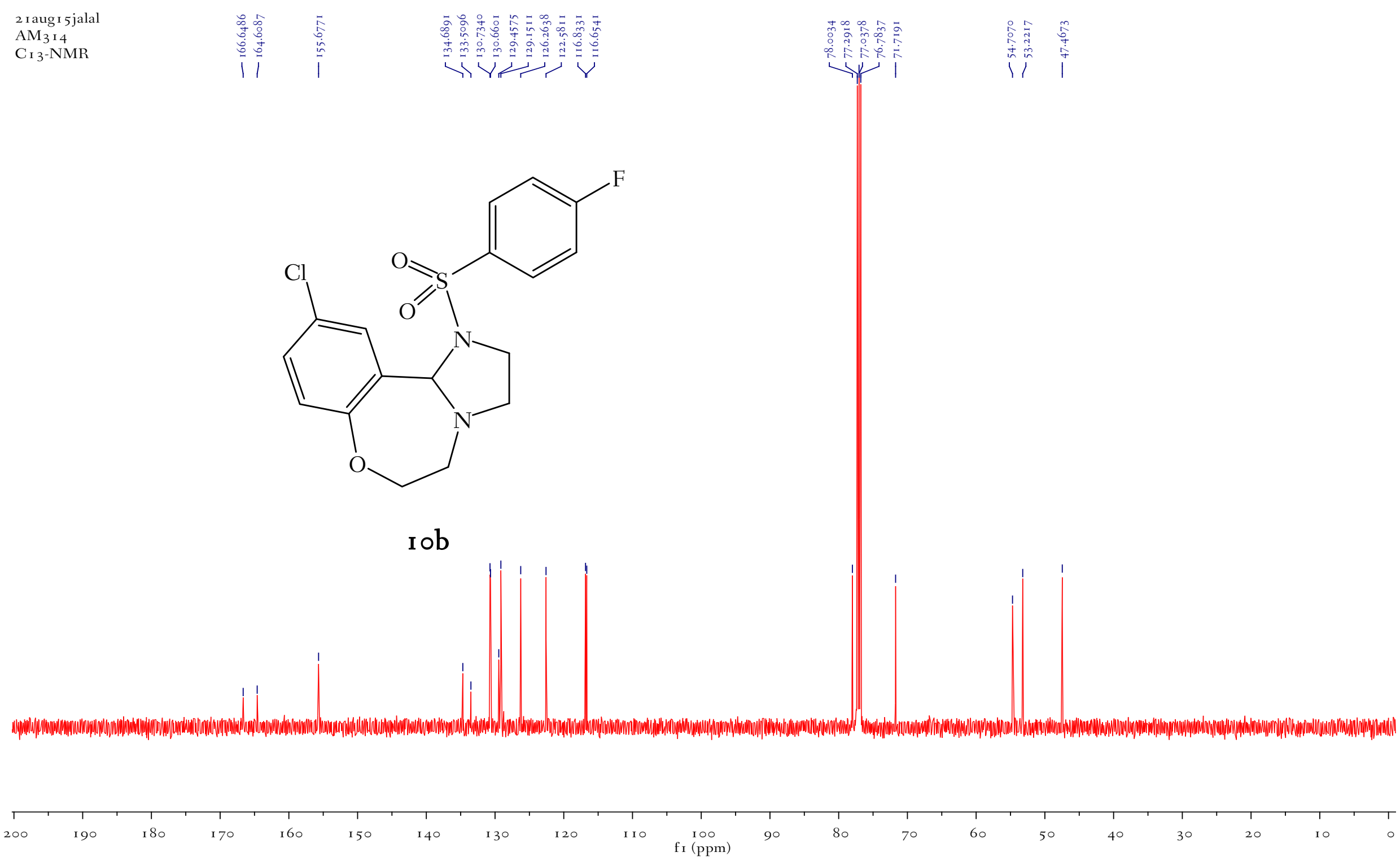

Fig.S35. $^{13}$C NMR spectrum of compound 10b $CDCl_3$

# The University of Jordan
## Faculty of science
## Department of Chemistry

## Mass Spectrum Molecular Formula Report

**Analysis Info**

| | | | |
|---|---|---|---|
| Analysis Name | F:\Data\2022MAR14\Mustafa_000013.d | Acquisition Date | 3/14/2022 1:31:50 PM |
| Method | ESI_pos_20181024 | Operator | Bruker_PC |
| Sample Name | AM314 | Instrument | apex-IV |
| Comment | CHCl3+ACN | | |

**Acquisition Parameter**

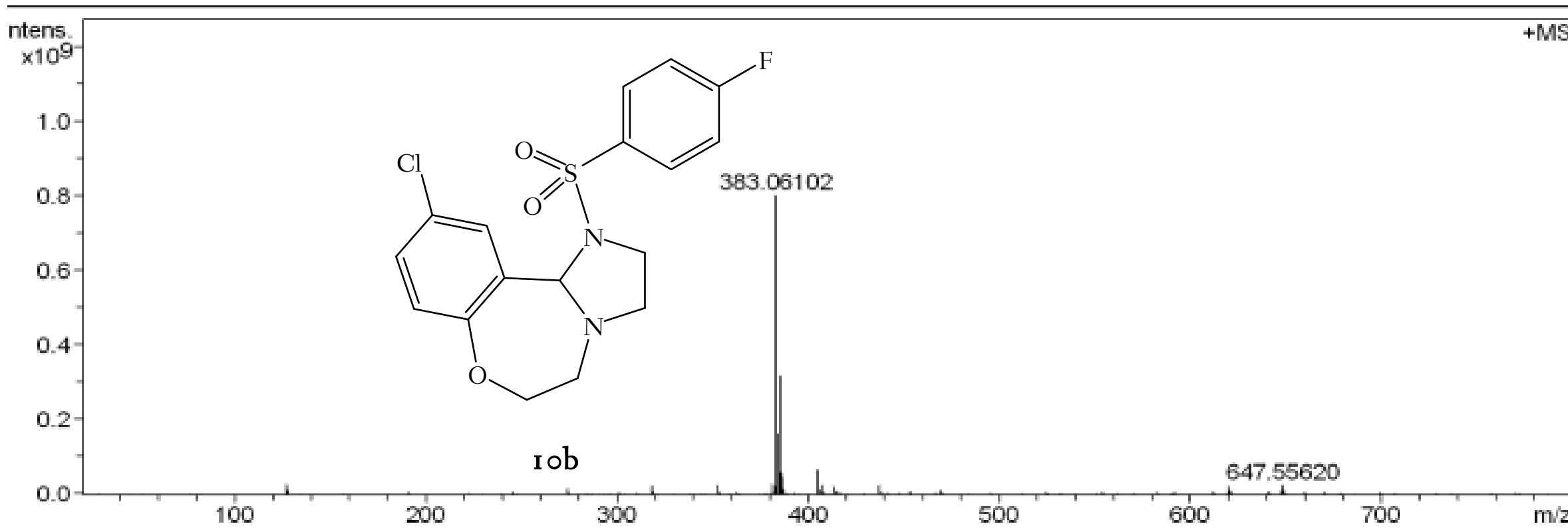


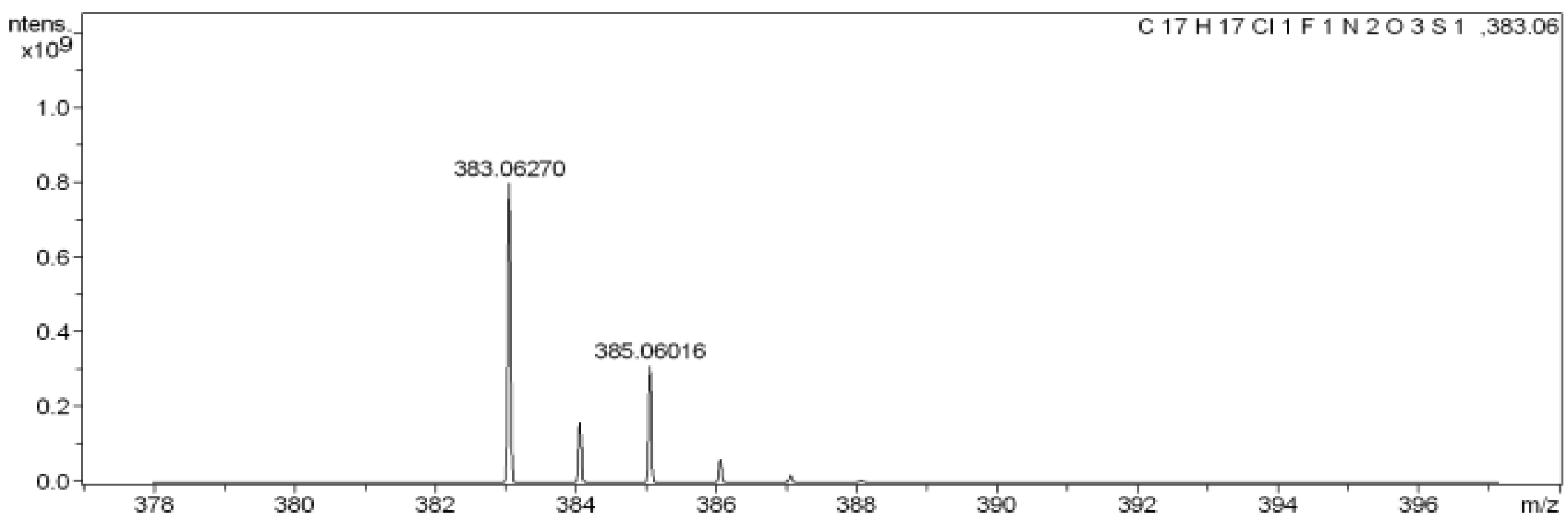


| Sum Formula | Sigma | m/z | Err [ppm] | Mean Err [ppm] | Err [mDa] | rdb | N Rule | e⁻ |
|---|---|---|---|---|---|---|---|---|
| C 17 H 17 Cl 1 F 1 N 2 O 3 S 1 | 0.004 | 383.06270 | 4.37 | 3.33 | 1.67 | 9.50 | ok | even |

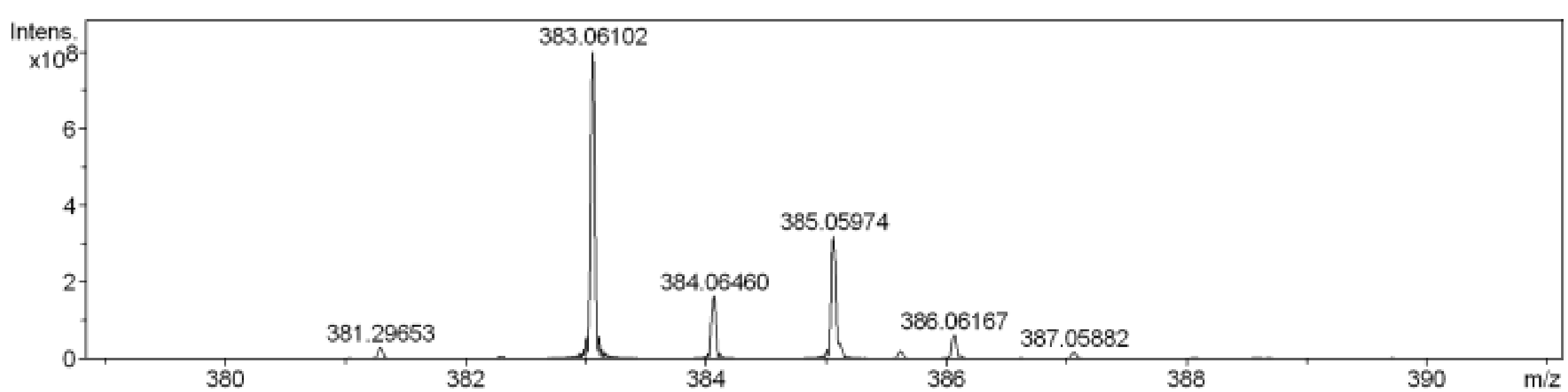


Fig.S36. HRMS spectrum of compound 10b

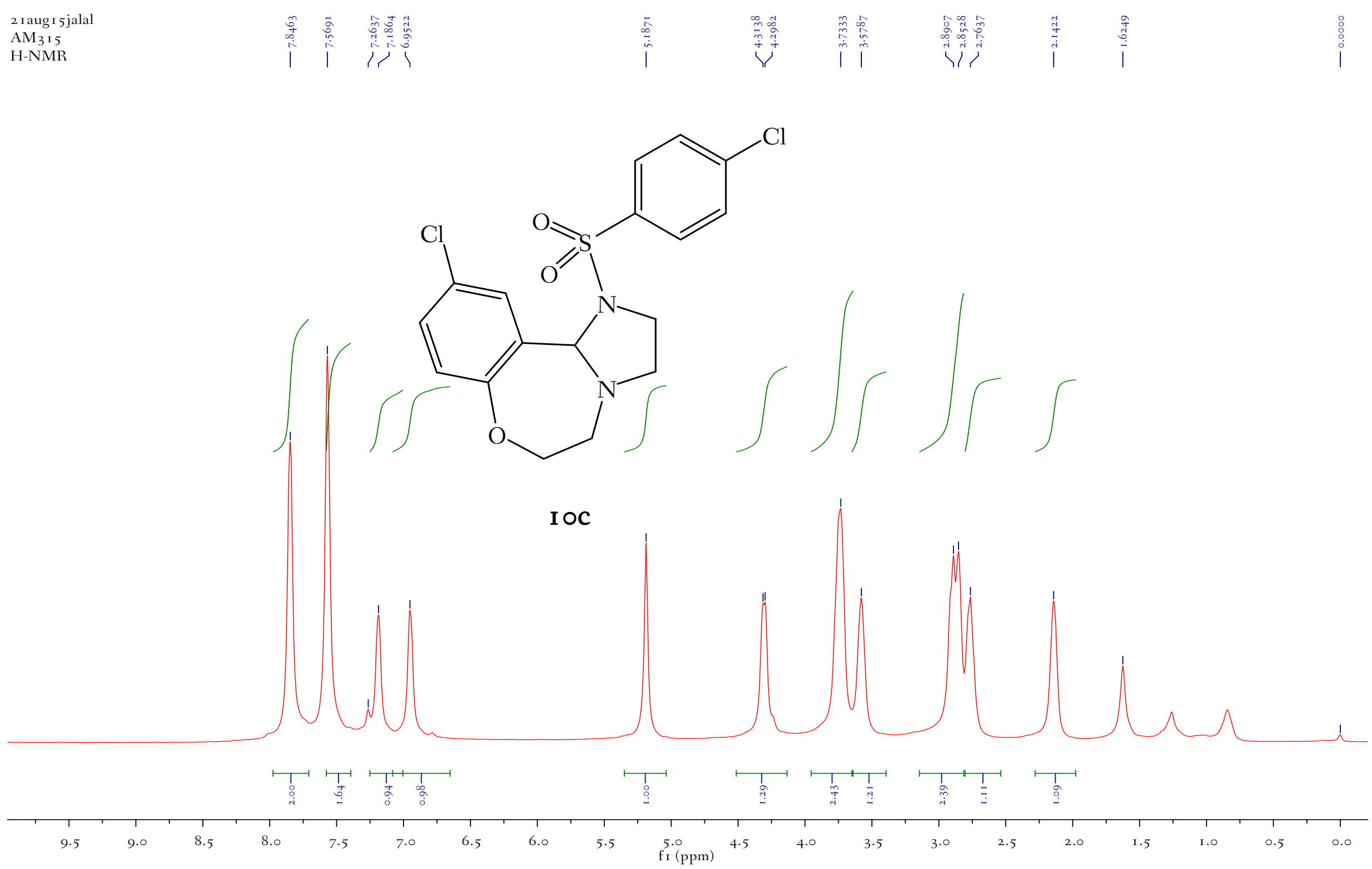


Fig.S37. $^{1}$H NMR spectrum of compound 10c $CDCl_3$

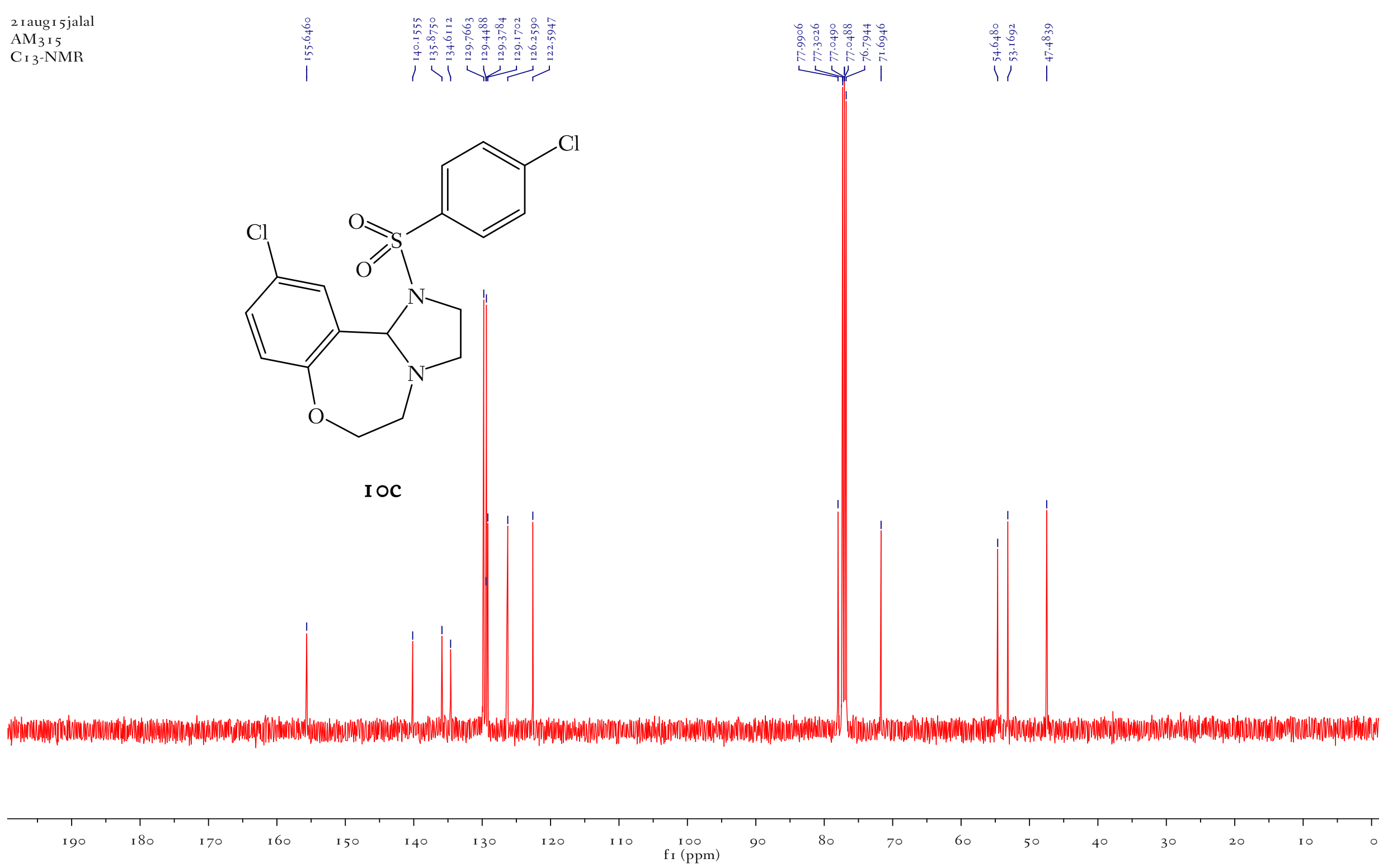

Fig.S38. $^{13}C$ NMR spectrum of compound 10c $CDCl_3$

**The University of Jordan**

**Faculty of science**

**Department of Chemistry**

## Mass Spectrum Molecular Formula Report

| **Analysis Info** | | Acquisition Date | 3/14/2022 1:41:31 PM |
|---|---|---|---|
| Analysis Name | F:\Data\2022MAR14\Mustafa_000015.d | | |
| Method | ESI_pos_20181024 | Operator | Bruker_PC |
| Sample Name | AM315 | Instrument | apex-IV |
| Comment | CHCl3+ACN | | |

**Acquisition Parameter**

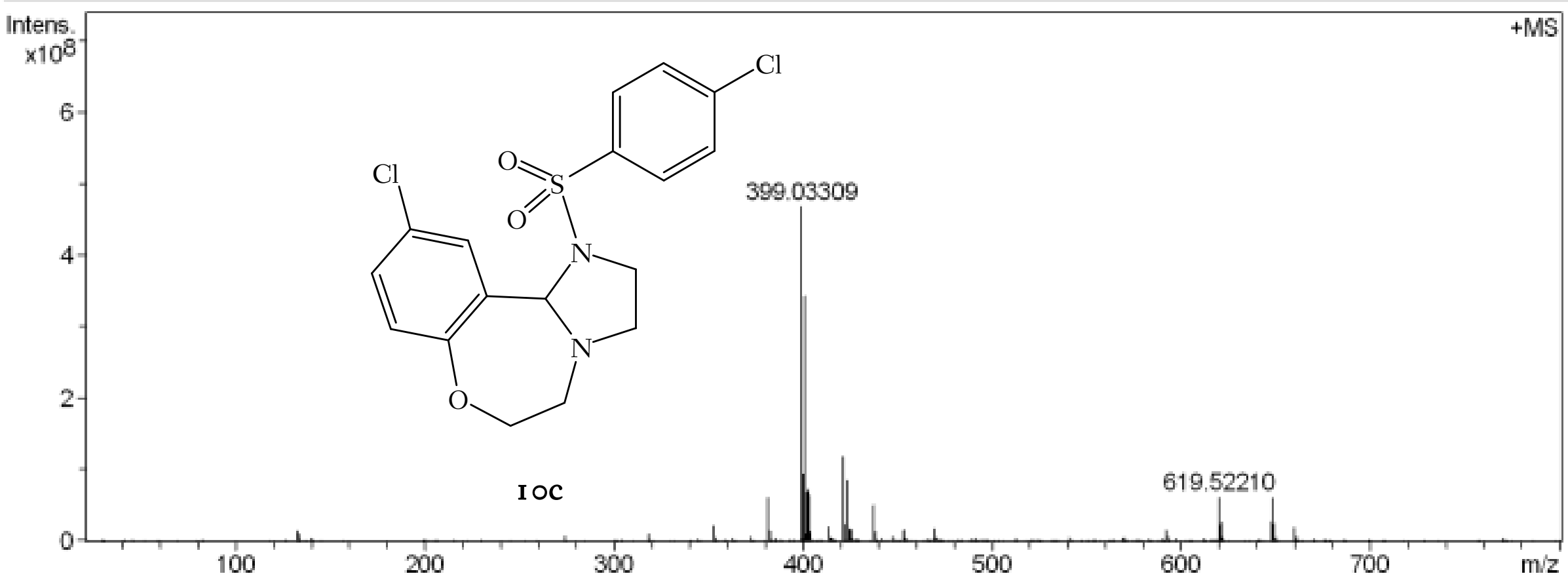


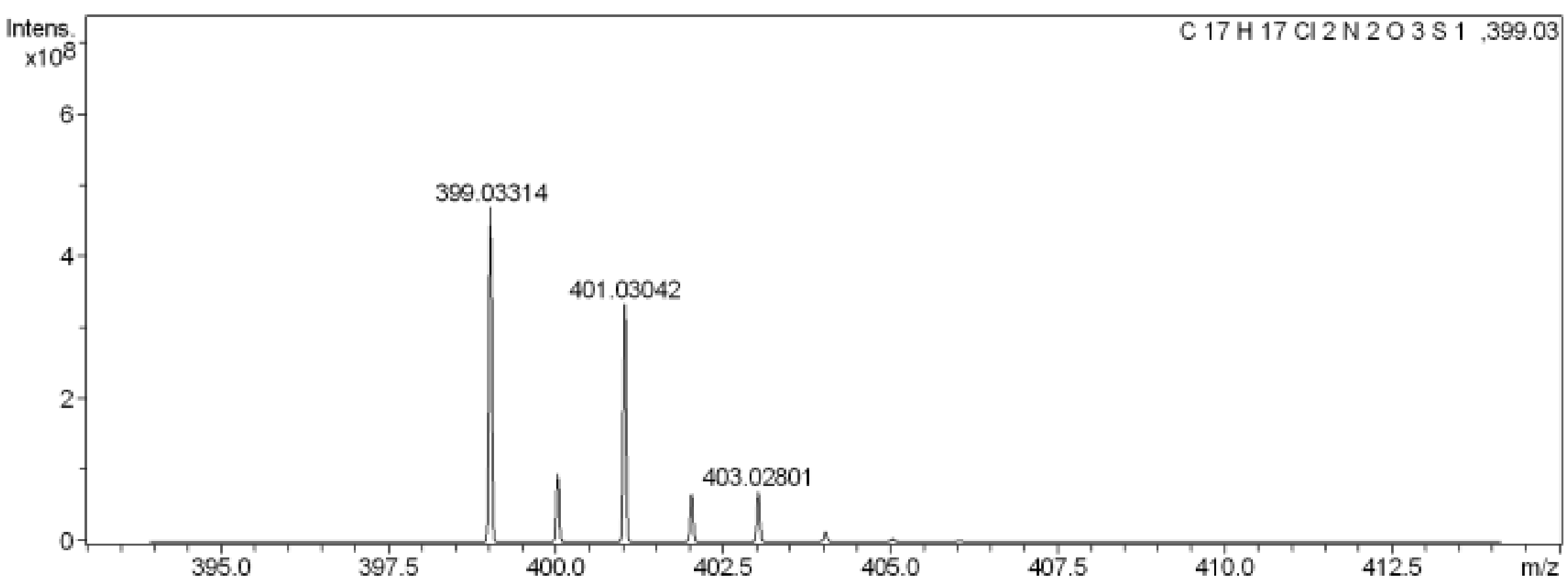


| Sum Formula | Sigma | m/z | Err [ppm] | Mean Err [ppm] | Err [mDa] | rdb | N Rule | e⁻ |
|---|---|---|---|---|---|---|---|---|
| C 17 H 17 Cl 2 N 2 O 3 S 1 | 0.010 | 399.03314 | 0.13 | -0.26 | 0.05 | 9.50 | ok | even |

Fig.S39. HRMS spectrum of compound 10c

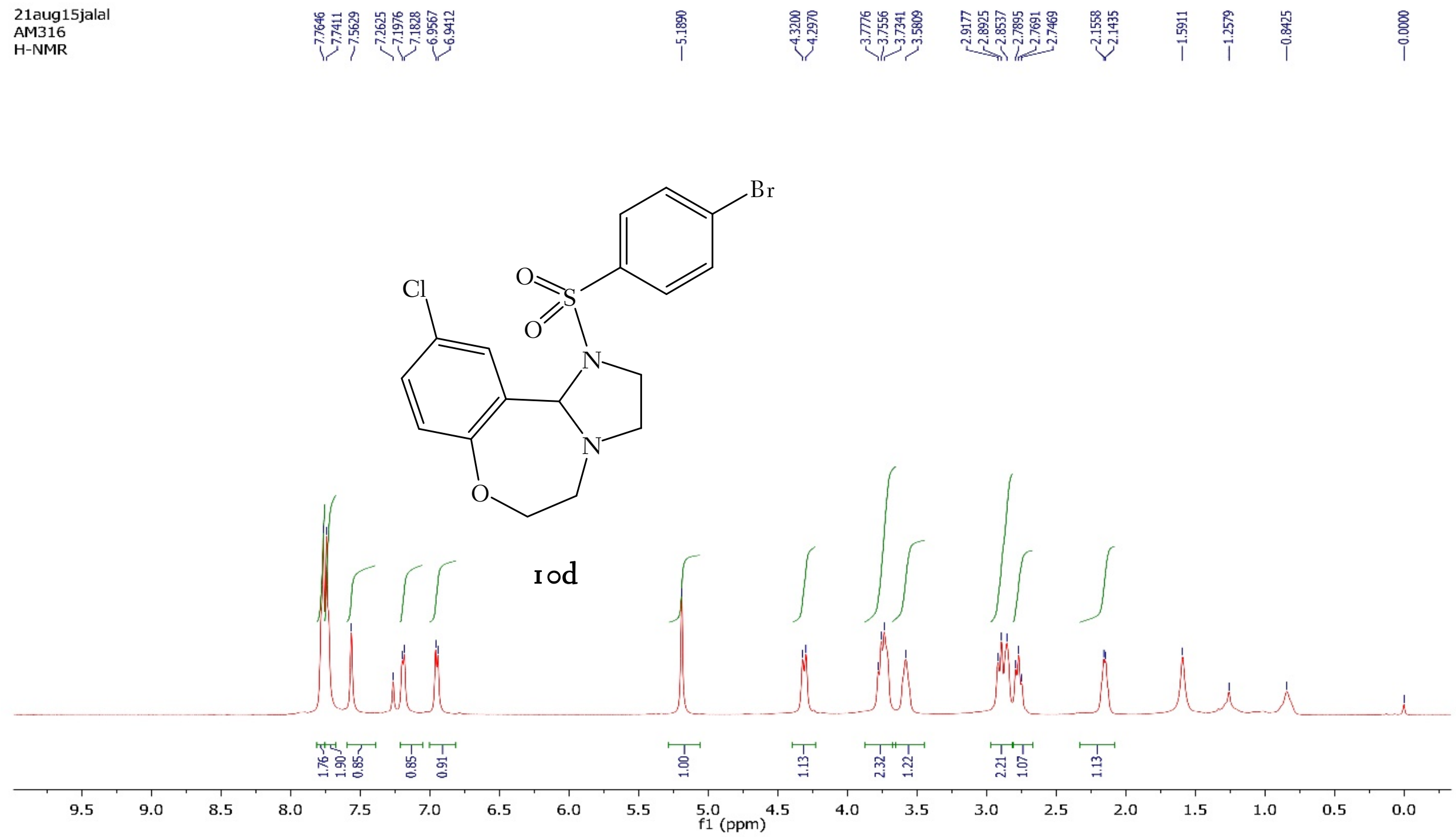


Fig.S40. $^{1}$H NMR spectrum of compound 10d $CDCl_3$

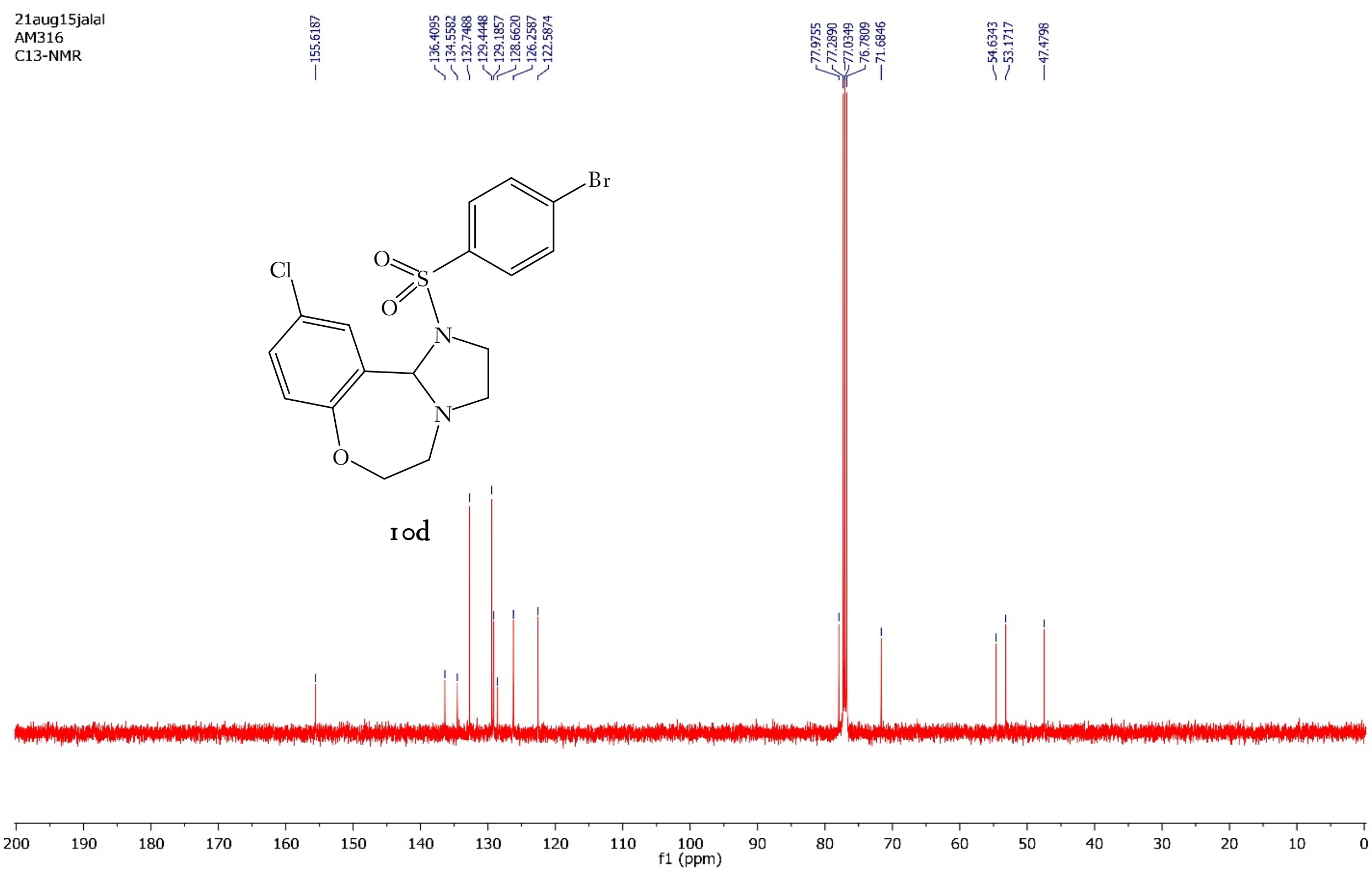


Fig.S41. $^{13}$C NMR spectrum of compound 10d $CDCl_3$

# The University of Jordan
## Faculty of science
## Department of Chemistry

## Mass Spectrum Molecular Formula Report

**Analysis Info**

| | | | |
|---|---|---|---|
| Analysis Name | F:\Data\2022MAR20\Mustafa_000048.d | Acquisition Date | 3/23/2022 1:58:04 PM |
| Method | ESI_pos_20181024 | Operator | Bruker_PC |
| Sample Name | AM316 | Instrument | apex-IV |
| Comment | CHCl3+ACN | | |

**Acquisition Parameter**

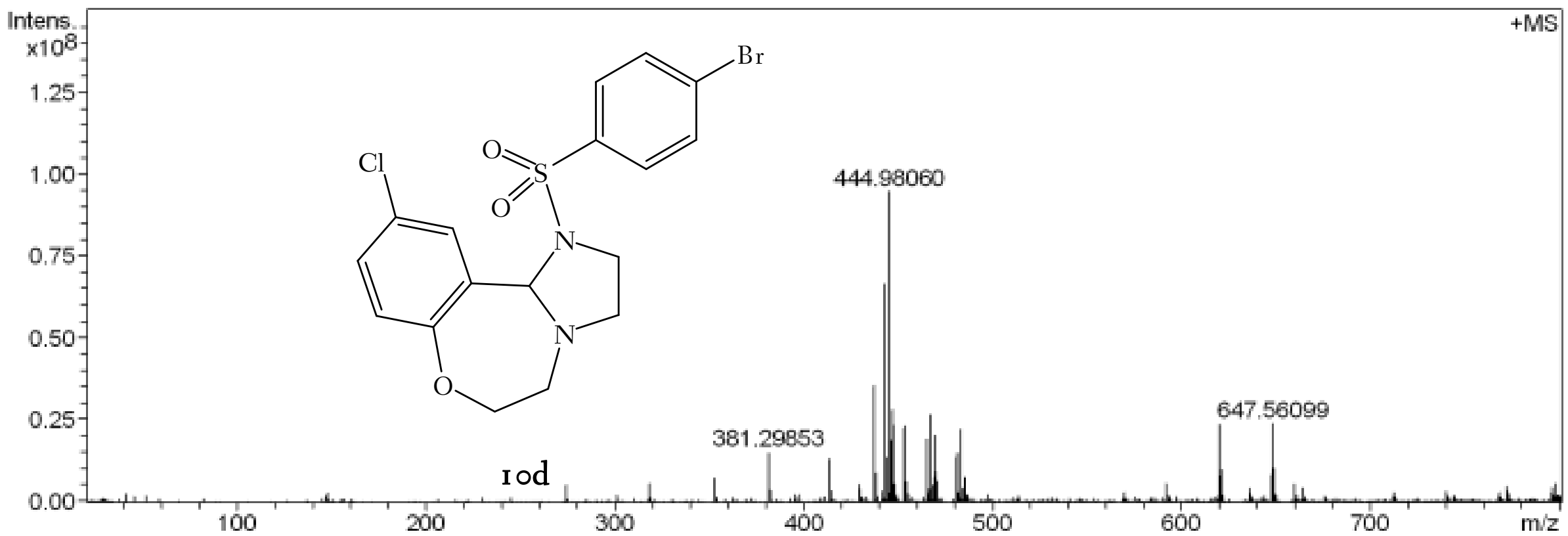


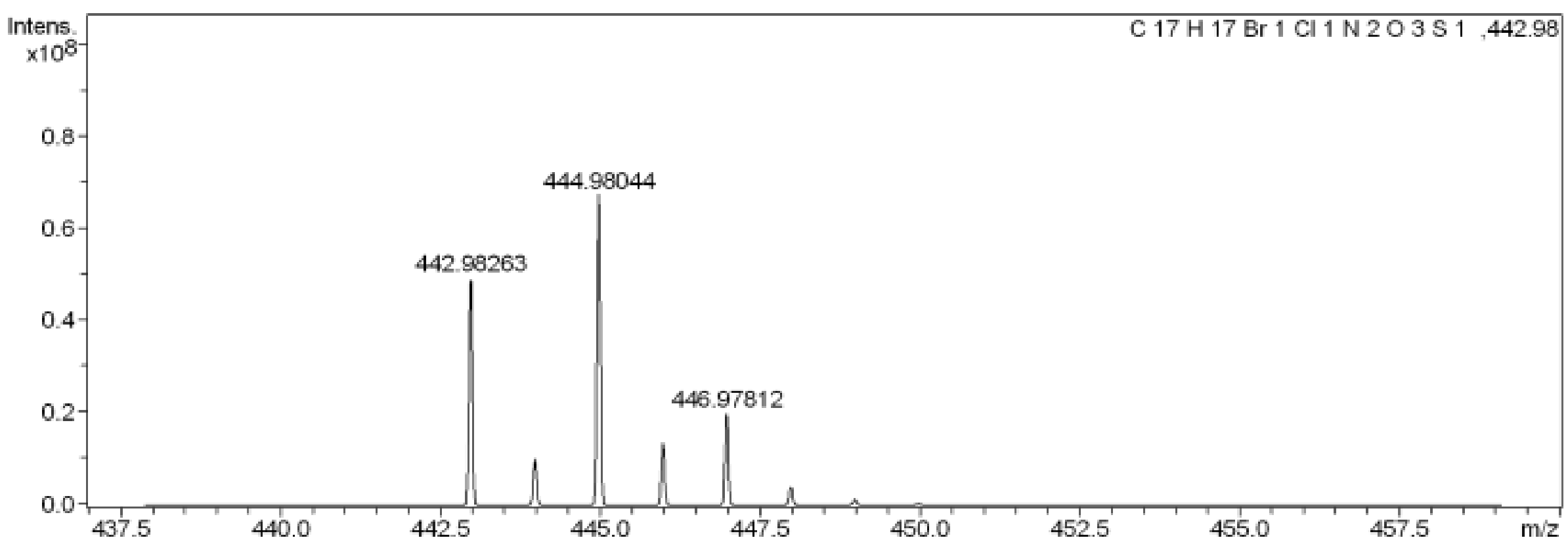


| Sum Formula | Sigma | m/z | Err [ppm] | Mean Err [ppm] | Err [mDa] | rdb | N Rule | e⁻ |
|---|---|---|---|---|---|---|---|---|
| C 17 H 17 Br 1 Cl 1 N 2 O 3 S 1 | 0.010 | 442.98263 | -0.50 | -0.85 | -0.22 | 9.50 | ok | even |

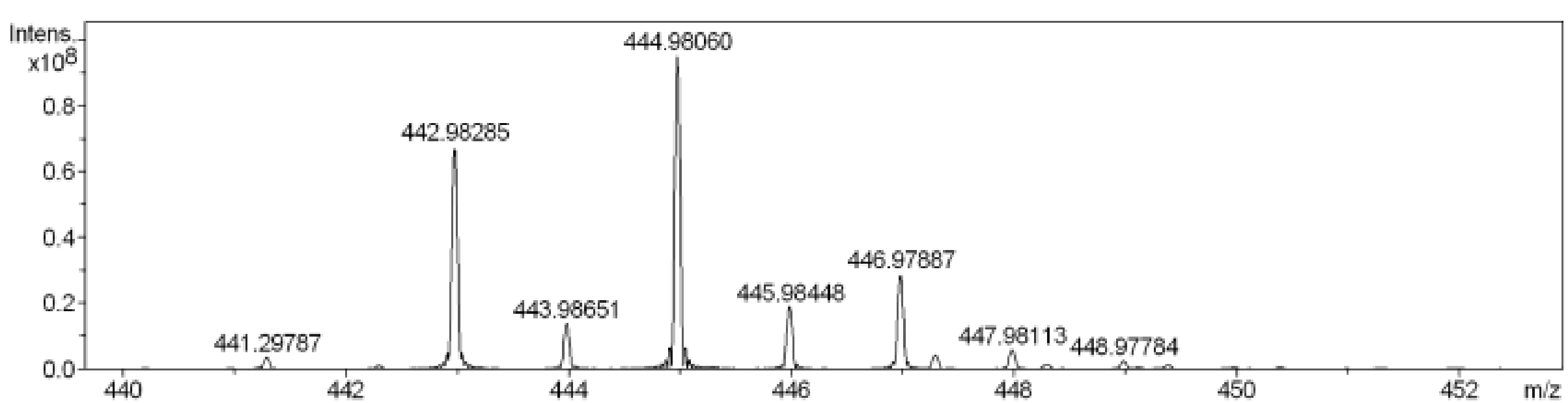


Fig.S42. HRMS spectrum of compound 10d

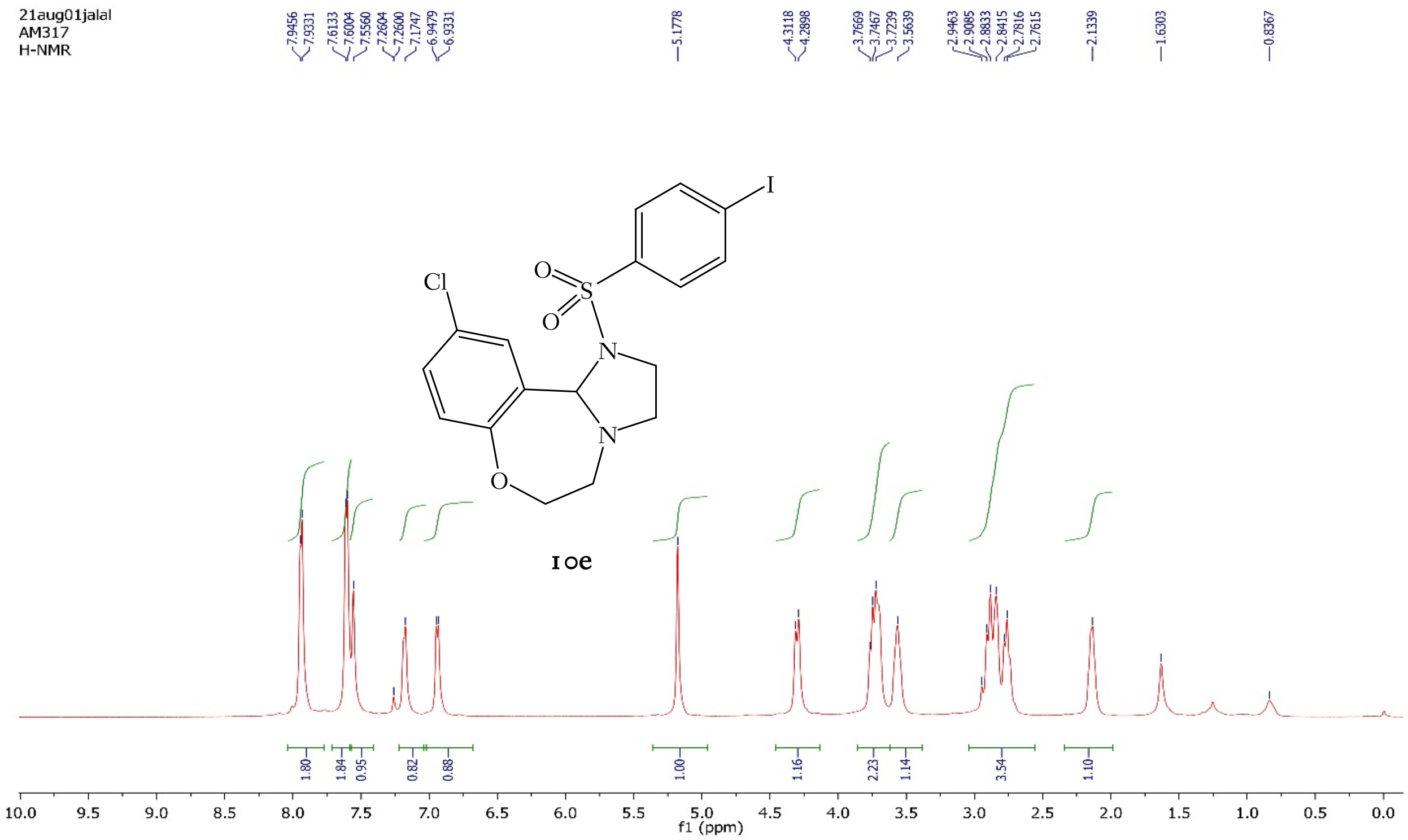


Fig.S43. $^{1}$H NMR spectrum of compound 10e $CDCl_3$

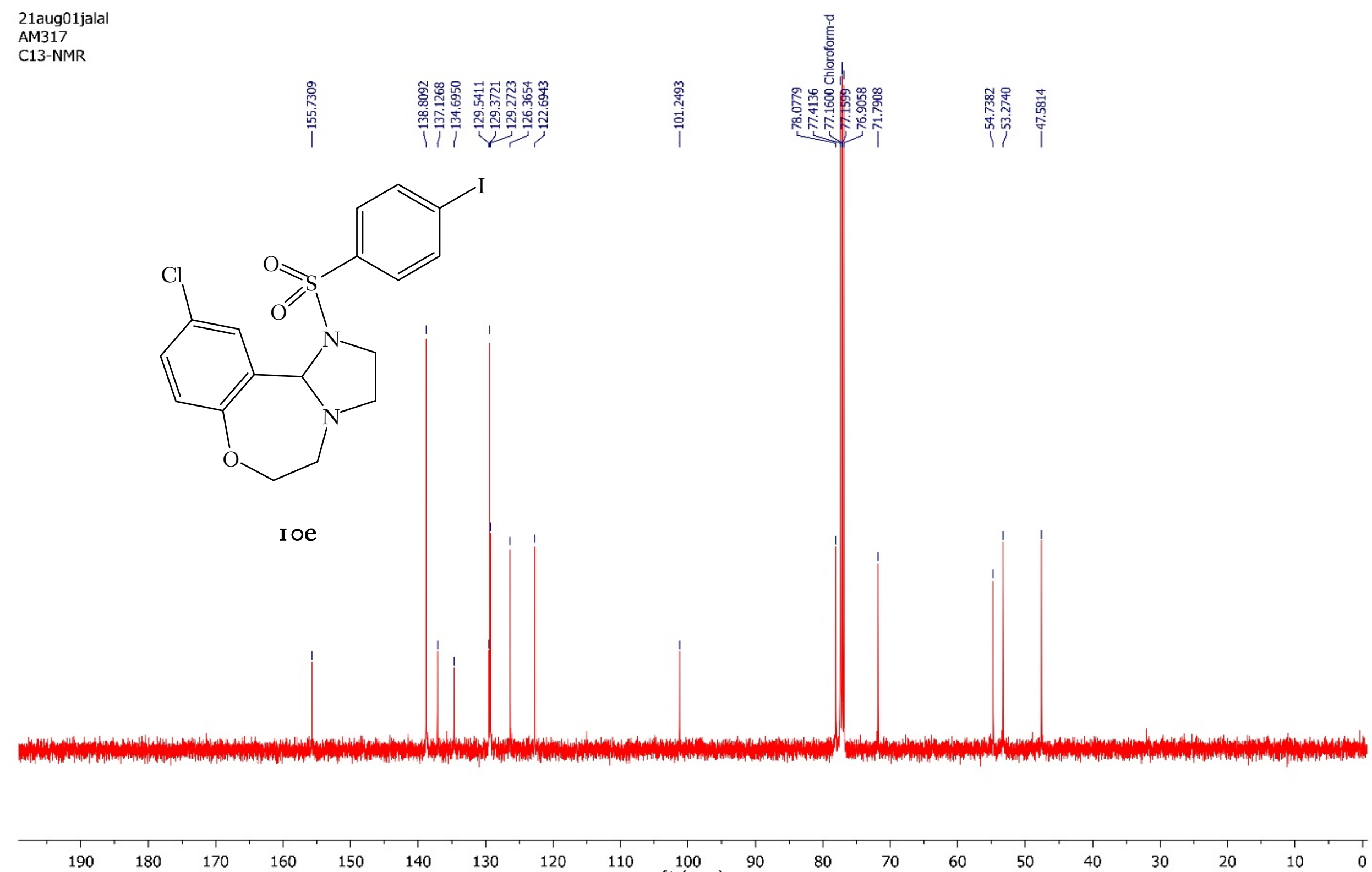

Fig.S44. $^{13}C$ NMR spectrum of compound 10e $CDCl_3$

# The University of Jordan
## Faculty of science
## Department of Chemistry

## Mass Spectrum Molecular Formula Report

**Analysis Info**

| | | | |
|---|---|---|---|
| Analysis Name | F:\Data\2022MAR30\Mustafa_000012.d | Acquisition Date | 3/30/2022 11:25:43 AM |
| Method | ESI_pos_20181024 | Operator | Bruker_PC |
| Sample Name | AM317 | Instrument | apex-IV |
| Comment | CHCl3+ACN | | |

**Acquisition Parameter**

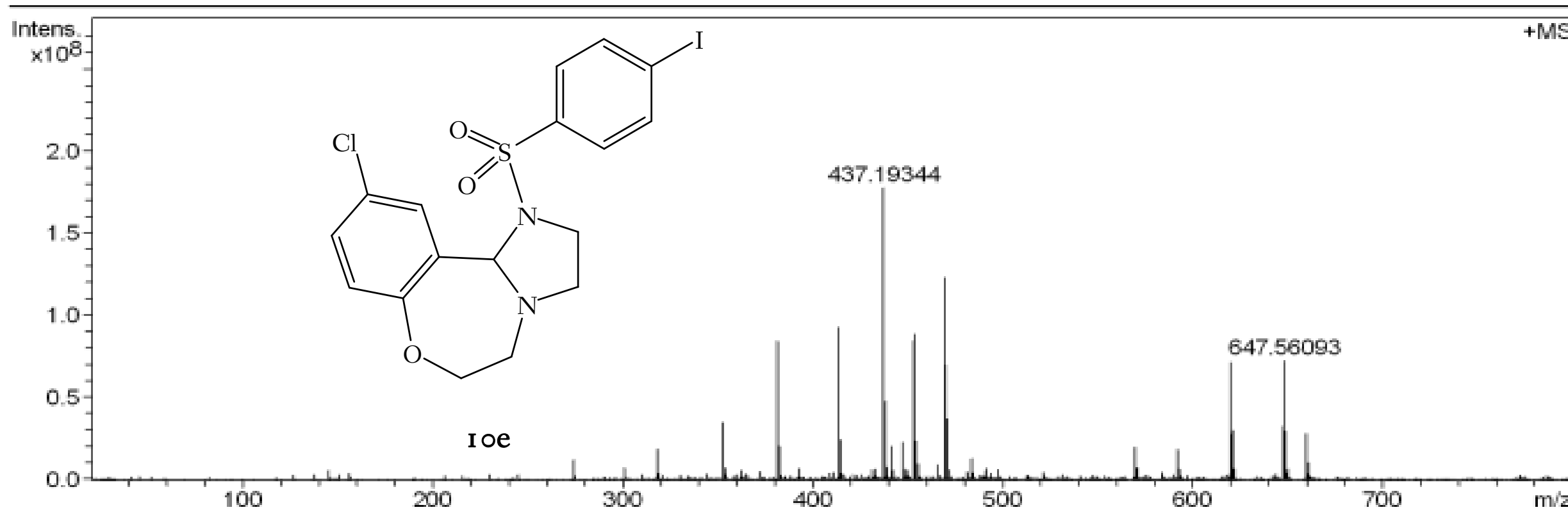


10e

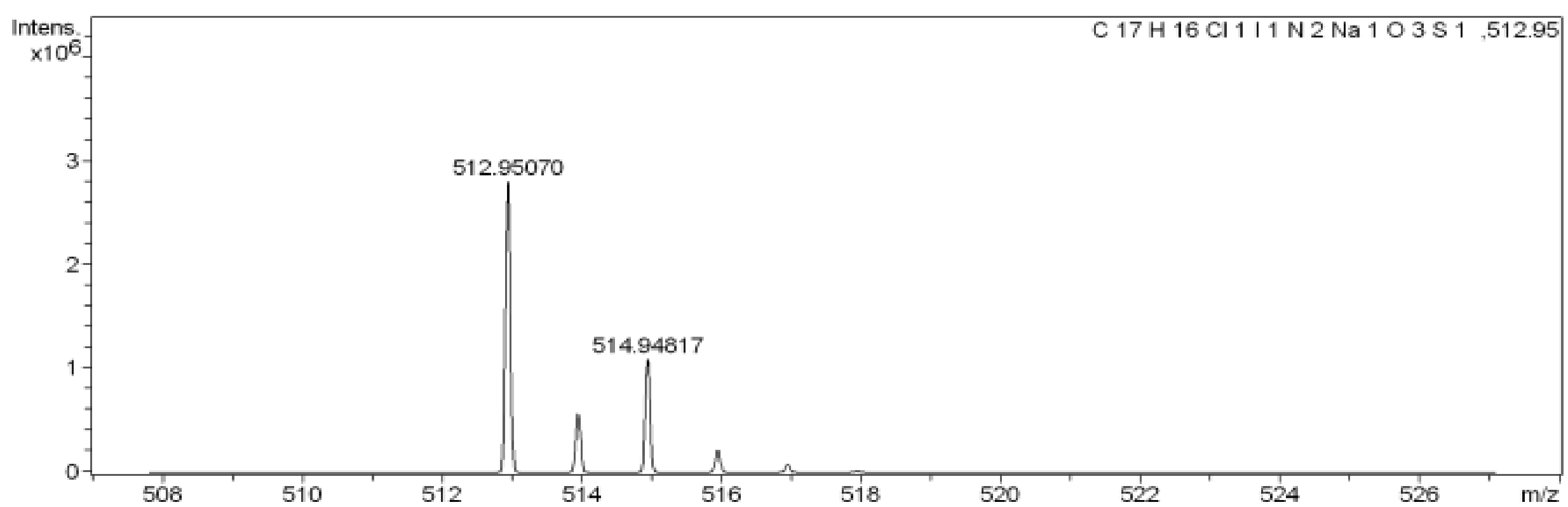


| Sum Formula | Sigma | m/z | Err [ppm] | Mean Err [ppm] | Err [mDa] | rdb | N Rule | e⁻ |
|---|---|---|---|---|---|---|---|---|
| C 17 H 16 Cl 1 I 1 N 2 Na 1 O 3 S 1 | 0.097 | 512.95070 | -2.47 | -1.49 | -1.27 | 9.50 | ok | even |

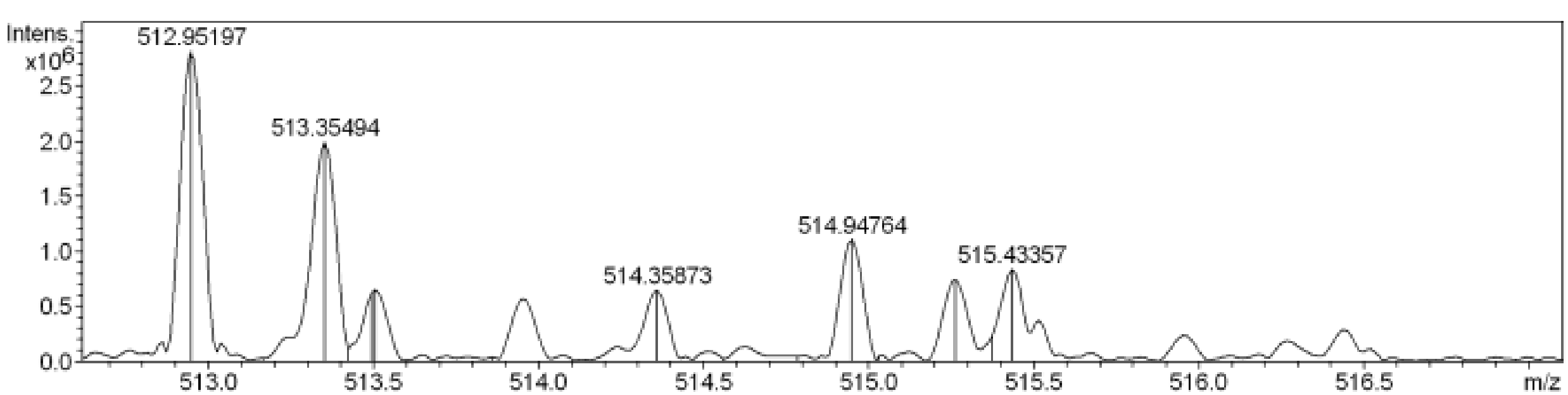

Fig.S45. HRMS spectrum of compound 10e

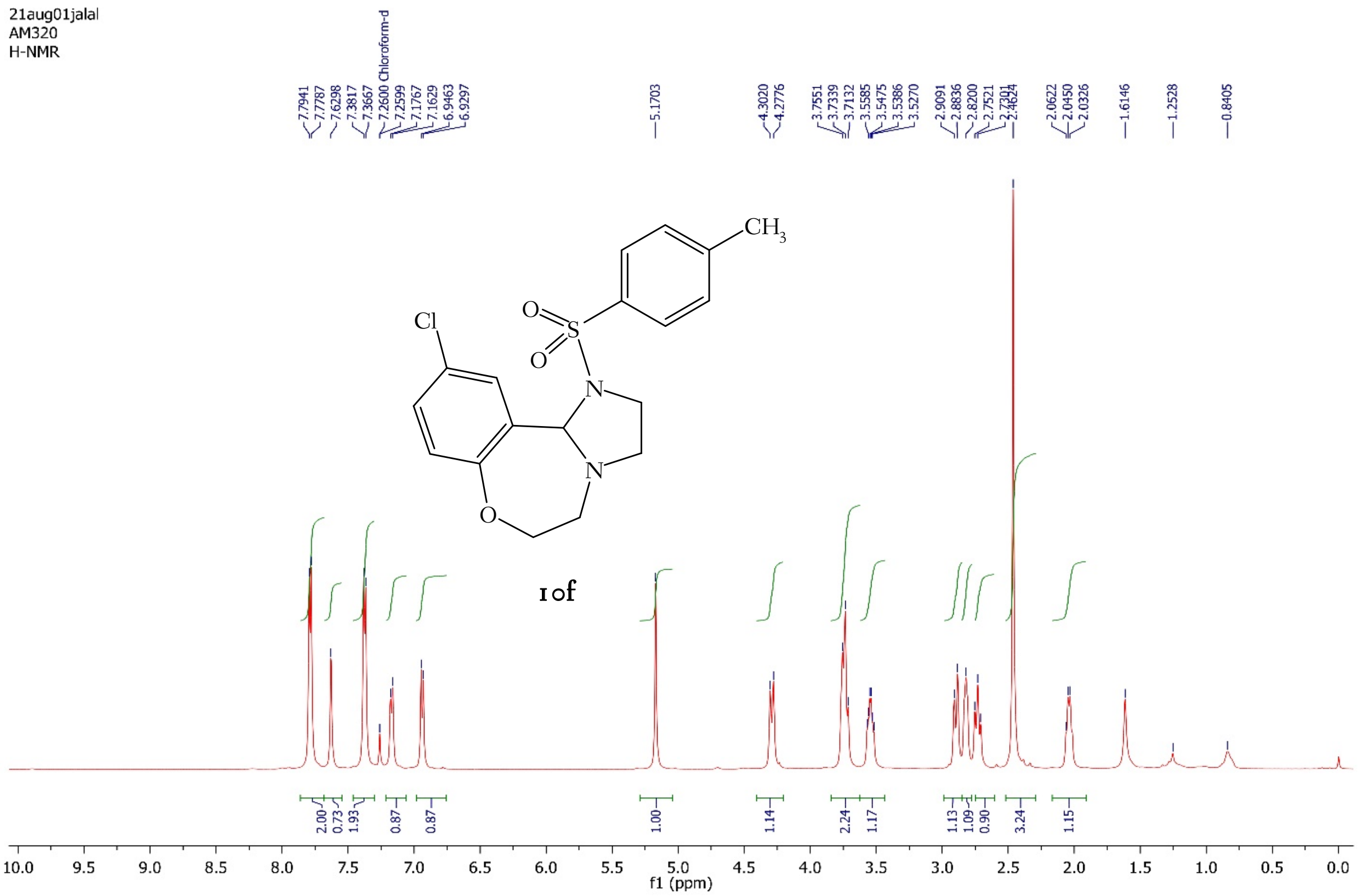


Fig.S46. $^{1}$H NMR spectrum of compound 10f $CDCl_3$

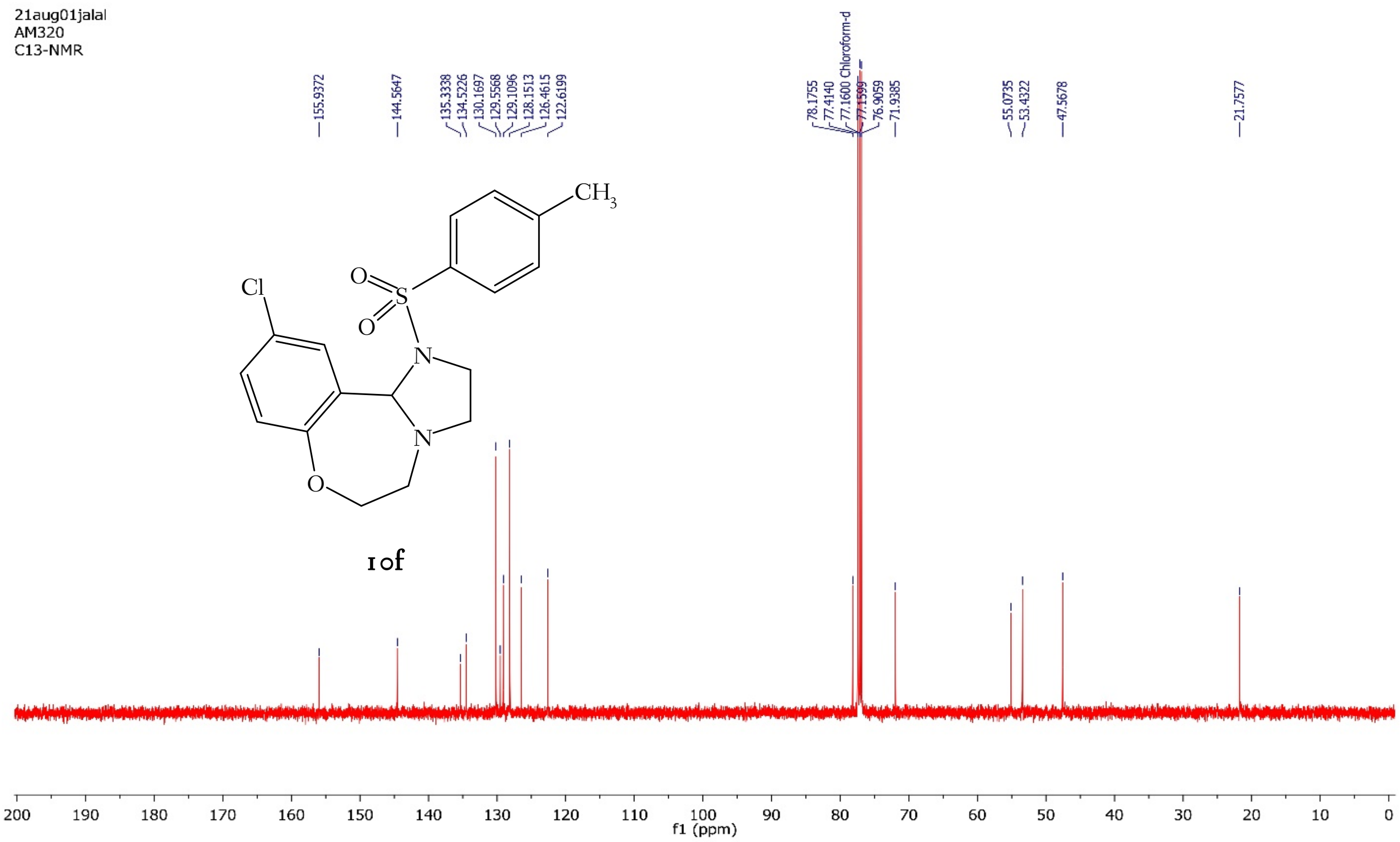


Fig.S47. $^{13}$C NMR spectrum of compound 10f CDCl$_3$

# The University of Jordan
## Faculty of science
## Department of Chemistry

### Mass Spectrum Molecular Formula Report

| | | | |
|---|---|---|---|
| **Analysis Info** | | Acquisition Date | 3/30/2022 11:06:38 AM |
| Analysis Name | F:\Data\2022MAR30\Mustafa_000009.d | | |
| Method | ESI_pos_20181024 | Operator | Bruker_PC |
| Sample Name | AM320 | Instrument | apex-IV |
| Comment | CHCl3+ACN | | |

**Acquisition Parameter**

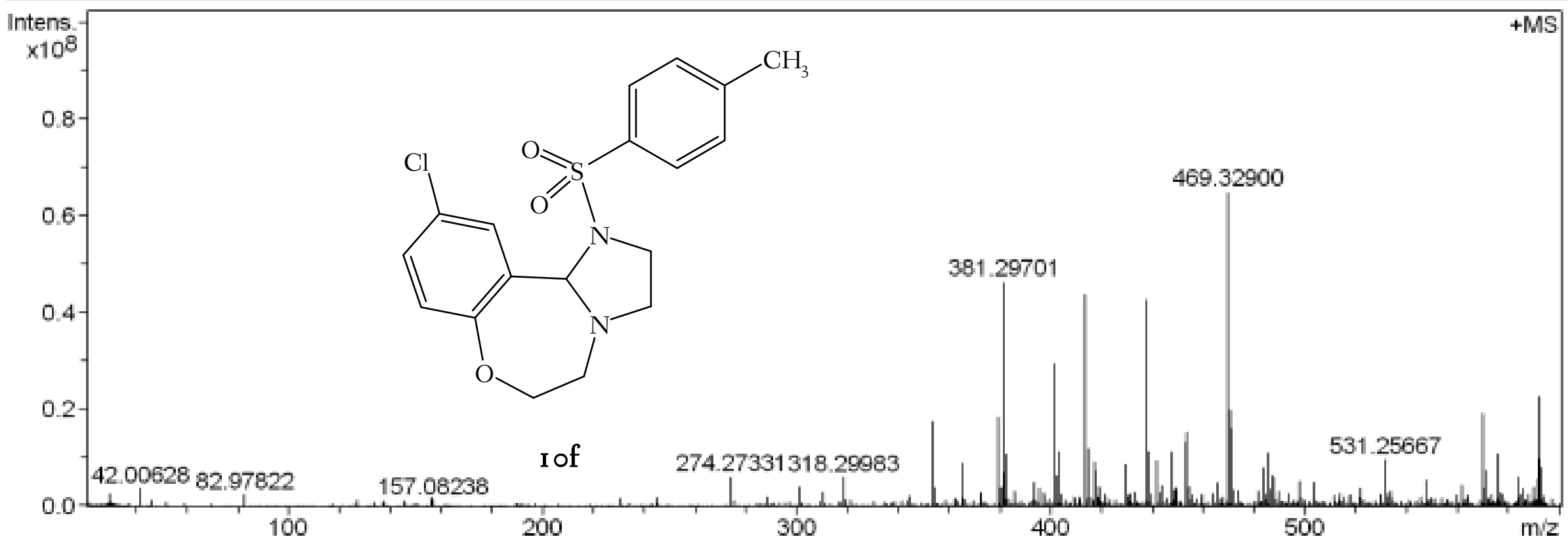


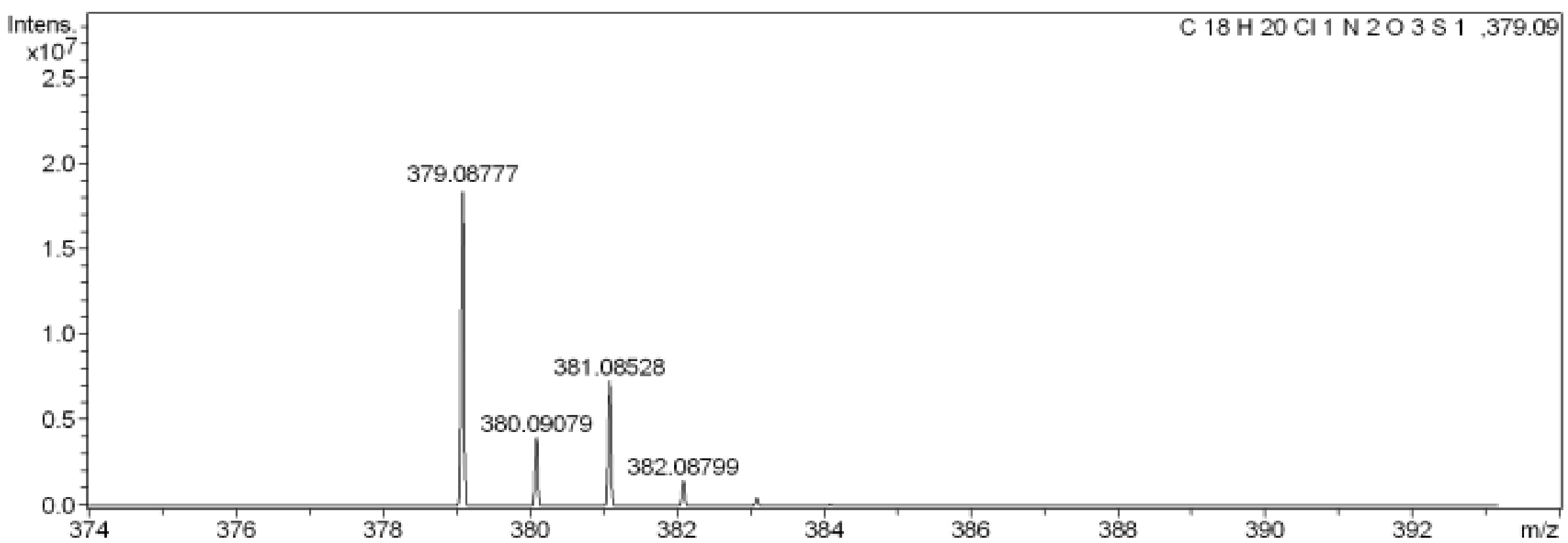


| Sum Formula | Sigma | m/z | Err [ppm] | Mean Err [ppm] | Err [mDa] | rdb | N Rule | e⁻ |
|---|---|---|---|---|---|---|---|---|
| C 18 H 20 Cl 1 N 2 O 3 S 1 | 0.013 | 379.08777 | 0.72 | 1.64 | 0.27 | 9.50 | ok | even |

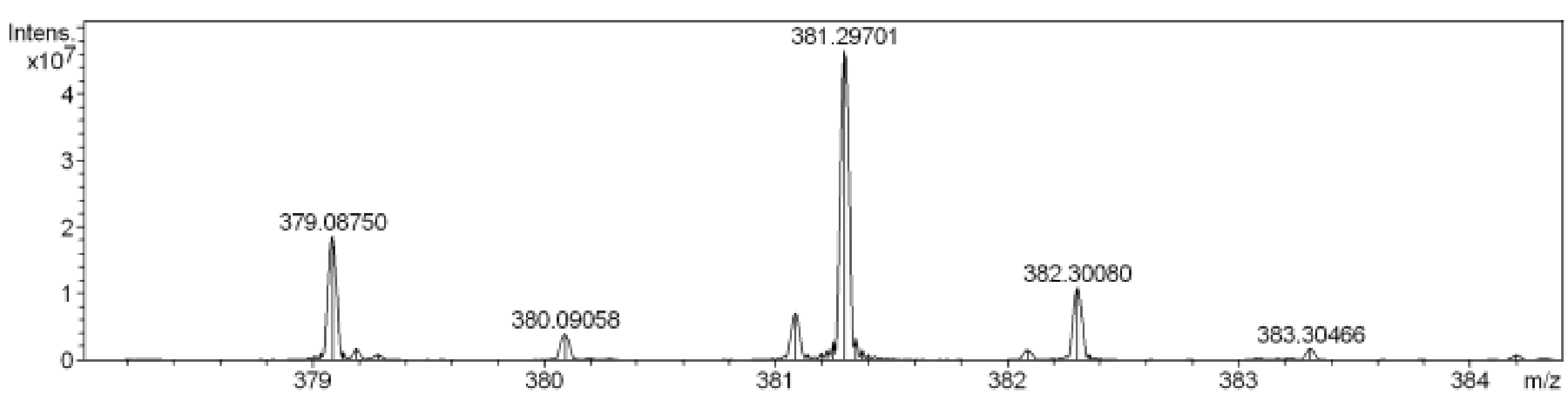


Fig.S48. HRMS spectrum of compound 10f

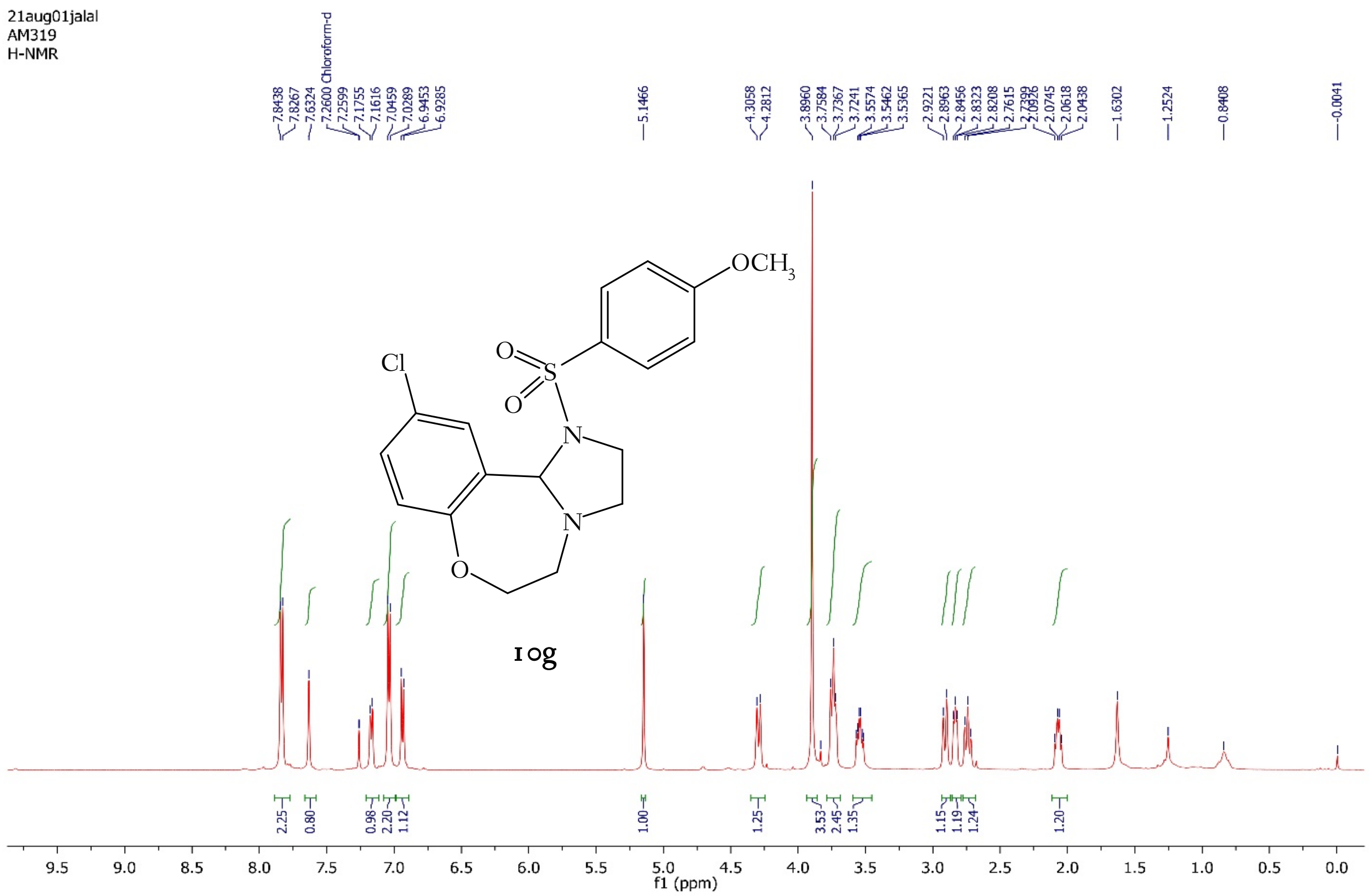


Fig.S49. $^{1}$H NMR spectrum of compound 10g $CDCl_3$

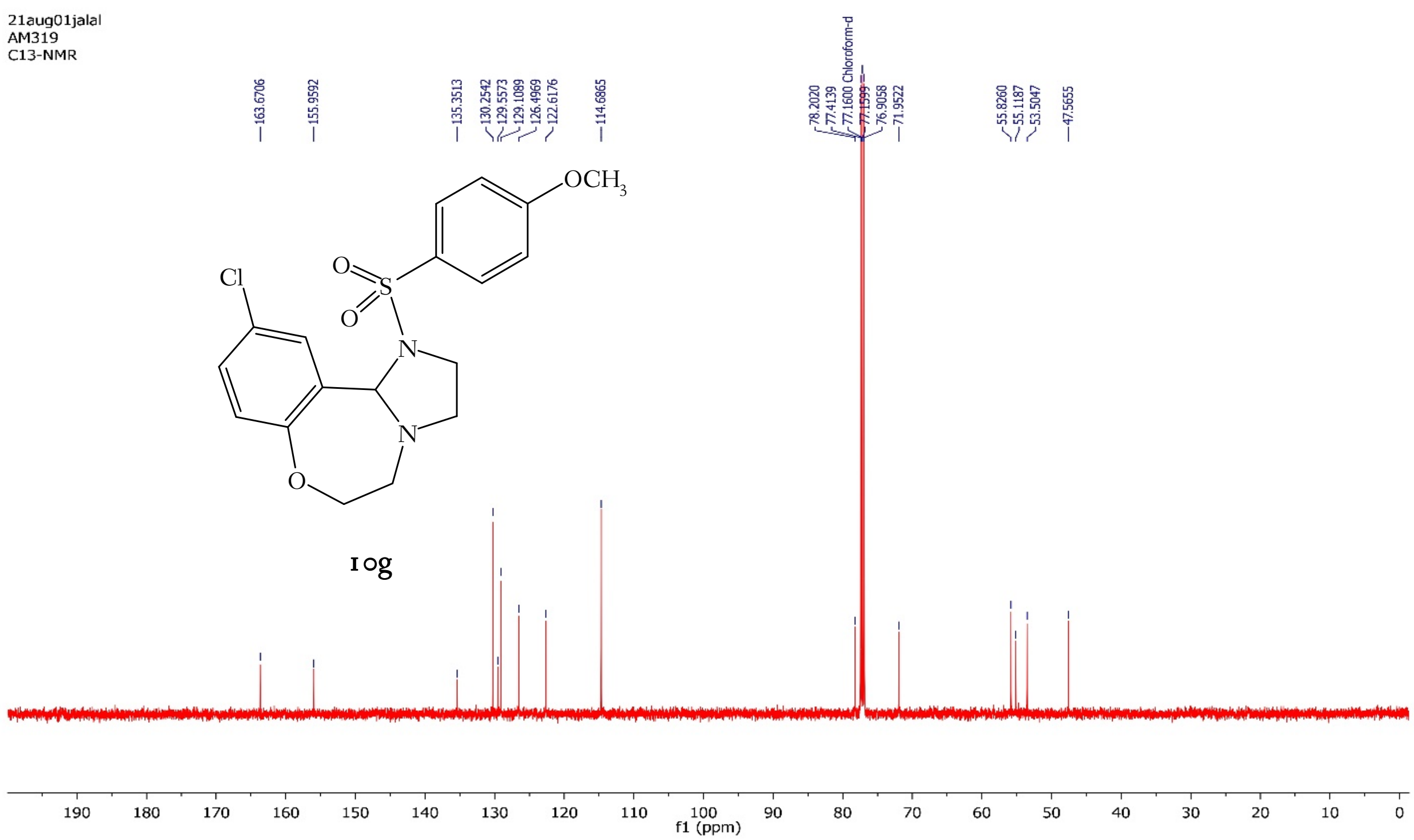


Fig.S50. $^{13}$C NMR spectrum of compound 10g $CDCl_3$

# The University of Jordan
## Faculty of science
## Department of Chemistry

## Mass Spectrum Molecular Formula Report

**Analysis Info**

| | | | |
|---|---|---|---|
| Analysis Name | F:\Data\2022MAR14\Mustafa_000042.d | Acquisition Date | 3/15/2022 12:19:15 PM |
| Method | ESI_pos_20181024 | Operator | Bruker_PC |
| Sample Name | AM319 | Instrument | apex-IV |
| Comment | CHCl3+ACN | | |

**Acquisition Parameter**

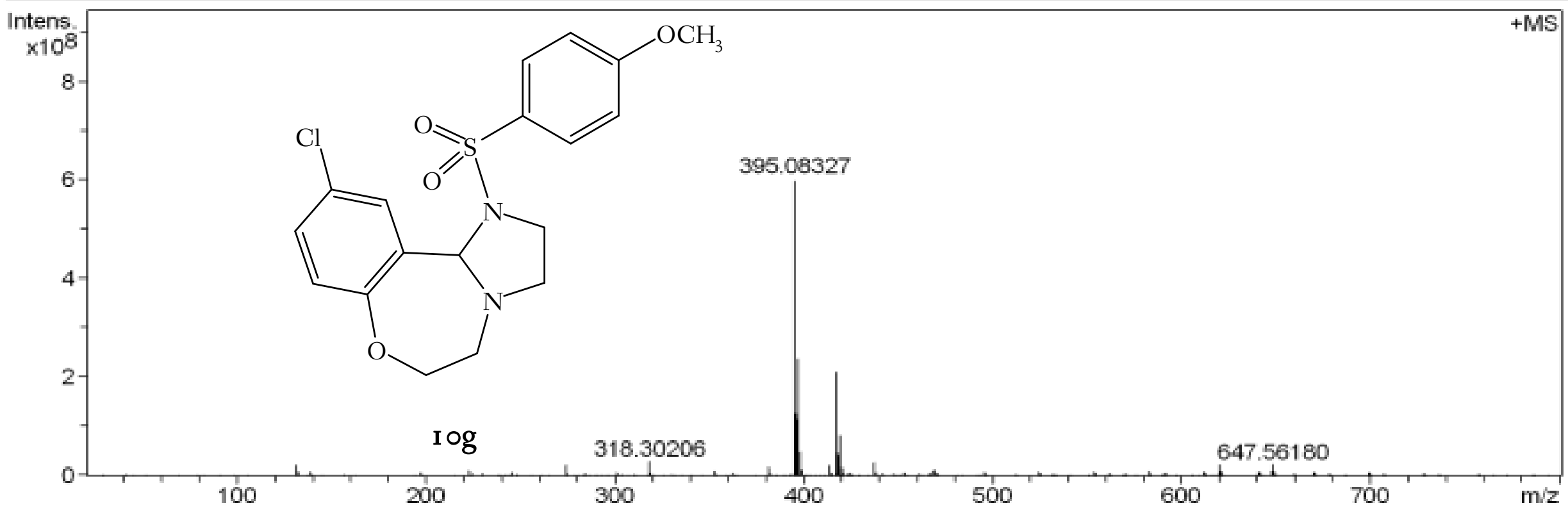


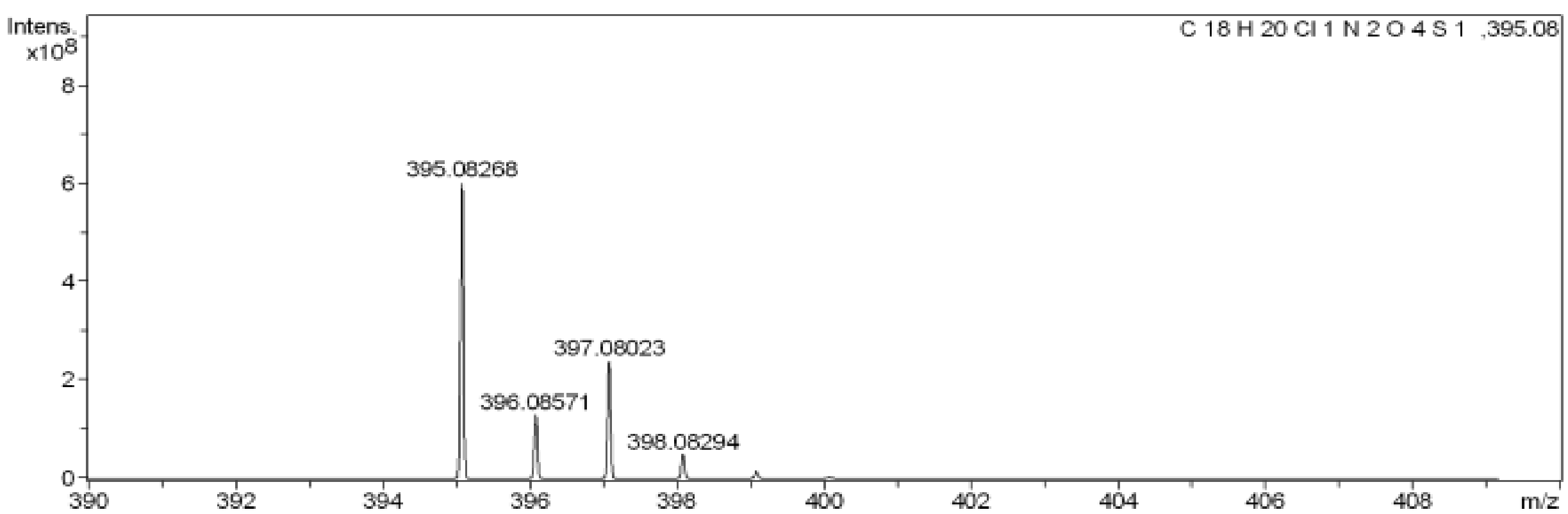


| Sum Formula | Sigma | m/z | Err [ppm] | Mean Err [ppm] | Err [mDa] | rdb | N Rule | e⁻ |
|---|---|---|---|---|---|---|---|---|
| C 18 H 20 Cl 1 N 2 O 4 S 1 | 0.003 | 395.08268 | -1.50 | -1.56 | -0.59 | 9.50 | ok | even |

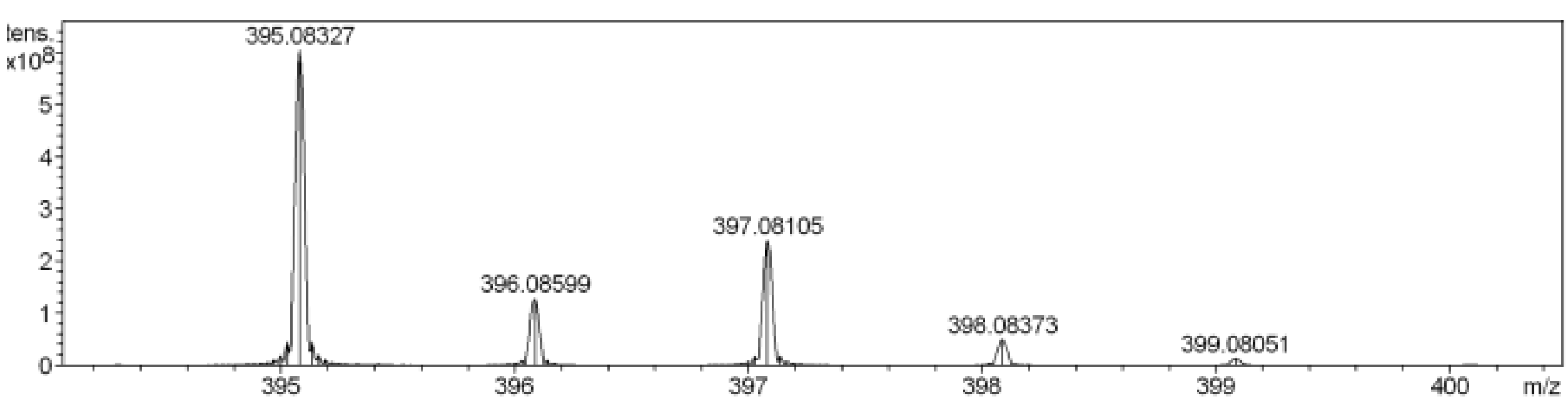


Fig.S51. HRMS spectrum of compound 10g

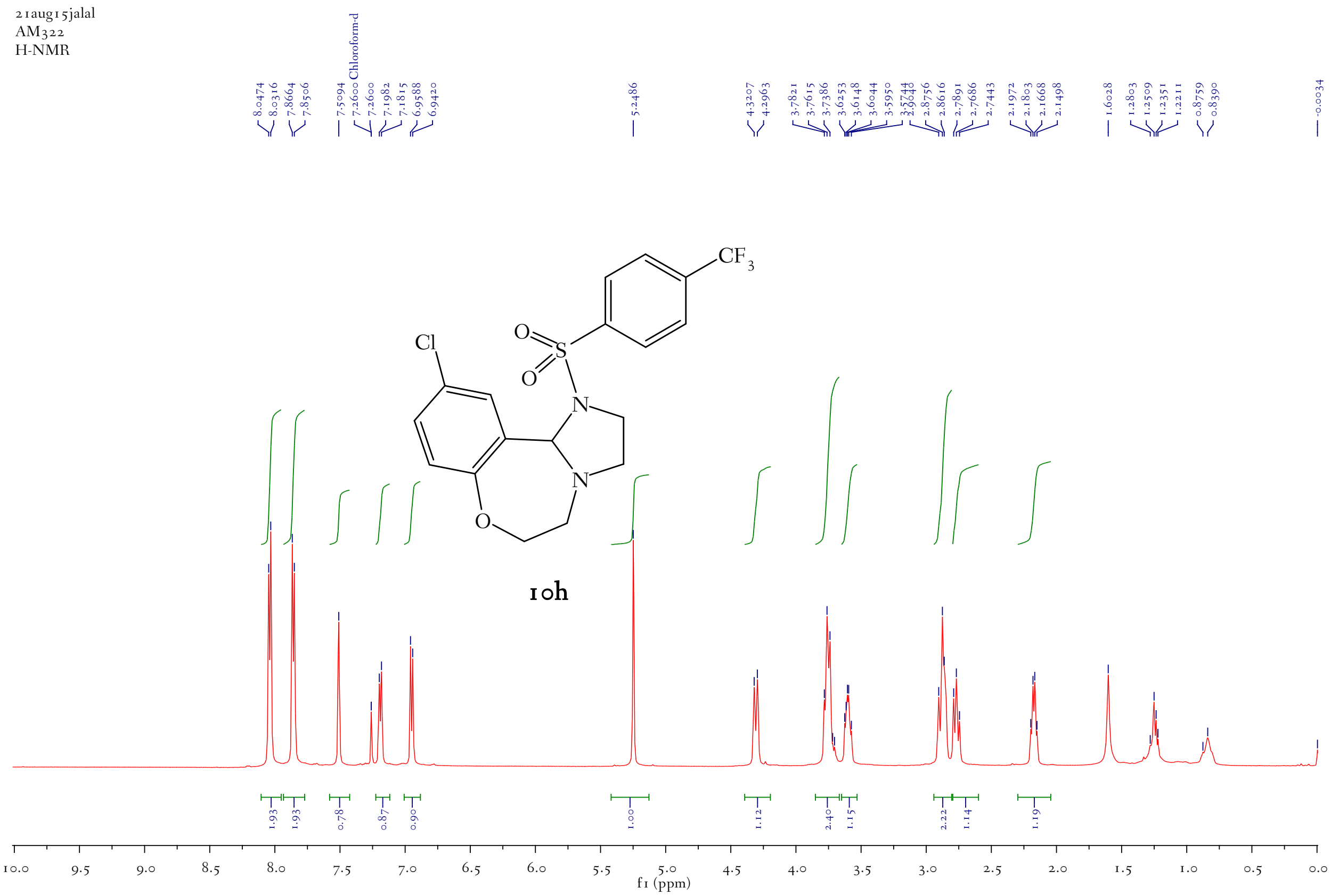


Fig.S52. $^{1}H$ NMR spectrum of compound 10h $CDCl_3$

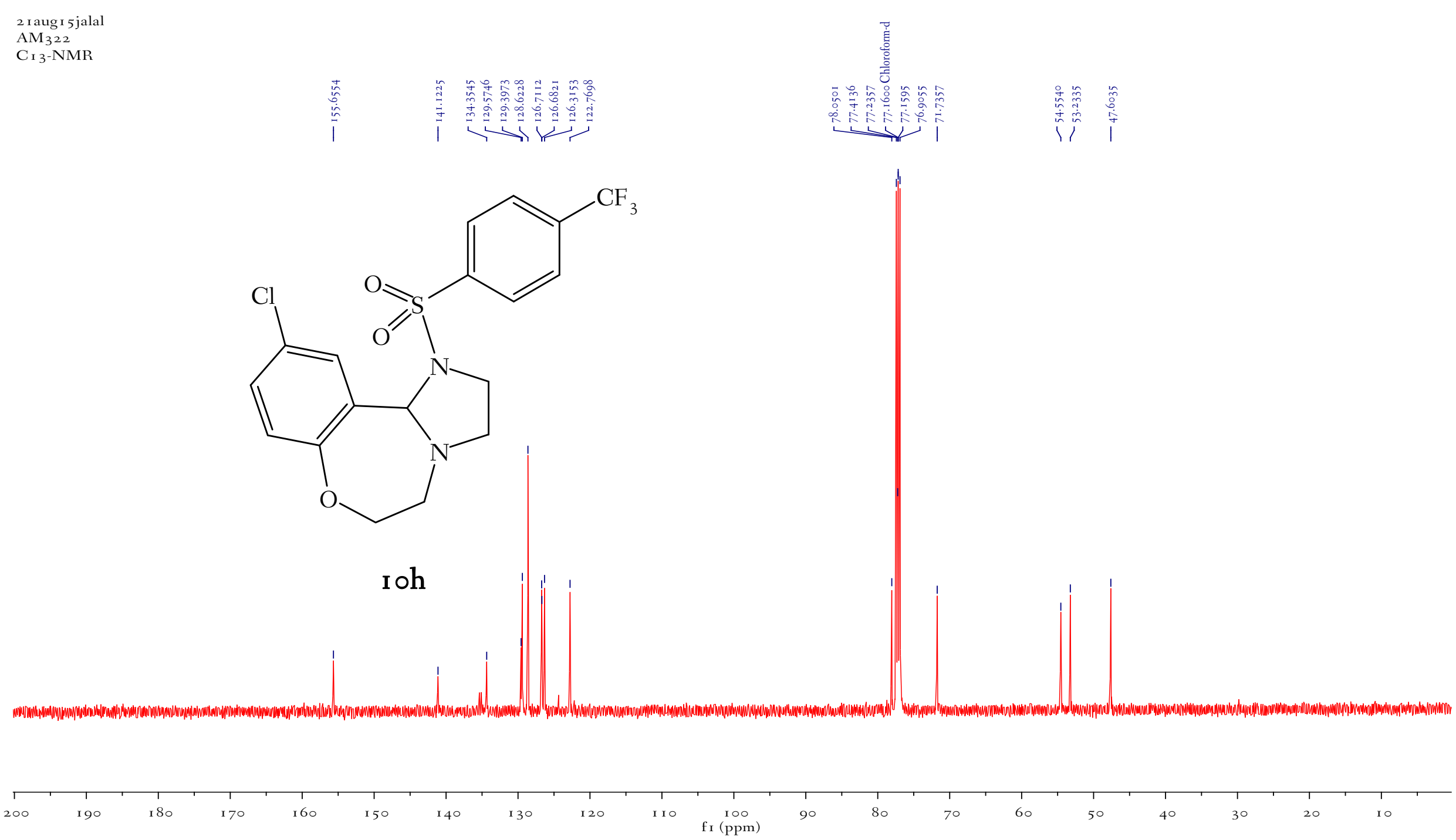


Fig.S53. $^{13}C$ NMR spectrum of compound 10h $CDCl_3$

**The University of Jordan**

**Faculty of science**

**Department of Chemistry**


## Mass Spectrum Molecular Formula Report

**Analysis Info**

| | | | |
|---|---|---|---|
| Analysis Name | F:\Data\2022MAR14\Mustafa_000016.d | Acquisition Date | 3/14/2022 2:52:46 PM |
| Method | ESI_pos_20181024 | Operator | Bruker_PC |
| Sample Name | AM322 | Instrument | apex-IV |
| Comment | CHCl3+ACN | | |

**Acquisition Parameter**

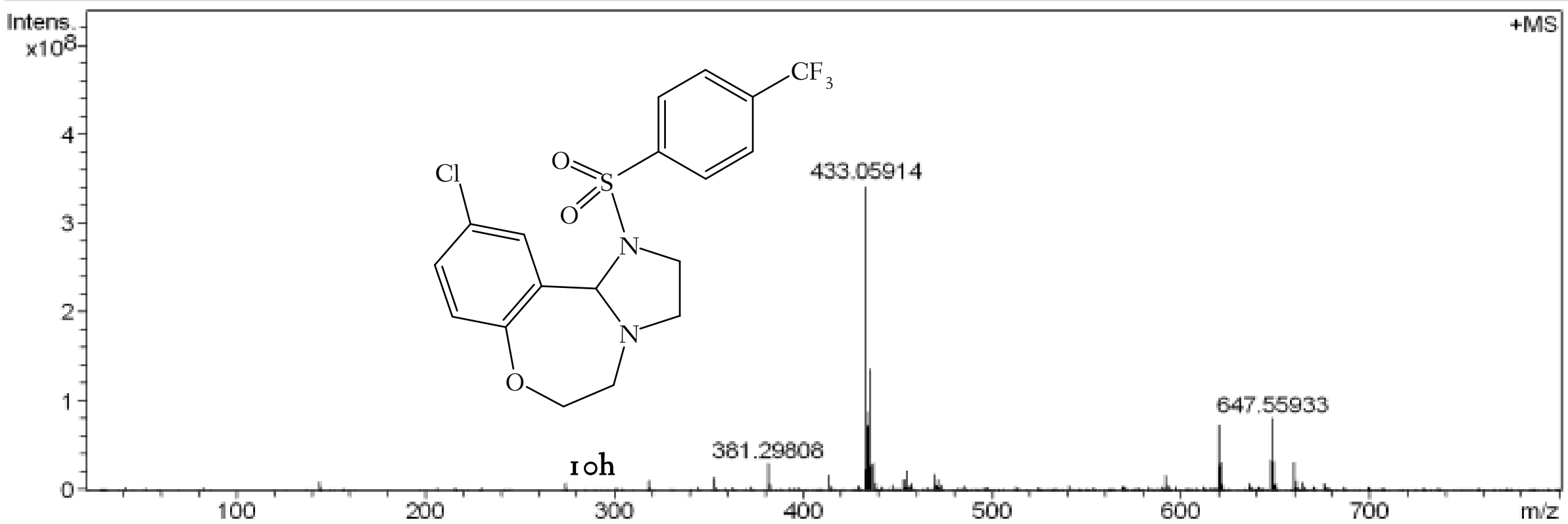


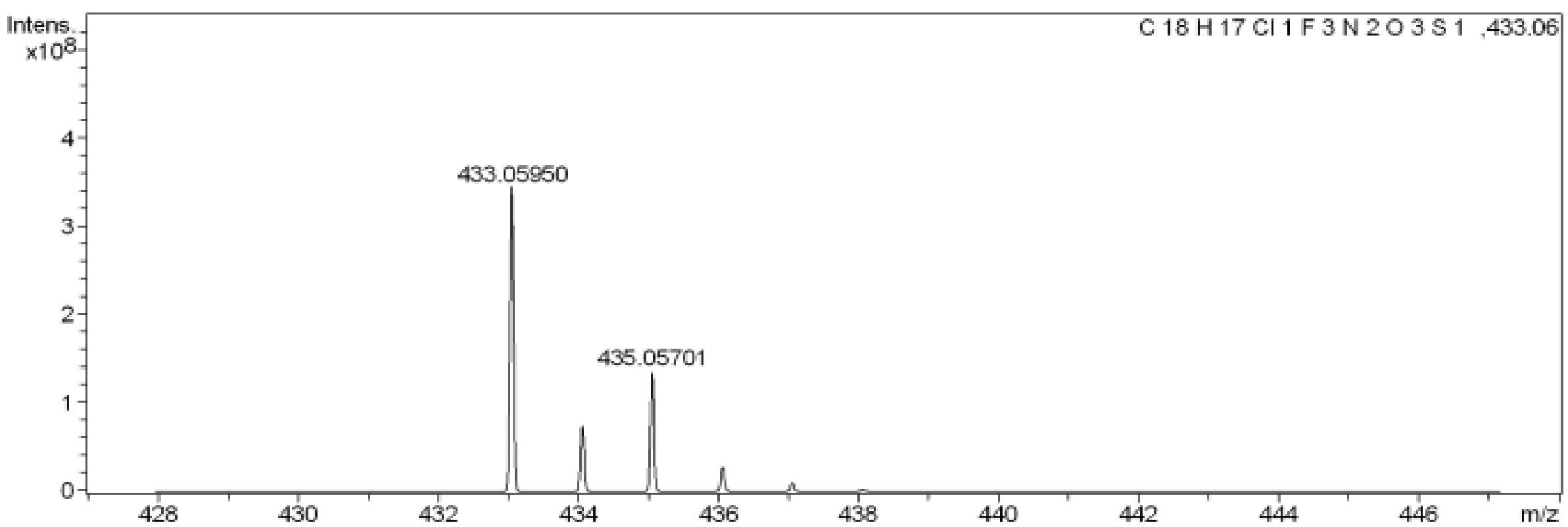


| Sum Formula | Sigma | m/z | Err [ppm] | Mean Err [ppm] | Err [mDa] | rdb | N Rule | e⁻ |
|---|---|---|---|---|---|---|---|---|
| C 18 H 17 Cl 1 F 3 N 2 O 3 S 1 | 0.003 | 433.05950 | 0.83 | 0.45 | 0.36 | 9.50 | ok | even |

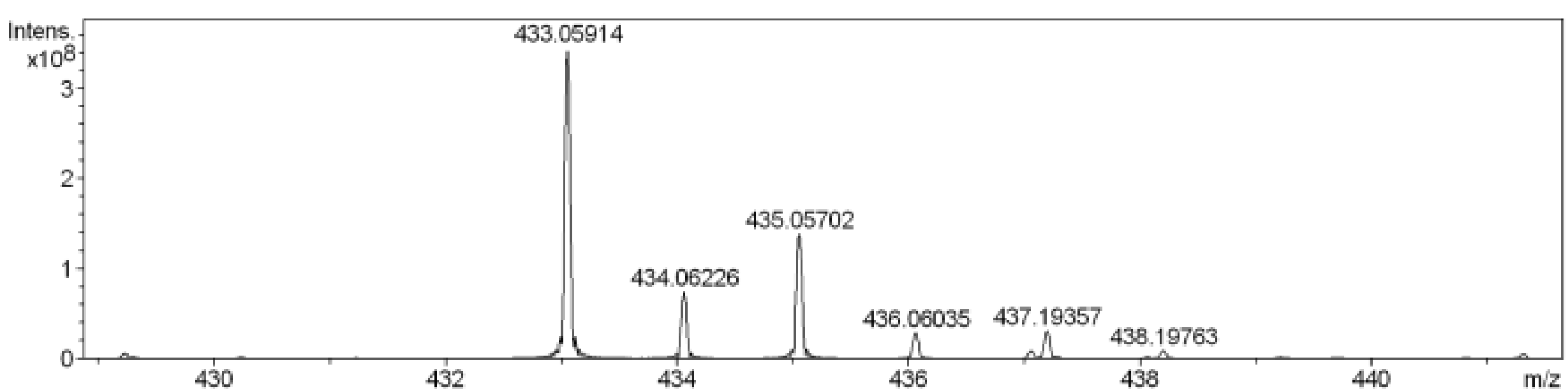


Fig.S54. HRMS spectrum of compound 10h

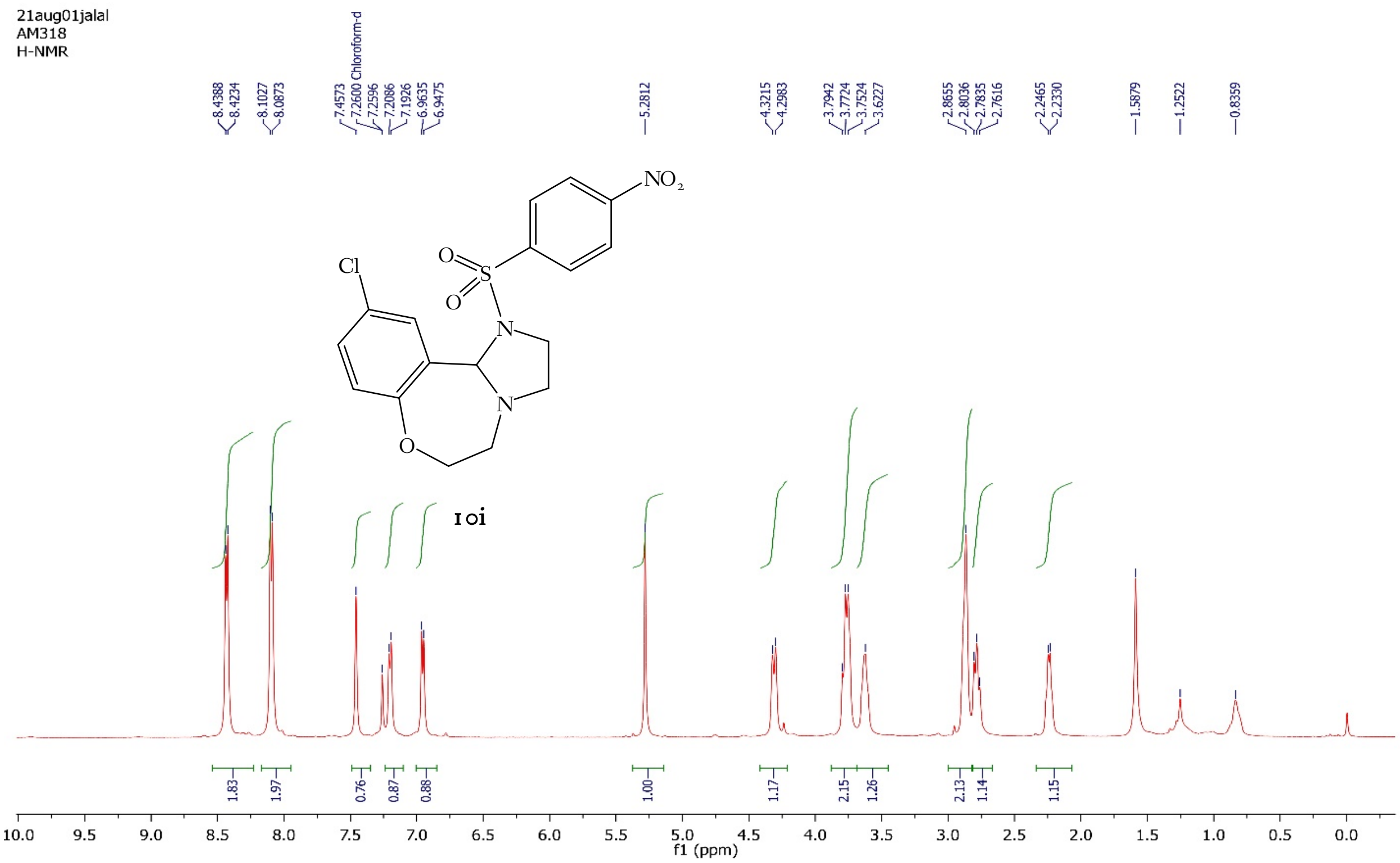


Fig.S55. $^{1}$H NMR spectrum of compound 10i $CDCl_3$

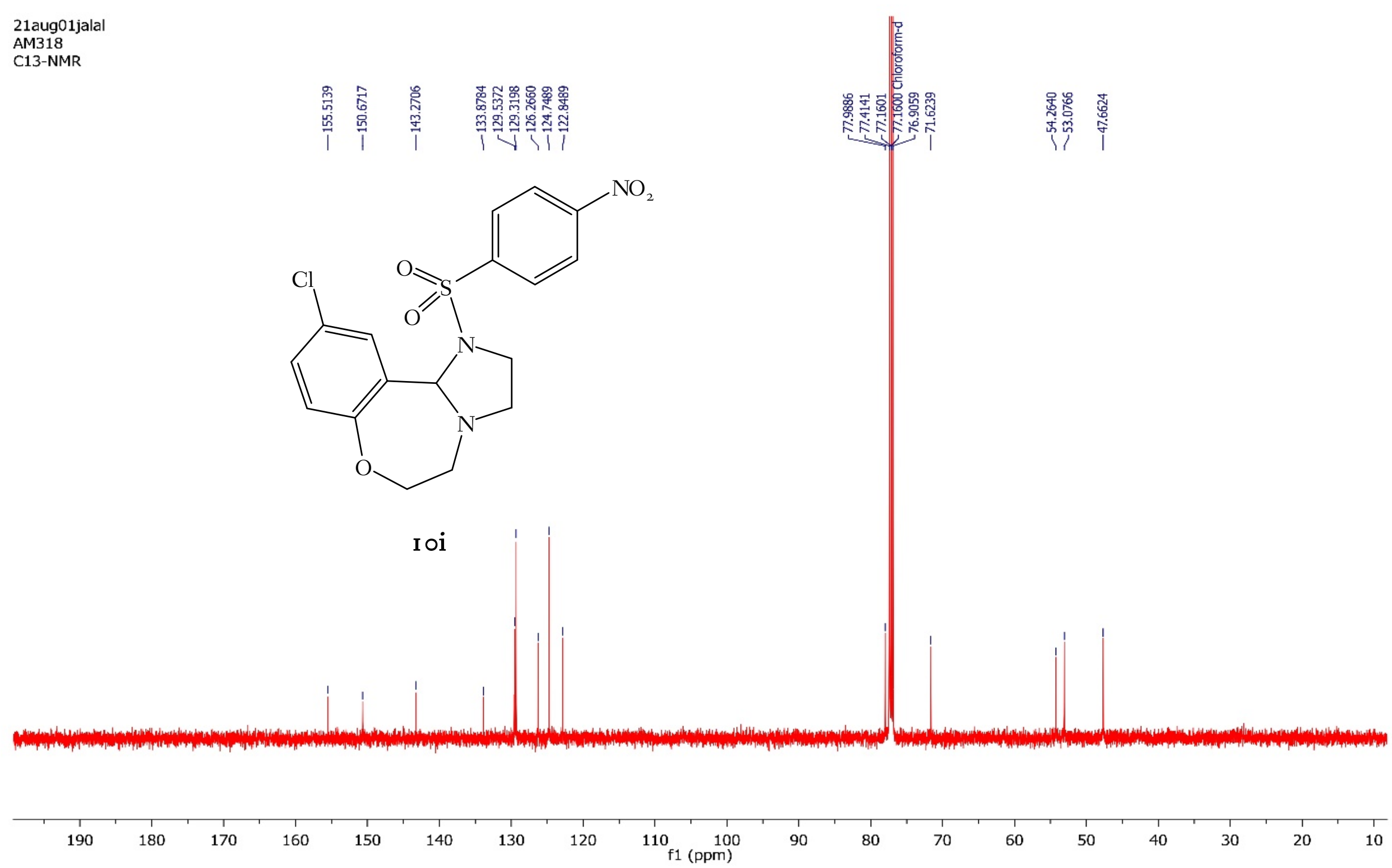


Fig.S56. $^{13}$C NMR spectrum of compound 10i $CDCl_3$

# The University of Jordan
## Faculty of science
## Department of Chemistry

## Mass Spectrum Molecular Formula Report

**Analysis Info**

| | | | |
|---|---|---|---|
| Analysis Name | F:\Data\2022MAR14\Mustafa_000037.d | Acquisition Date | 3/15/2022 12:02:55 PM |
| Method | ESI_pos_20181024 | Operator | Bruker_PC |
| Sample Name | AM318 | Instrument | apex-IV |
| Comment | CHCl3+ACN | | |

**Acquisition Parameter**

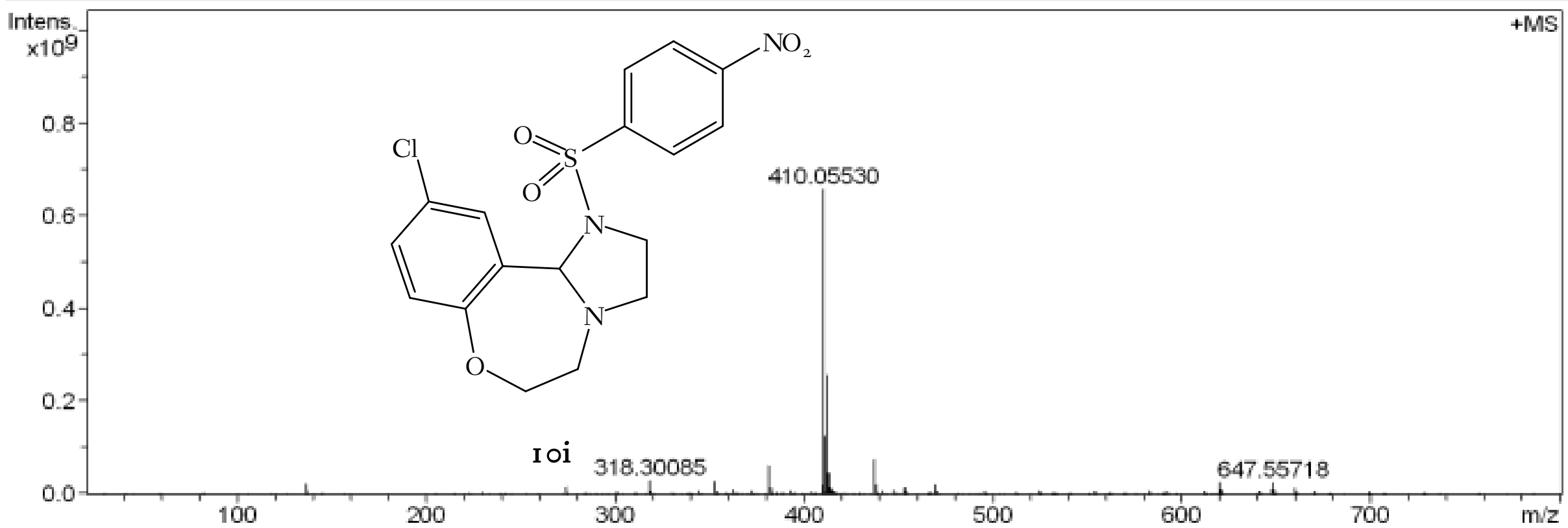


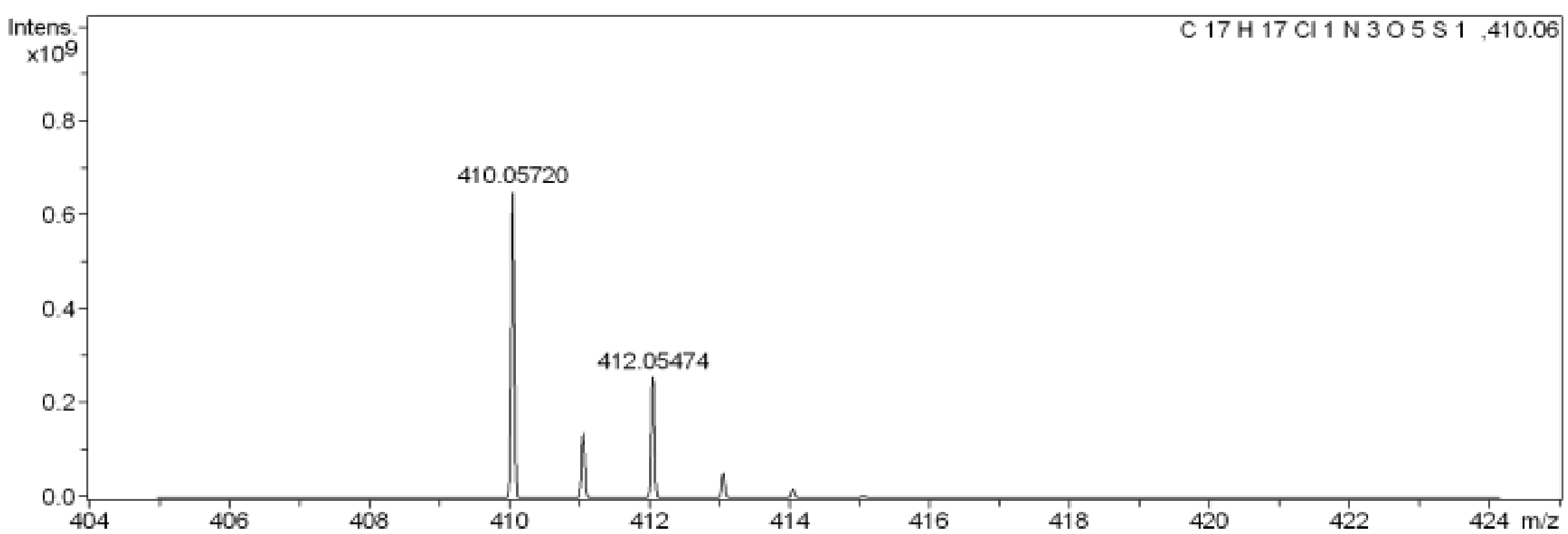


| Sum Formula | Sigma | m/z | Err [ppm] | Mean Err [ppm] | Err [mDa] | rdb | N Rule | e⁻ |
|---|---|---|---|---|---|---|---|---|
| C 17 H 17 Cl 1 N 3 O 5 S 1 | 0.008 | 410.05720 | 4.62 | 3.98 | 1.89 | 10.50 | ok | even |

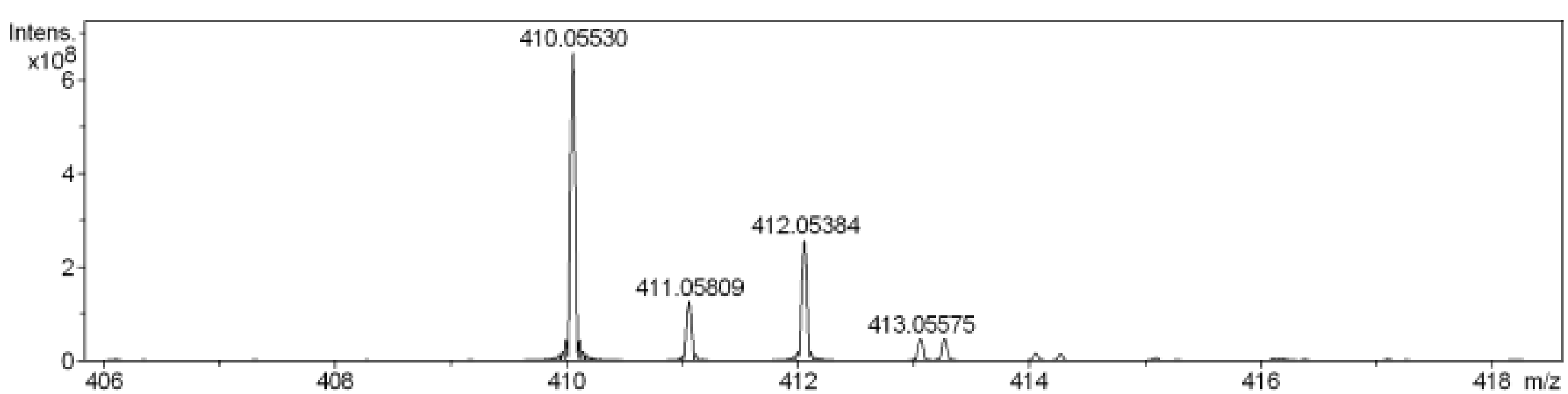


Fig.S57. HRMS spectrum of compound 10i

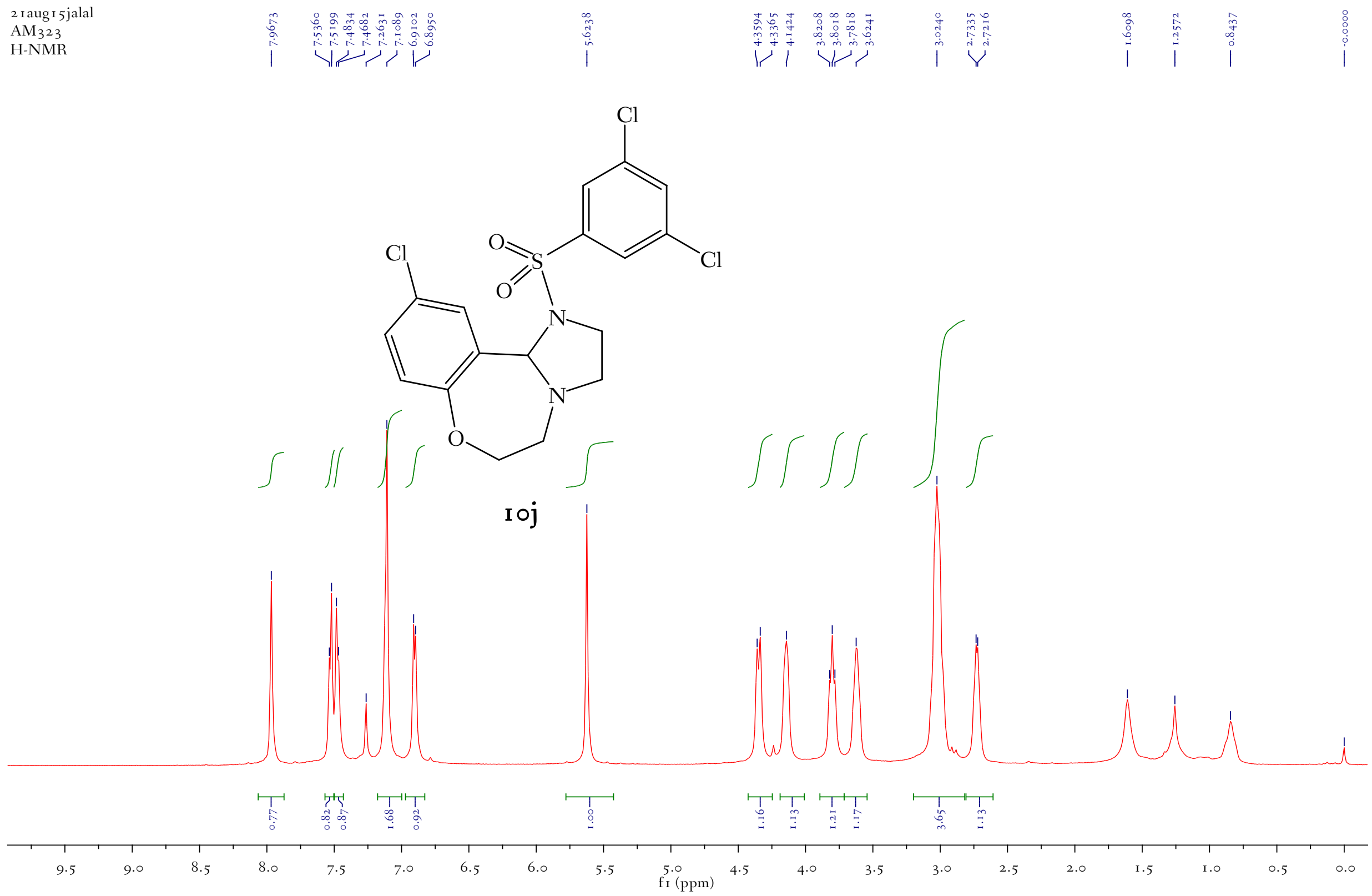


Fig.S58. $^{1}$H NMR spectrum of compound 10j $CDCl_3$

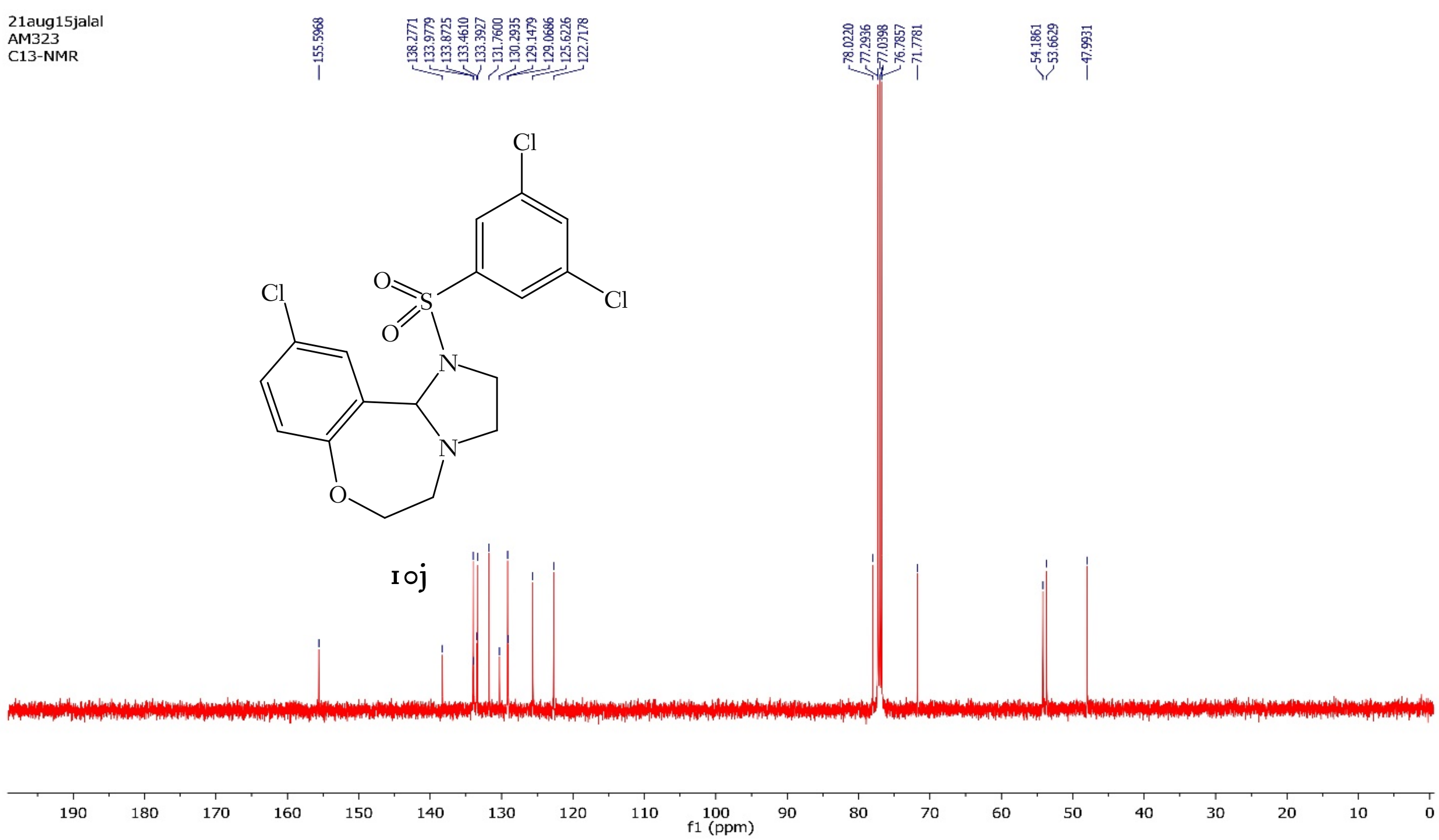


Fig.S59. $^{13}$C NMR spectrum of compound 10j $CDCl_3$

# The University of Jordan
## Faculty of science
## Department of Chemistry

## Mass Spectrum Molecular Formula Report

**Analysis Info**

| | | | |
|---|---|---|---|
| Analysis Name | F:\Data\2022MAR14\Mustafa_000020.d | Acquisition Date | 3/14/2022 3:08:03 PM |
| Method | ESI_pos_20181024 | Operator | Bruker_PC |
| Sample Name | AM323 | Instrument | apex-IV |
| Comment | CHCl3+ACN | | |

**Acquisition Parameter**

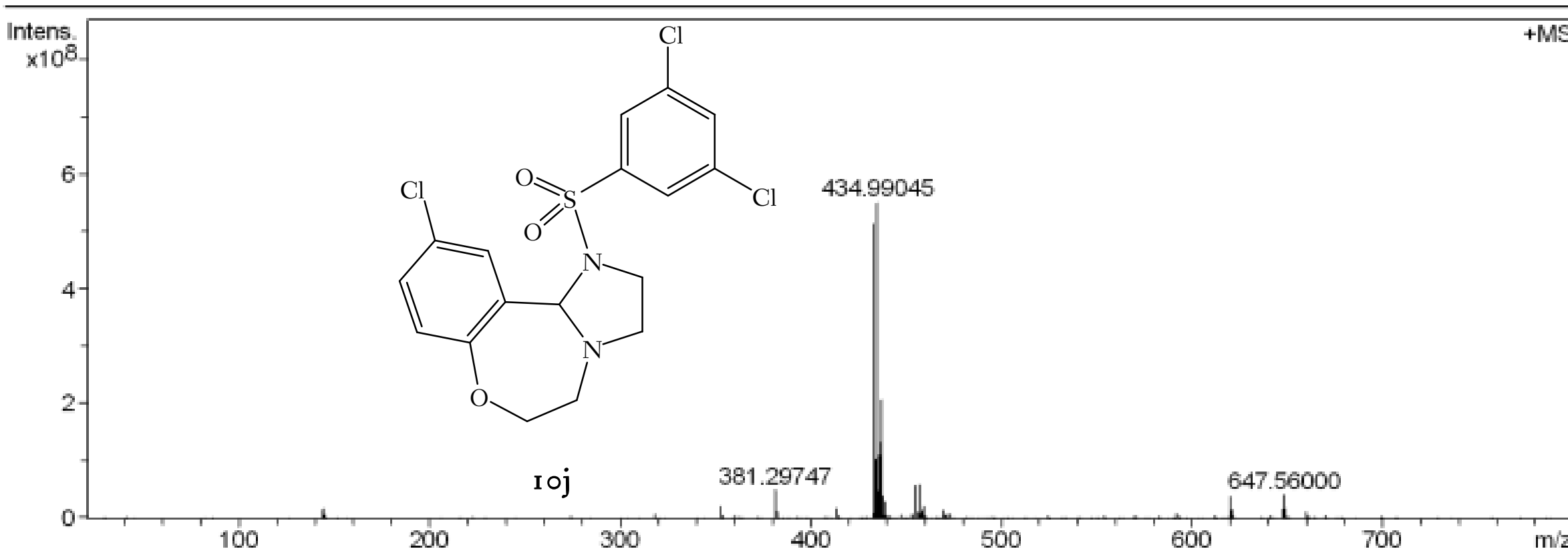


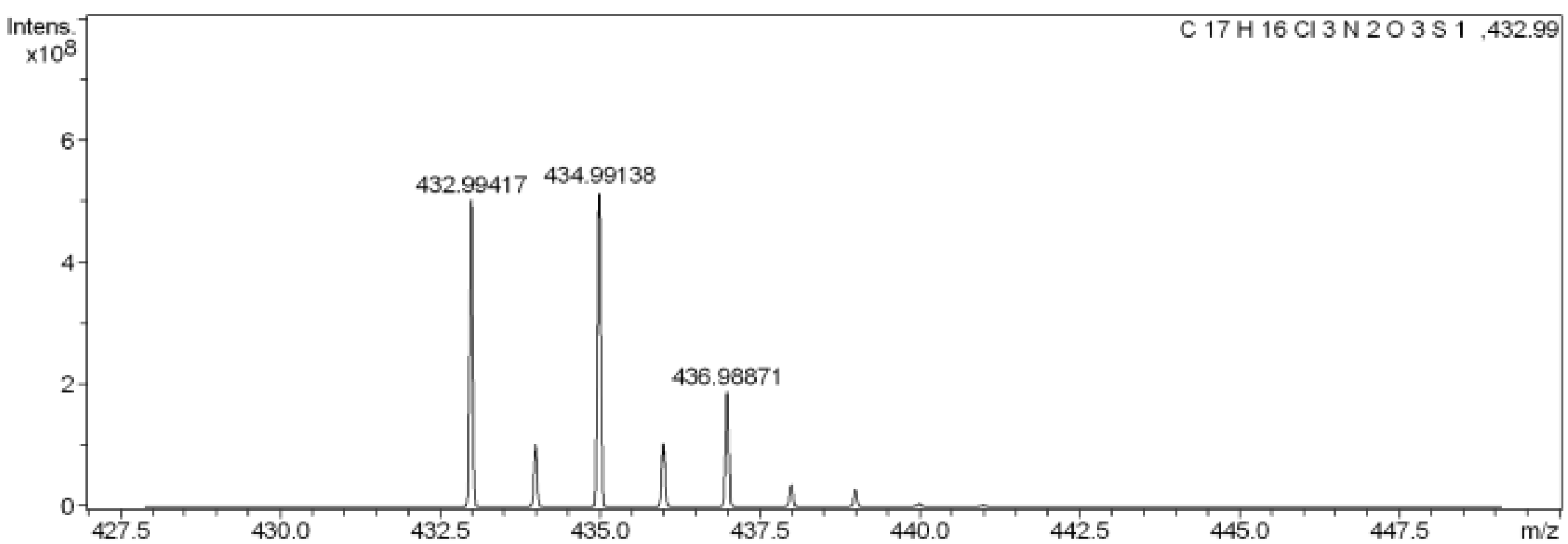


| Sum Formula | Sigma | m/z | Err [ppm] | Mean Err [ppm] | Err [mDa] | rdb | N Rule | $e^-$ |
|---|---|---|---|---|---|---|---|---|
| C 17 H 16 Cl 3 N 2 O 3 S 1 | 0.011 | 432.99417 | 4.22 | 2.56 | 1.83 | 9.50 | ok | even |

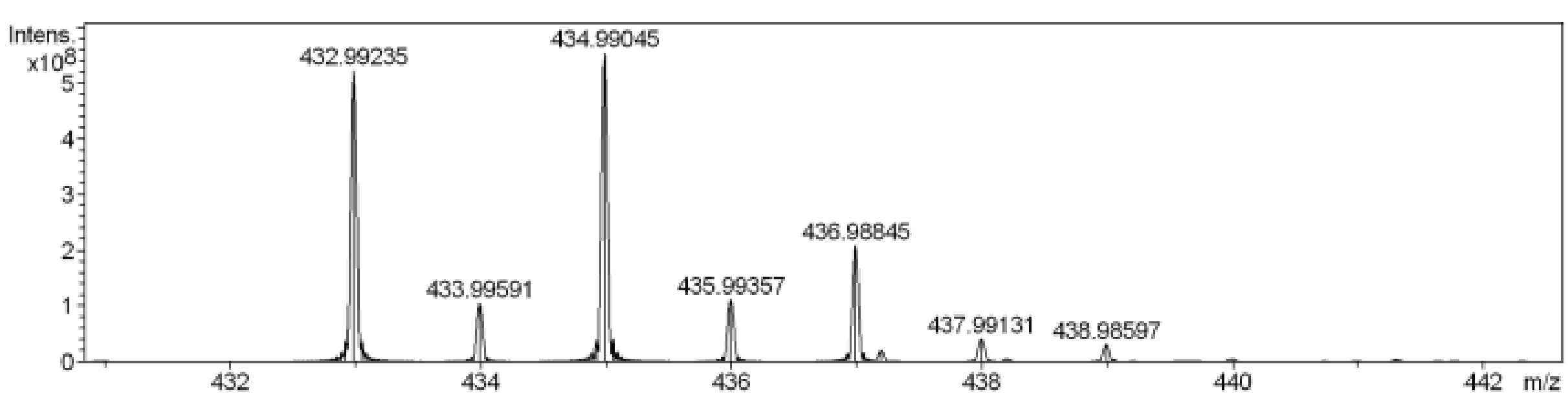


Fig.S60. HRMS spectrum of compound 10j

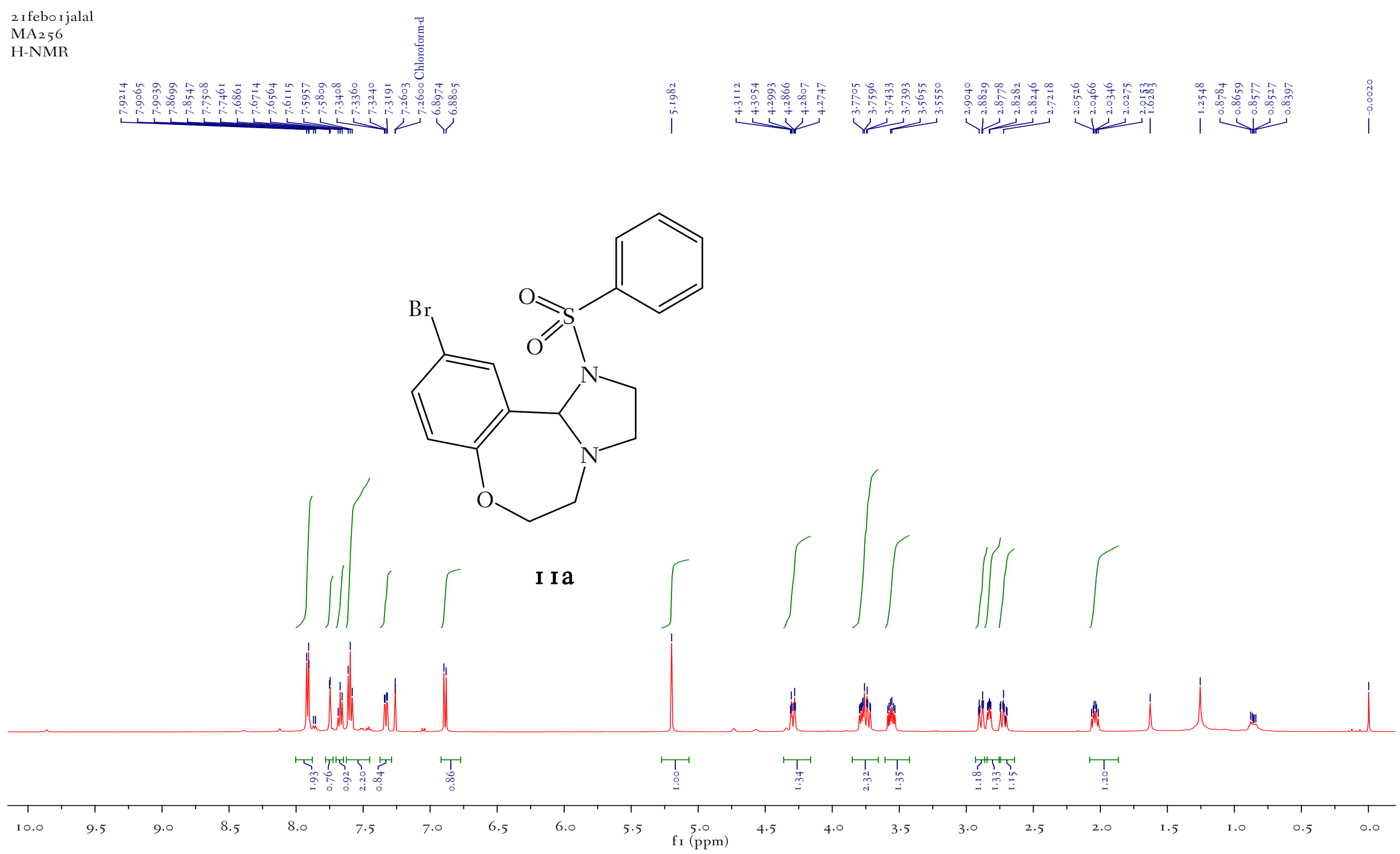


Fig.S61. $^{1}$H NMR spectrum of compound 11a $CDCl_3$

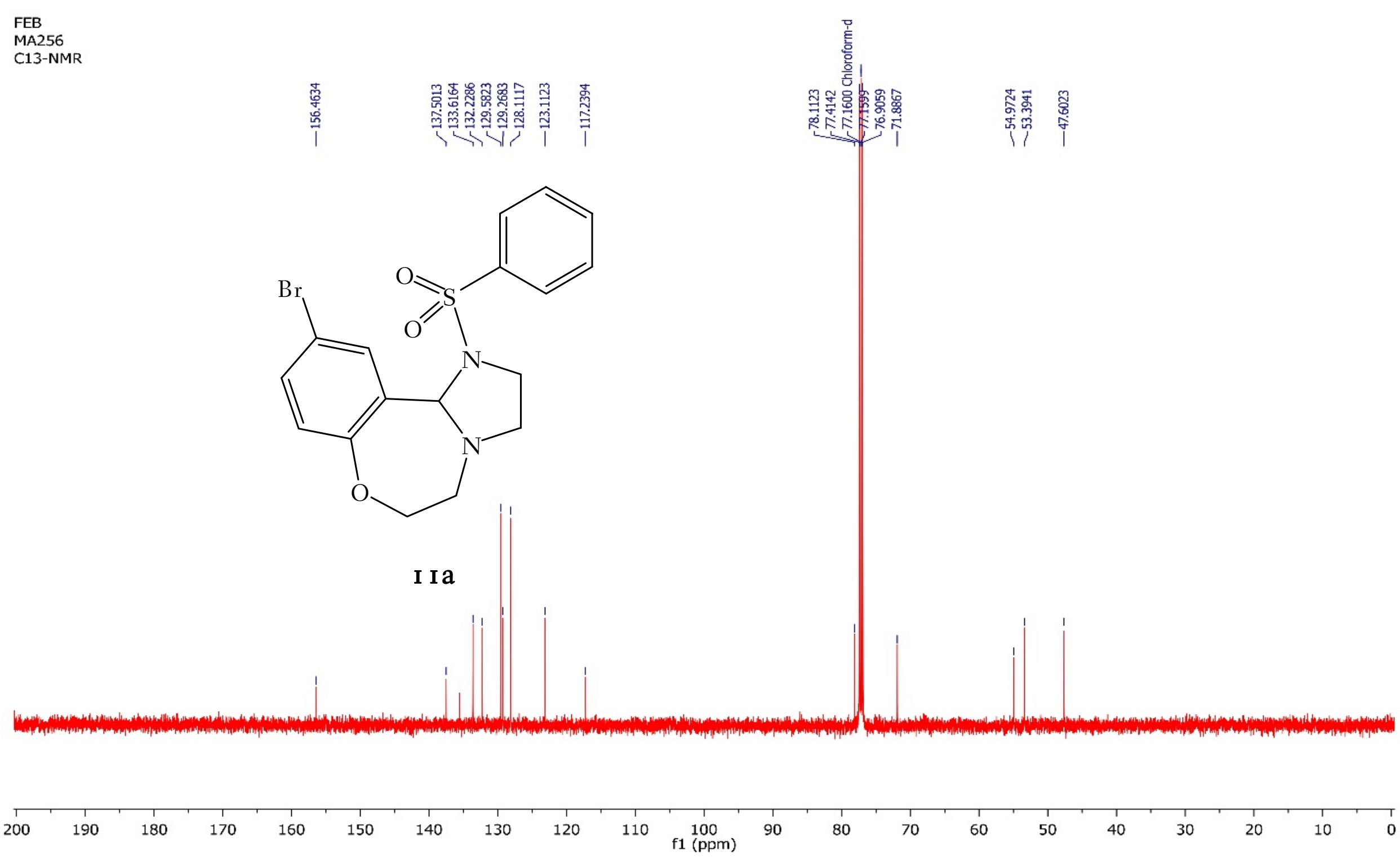


Fig.S62. $^{13}$C NMR spectrum of compound 11a $CDCl_3$

**The University of Jordan**

**Faculty of science**

**Department of Chemistry**

## Mass Spectrum Molecular Formula Report

**Analysis Info**

| | | | |
|---|---|---|---|
| Analysis Name | F:\Data\2022MAR20\Mustafa_000043.d | Acquisition Date | 3/23/2022 1:42:39 PM |
| Method | ESI_pos_20181024 | Operator | Bruker_PC |
| Sample Name | AM256 | Instrument | apex-IV |
| Comment | CHCl3+ACN | | |

**Acquisition Parameter**

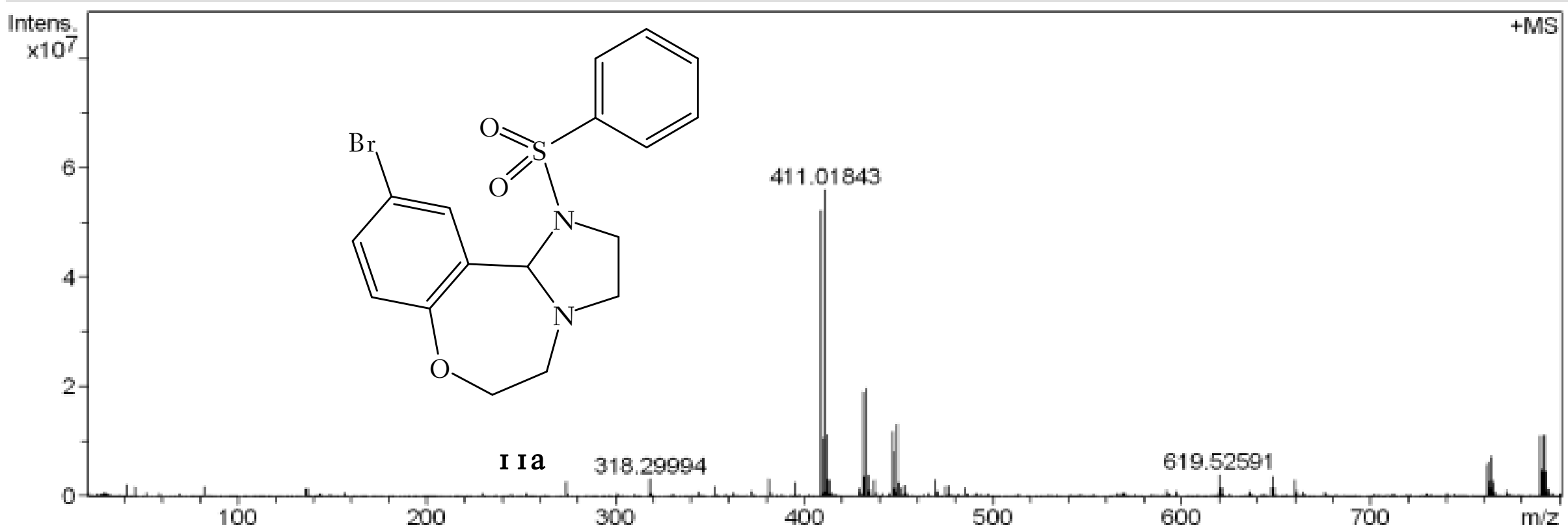


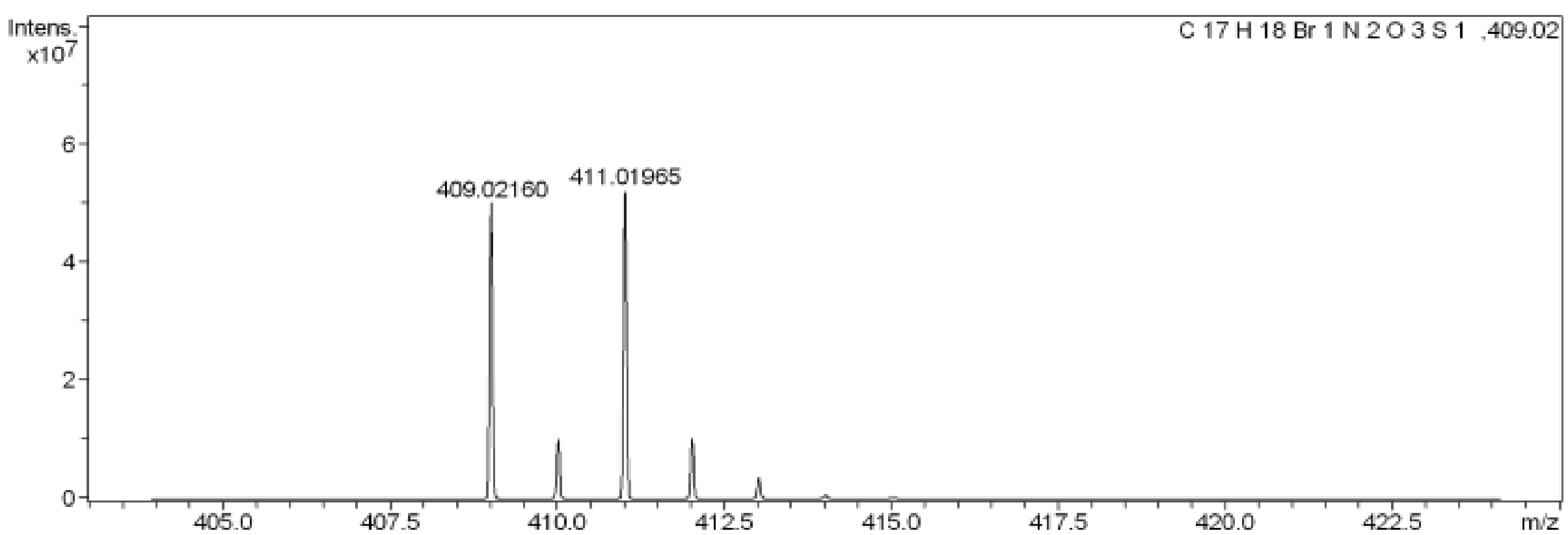


| Sum Formula | Sigma | m/z | Err [ppm] | Mean Err [ppm] | Err [mDa] | rdb | N Rule | e⁻ |
|---|---|---|---|---|---|---|---|---|
| C 17 H 18 Br 1 N 2 O 3 S 1 | 0.012 | 409.02160 | 2.73 | 2.77 | 1.12 | 9.50 | ok | even |

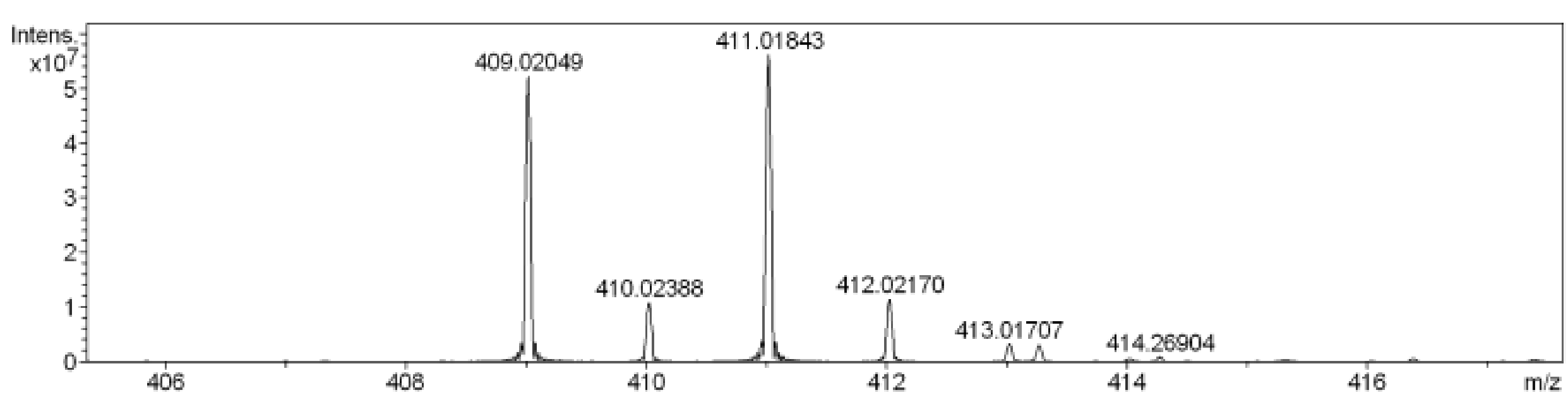


Fig.S63. HRMS spectrum of compound 11a

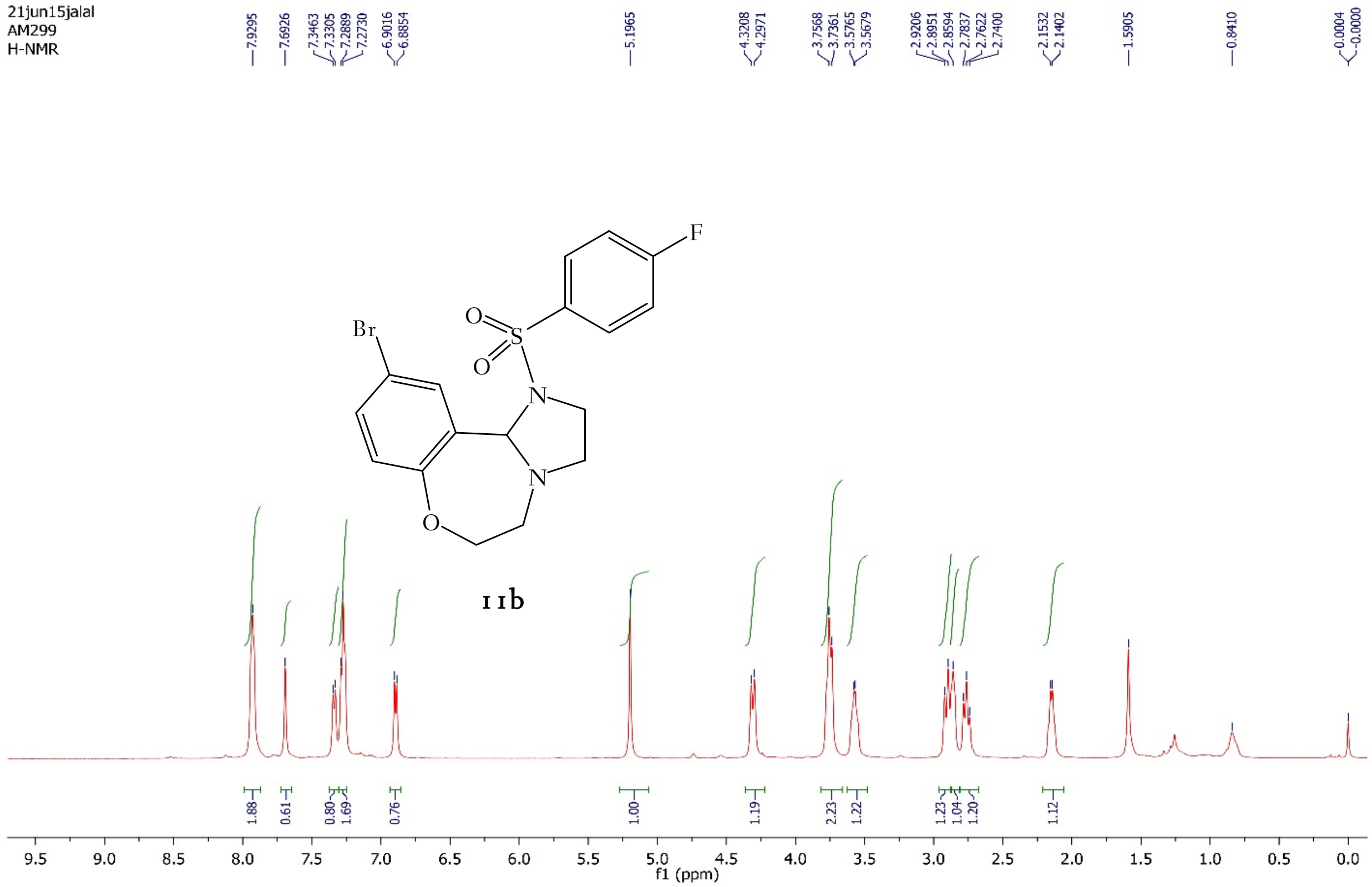


Fig.S64. $^{1}$H NMR spectrum of compound 11b $CDCl_3$

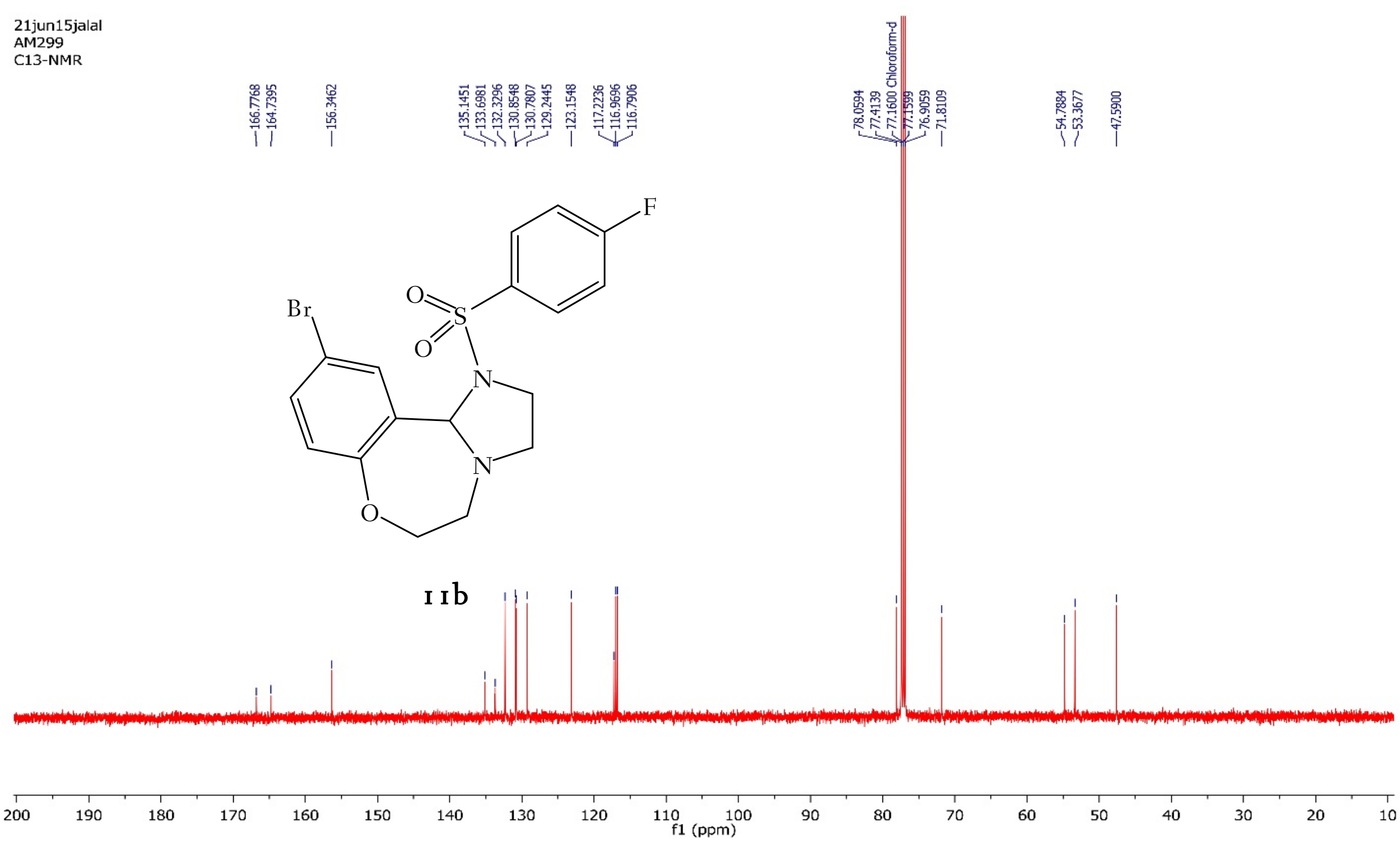


Fig.S65. $^{13}$C NMR spectrum of compound 11b $CDCl_3$

# The University of Jordan
## Faculty of science
## Department of Chemistry

# Mass Spectrum Molecular Formula Report

**Analysis Info**

| | | | |
|---|---|---|---|
| Analysis Name | F:\Data\2022MAR20\Mustafa_000057.d | Acquisition Date | 3/23/2022 2:23:28 PM |
| Method | ESI_pos_20181024 | Operator | Bruker_PC |
| Sample Name | AM299 | Instrument | apex-IV |
| Comment | CHCl3+ACN | | |

**Acquisition Parameter**

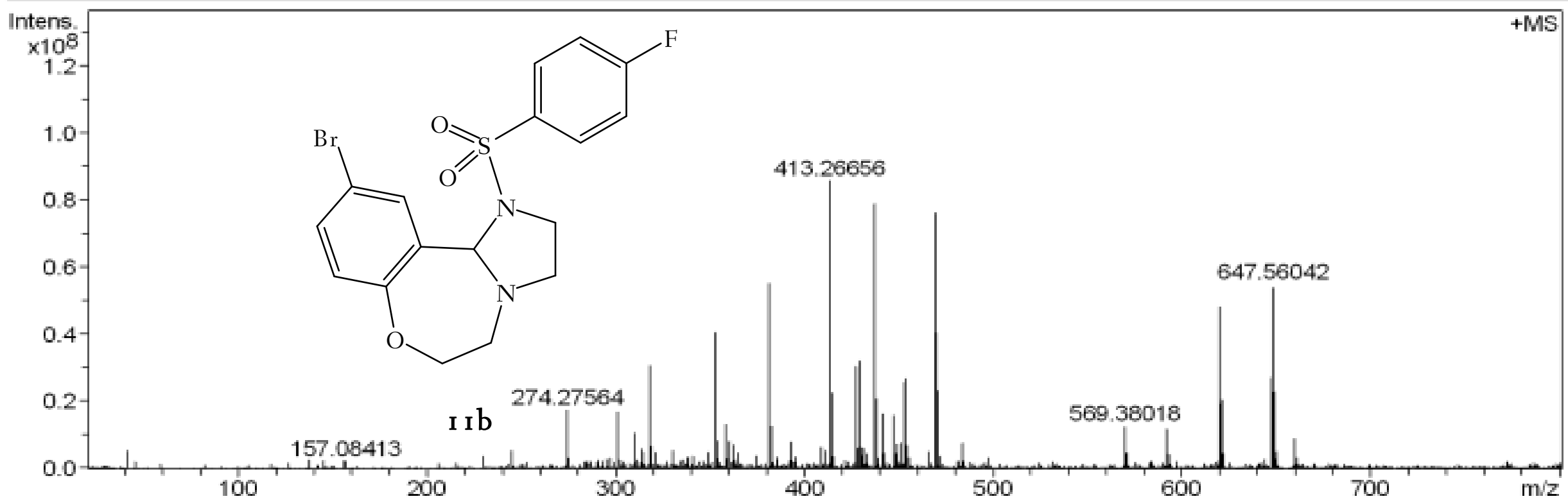


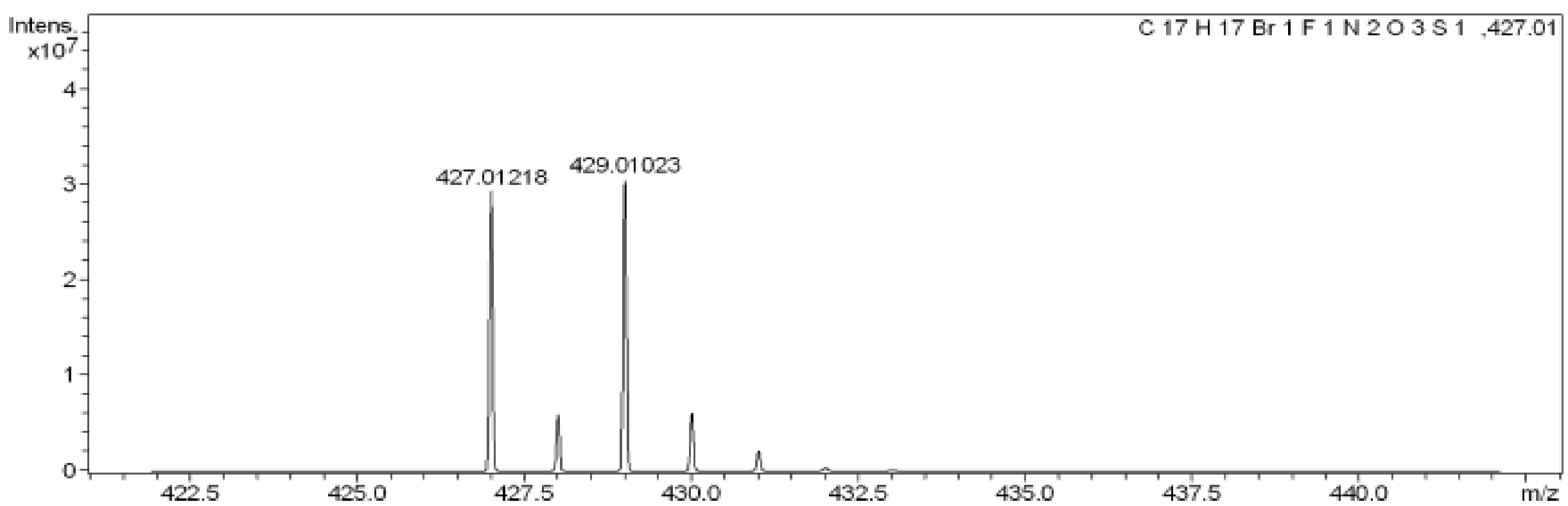


| Sum Formula | Sigma | m/z | Err [ppm] | Mean Err [ppm] | Err [mDa] | rdb | N Rule | e⁻ |
|---|---|---|---|---|---|---|---|---|
| C 17 H 17 Br 1 F 1 N 2 O 3 S 1 | 0.050 | 427.01218 | -0.54 | -0.55 | -0.23 | 9.50 | ok | even |

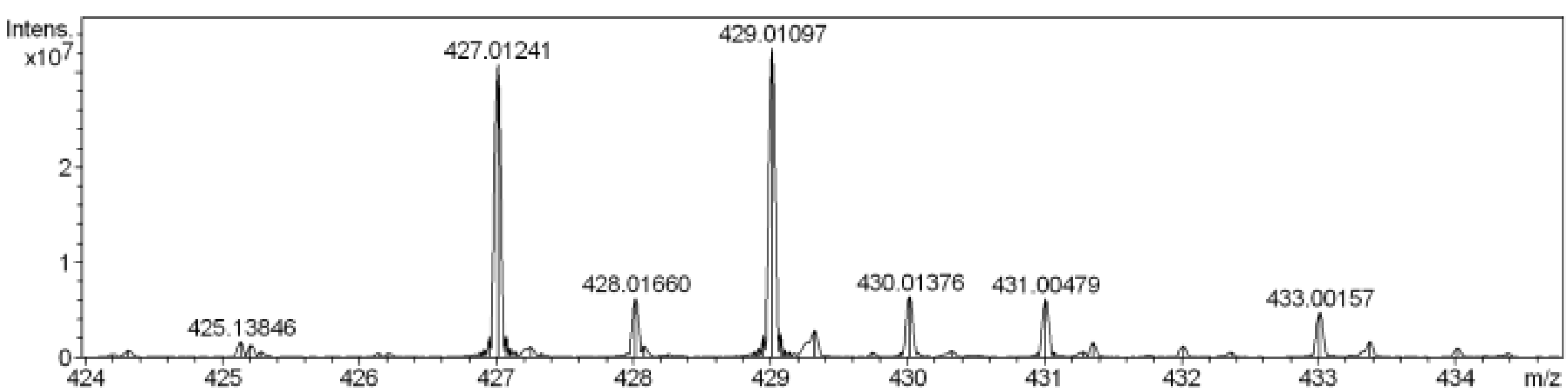

Fig.S66. HRMS spectrum of compound 11b

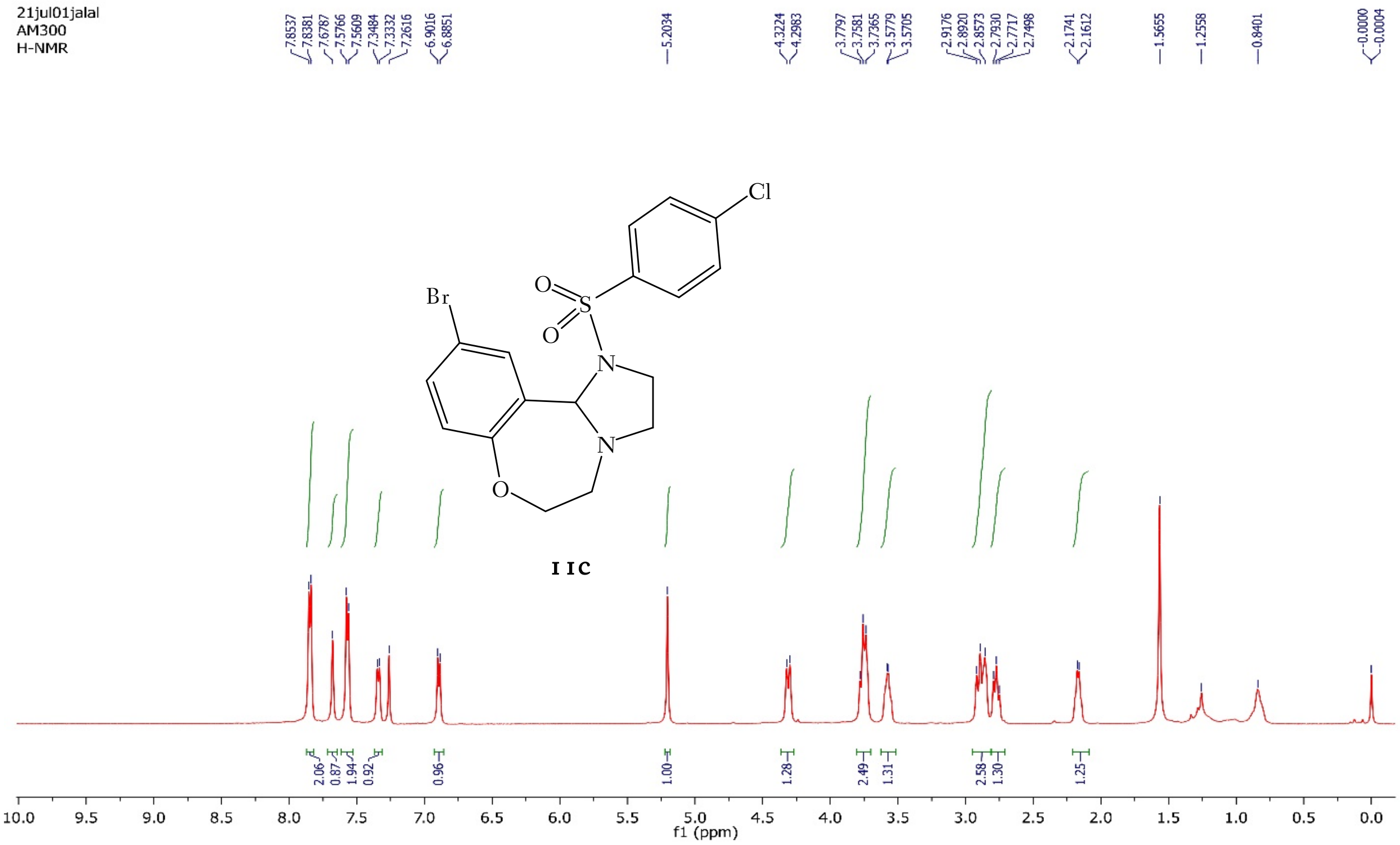

Fig.S67. ¹H NMR spectrum of compound 11c $CDCl_3$

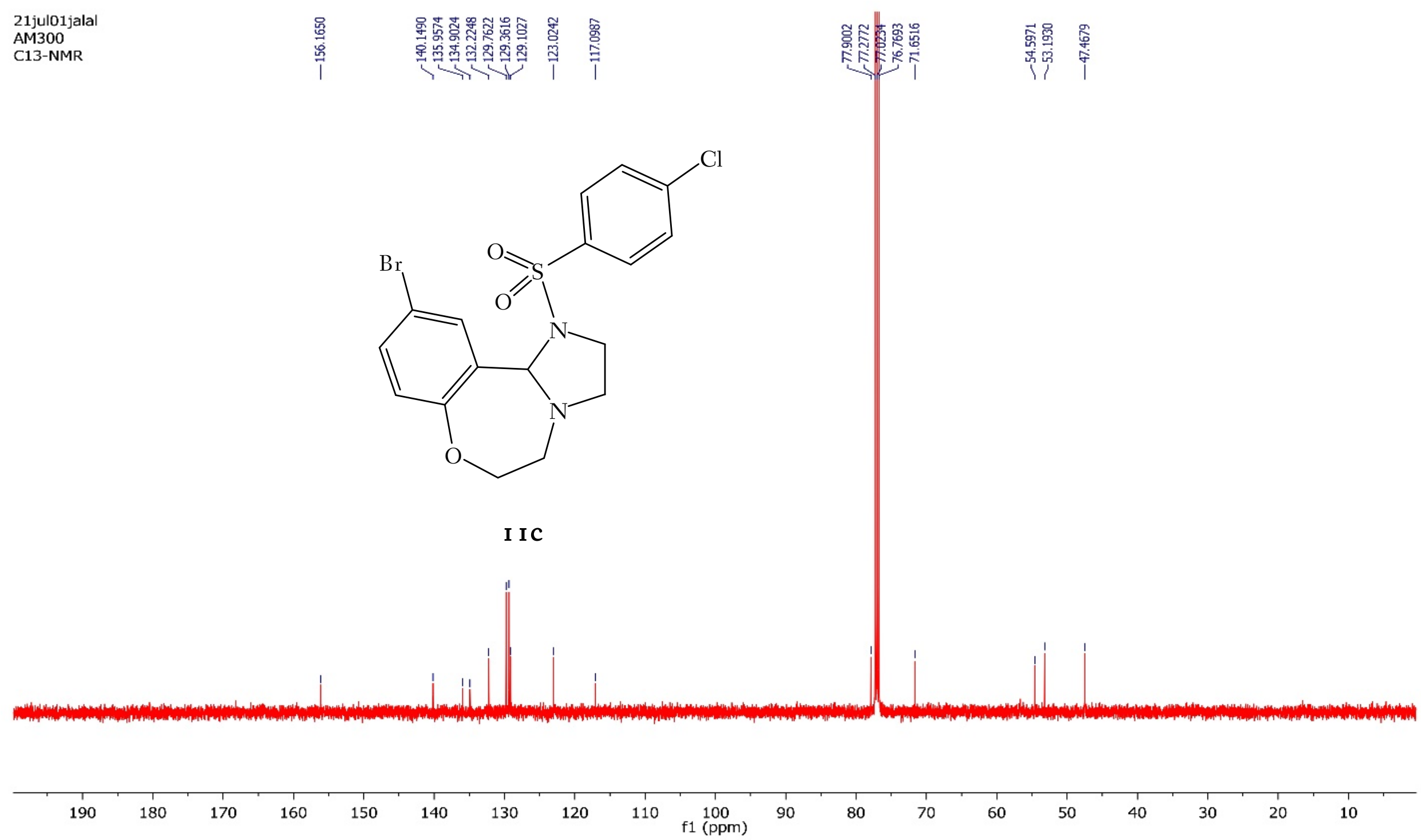

Fig.S68. ¹³C NMR spectrum of compound 11c $CDCl_3$

# The University of Jordan
## Faculty of science
## Department of Chemistry

## Mass Spectrum Molecular Formula Report

**Analysis Info**

| | | | |
|---|---|---|---|
| Analysis Name | F:\Data\2022MAR08\Mustafa_000031.d | Acquisition Date | 3/13/2022 11:30:09 AM |
| Method | ESI_pos_20181024 | Operator | Bruker_PC |
| Sample Name | AM300 | Instrument | apex-IV |
| Comment | CHCl3+ACN | | |

**Acquisition Parameter**

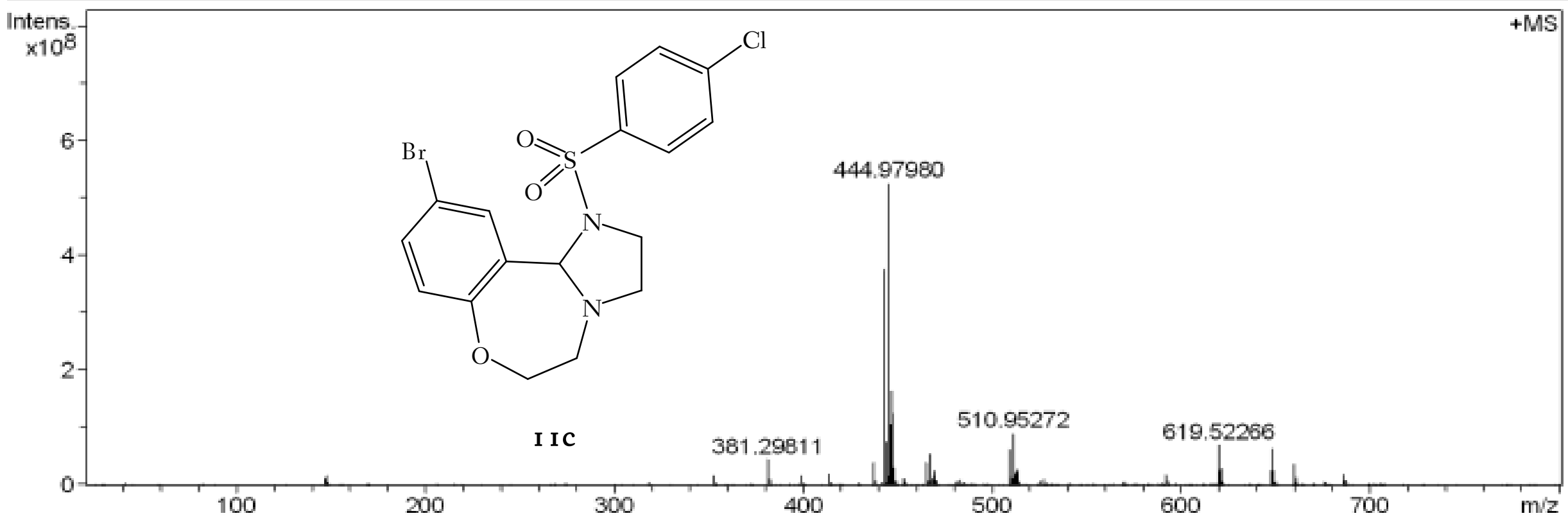


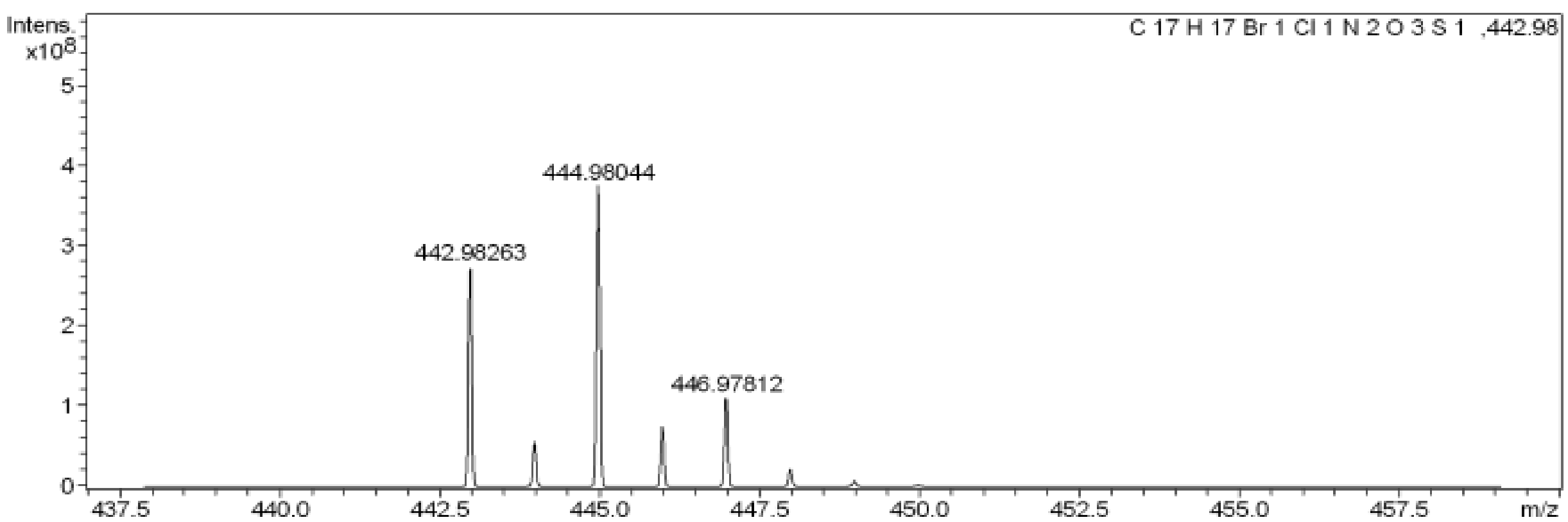


| Sum Formula | Sigma | m/z | Err [ppm] | Mean Err [ppm] | Err [mDa] | rdb | N Rule | e⁻ |
|---|---|---|---|---|---|---|---|---|
| C 17 H 17 Br 1 Cl 1 N 2 O 3 S 1 | 0.010 | 442.98263 | 2.23 | 1.22 | 0.99 | 9.50 | ok | even |

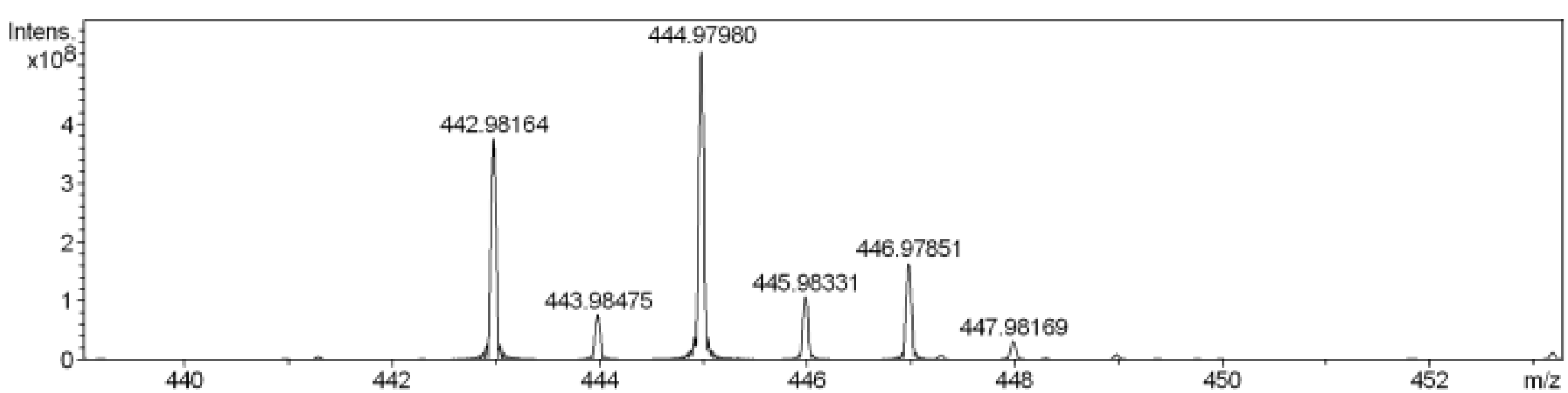


Fig.S69. HRMS spectrum of compound 11c

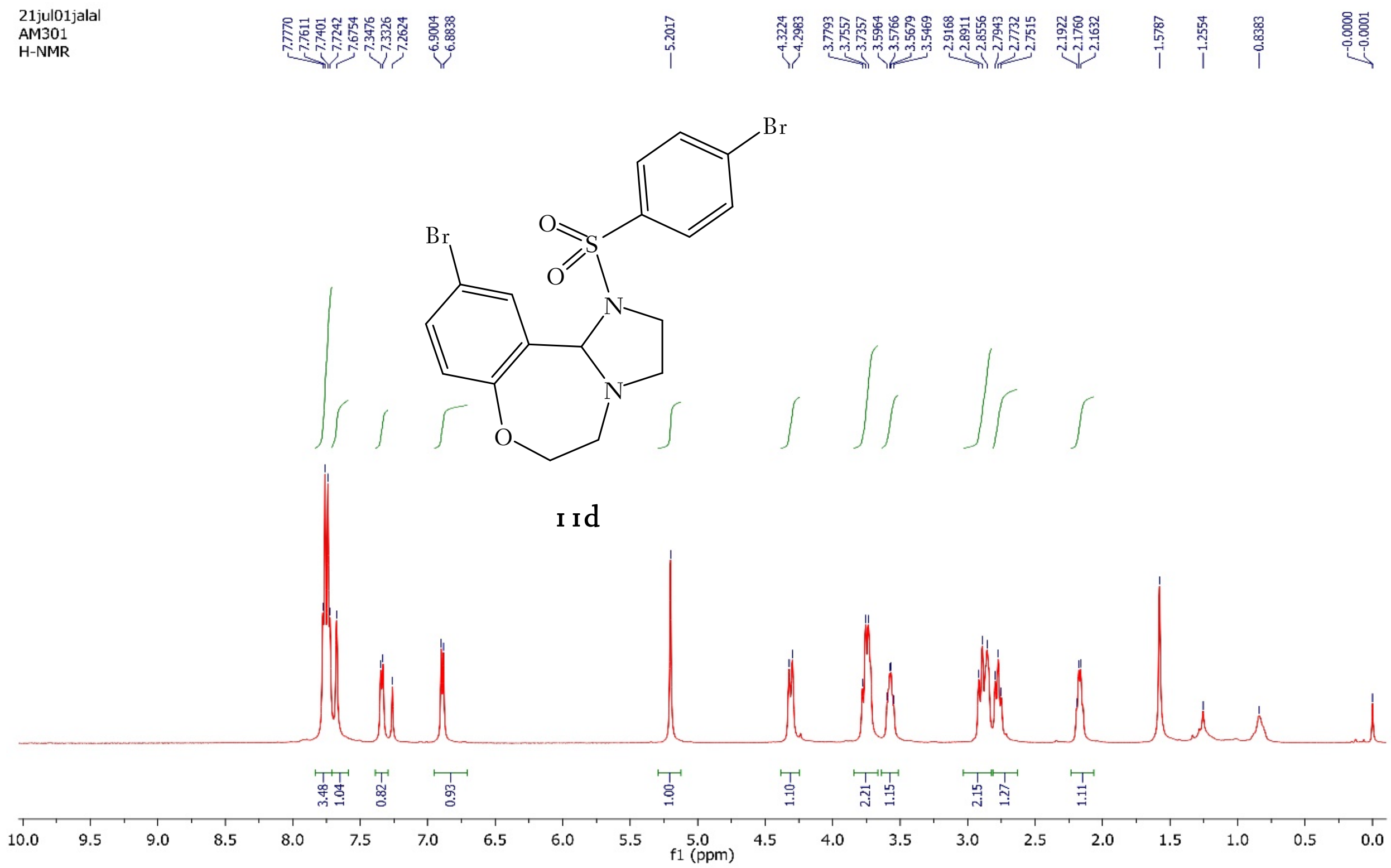


Fig.S70. ¹H NMR spectrum of compound 11d $CDCl_3$

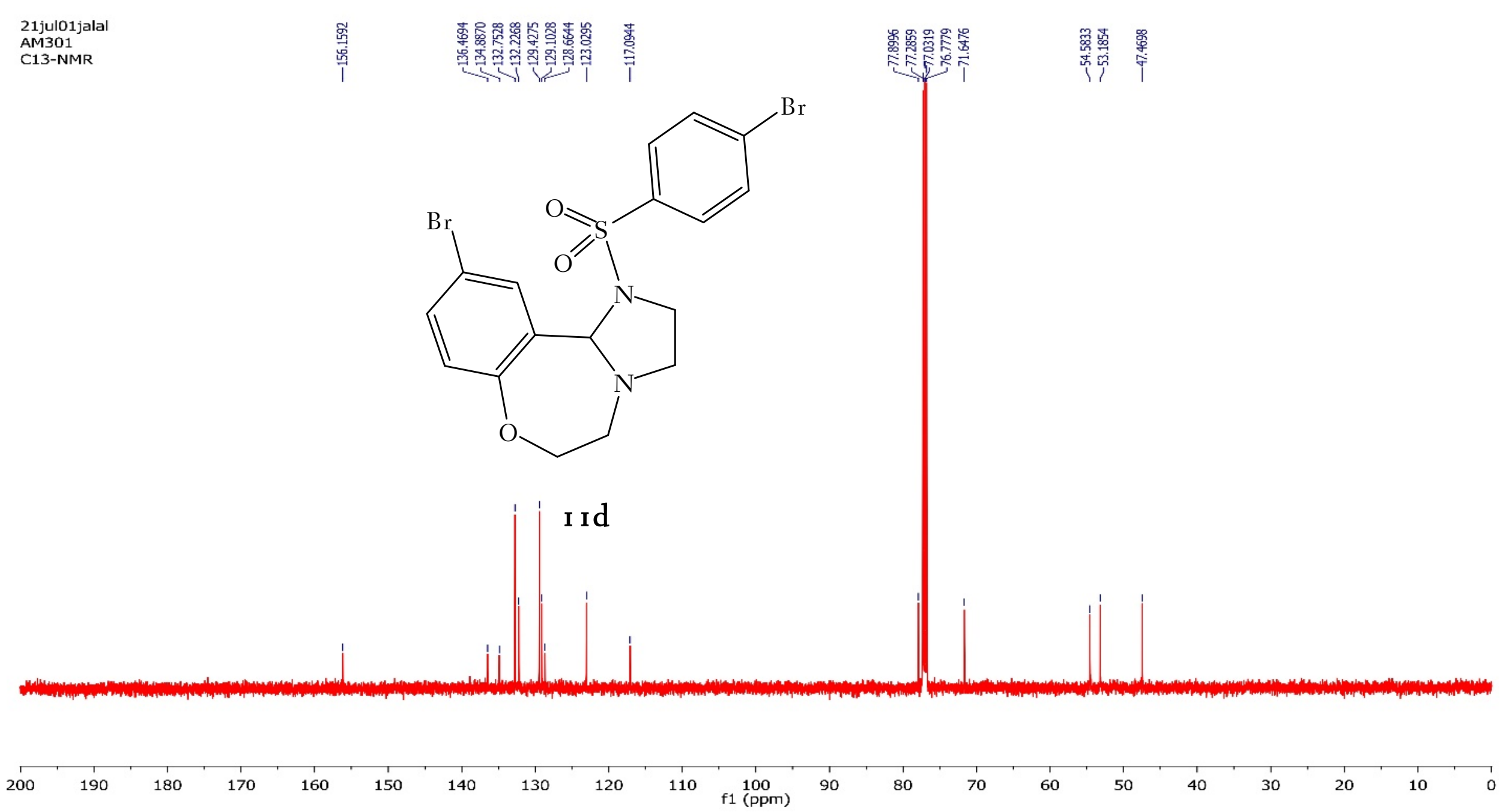


Fig.S71. ¹³C NMR spectrum of compound 11d $CDCl_3$

# The University of Jordan
## Faculty of science
## Department of Chemistry

## Mass Spectrum Molecular Formula Report

**Analysis Info**

| | | | |
|---|---|---|---|
| Analysis Name | F:\Data\2022MAR08\Mustafa_000032.d | Acquisition Date | 3/13/2022 11:34:36 AM |
| Method | ESI_pos_20181024 | Operator | Bruker_PC |
| Sample Name | AM301 | Instrument | apex-IV |
| Comment | CHCl3+ACN | | |

**Acquisition Parameter**

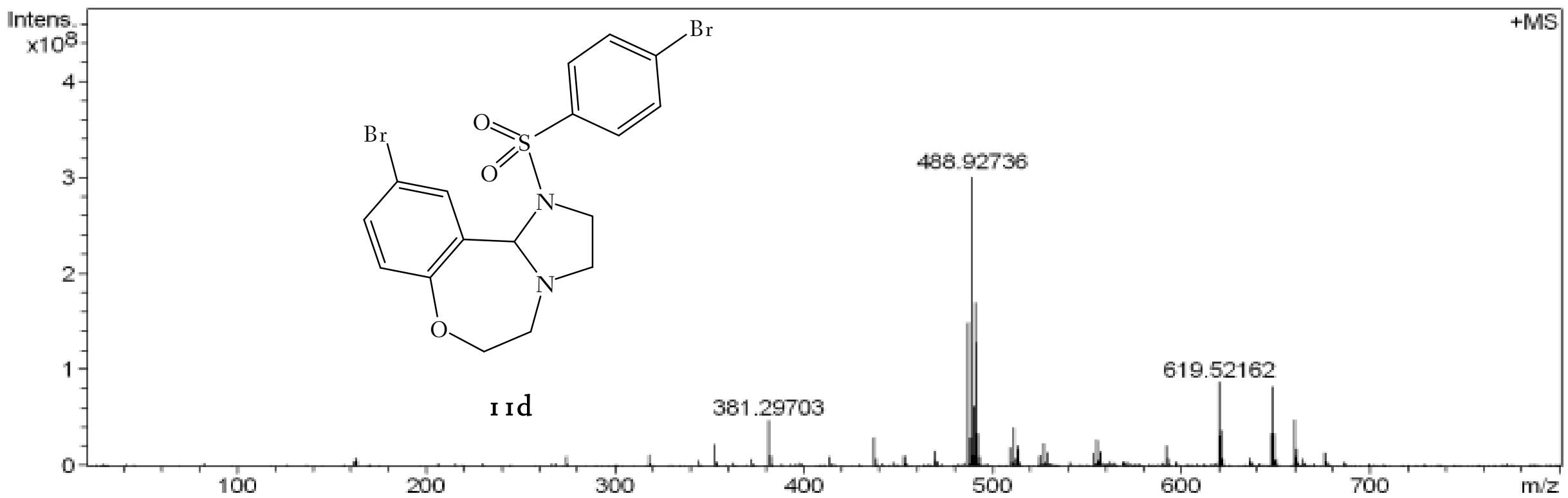


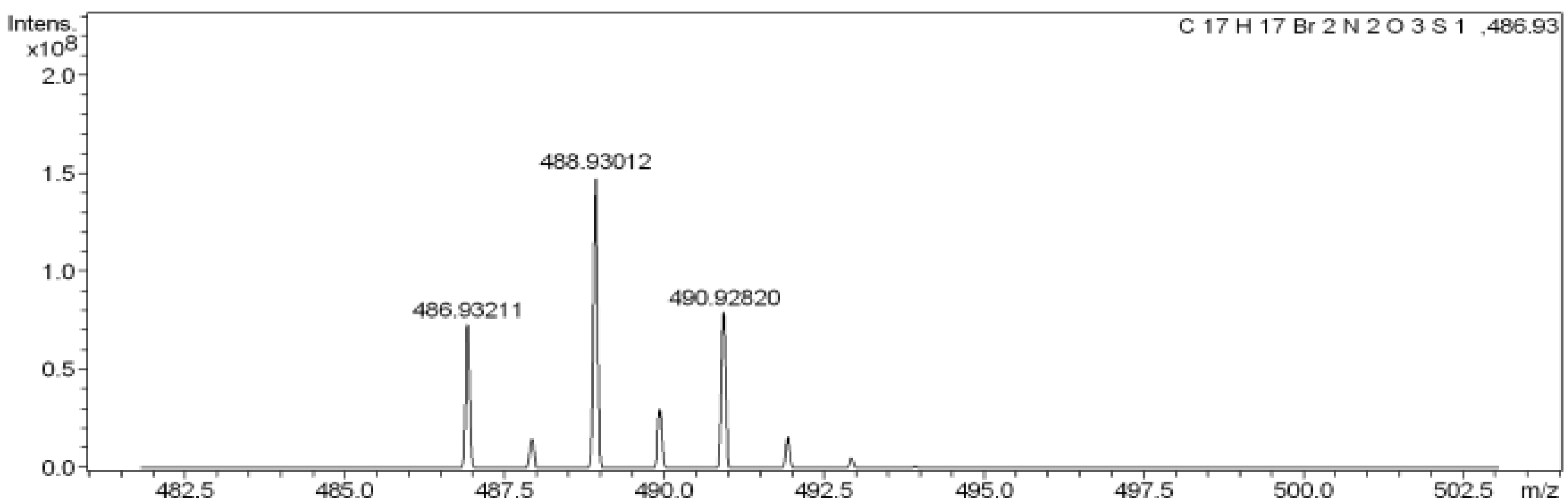


| Sum Formula | Sigma | m/z | Err [ppm] | Mean Err [ppm] | Err [mDa] | rdb | N Rule | e⁻ |
|---|---|---|---|---|---|---|---|---|
| C 17 H 17 Br 2 N 2 O 3 S 1 | 0.009 | 486.93211 | 3.94 | 4.32 | 1.92 | 9.50 | ok | even |

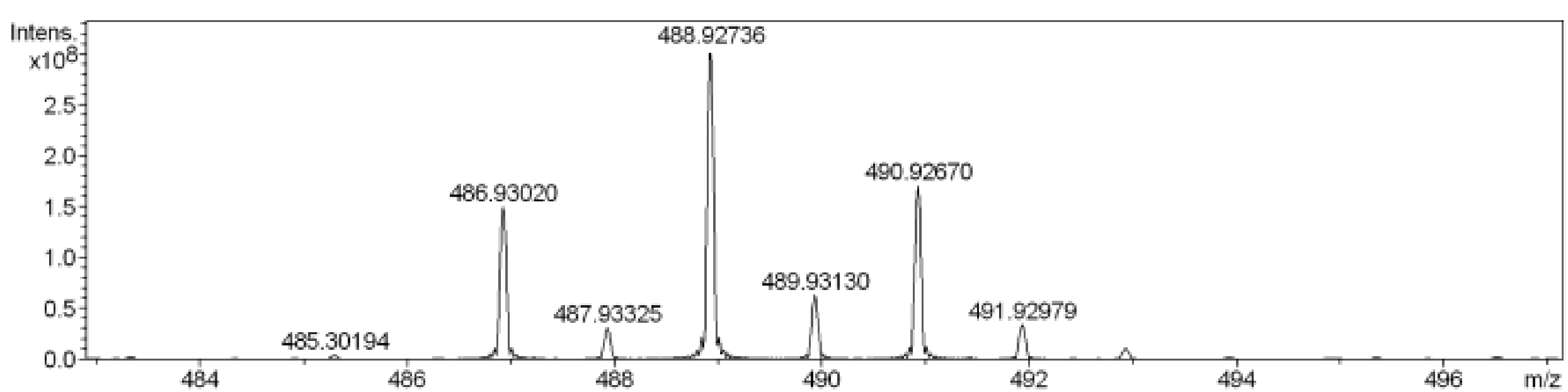


Fig.S72. HRMS spectrum of compound 11d

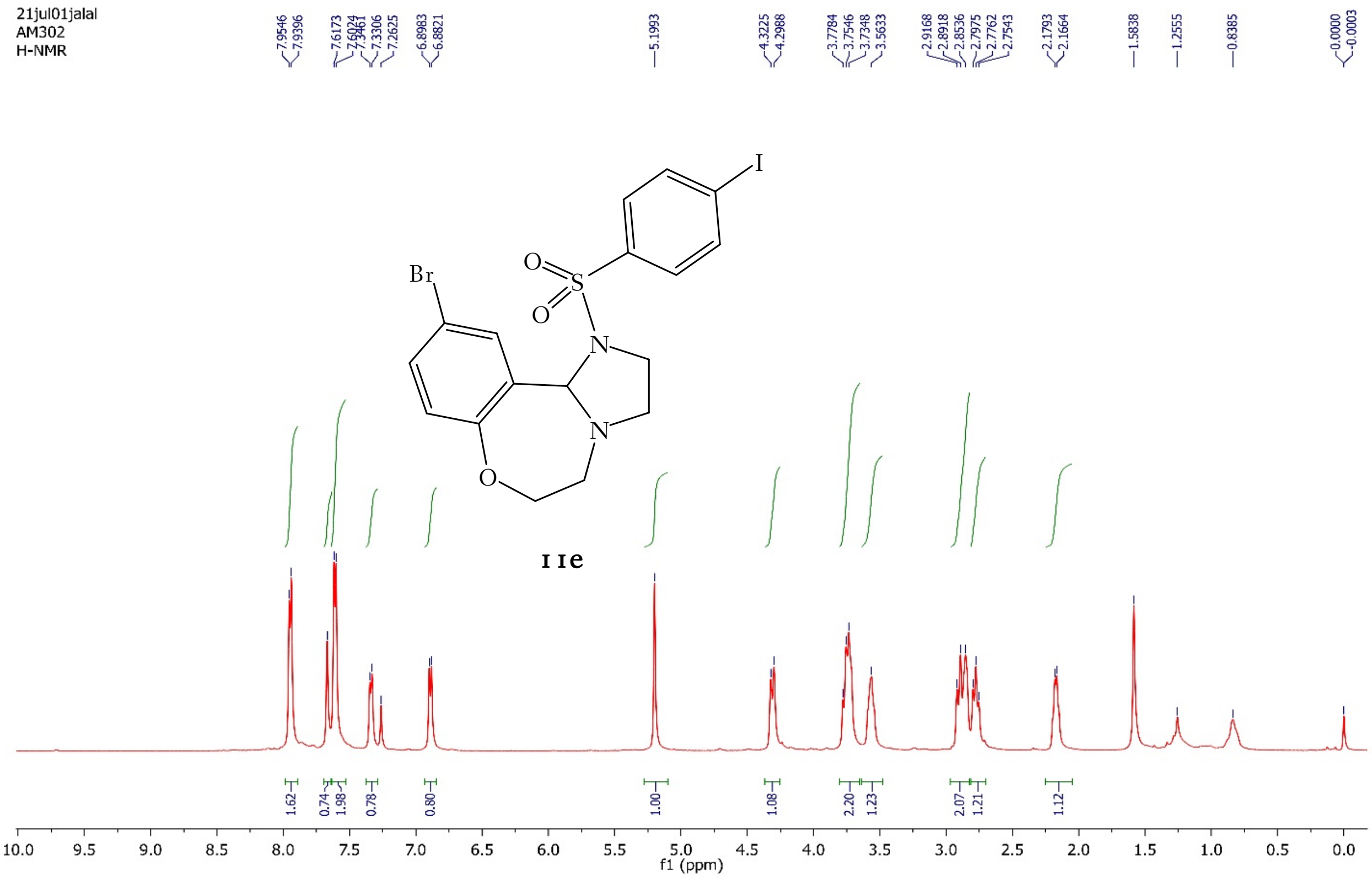


Fig.S73. $^{1}$H NMR spectrum of compound 11e $CDCl_3$

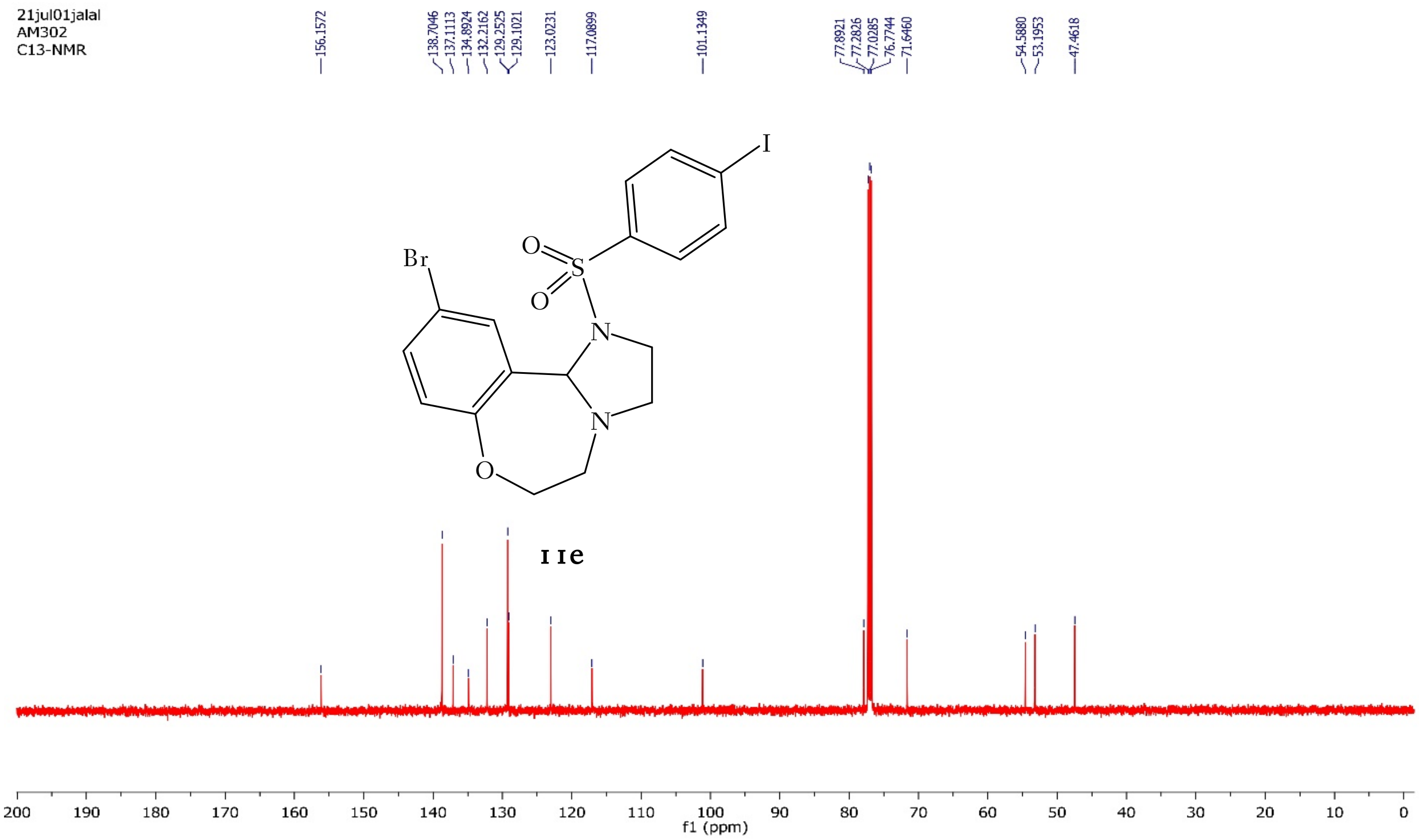


Fig.S74. $^{13}$C NMR spectrum of compound 11e $CDCl_3$

# The University of Jordan
## Faculty of science
## Department of Chemistry

## Mass Spectrum Molecular Formula Report

**Analysis Info**

| | | | |
|---|---|---|---|
| Analysis Name | F:\Data\2022MAR30\Mustafa_000008.d | Acquisition Date | 3/30/2022 11:00:59 AM |
| Method | ESI_pos_20181024 | Operator | Bruker_PC |
| Sample Name | AM302 | Instrument | apex-IV |
| Comment | CHCl3+ACN | | |

**Acquisition Parameter**

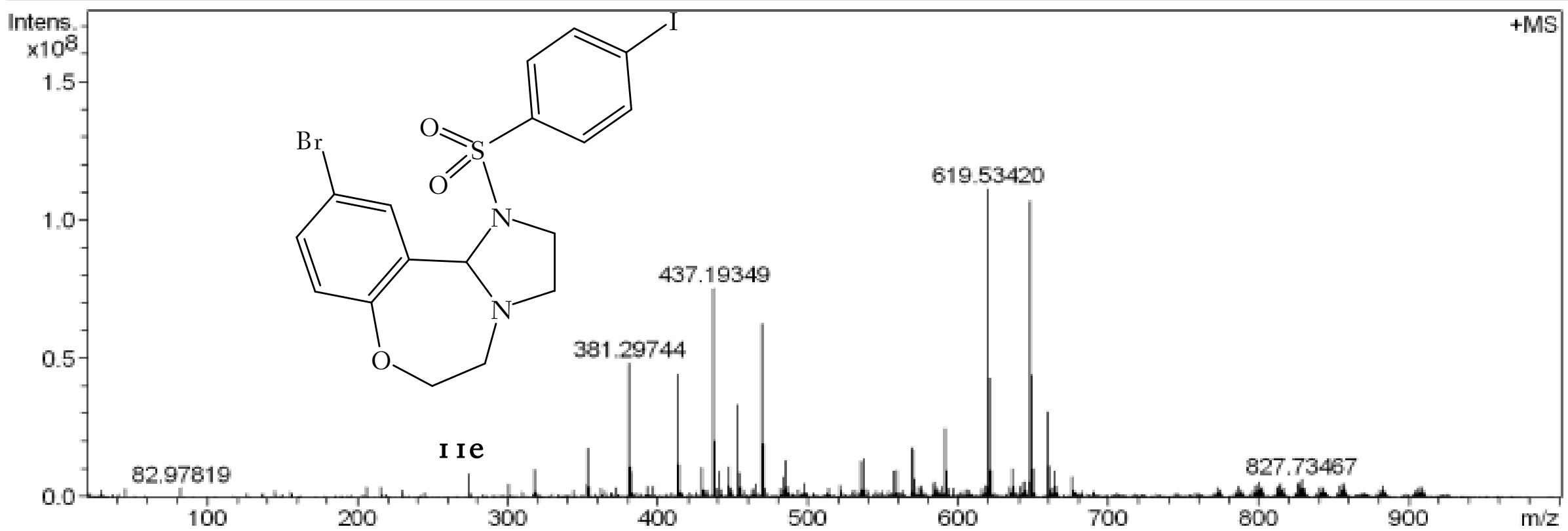


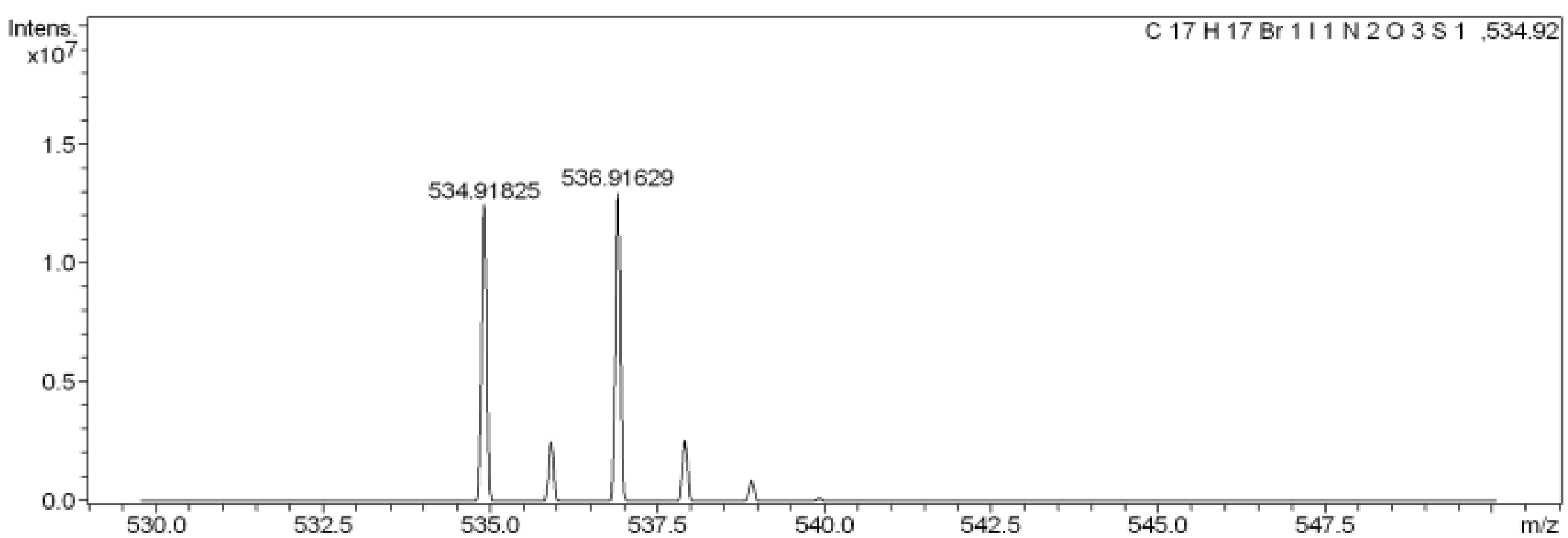


| Sum Formula | Sigma | m/z | Err [ppm] | Mean Err [ppm] | Err [mDa] | rdb | N Rule | e⁻ |
|---|---|---|---|---|---|---|---|---|
| C 17 H 17 Br 1 I 1 N 2 O 3 S 1 | 0.014 | 534.91825 | -3.55 | -5.52 | -1.90 | 9.50 | ok | even |

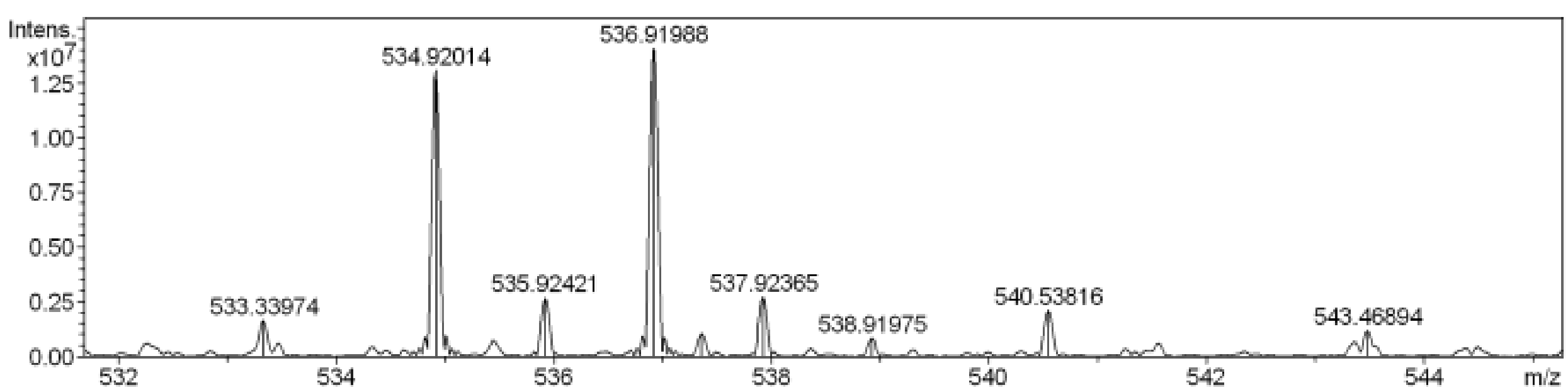


Fig.S75. HRMS spectrum of compound 11e

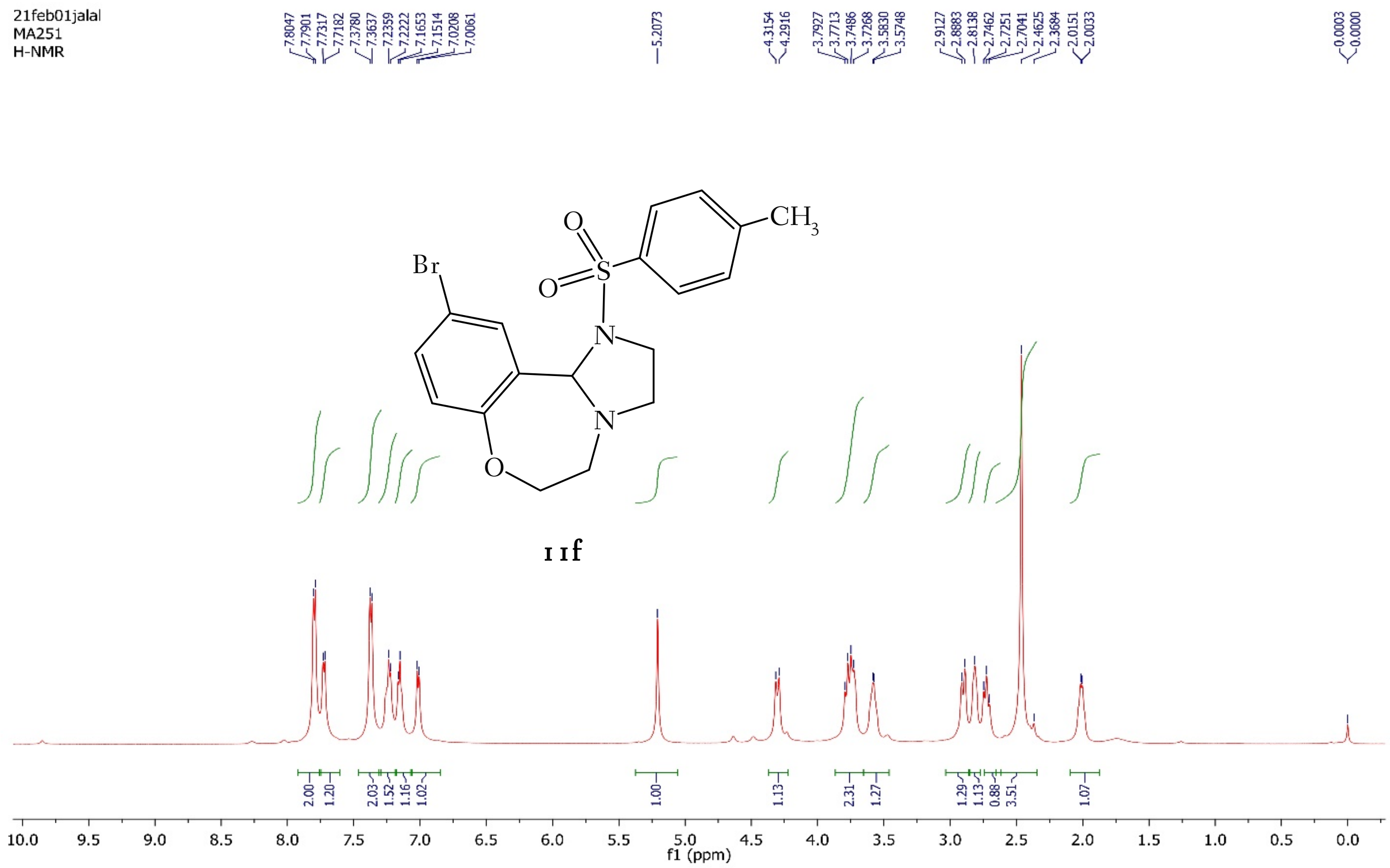


Fig.S76. $^{1}$H NMR spectrum of compound 11f $CDCl_3$

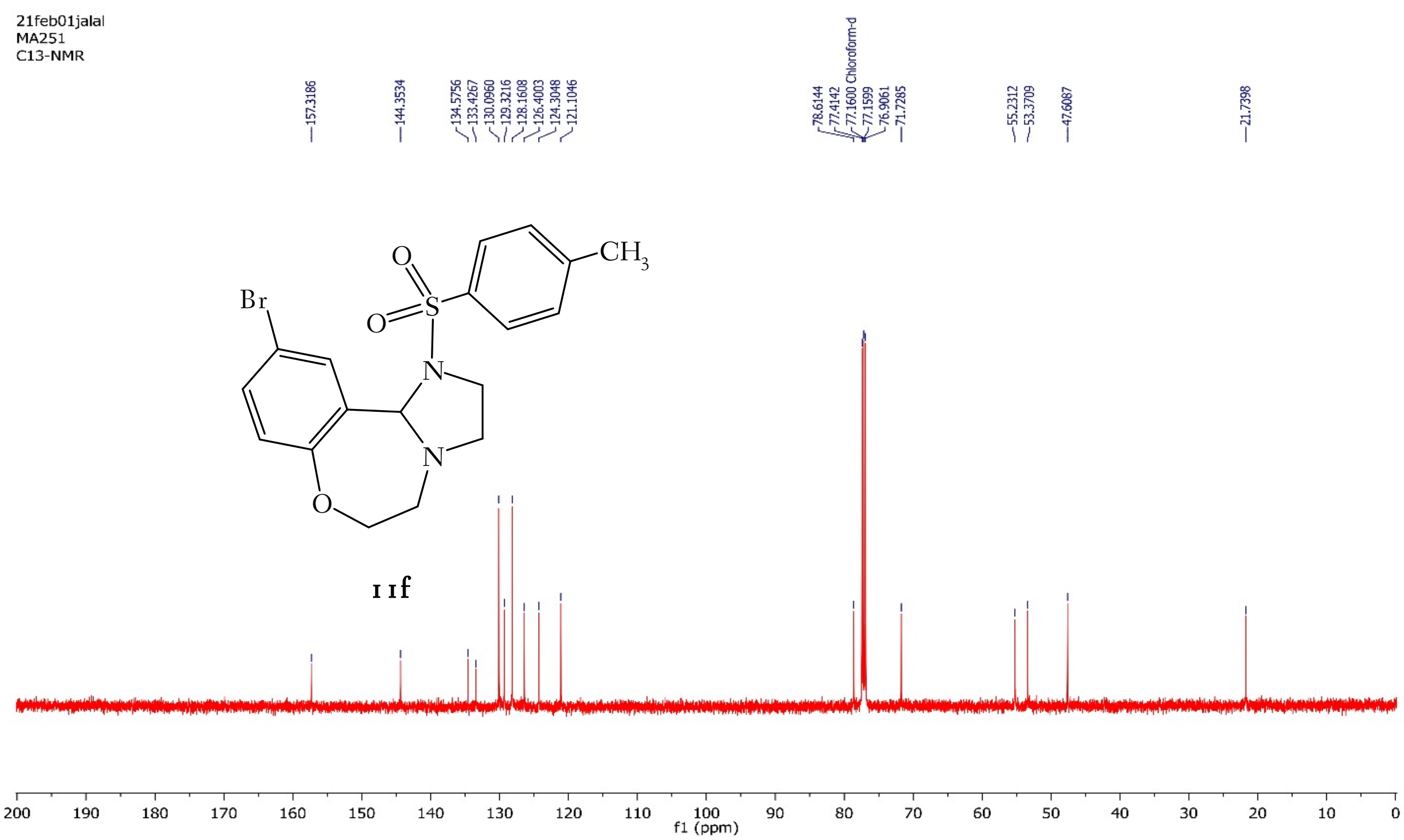


Fig.S77. $^{13}$C NMR spectrum of compound 11f $CDCl_3$

# The University of Jordan
## Faculty of science
## Department of Chemistry

### Mass Spectrum Molecular Formula Report

**Analysis Info**

| | | | |
|---|---|---|---|
| Analysis Name | F:\Data\2022MAR20\Mustafa_000068.d | Acquisition Date | 3/23/2022 2:52:13 PM |
| Method | ESI_pos_20181024 | Operator | Bruker_PC |
| Sample Name | AM251 | Instrument | apex-IV |
| Comment | CHCl3+ACN | | |

**Acquisition Parameter**

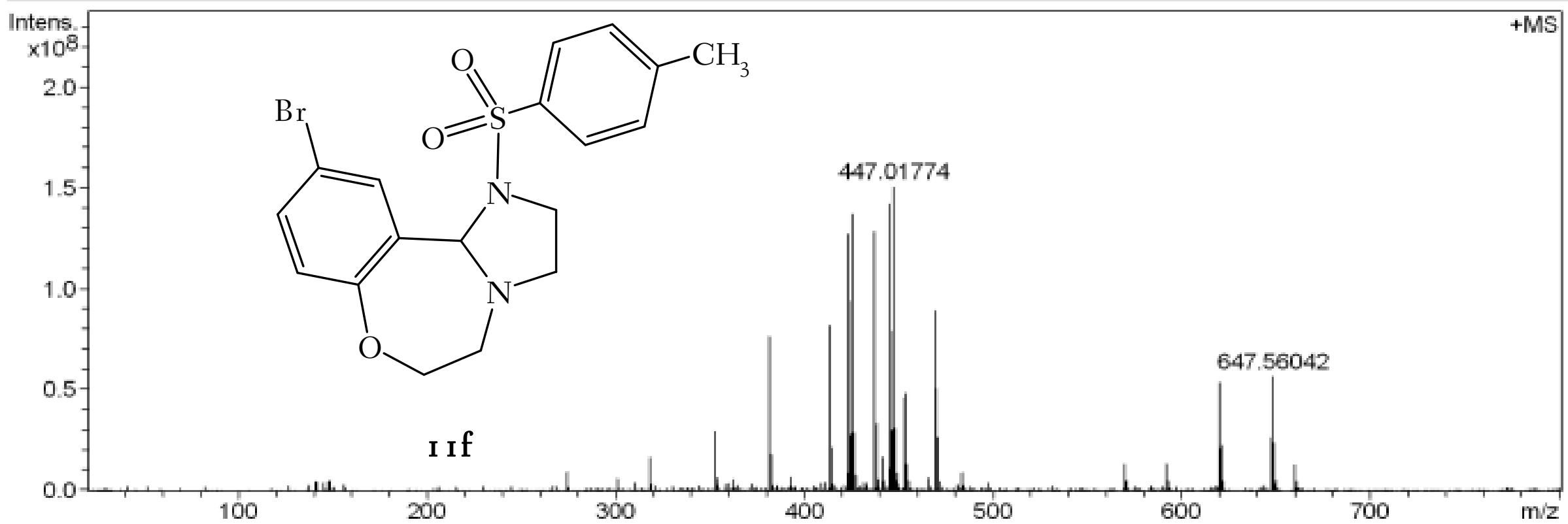


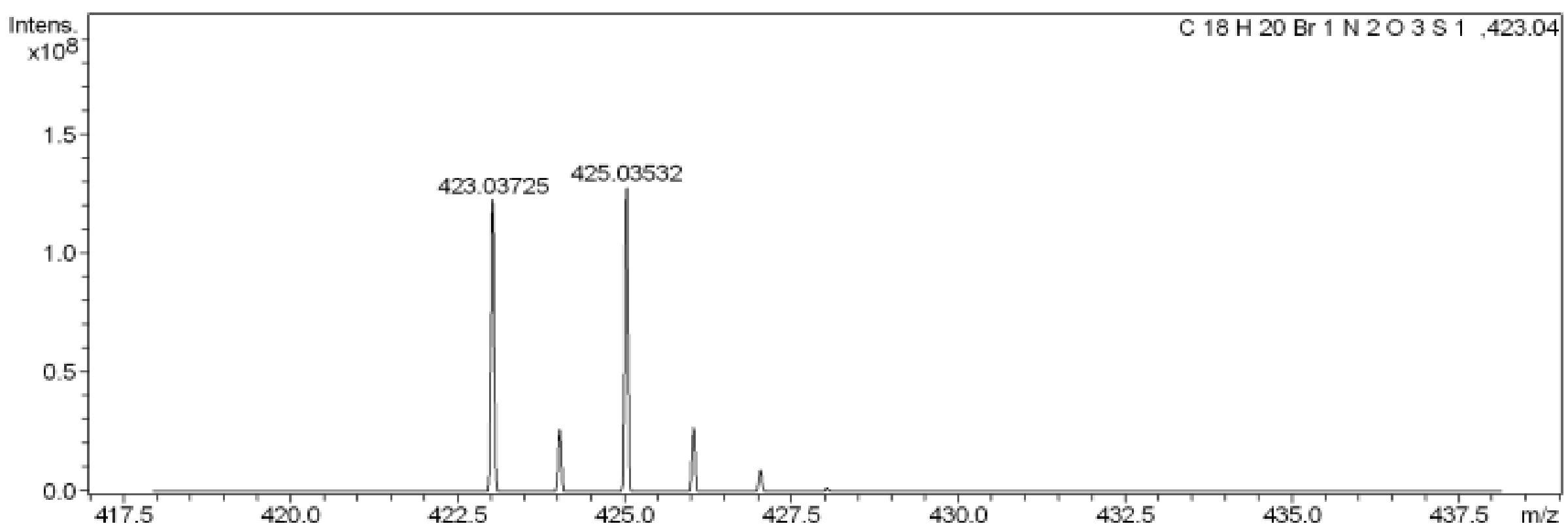


| Sum Formula | Sigma | m/z | Err [ppm] | Mean Err [ppm] | Err [mDa] | rdb | N Rule | e⁻ |
|---|---|---|---|---|---|---|---|---|
| C 18 H 20 Br 1 N 2 O 3 S 1 | 0.011 | 423.03725 | 0.41 | -0.55 | 0.17 | 9.50 | ok | even |

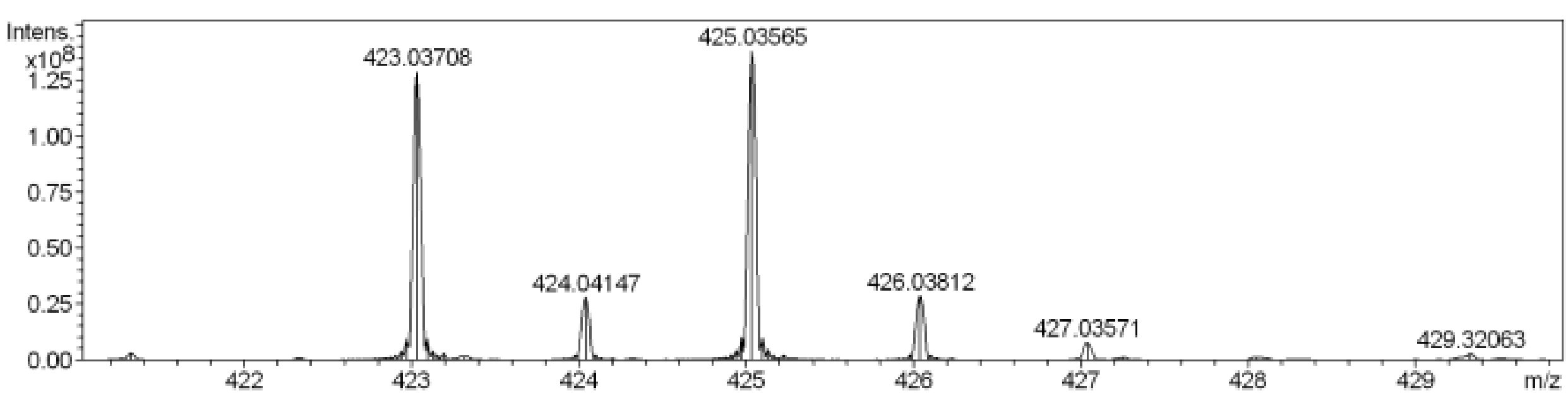


Fig.S78. HRMS spectrum of compound 11f

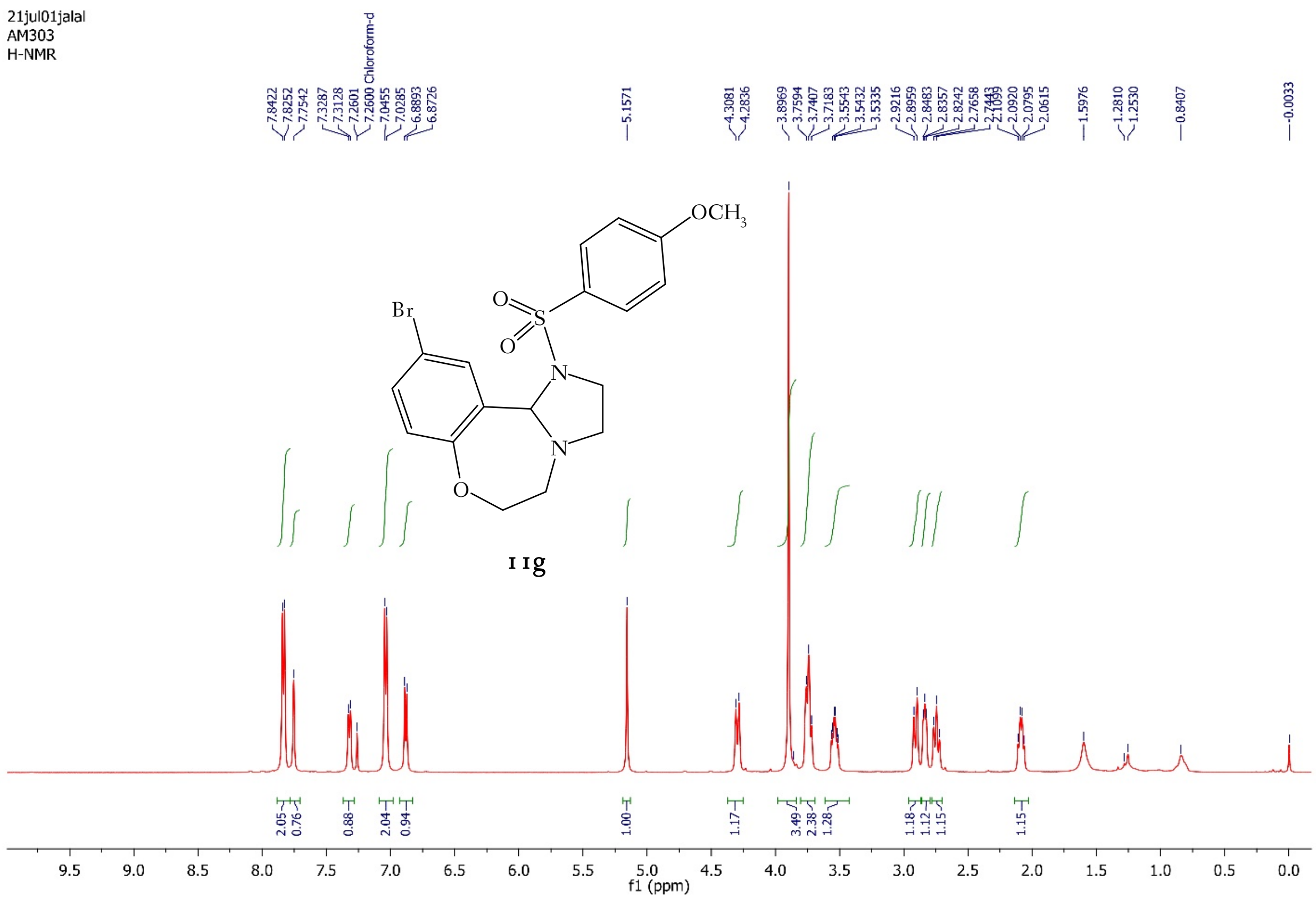

Fig.S79. $^1$H NMR spectrum of compound 11g $CDCl_3$

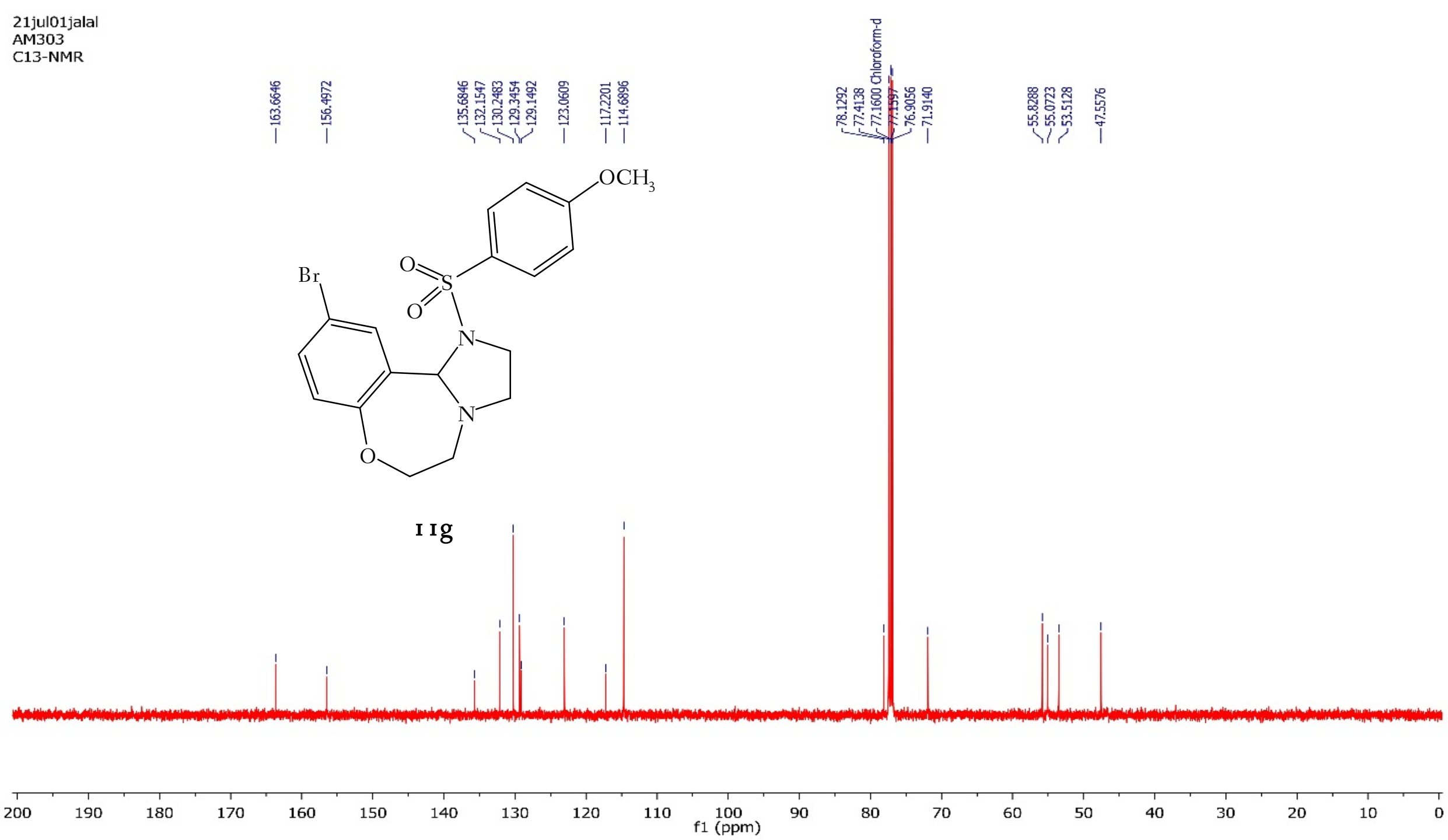

Fig.S80. $^{13}C$ NMR spectrum of compound 11g $CDCl_3$

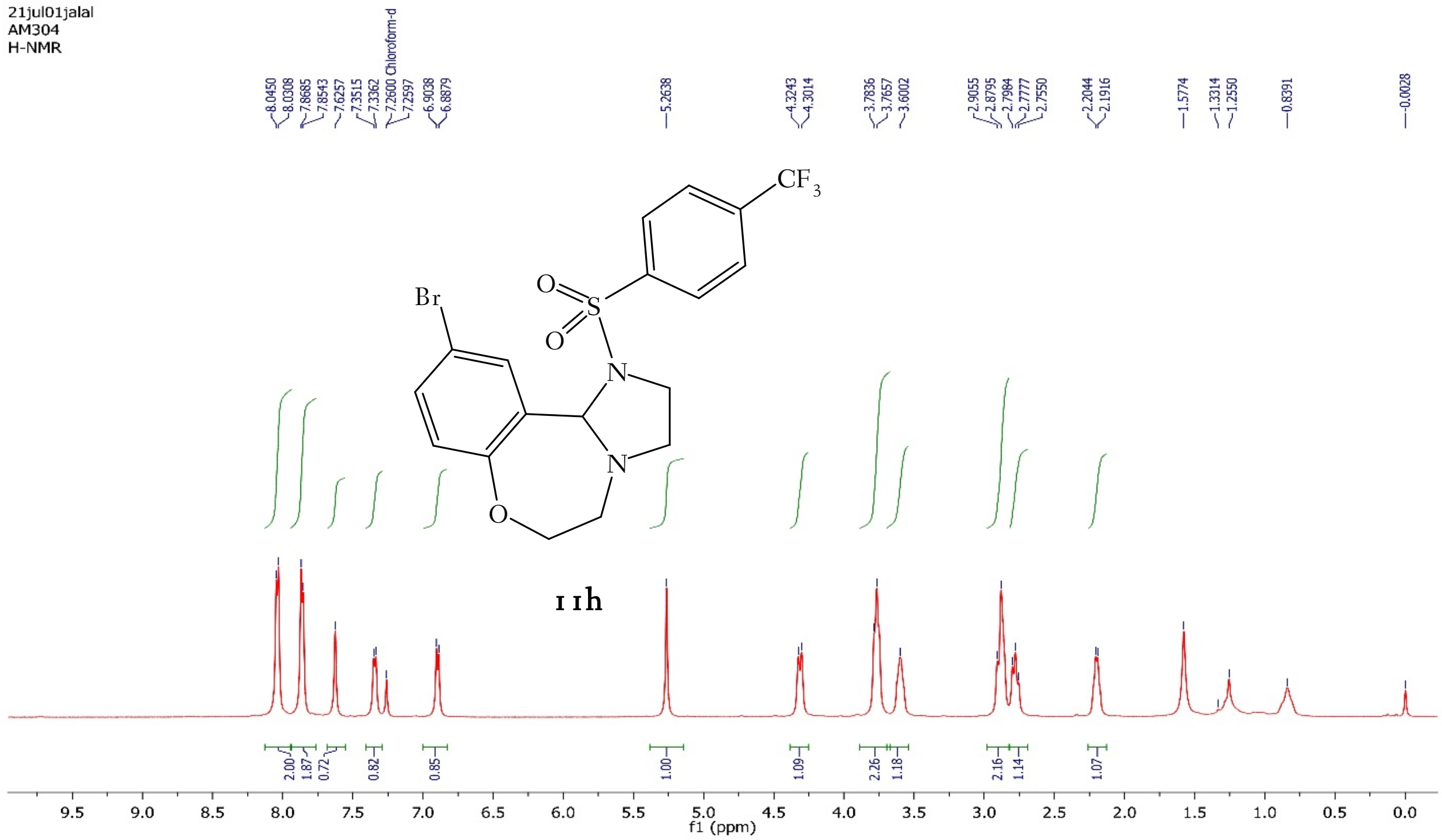


Fig.S81. $^{1}H$ NMR spectrum of compound 11h $CDCl_3$

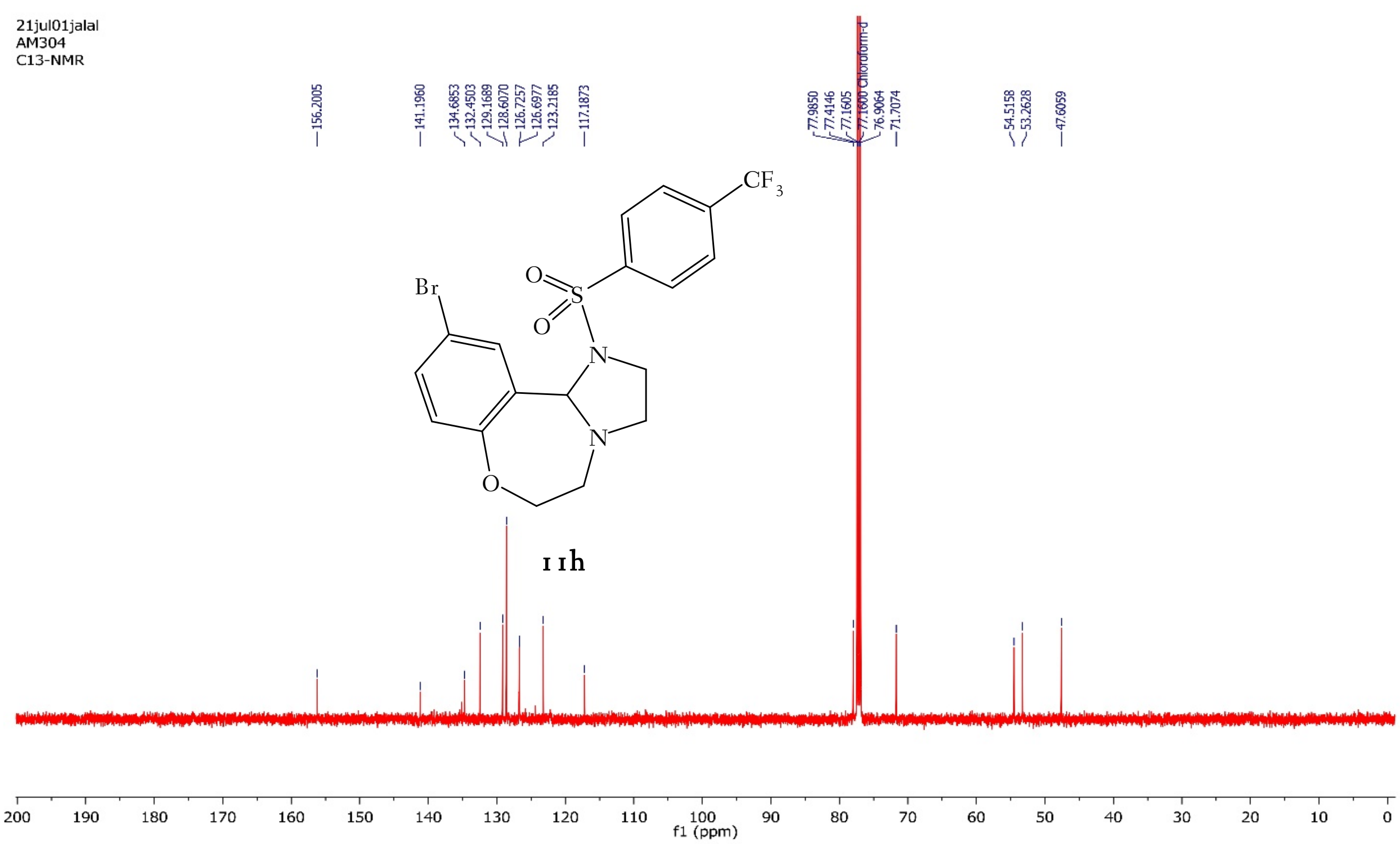


Fig.S82. $^{13}C$ NMR spectrum of compound 11h $CDCl_3$

# The University of Jordan
## Faculty of science
## Department of Chemistry

### Mass Spectrum Molecular Formula Report

**Analysis Info**

| | | | |
|---|---|---|---|
| Analysis Name | F:\Data\2022MAR08\Mustafa_000040.d | Acquisition Date | 3/13/2022 12:12:49 PM |
| Method | ESI_pos_20181024 | Operator | Bruker_PC |
| Sample Name | AM304 | Instrument | apex-IV |
| Comment | CHCl3+ACN | | |

**Acquisition Parameter**

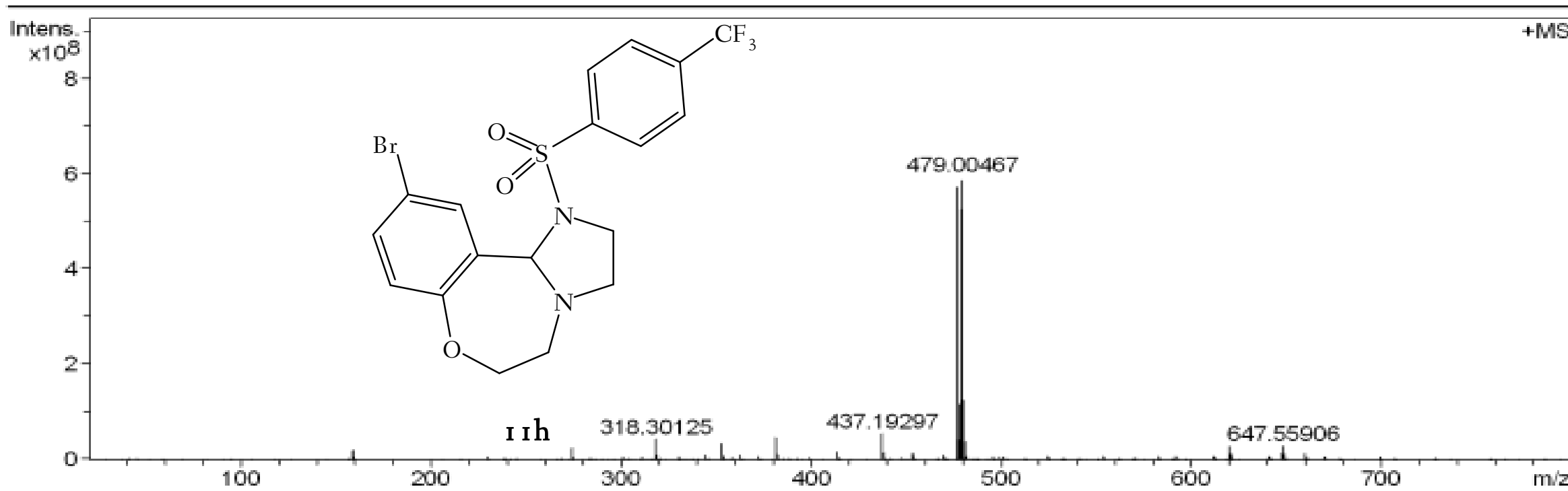


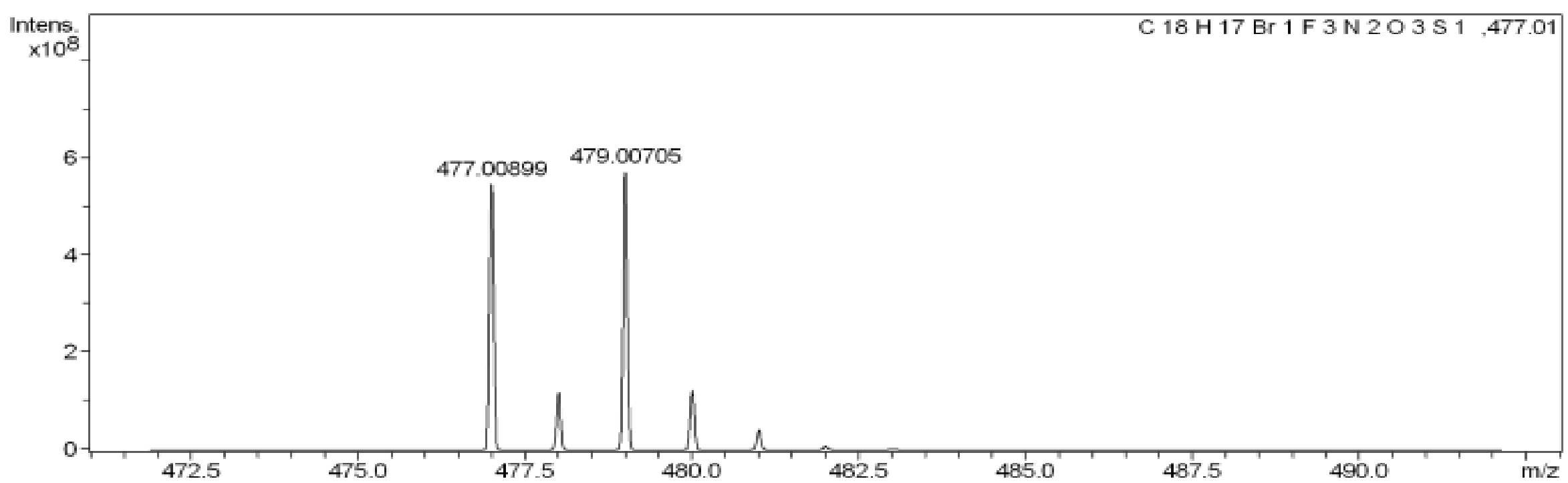


| Sum Formula | Sigma | m/z | Err [ppm] | Mean Err [ppm] | Err [mDa] | rdb | N Rule | e⁻ |
|---|---|---|---|---|---|---|---|---|
| C 18 H 17 Br 1 F 3 N 2 O 3 S 1 | 0.008 | 477.00899 | 1.61 | 3.23 | 0.77 | 9.50 | ok | even |

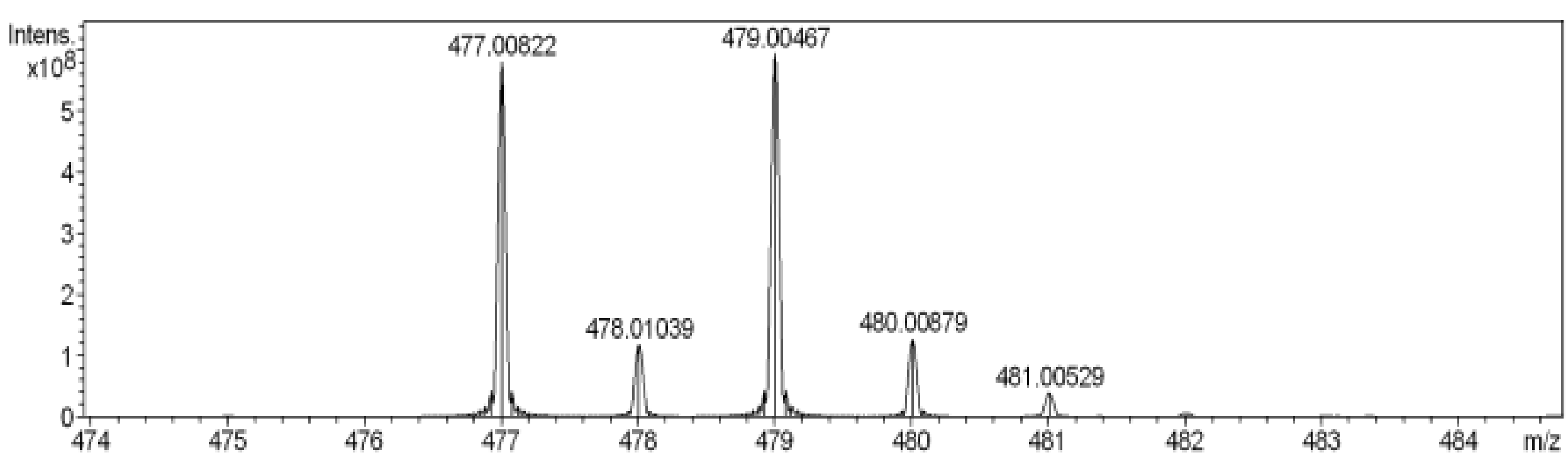


Fig.S83. HRMS spectrum of compound 11h

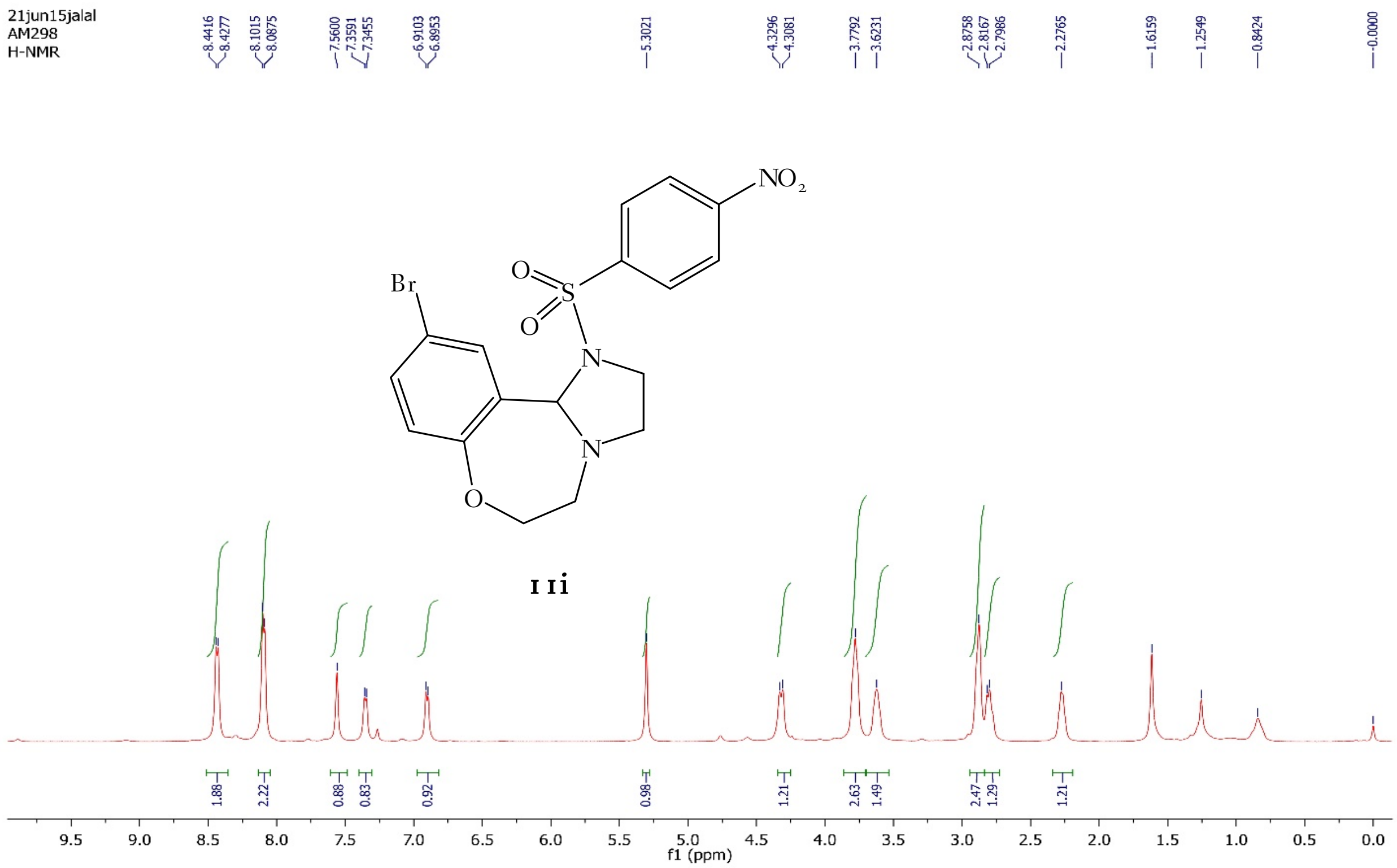


Fig.S84. $^{1}$H NMR spectrum of compound 11i $CDCl_3$

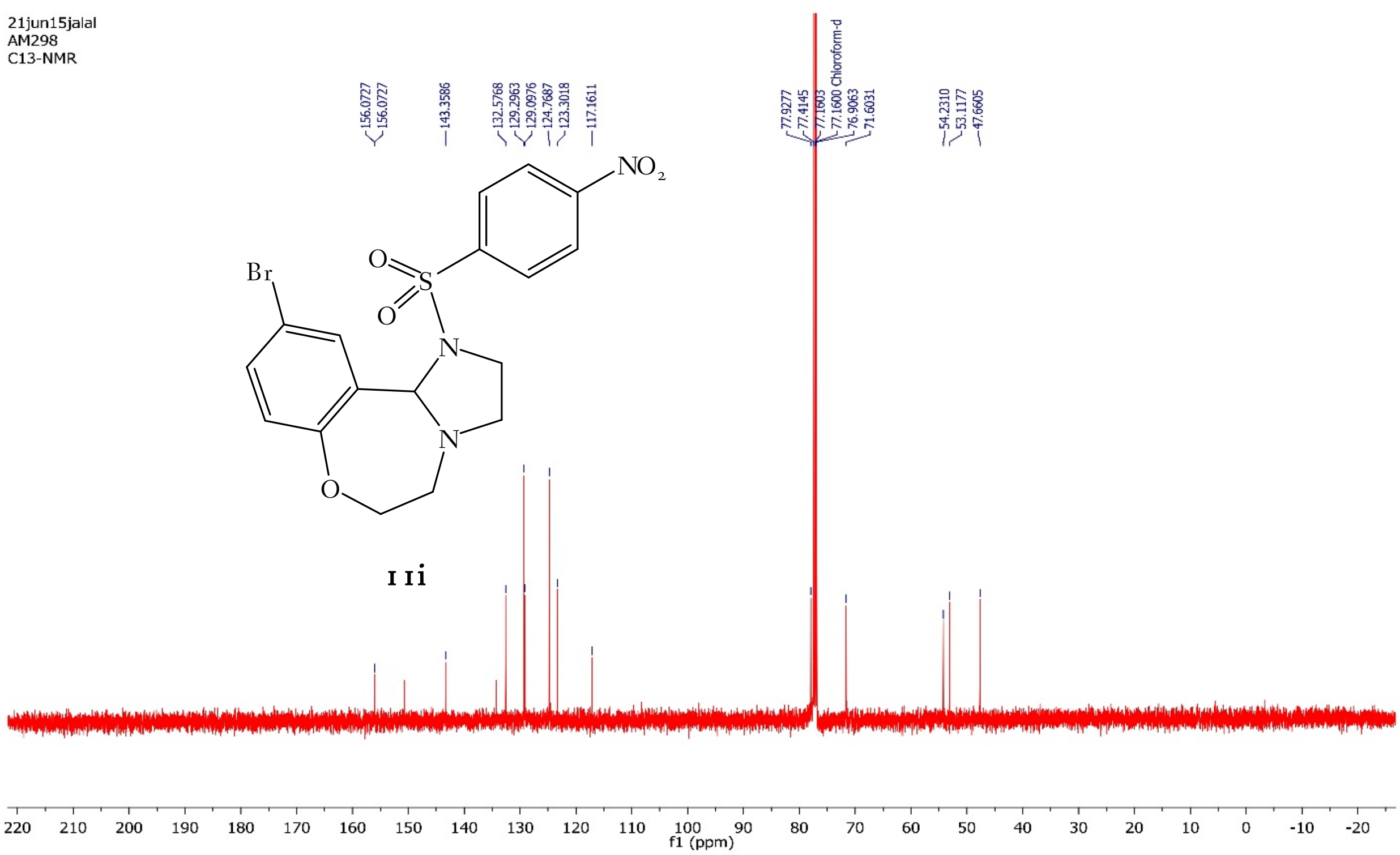


Fig.S85. $^{13}$C NMR spectrum of compound 11i $CDCl_3$

The University of Jordan
Faculty of science
Department of Chemistry

# Mass Spectrum Molecular Formula Report

| | | | |
|---|---|---|---|
| **Analysis Info** | | Acquisition Date | 3/30/2022 11:18:37 AM |
| Analysis Name | F:\Data\2022MAR30\Mustafa_000011.d | | |
| Method | ESI_pos_20181024 | Operator | Bruker_PC |
| Sample Name | AM298 | Instrument | apex-IV |
| Comment | CHCl3+ACN | | |

**Acquisition Parameter**

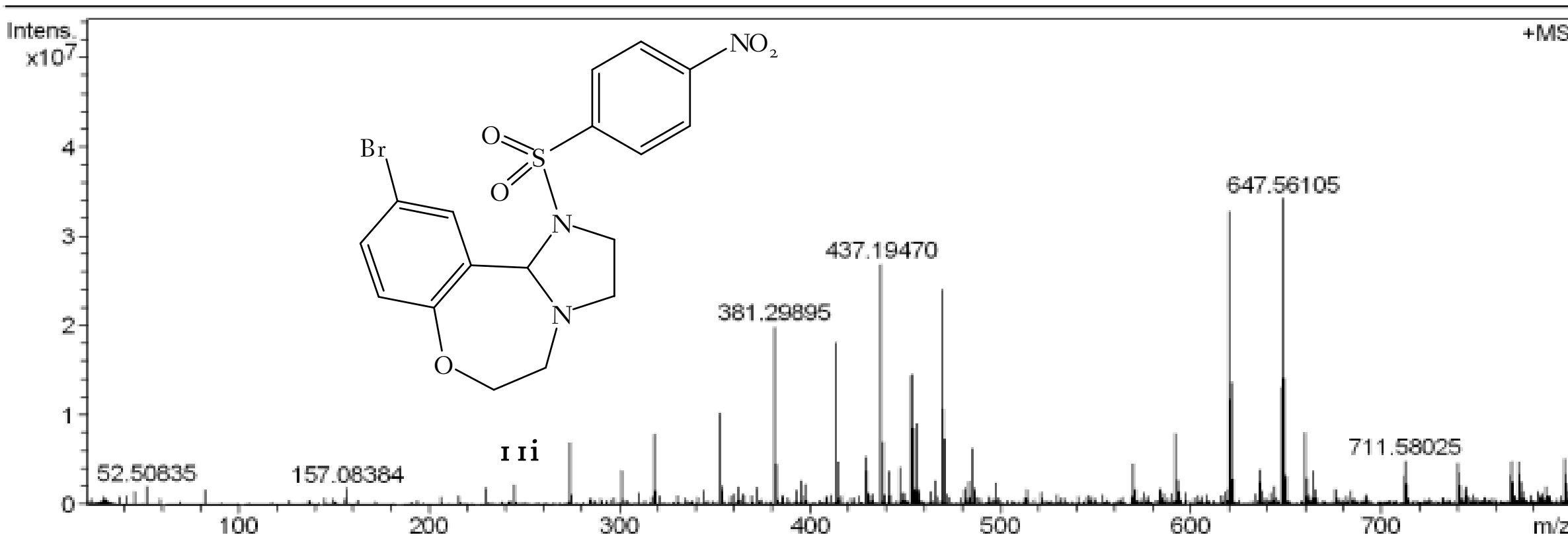


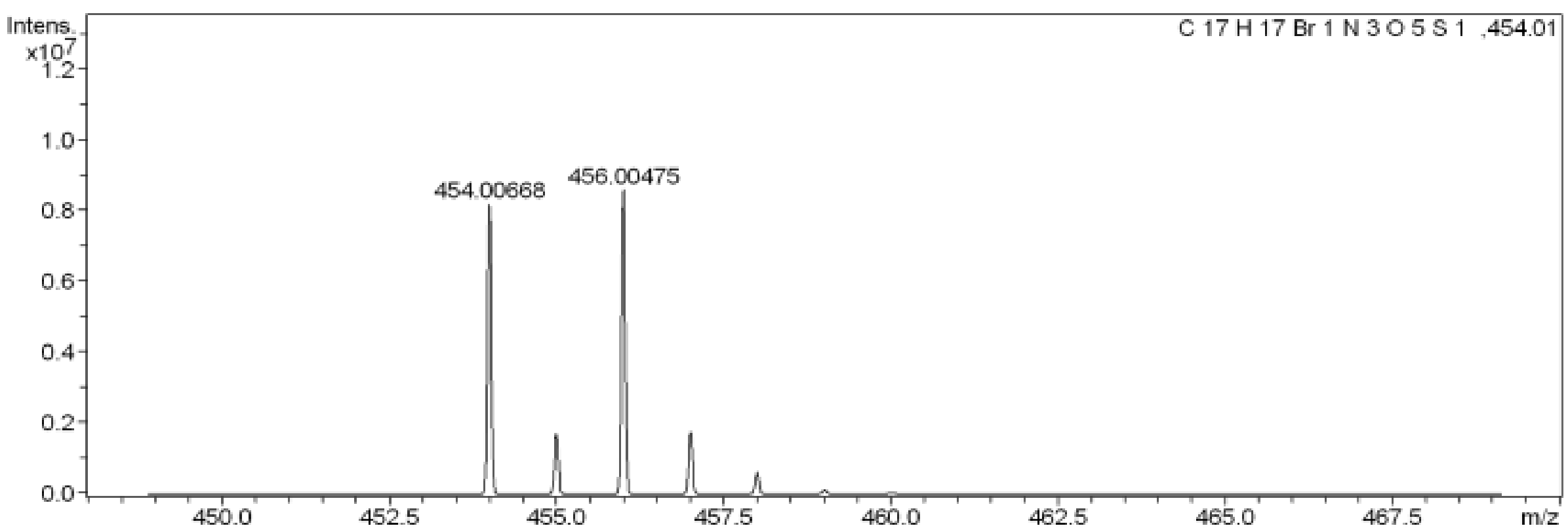


| Sum Formula | Sigma | m/z | Err [ppm] | Mean Err [ppm] | Err [mDa] | rdb | N Rule | e⁻ |
|---|---|---|---|---|---|---|---|---|
| C 17 H 17 Br 1 N 3 O 5 S 1 | 0.008 | 454.00668 | -3.25 | -3.32 | -1.48 | 10.50 | ok | even |

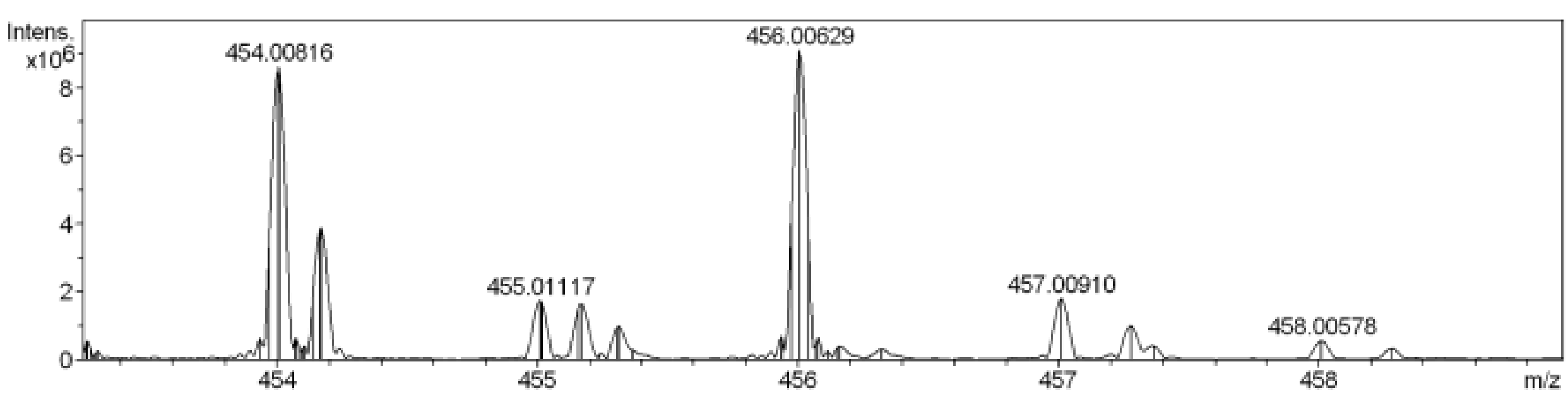


Fig.S86. HRMS spectrum of compound 11i

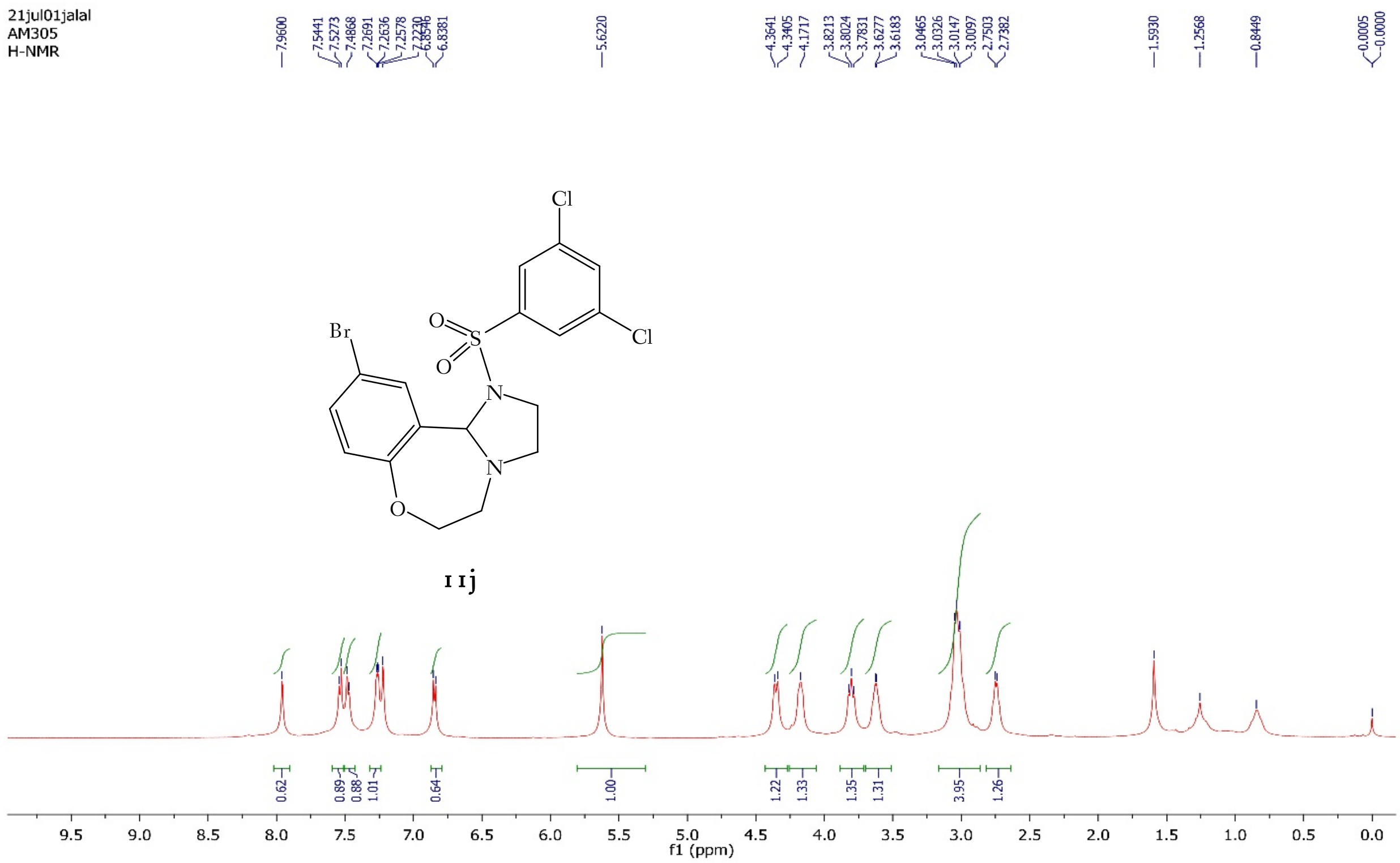


Fig.S87. $^{1}$H NMR spectrum of compound 11j $CDCl_3$

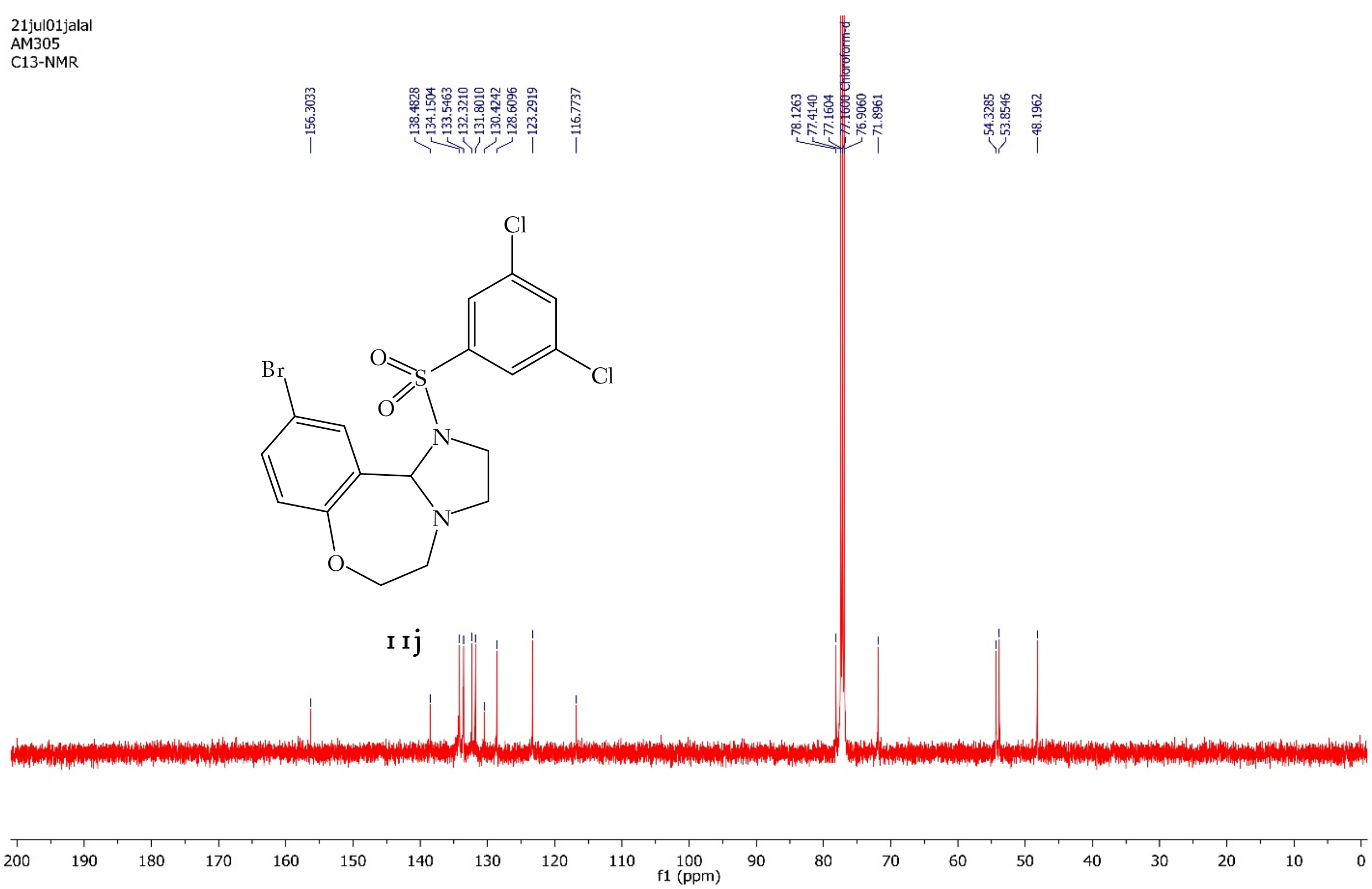

Fig.S88. $^{13}$C NMR spectrum of compound 11j $CDCl_3$

# The University of Jordan

## Faculty of science

## Department of Chemistry

## Mass Spectrum Molecular Formula Report

**Analysis Info**

| | | | |
|---|---|---|---|
| Analysis Name | F:\Data\2022MAR08\Mustafa_000042.d | Acquisition Date | 3/13/2022 12:21:09 PM |
| Method | ESI_pos_20181024 | Operator | Bruker_PC |
| Sample Name | AM305 | Instrument | apex-IV |
| Comment | CHCl3+ACN | | |

**Acquisition Parameter**

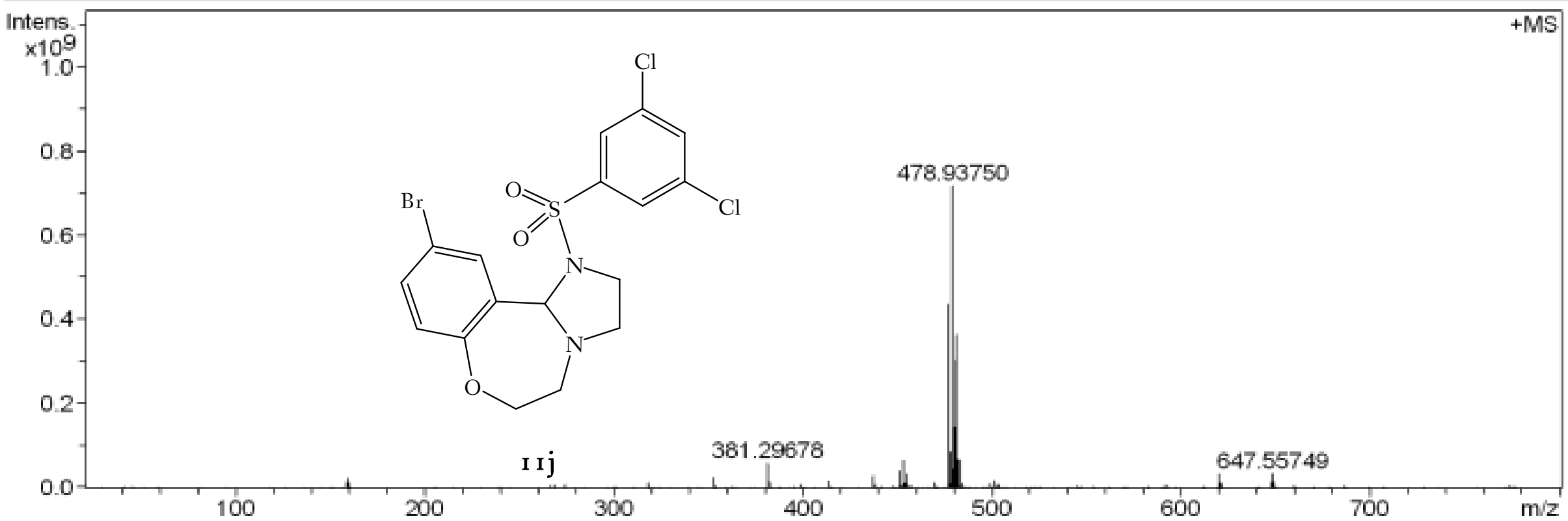


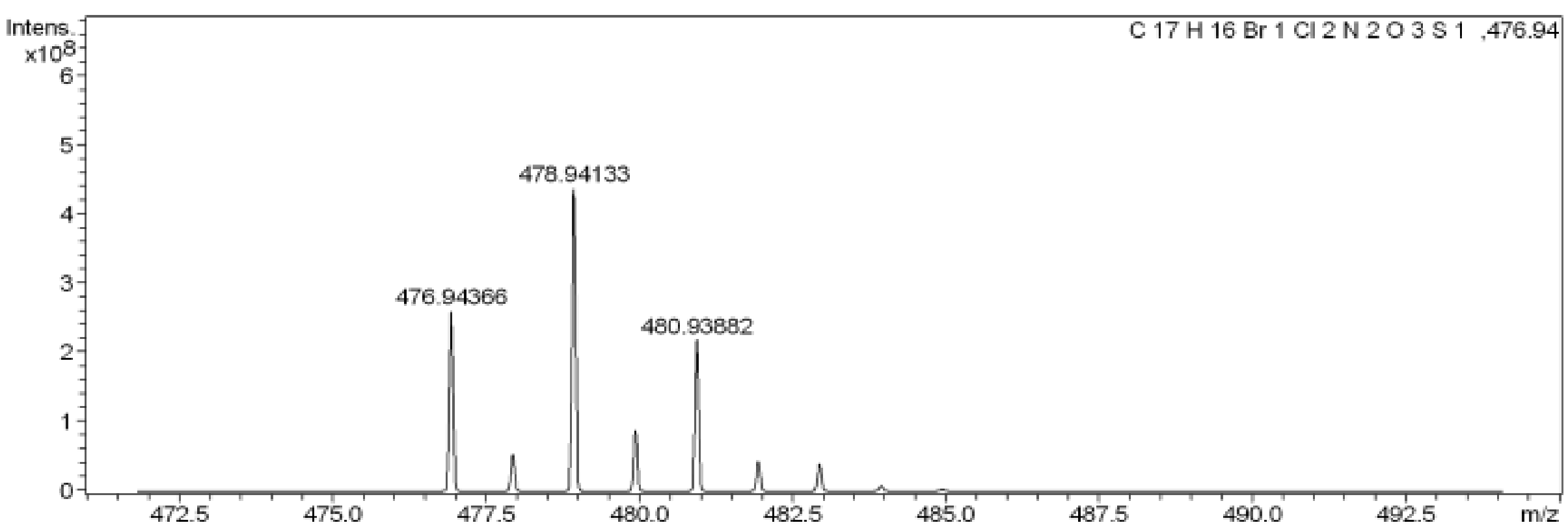


| Sum Formula | Sigma | m/z | Err [ppm] | Mean Err [ppm] | Err [mDa] | rdb | N Rule | e⁻ |
|---|---|---|---|---|---|---|---|---|
| C 17 H 16 Br 1 Cl 2 N 2 O 3 S 1 | 0.007 | 476.94366 | 2.81 | 5.44 | 1.34 | 9.50 | ok | even |

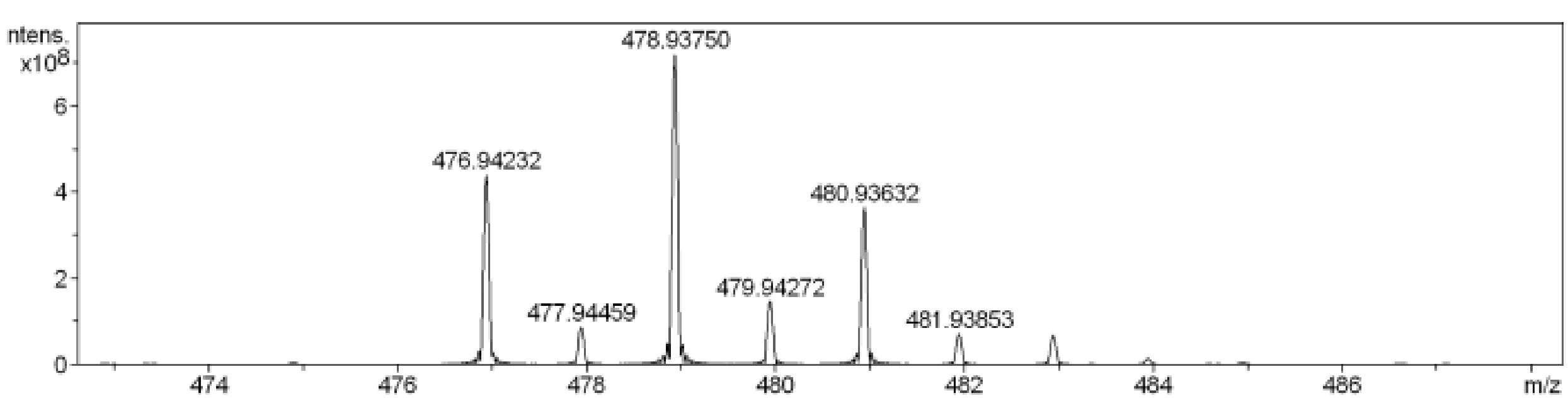

Fig.S89. HRMS spectrum of compound 11j

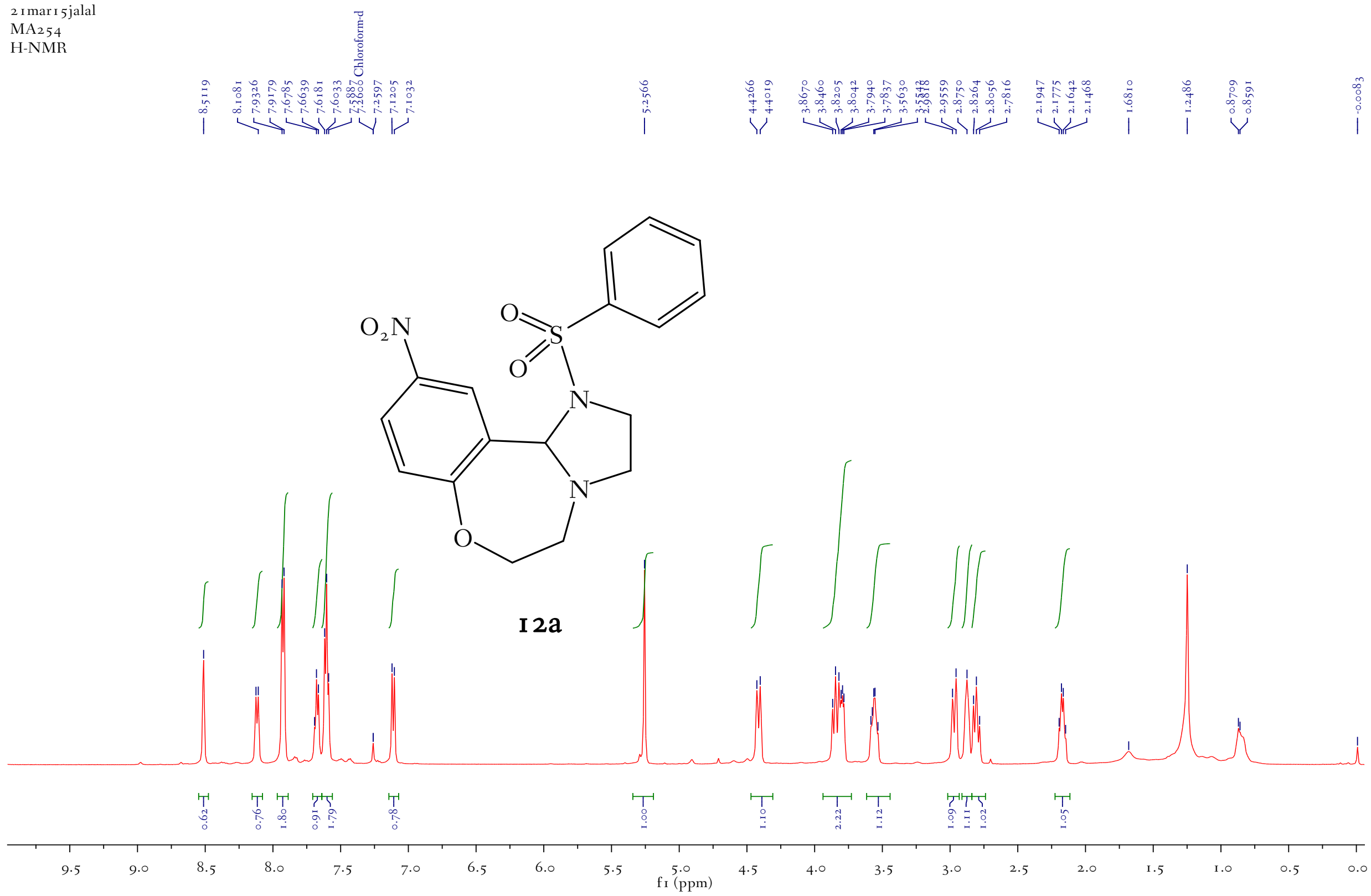


Fig.S90. $^1$H NMR spectrum of compound 12a $CDCl_3$

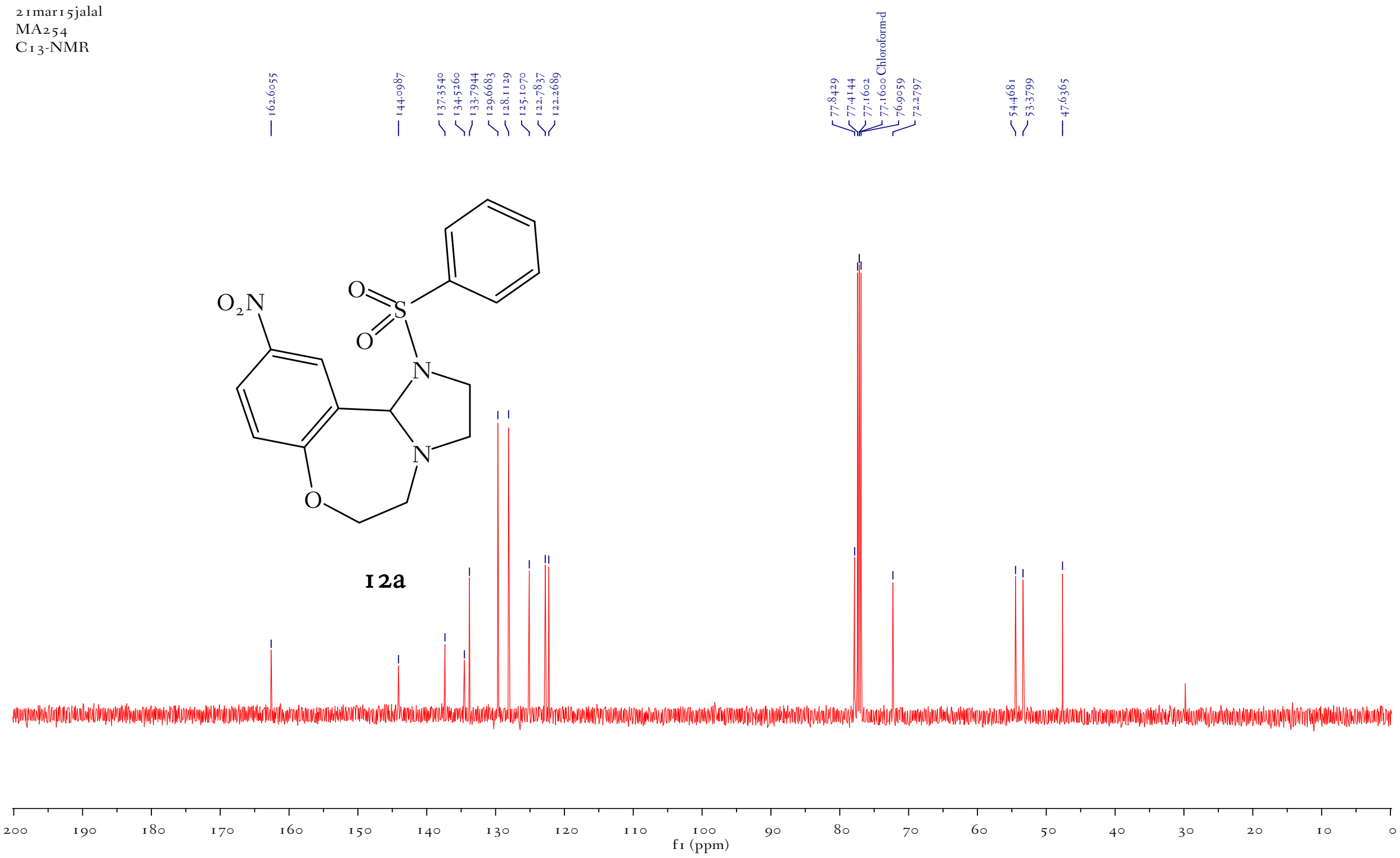


Fig.S91. $^{13}$C NMR spectrum of compound 12a $CDCl_3$

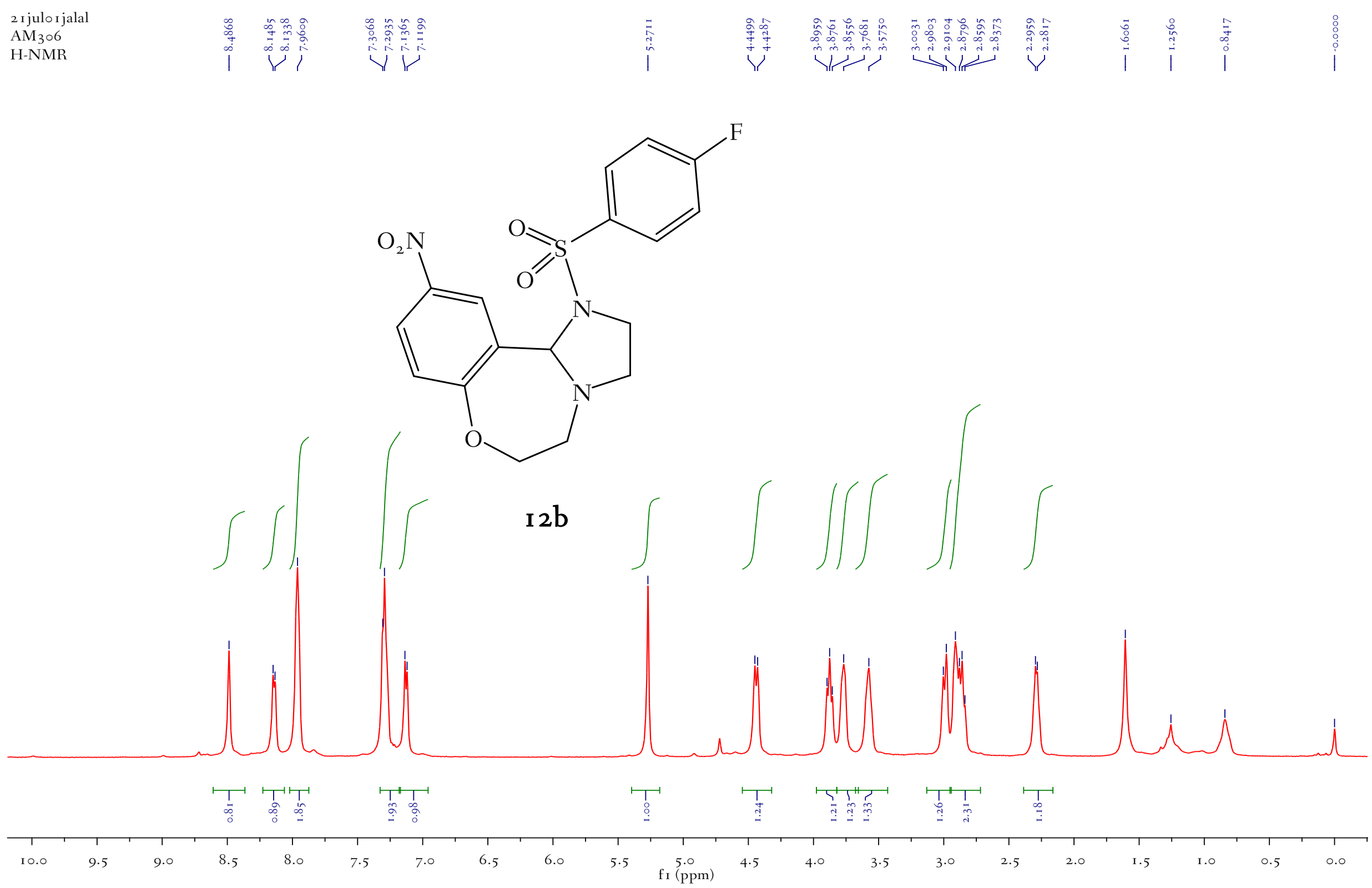


Fig.S92. $^{1}$H NMR spectrum of compound 12b $CDCl_3$

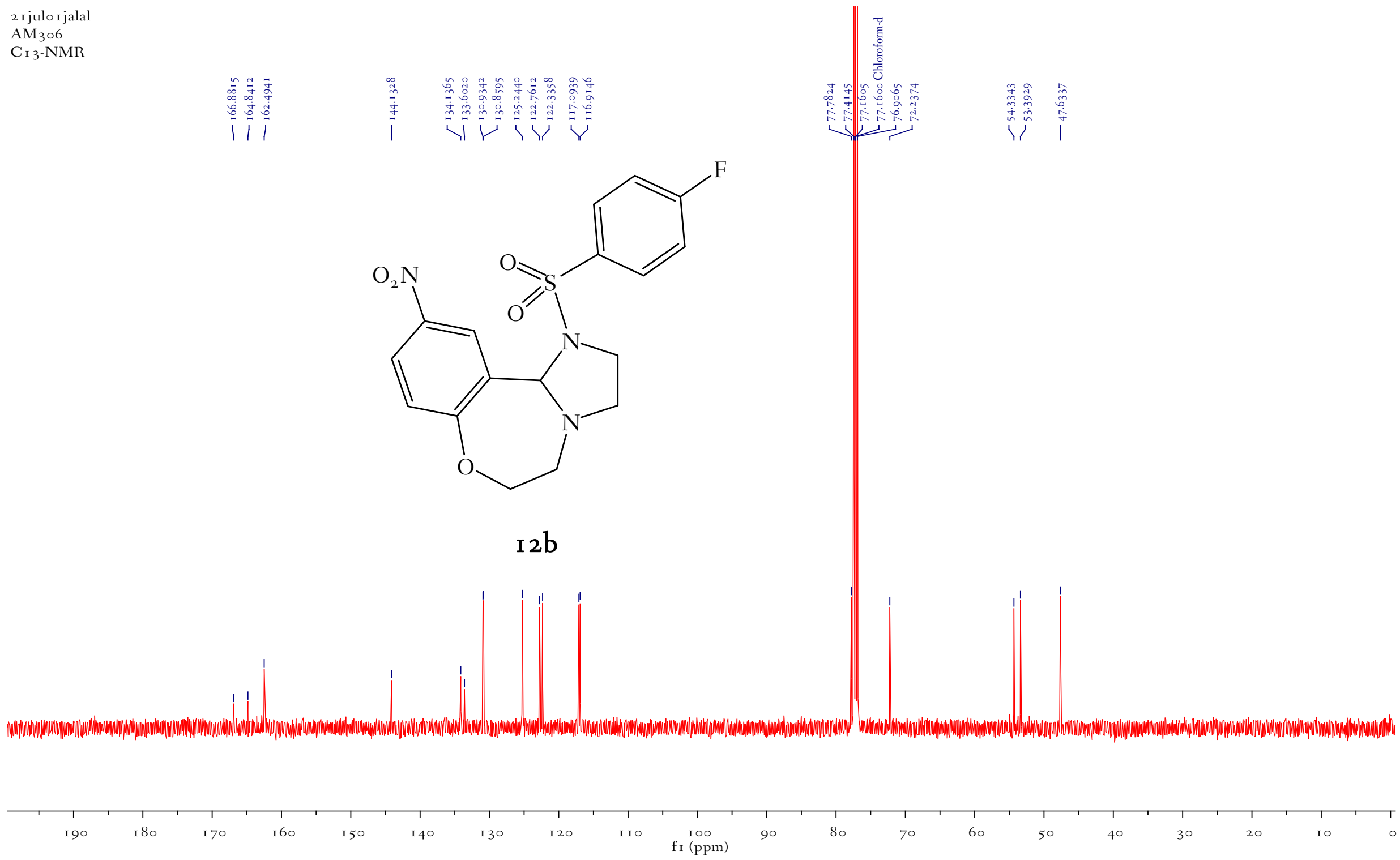


**12b**

Fig.S93. $^{13}$C NMR spectrum of compound 12b $CDCl_3$

# The University of Jordan
## Faculty of science
## Department of Chemistry

# Mass Spectrum Molecular Formula Report

**Analysis Info**

| | | | |
|---|---|---|---|
| Analysis Name | F:\Data\2022MAR08\Mustafa_000033.d | Acquisition Date | 3/13/2022 11:41:26 AM |
| Method | ESI_pos_20181024 | Operator | Bruker_PC |
| Sample Name | AM306 | Instrument | apex-IV |
| Comment | CHCl3+ACN | | |

**Acquisition Parameter**

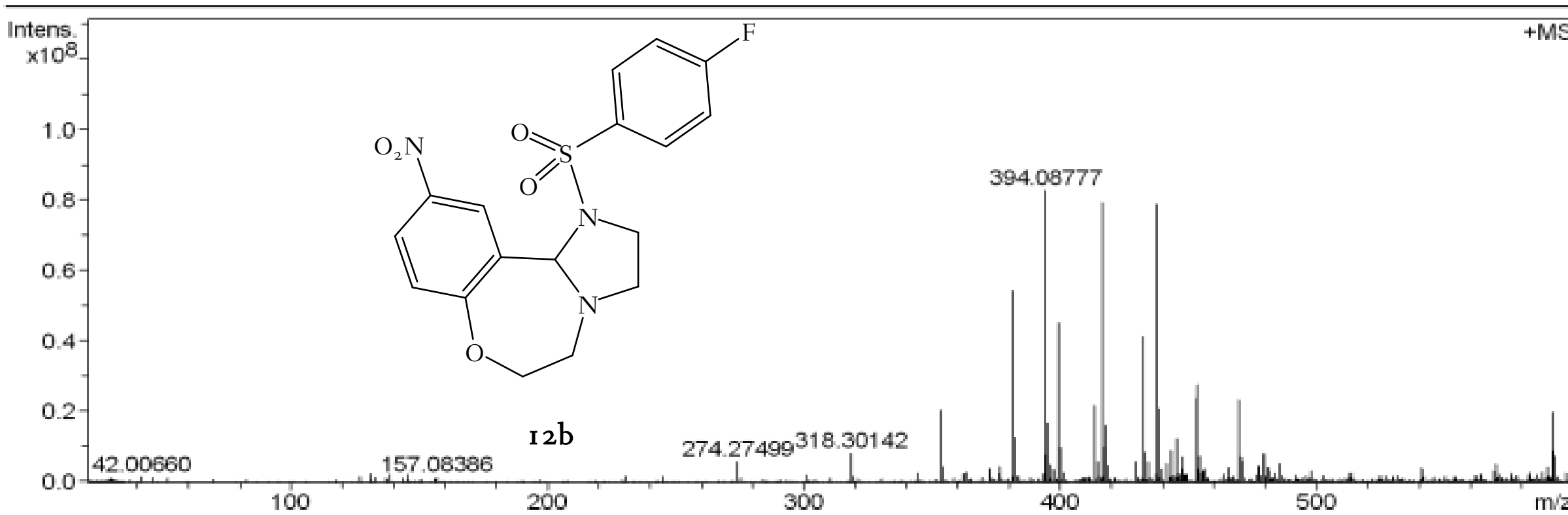


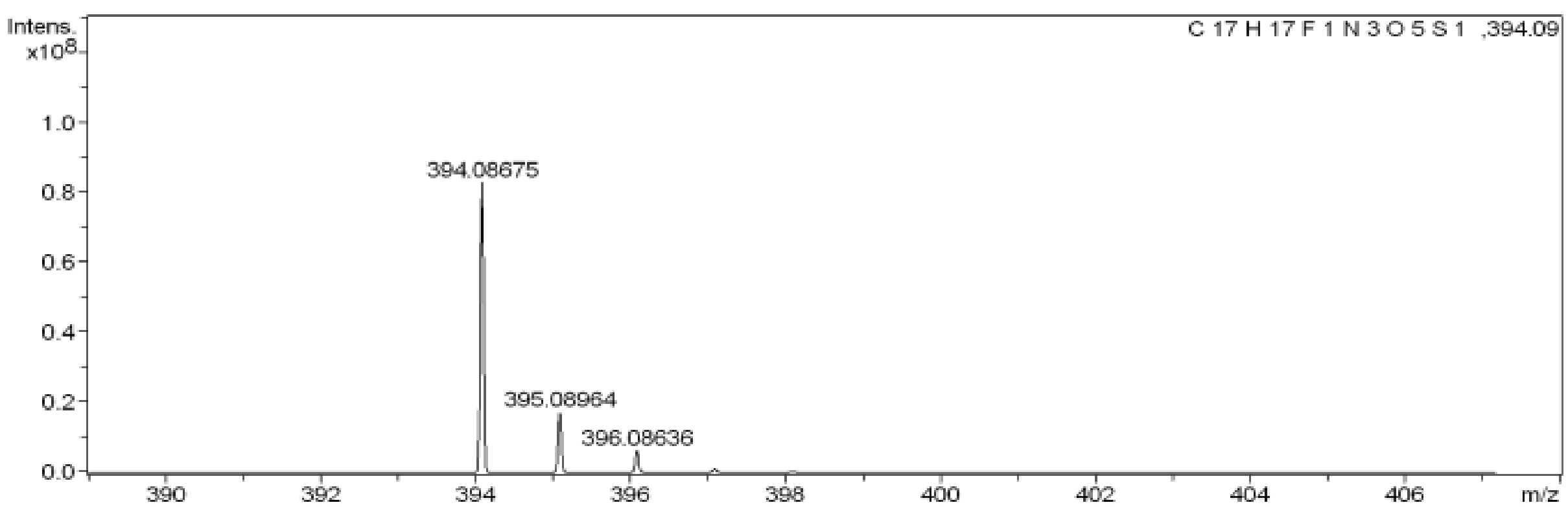


| Sum Formula | Sigma | m/z | Err [ppm] | Mean Err [ppm] | Err [mDa] | rdb | N Rule | e⁻ |
|---|---|---|---|---|---|---|---|---|
| C 17 H 17 F 1 N 3 O 5 S 1 | 0.008 | 394.08675 | -2.60 | -2.64 | -1.02 | 10.50 | ok | even |

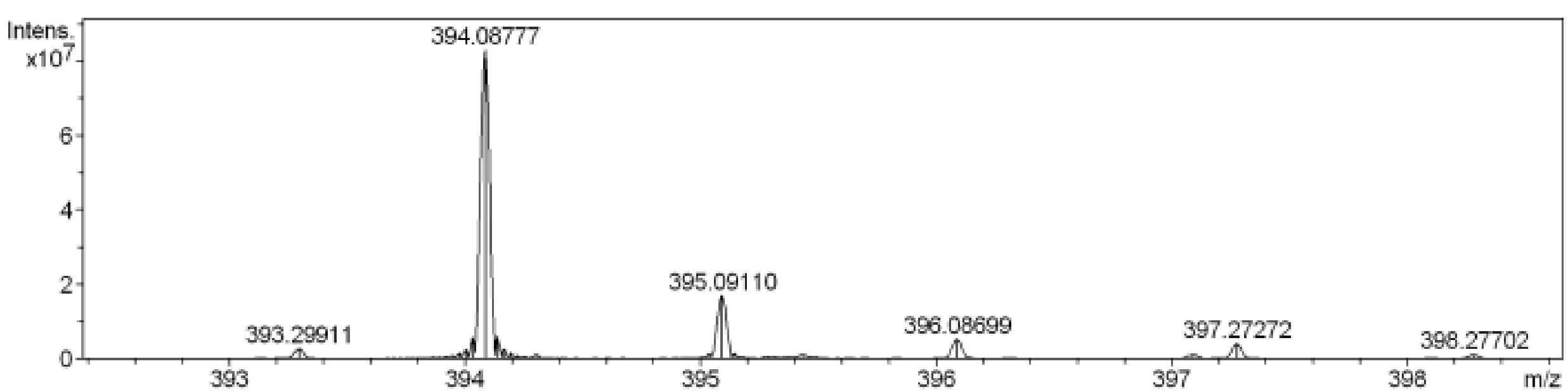


Fig.S94. HRMS spectrum of compound 12b

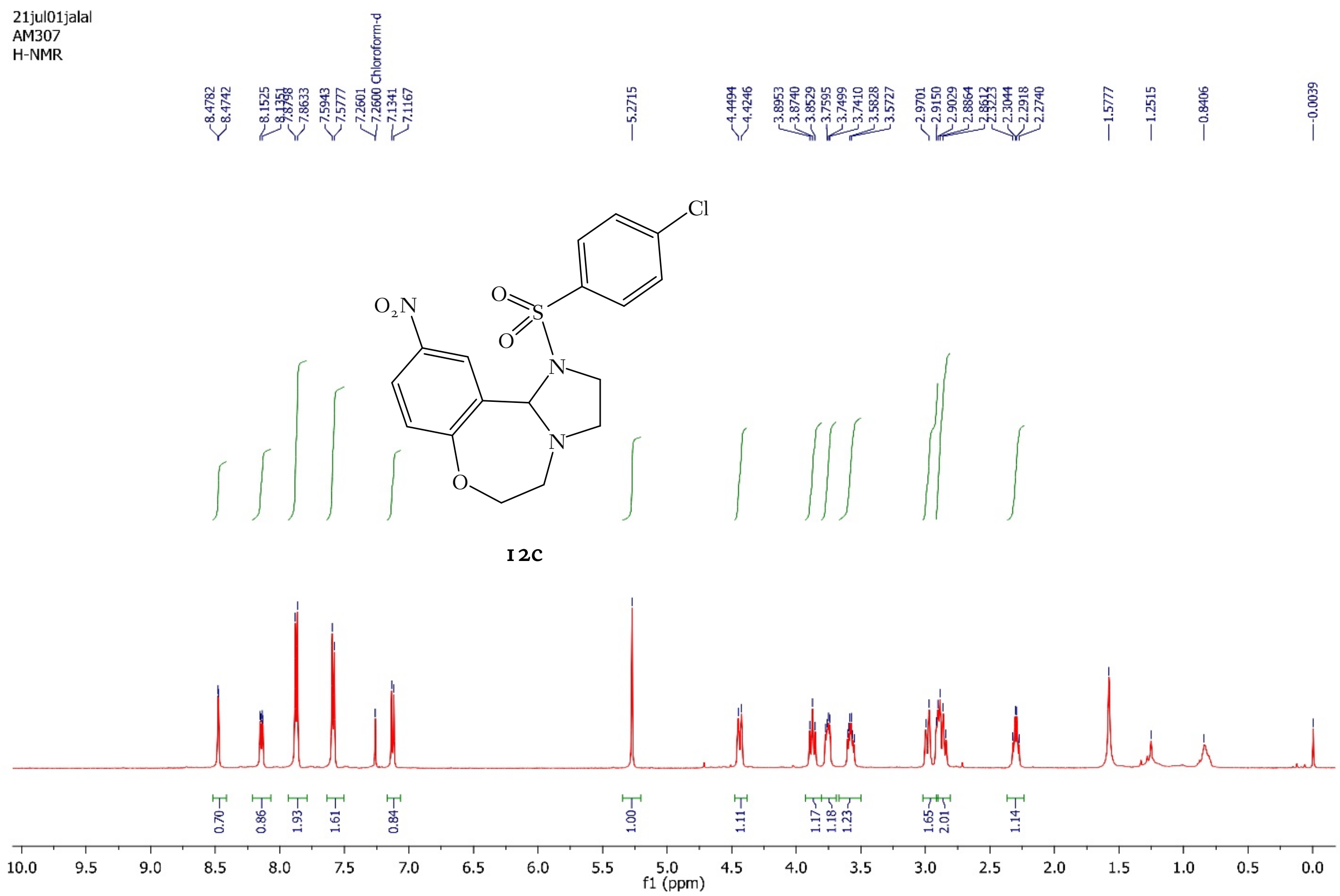


Fig.S95. $^{1}$H NMR spectrum of compound 12c $CDCl_3$

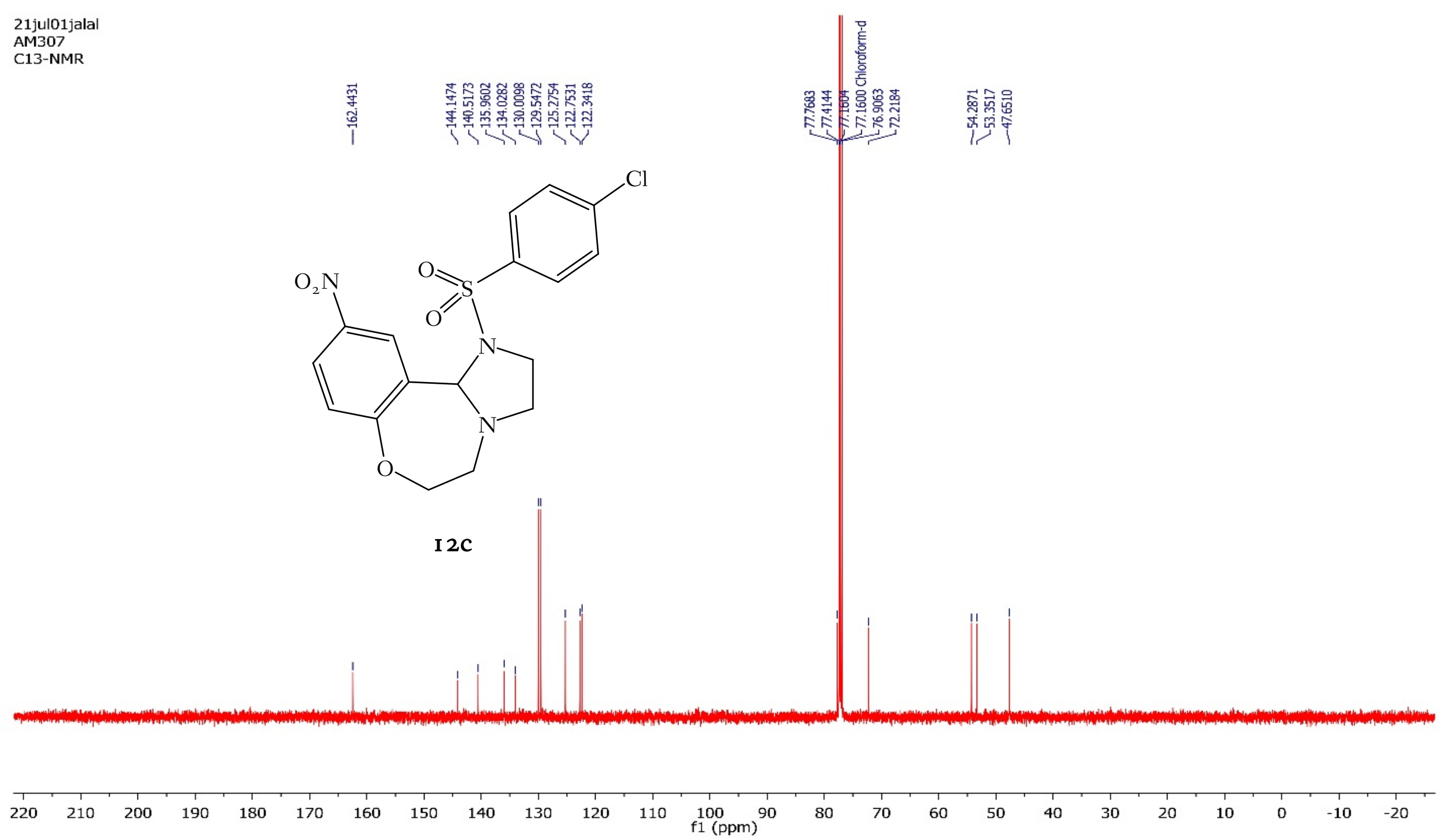


Fig. S96. $^{13}$C NMR spectrum of compound 12c $CDCl_3$

# The University of Jordan
## Faculty of science
## Department of Chemistry

## Mass Spectrum Molecular Formula Report

**Analysis Info**

| | | | |
|---|---|---|---|
| Analysis Name | F:\Data\2022MAR08\Mustafa_000034.d | Acquisition Date | 3/13/2022 11:45:30 AM |
| Method | ESI_pos_20181024 | Operator | Bruker_PC |
| Sample Name | AM307 | Instrument | apex-IV |
| Comment | CHCl3+ACN | | |

**Acquisition Parameter**

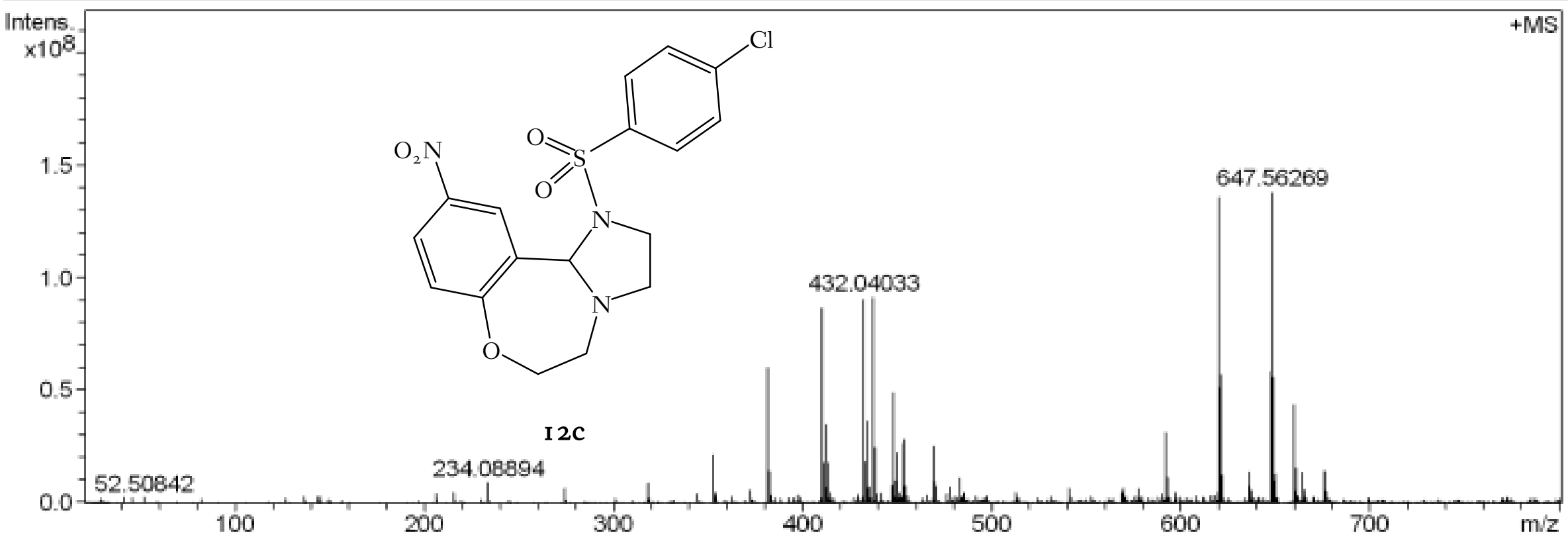


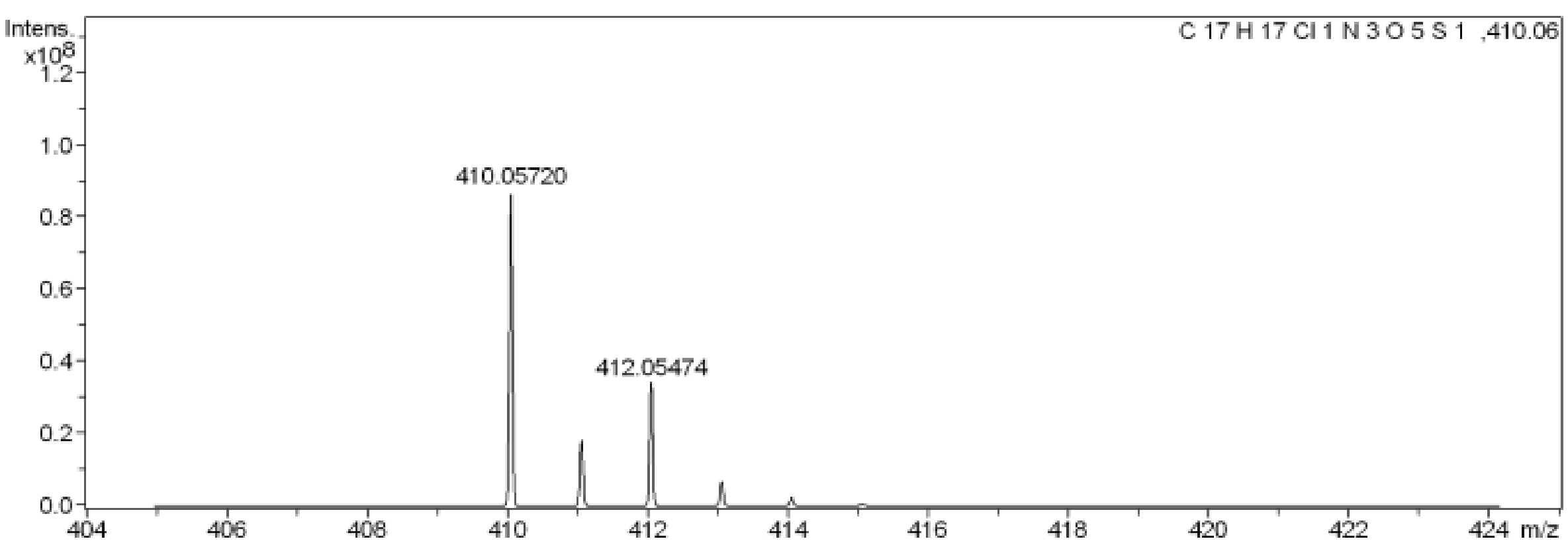


| Sum Formula | Sigma | m/z | Err [ppm] | Mean Err [ppm] | Err [mDa] | rdb | N Rule | e⁻ |
|---|---|---|---|---|---|---|---|---|
| C 17 H 17 Cl 1 N 3 O 5 S 1 | 0.003 | 410.05720 | -3.87 | -4.40 | -1.59 | 10.50 | ok | even |

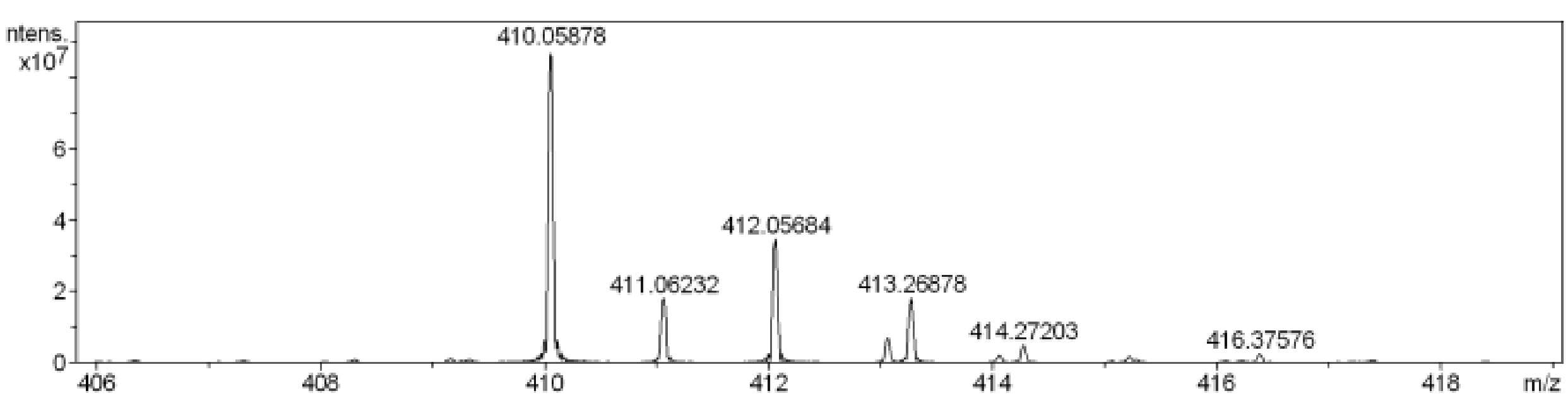


Fig.S97. HRMS spectrum of compound 12c

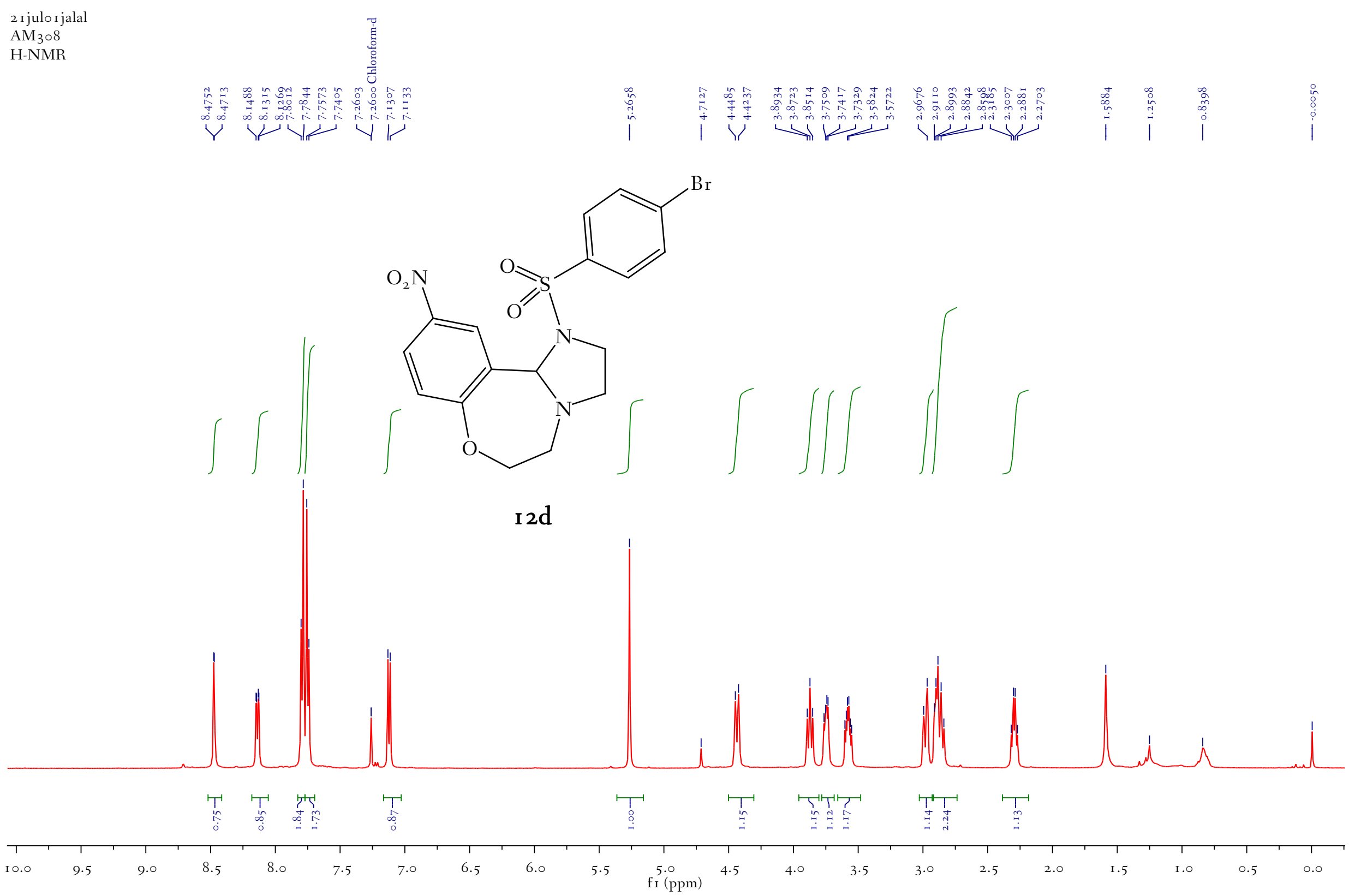


Fig.S98. ¹H NMR spectrum of compound 12d $CDCl_3$

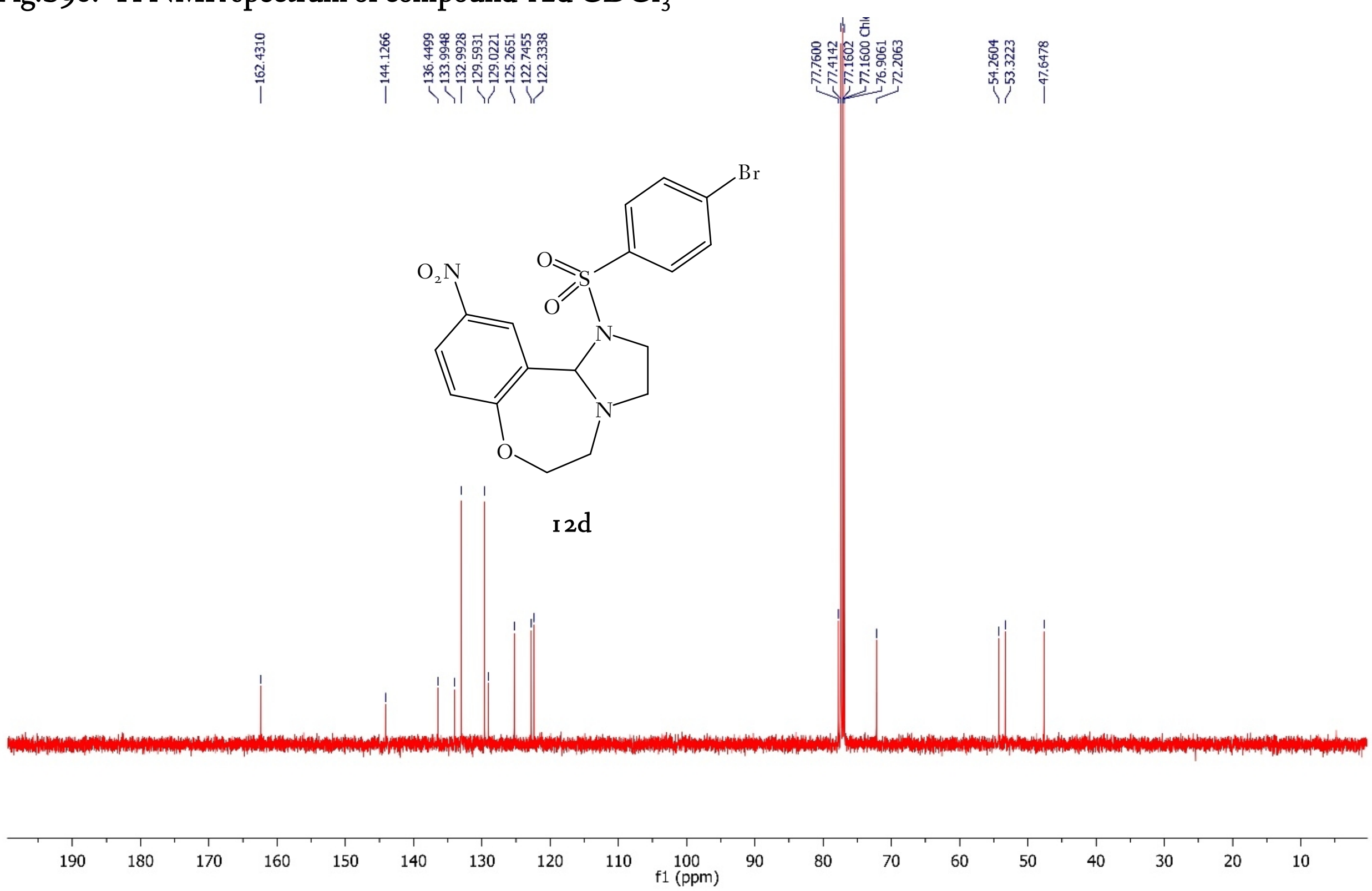


Fig.S99. ¹³C NMR spectrum of compound 12d $CDCl_3$

# The University of Jordan
## Faculty of science
## Department of Chemistry

## Mass Spectrum Molecular Formula Report

**Analysis Info**

| | | | |
|---|---|---|---|
| Analysis Name | F:\Data\2022MAR08\Mustafa_000048.d | Acquisition Date | 3/13/2022 2:22:54 PM |
| Method | ESI_pos_20181024 | Operator | Bruker_PC |
| Sample Name | AM308 | Instrument | apex-IV |
| Comment | CHCl3+ACN | | |

**Acquisition Parameter**

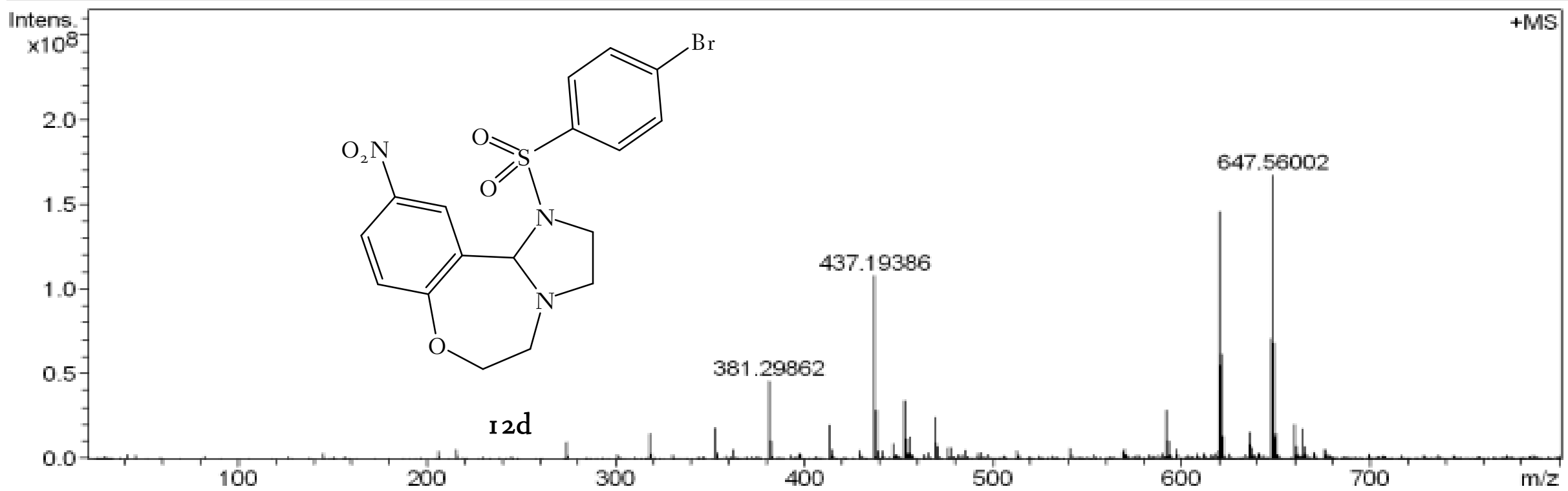


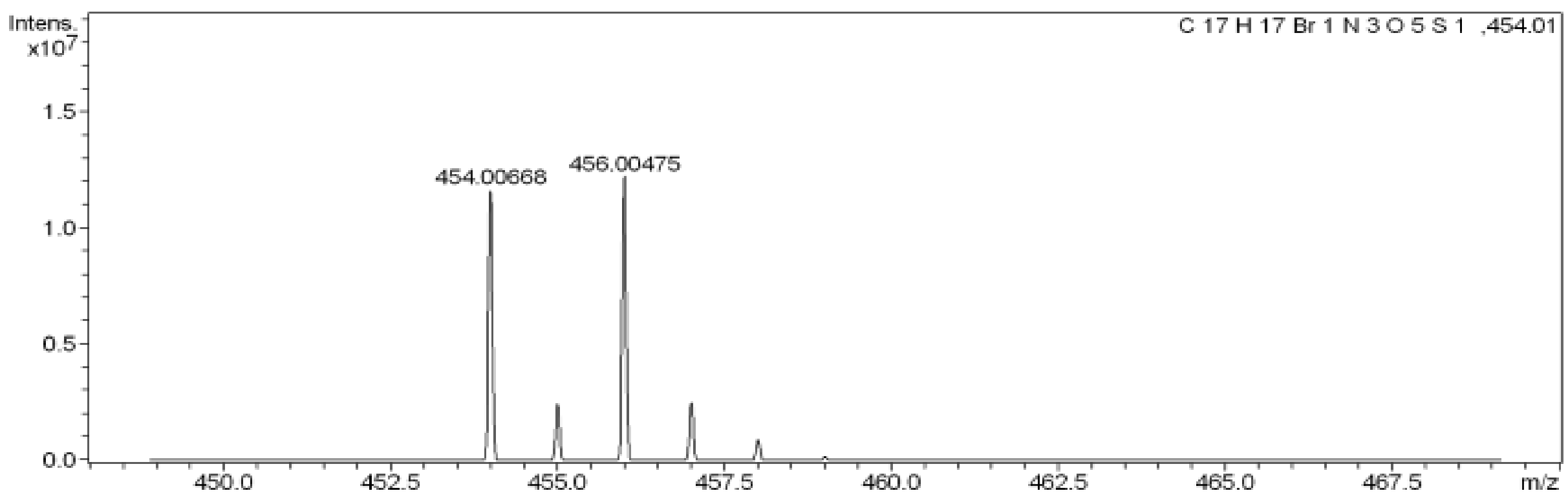


| Sum Formula | Sigma | m/z | Err [ppm] | Mean Err [ppm] | Err [mDa] | rdb | N Rule | e⁻ |
|---|---|---|---|---|---|---|---|---|
| C 17 H 17 Br 1 N 3 O 5 S 1 | 0.010 | 454.00668 | -2.34 | -2.94 | -1.06 | 10.50 | ok | even |

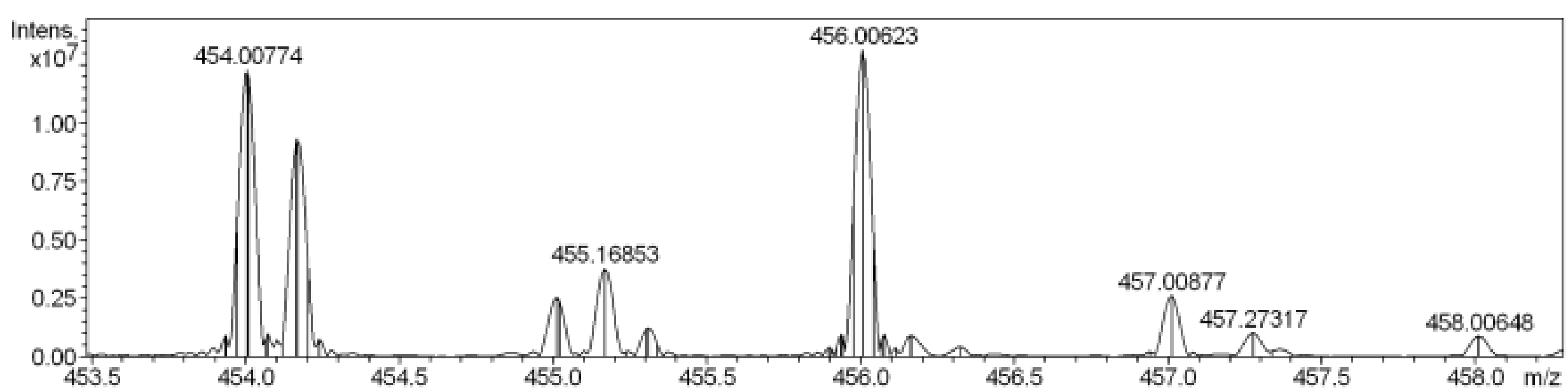


Fig.S100. HRMS spectrum of compound 12d

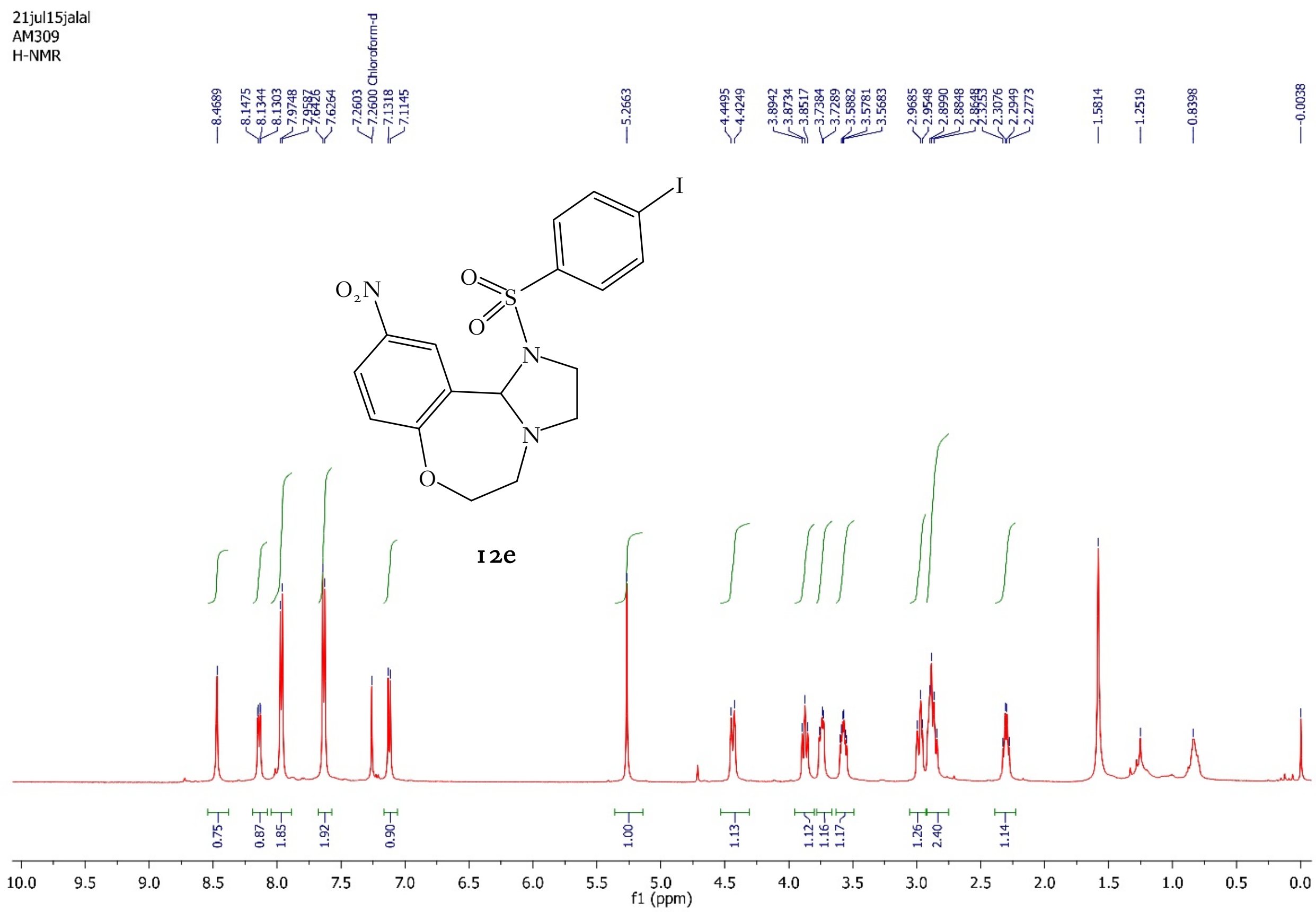


Fig.S101. $^1$H NMR spectrum of compound 12e $CDCl_3$

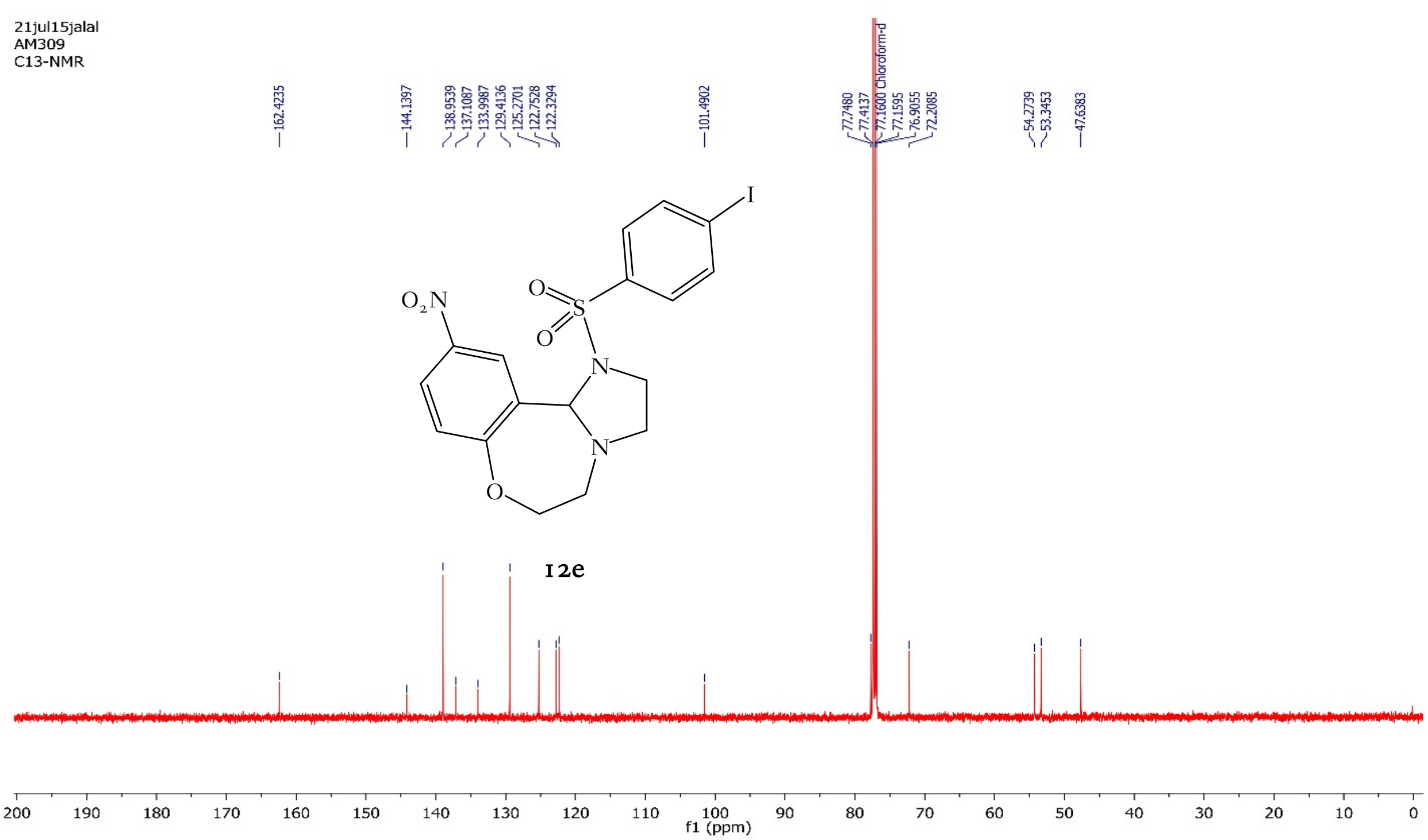

Fig.S102. $^{13}C$ NMR spectrum of compound 12e $CDCl_3$

**The University of Jordan**
**Faculty of science**
**Department of Chemistry**

## Mass Spectrum Molecular Formula Report

**Analysis Info**

| | | | |
|---|---|---|---|
| Analysis Name | F:\Data\2022MAR08\Mustafa_000050.d | Acquisition Date | 3/13/2022 2:30:13 PM |
| Method | ESI_pos_20181024 | Operator | Bruker_PC |
| Sample Name | AM309 | Instrument | apex-IV |
| Comment | CHCl3+ACN | | |

**Acquisition Parameter**

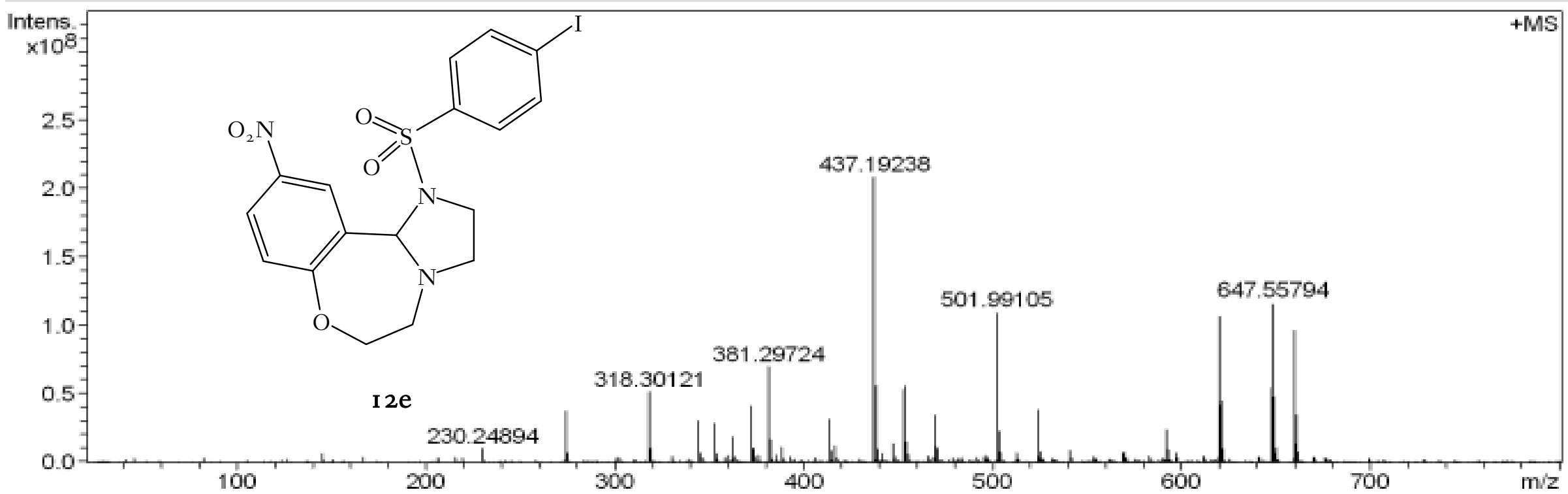


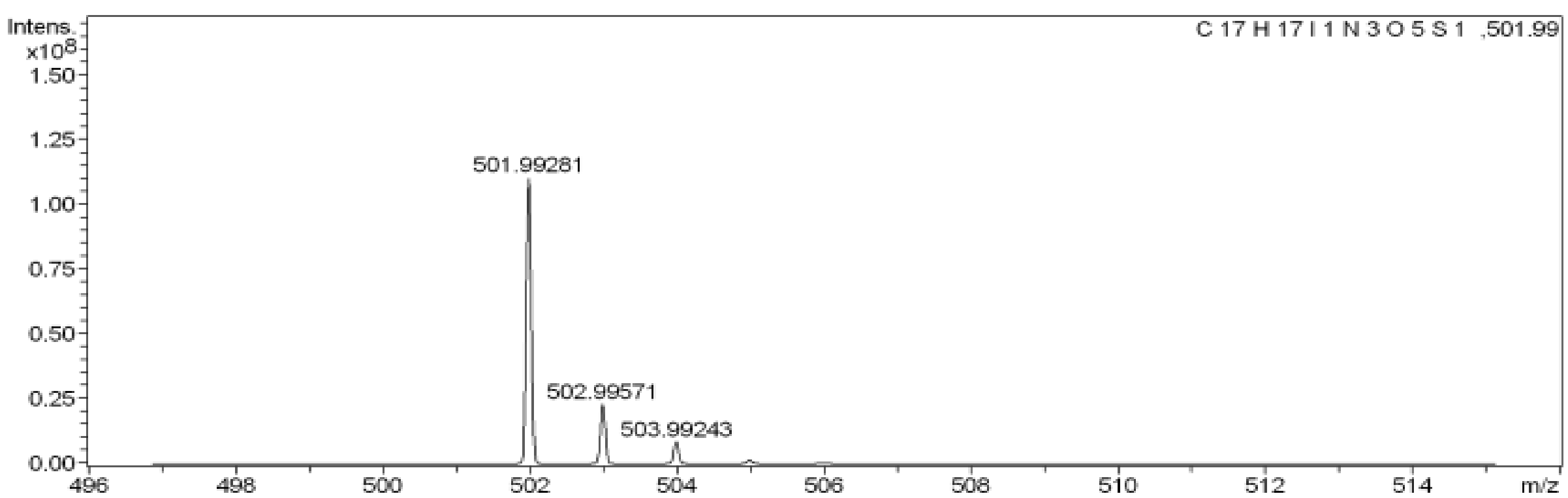


| Sum Formula | Sigma | m/z | Err [ppm] | Mean Err [ppm] | Err [mDa] | rdb | N Rule | e⁻ |
|---|---|---|---|---|---|---|---|---|
| C 17 H 17 I 1 N 3 O 5 S 1 | 0.004 | 501.99281 | 3.51 | 2.88 | 1.76 | 10.50 | ok | even |

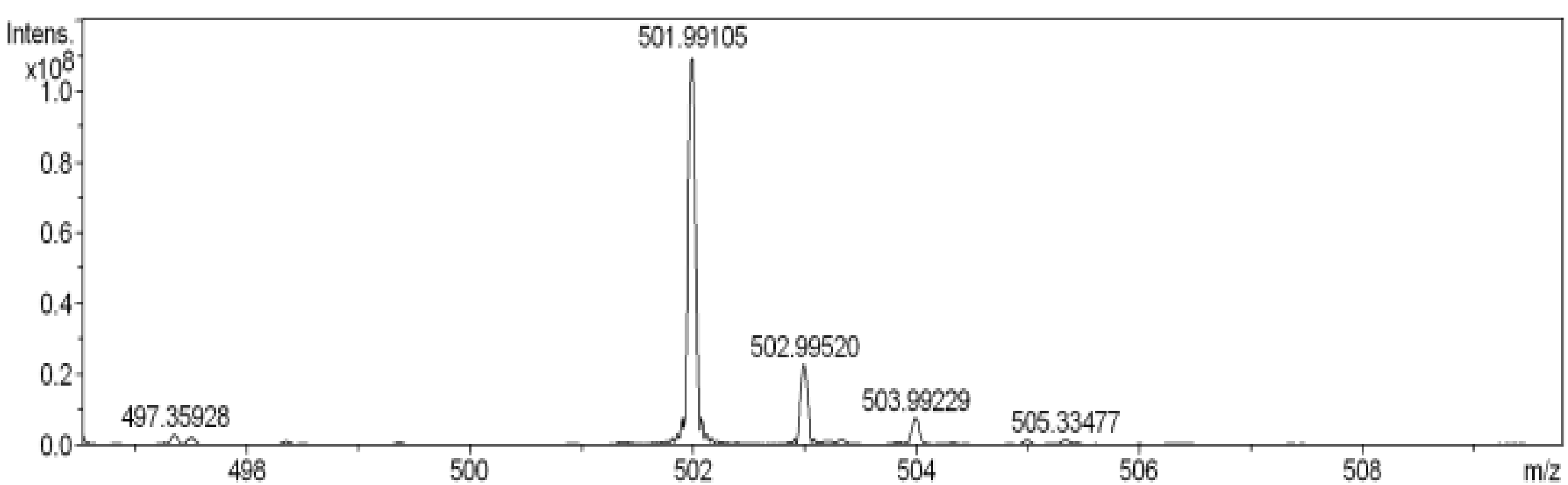

Fig.S103. HRMS spectrum of compound 12e

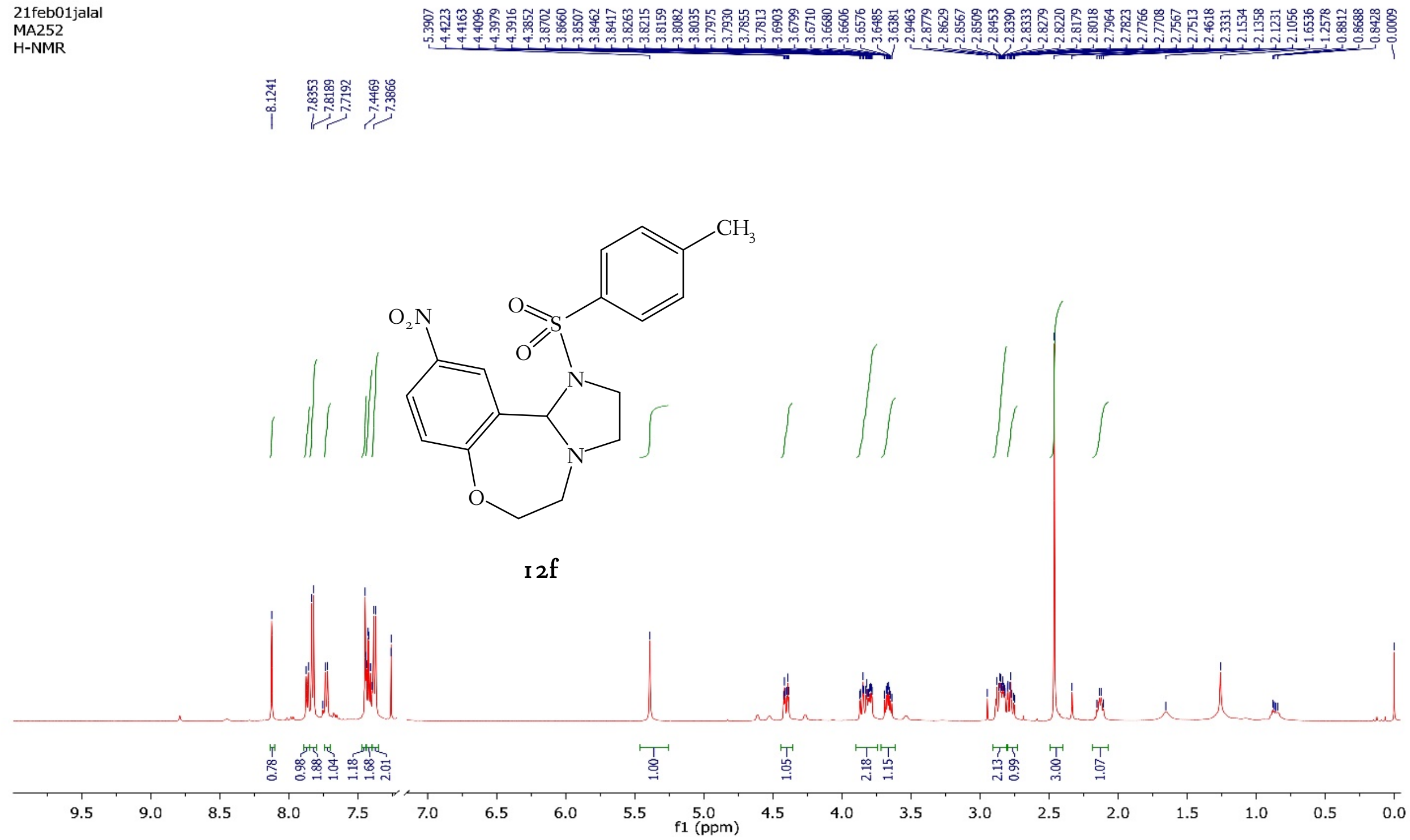


Fig.S104. $^{1}$H NMR spectrum of compound 12f $CDCl_3$

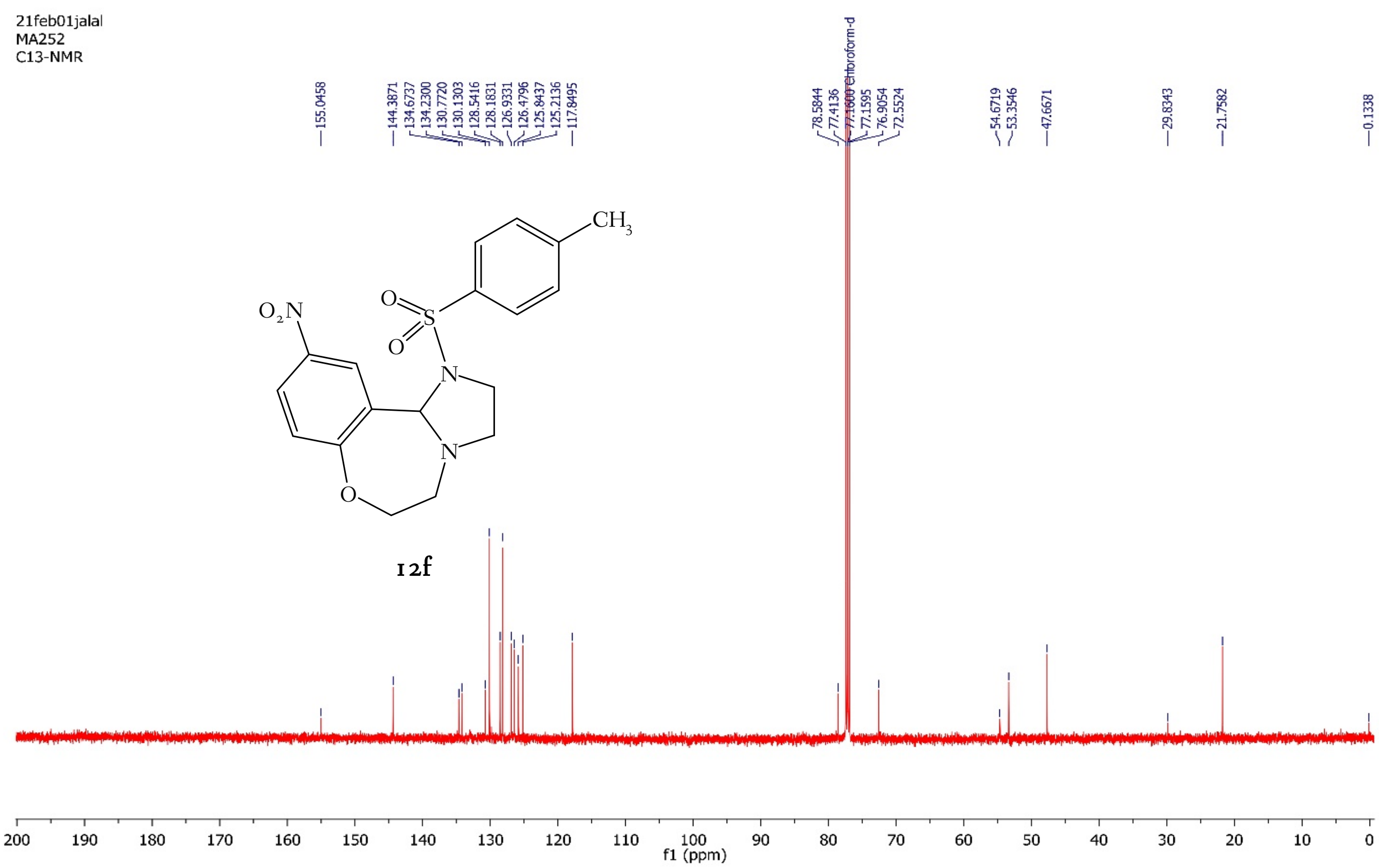

Fig.S105. $^{13}C$ NMR spectrum of compound 12f $CDCl_3$

**The University of Jordan**
**Faculty of science**
**Department of Chemistry**

## Mass Spectrum Molecular Formula Report

**Analysis Info**

| | | | |
|---|---|---|---|
| Analysis Name | F:\Data\2022MAR20\Mustafa_000061.d | Acquisition Date | 3/23/2022 2:35:24 PM |
| Method | ESI_pos_20181024 | Operator | Bruker_PC |
| Sample Name | AM252 | Instrument | apex-IV |
| Comment | CHCl3+ACN | | |

**Acquisition Parameter**

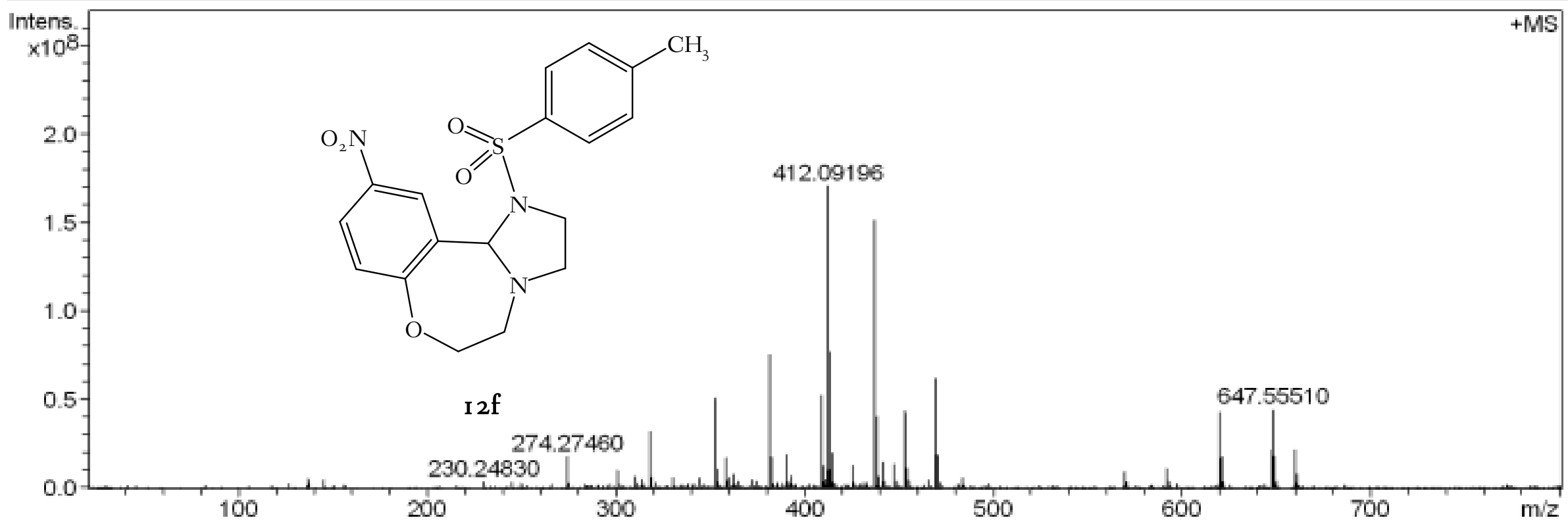


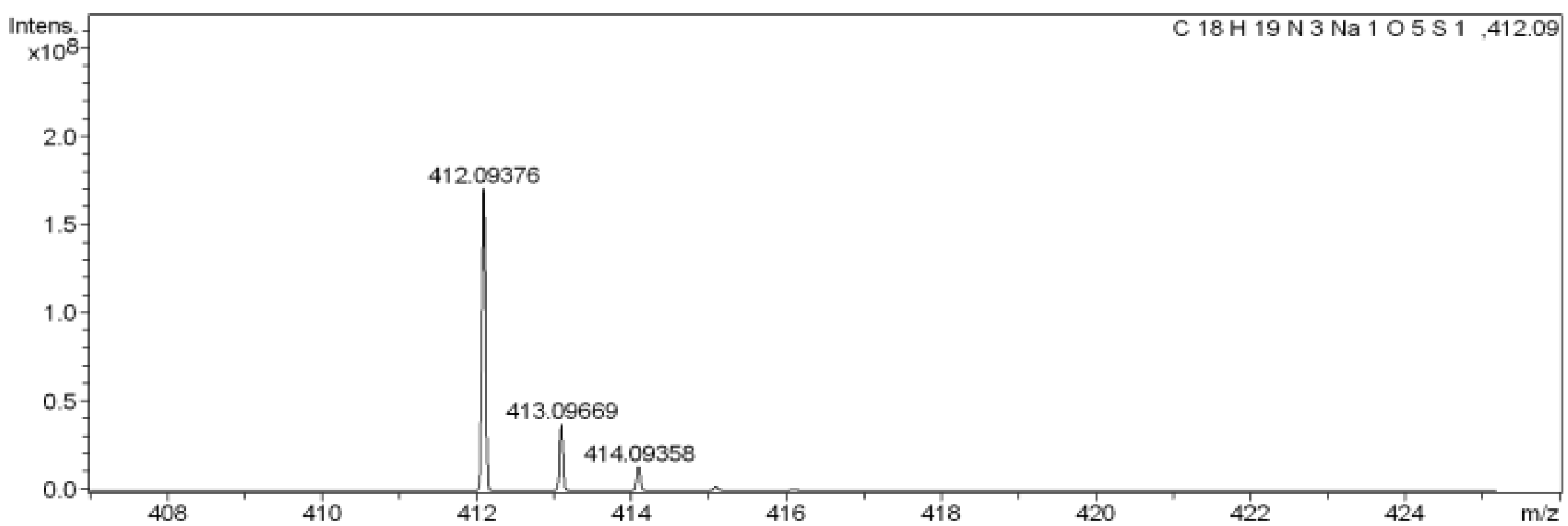


| Sum Formula | Sigma | m/z | Err [ppm] | Mean Err [ppm] | Err [mDa] | rdb | N Rule | e⁻ |
|---|---|---|---|---|---|---|---|---|
| C 18 H 19 N 3 Na 1 O 5 S 1 | 0.008 | 412.09376 | 4.38 | 4.83 | 1.80 | 10.50 | ok | even |

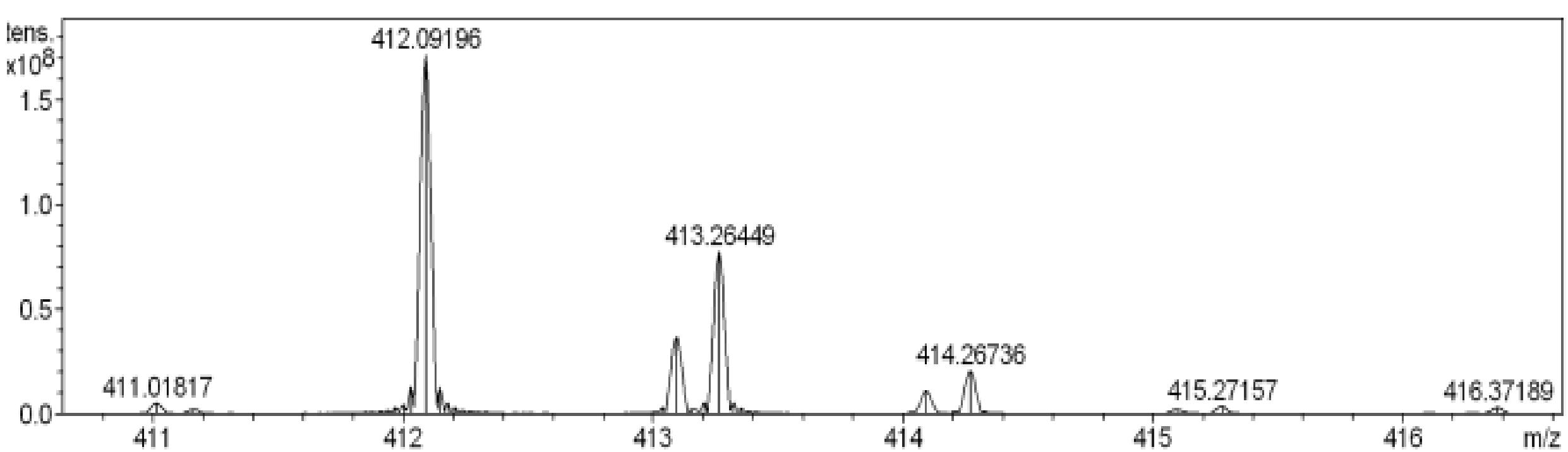

Fig.S106. HRMS spectrum of compound 12f

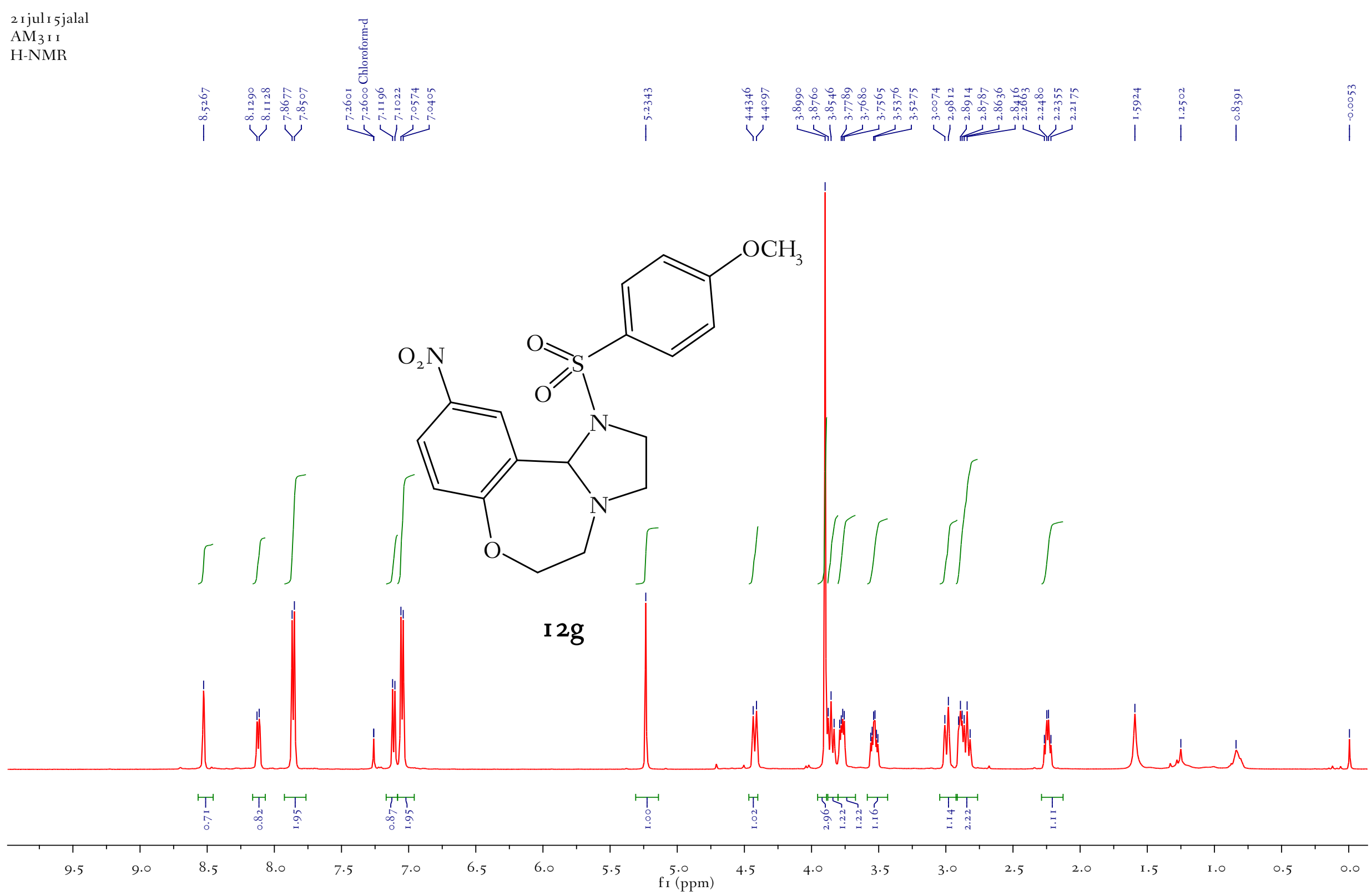


Fig.S107. $^{1}$H NMR spectrum of compound 12g $CDCl_3$

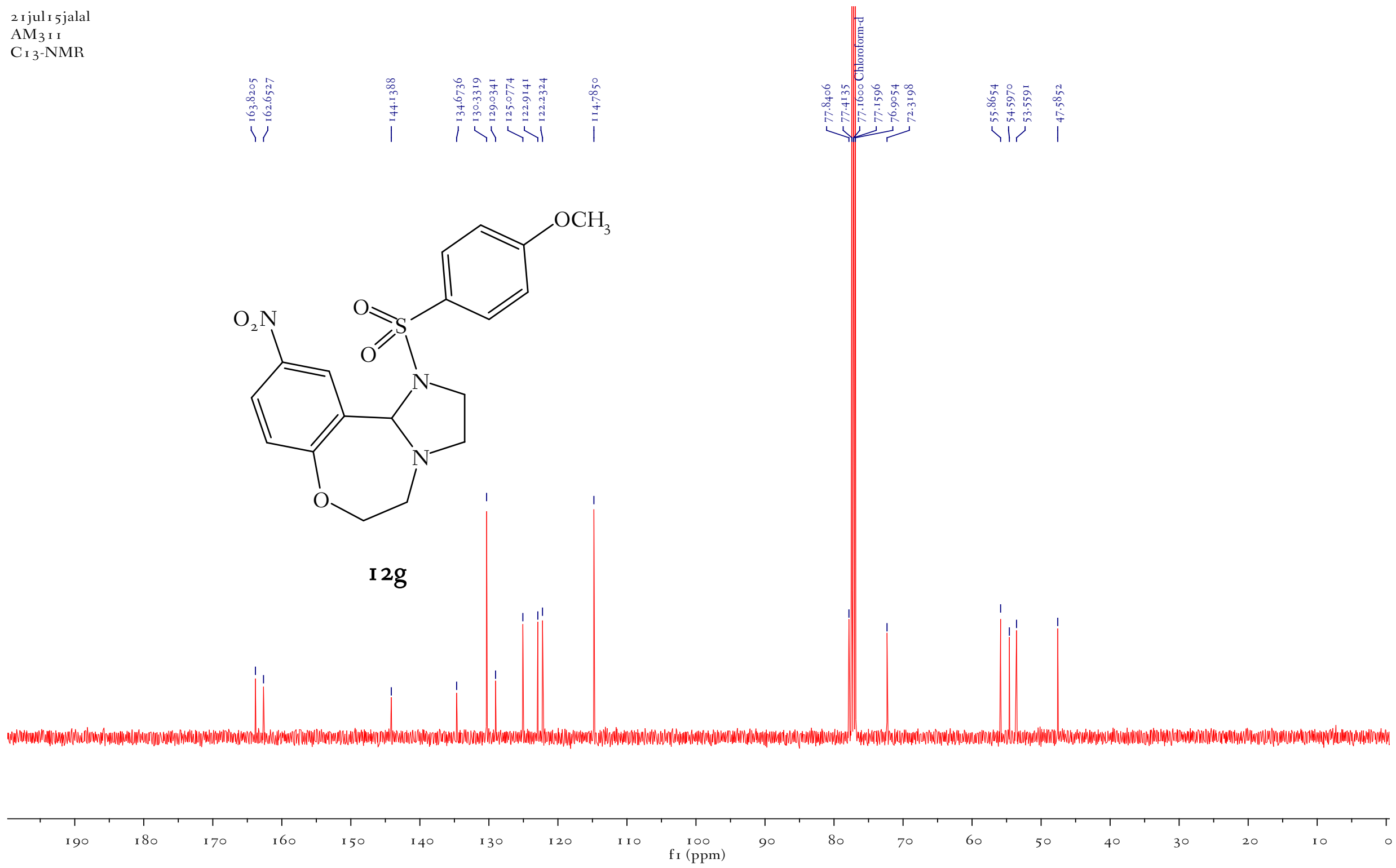


Fig.S108. $^{13}$C NMR spectrum of compound 12g $CDCl_3$

# The University of Jordan
## Faculty of science
## Department of Chemistry

# Mass Spectrum Molecular Formula Report

**Analysis Info**

| | | | |
|---|---|---|---|
| Analysis Name | F:\Data\2022MAR14\Mustafa_000034.d | Acquisition Date | 3/15/2022 11:51:50 AM |
| Method | ESI_pos_20181024 | Operator | Bruker_PC |
| Sample Name | AM311 | Instrument | apex-IV |
| Comment | CHCl3+ACN | | |

**Acquisition Parameter**

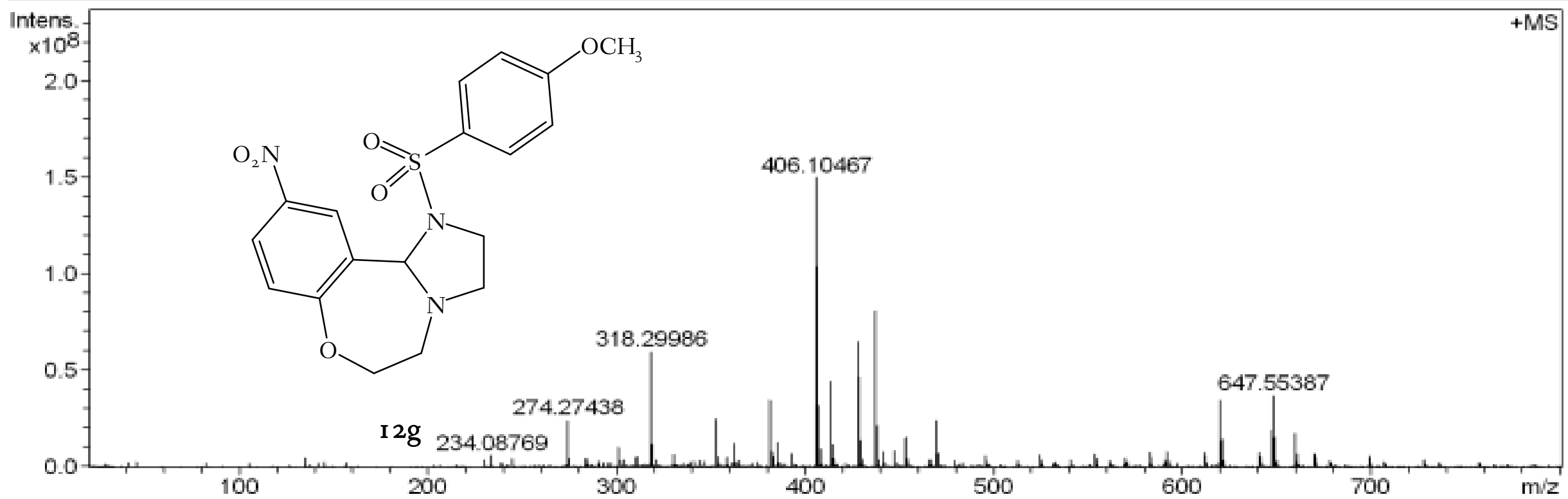


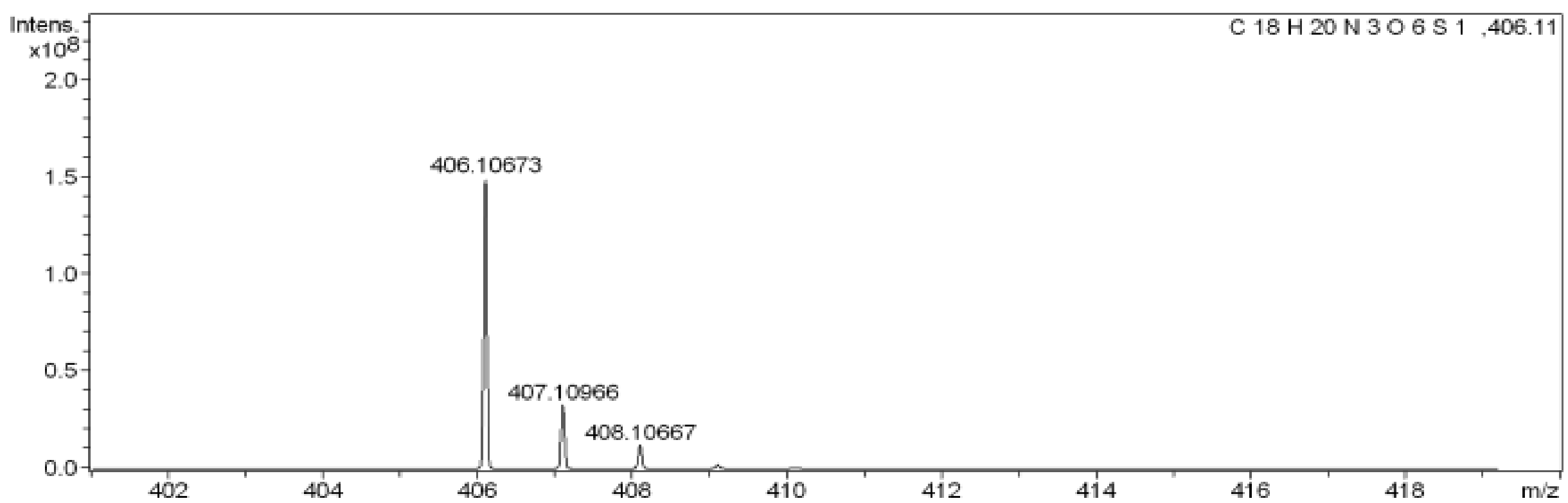


| Sum Formula | Sigma | m/z | Err [ppm] | Mean Err [ppm] | Err [mDa] | rdb | N Rule | e⁻ |
|---|---|---|---|---|---|---|---|---|
| C 18 H 20 N 3 O 6 S 1 | 0.009 | 406.10673 | 5.08 | 5.08 | 2.06 | 10.50 | ok | even |

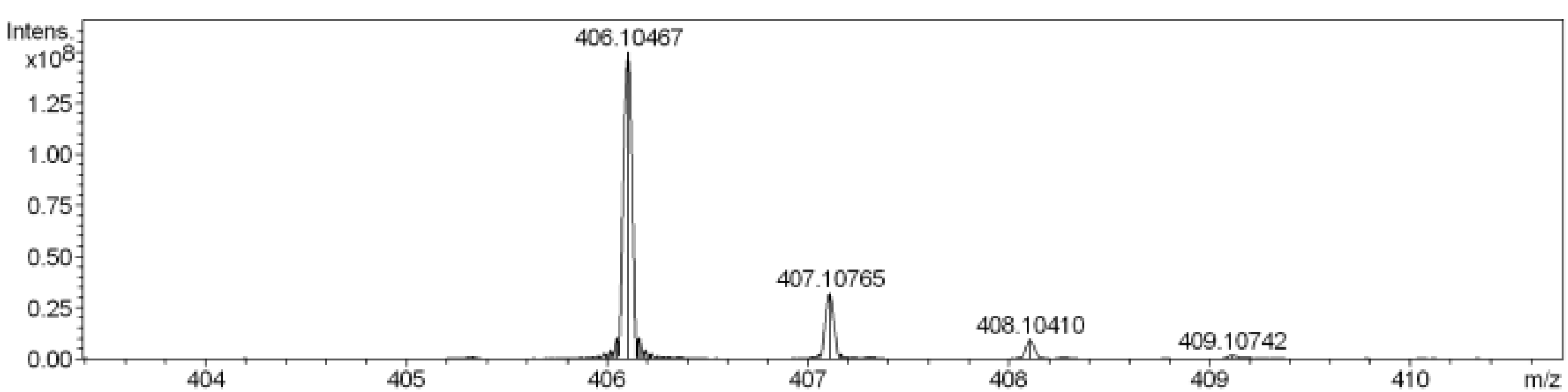


Fig.S109. HRMS spectrum of compound 12g

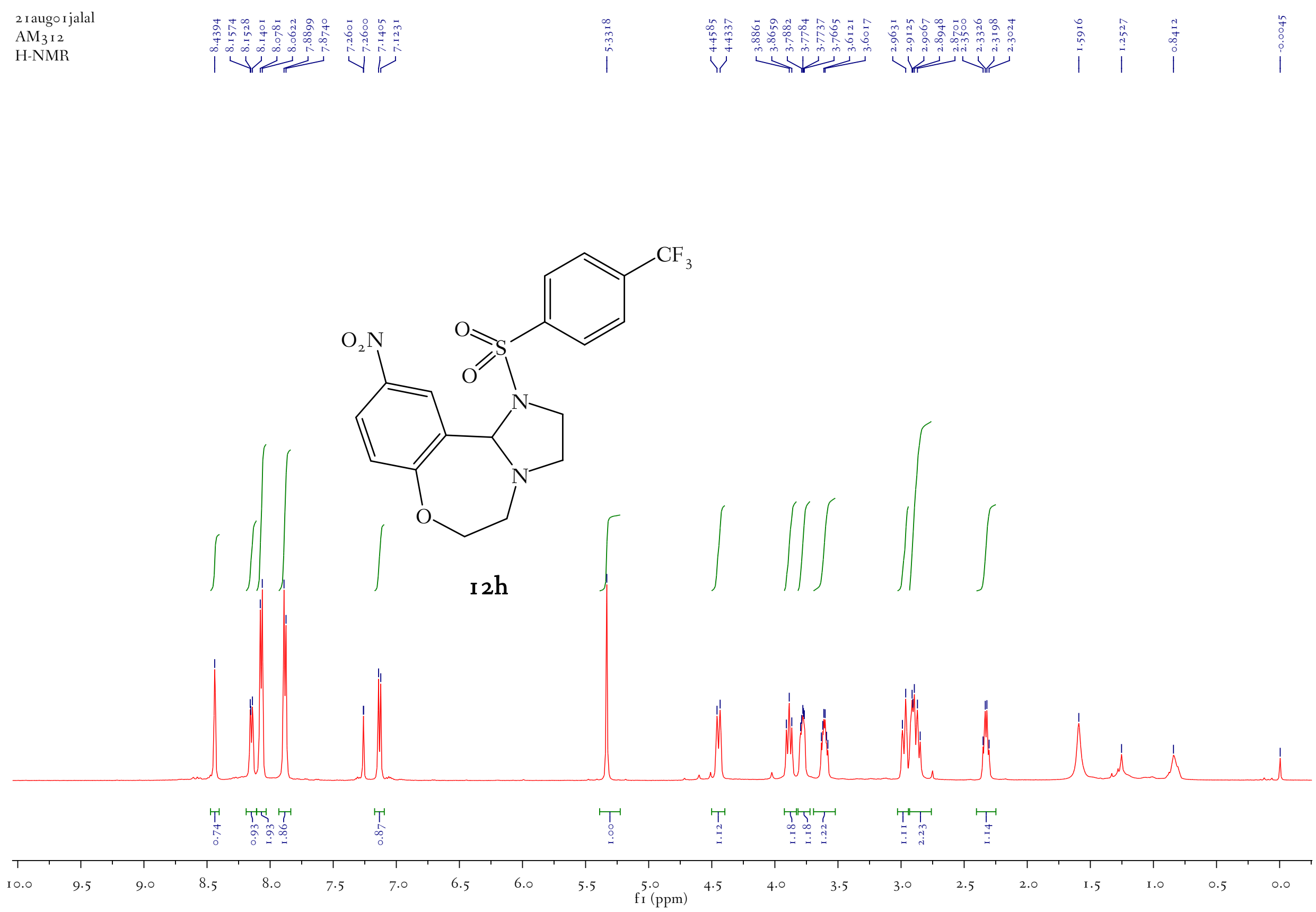


Fig.S110. $^{1}H$ NMR spectrum of compound 12h $CDCl_3$

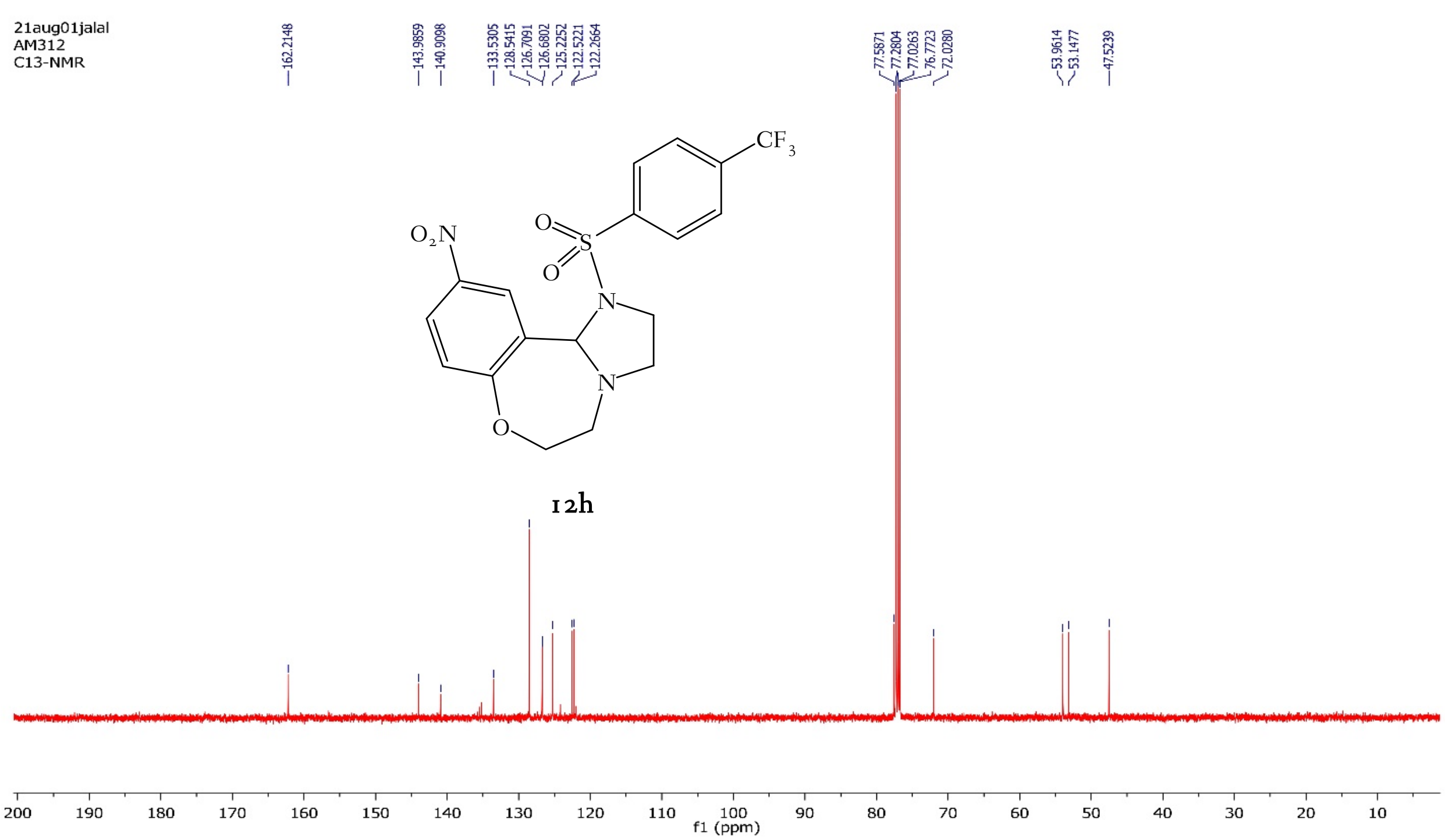


Fig.S111. $^{13}C$ NMR spectrum of compound 12h $CDCl_3$

# The University of Jordan
## Faculty of science
## Department of Chemistry

## Mass Spectrum Molecular Formula Report

**Analysis Info**

| | | | |
|---|---|---|---|
| Analysis Name | F:\Data\2022MAR14\Mustafa_000007.d | Acquisition Date | 3/14/2022 1:06:21 PM |
| Method | ESI_pos_20181024 | Operator | Bruker_PC |
| Sample Name | AM312 | Instrument | apex-IV |
| Comment | CHCl3+ACN | | |

**Acquisition Parameter**

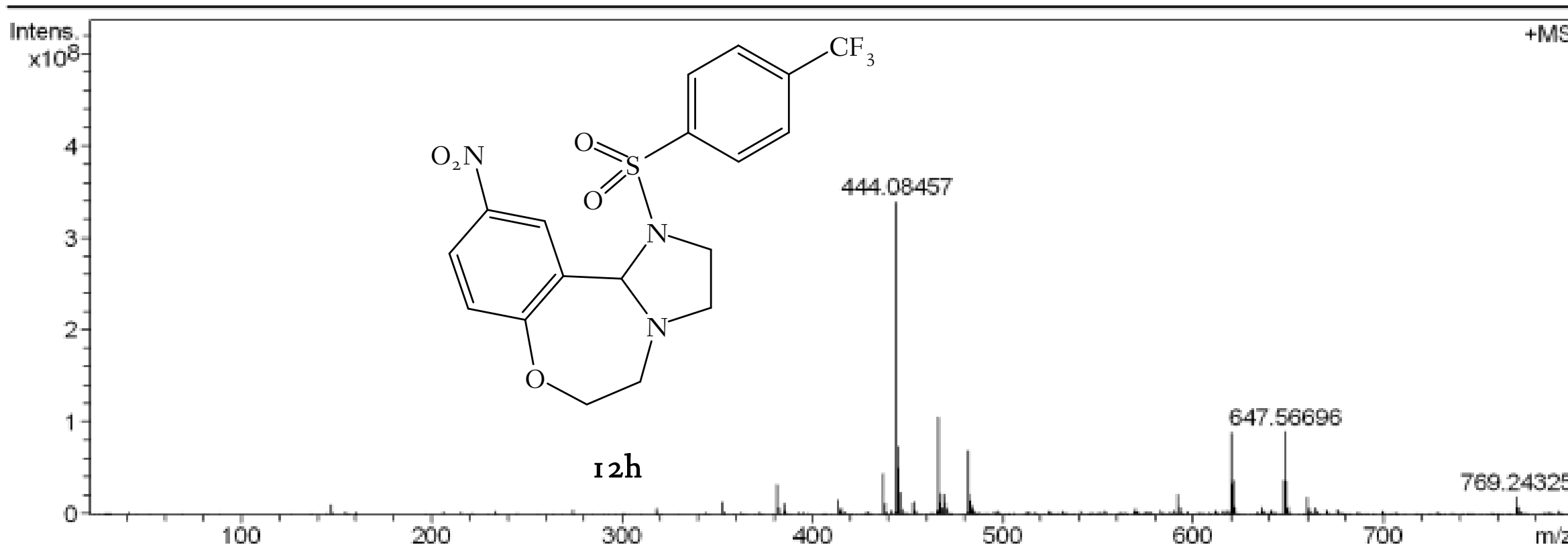


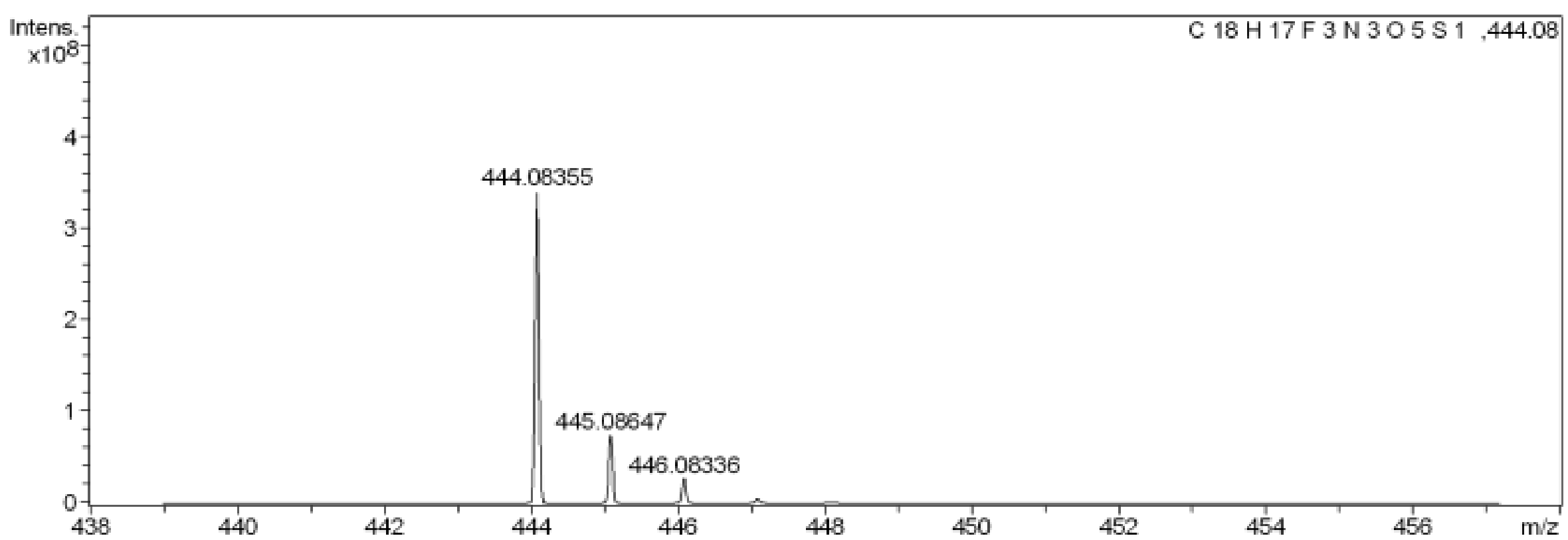


| Sum Formula | Sigma | m/z | Err [ppm] | Mean Err [ppm] | Err [mDa] | rdb | N Rule | e⁻ |
|---|---|---|---|---|---|---|---|---|
| C 18 H 17 F 3 N 3 O 5 S 1 | 0.003 | 444.08355 | -2.29 | -2.43 | -1.02 | 10.50 | ok | even |

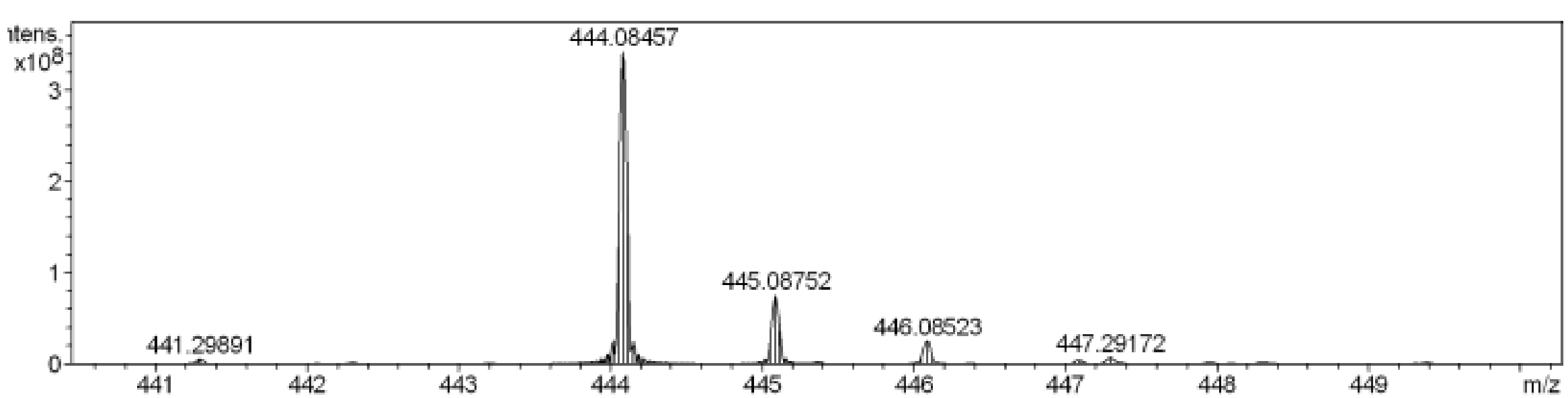


Fig.S112. HRMS spectrum of compound 12h

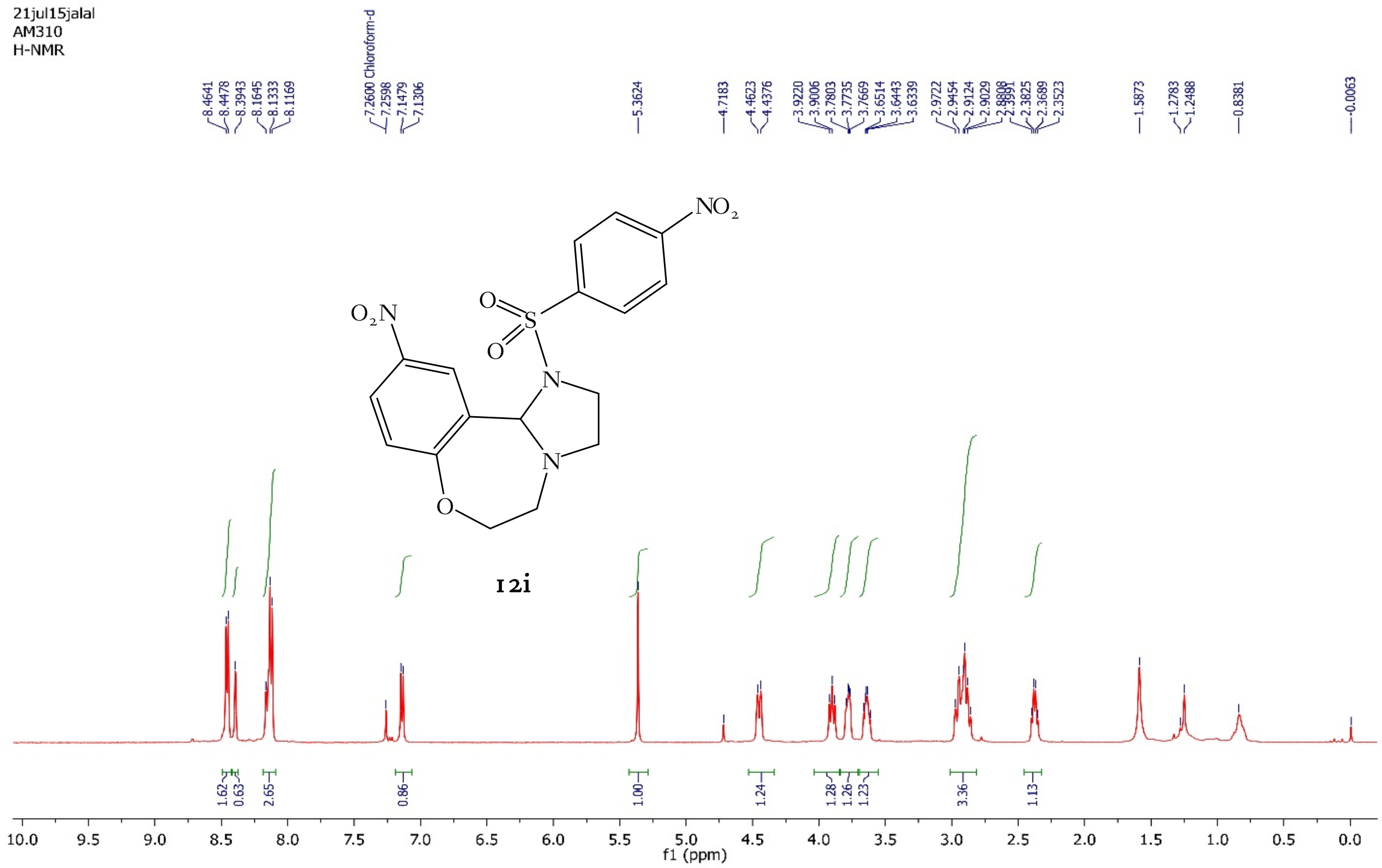


Fig.S113. $^{1}$H NMR spectrum of compound 12i $CDCl_3$

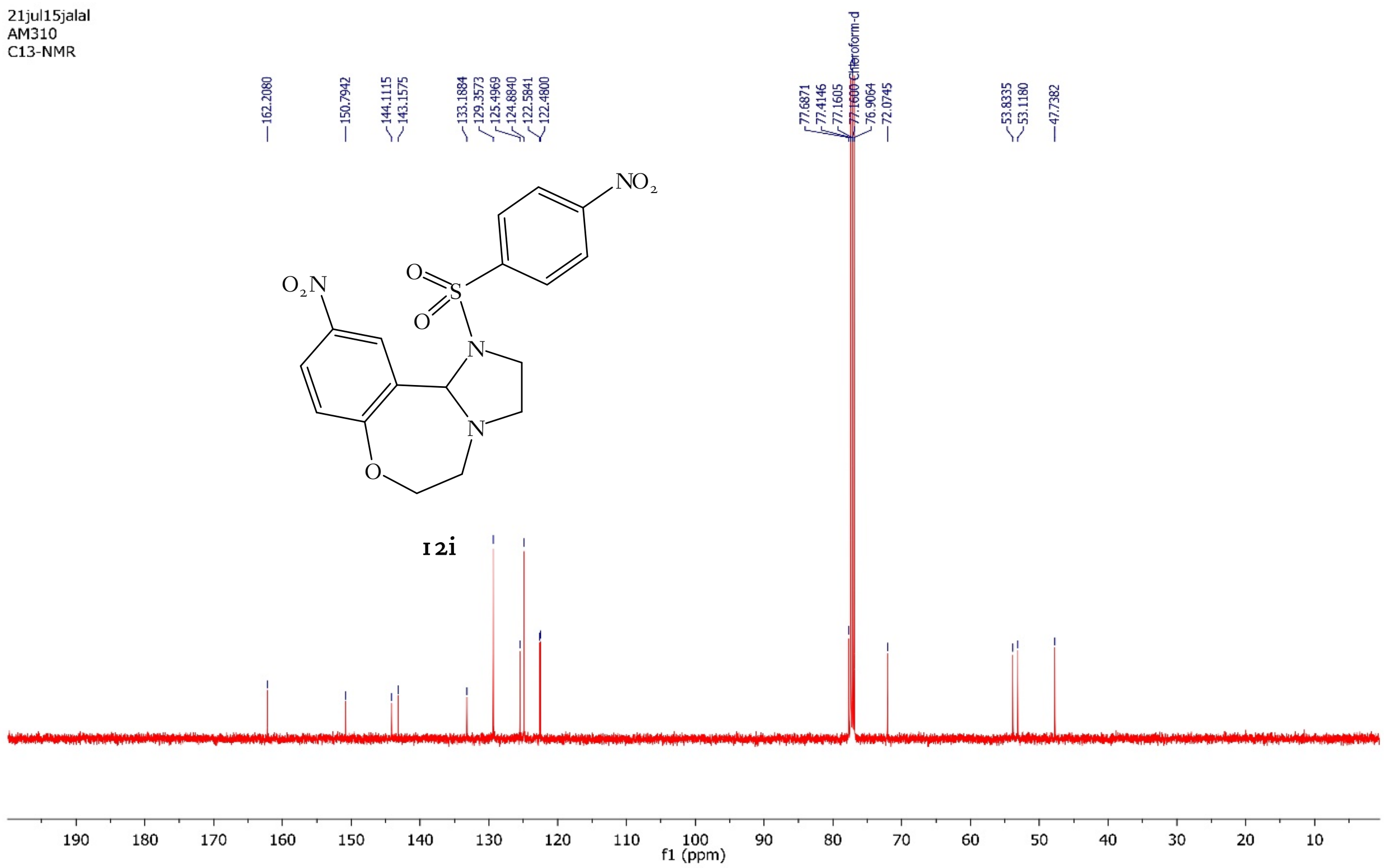


Fig.S114. $^{13}$C NMR spectrum of compound 12i $CDCl_3$

# The University of Jordan
## Faculty of science
## Department of Chemistry

### Mass Spectrum Molecular Formula Report

**Analysis Info**

| | | | |
|---|---|---|---|
| Analysis Name | F:\Data\2022MAR14\Mustafa_000029.d | Acquisition Date | 3/15/2022 11:28:41 AM |
| Method | ESI_pos_20181024 | Operator | Bruker_PC |
| Sample Name | AM310 | Instrument | apex-IV |
| Comment | CHCl3+ACN | | |

**Acquisition Parameter**

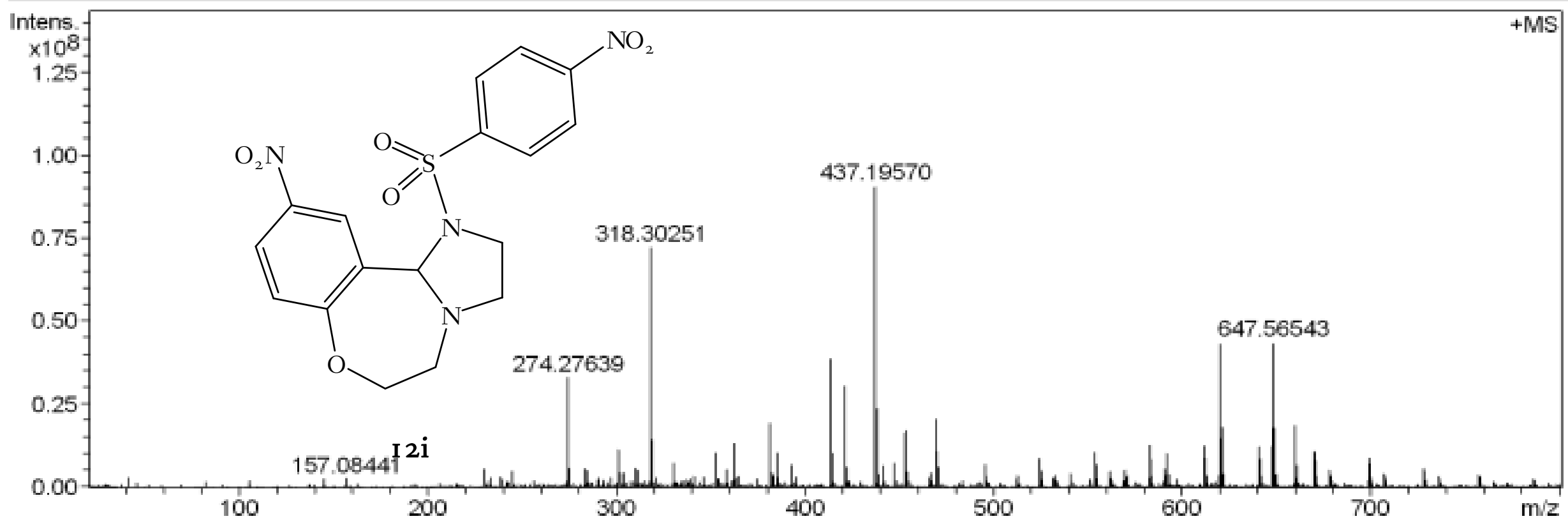


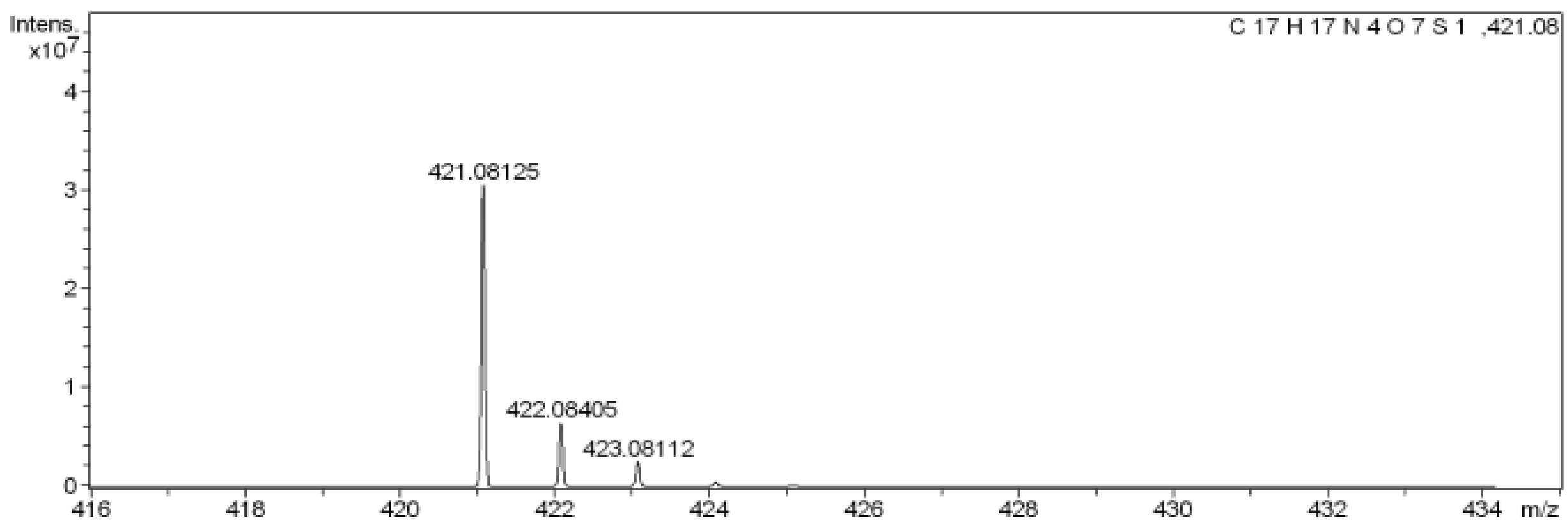


| Sum Formula | Sigma | m/z | Err [ppm] | Mean Err [ppm] | Err [mDa] | rdb | N Rule | e⁻ |
|---|---|---|---|---|---|---|---|---|
| C 17 H 17 N 4 O 7 S 1 | 0.008 | 421.08125 | -6.21 | -6.11 | -2.62 | 11.50 | ok | even |

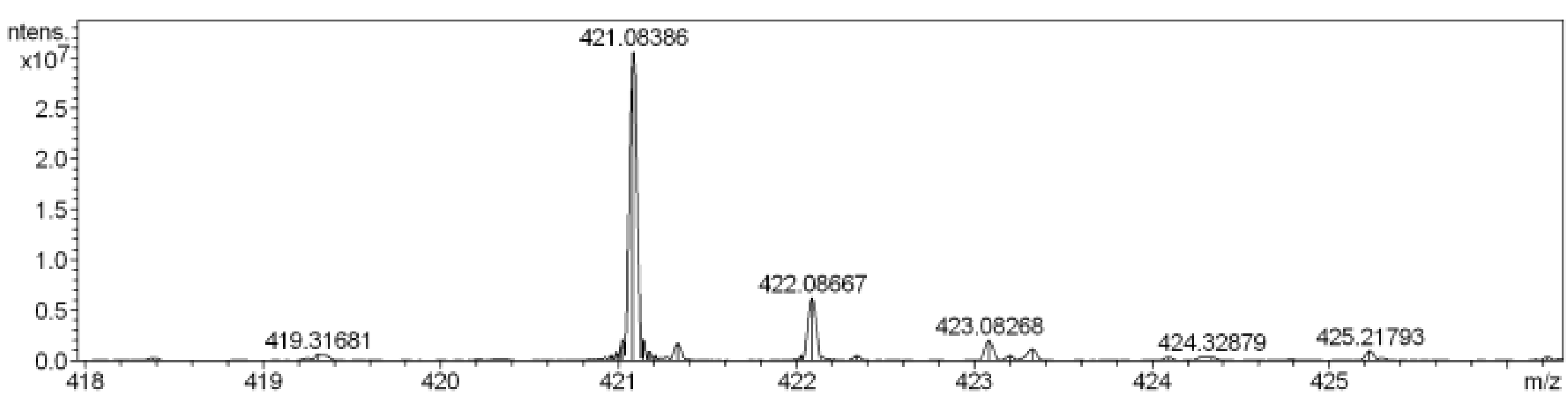


Fig.S115. HRMS spectrum of compound 12i

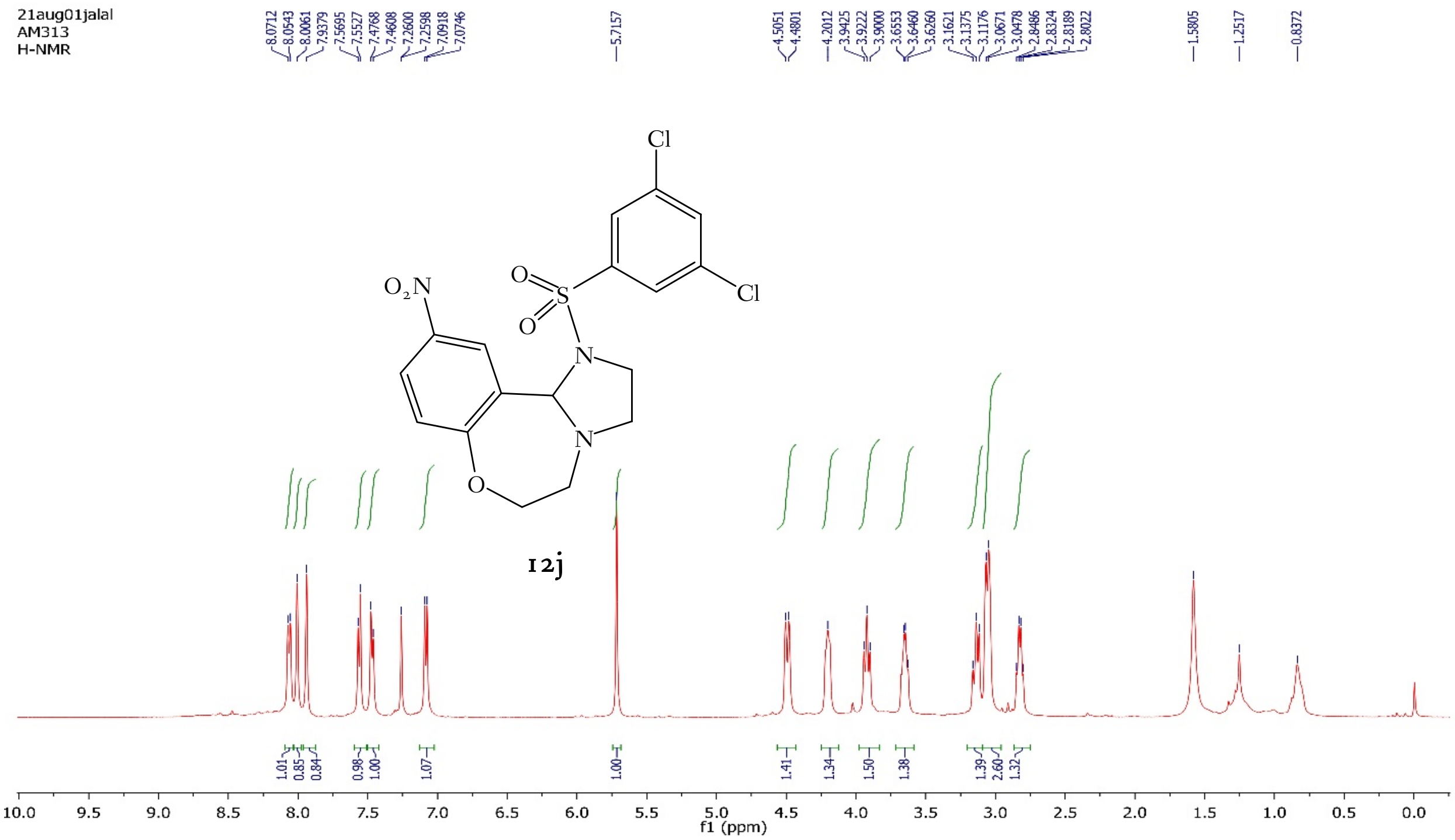


Fig.S116. $^{1}$H NMR spectrum of compound 12j $CDCl_3$

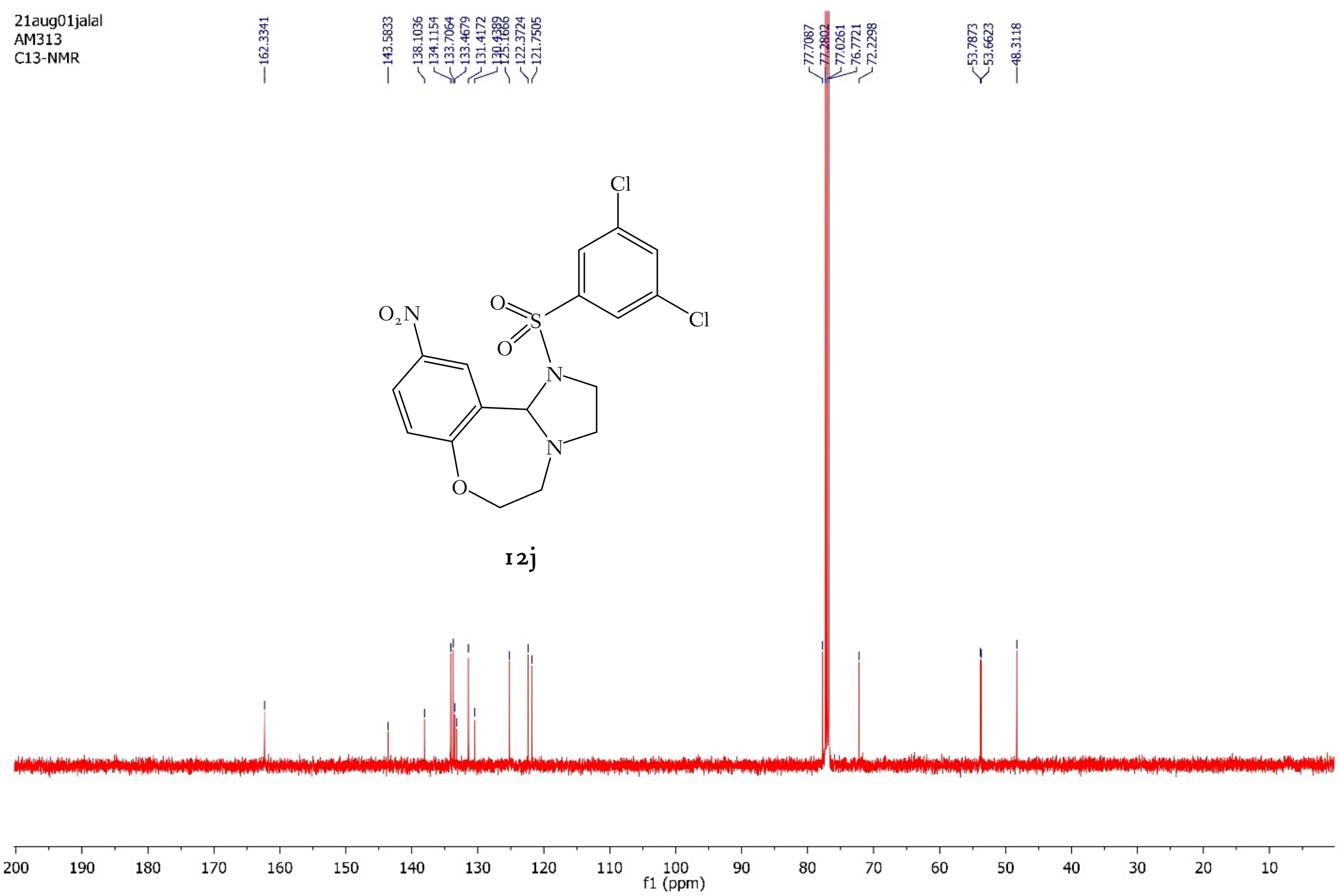


Fig.S117. $^{13}$C NMR spectrum of compound 12j $CDCl_3$

**The University of Jordan**

**Faculty of science**

**Department of Chemistry**

## Mass Spectrum Molecular Formula Report

**Analysis Info**

| | | | |
|---|---|---|---|
| Analysis Name | F:\Data\2022MAR14\Mustafa_000008.d | Acquisition Date | 3/14/2022 1:11:28 PM |
| Method | ESI_pos_20181024 | Operator | Bruker_PC |
| Sample Name | AM313 | Instrument | apex-IV |
| Comment | CHCl3+ACN | | |

**Acquisition Parameter**

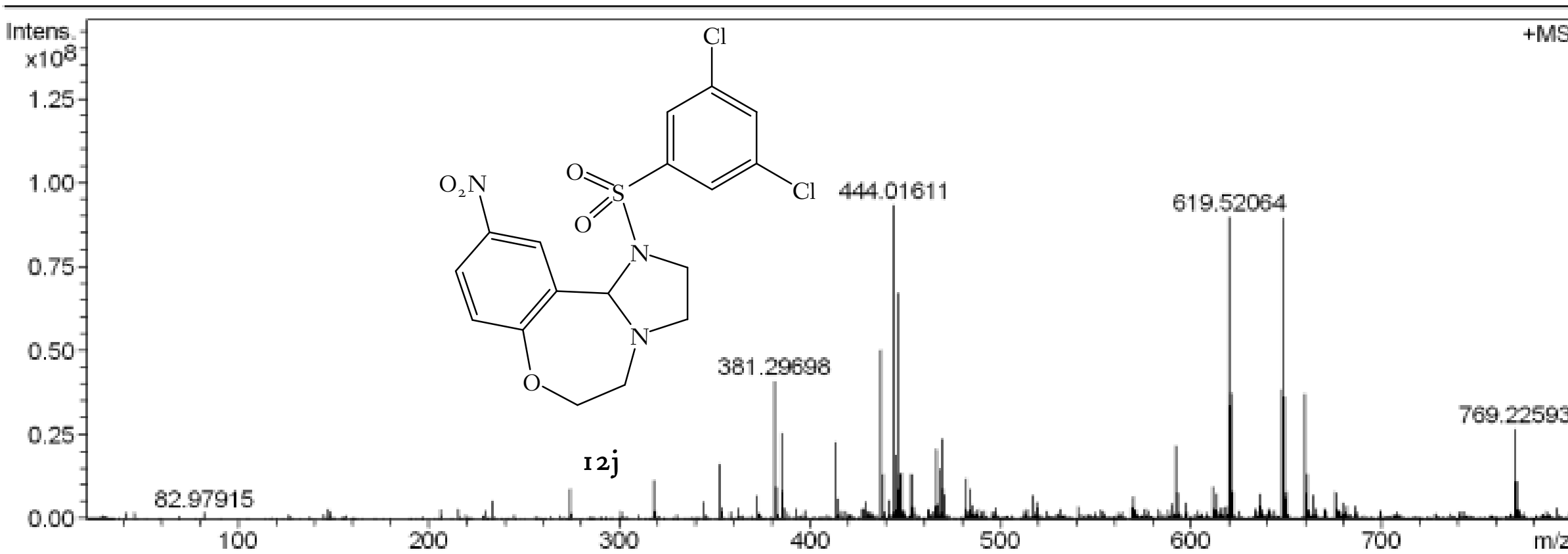


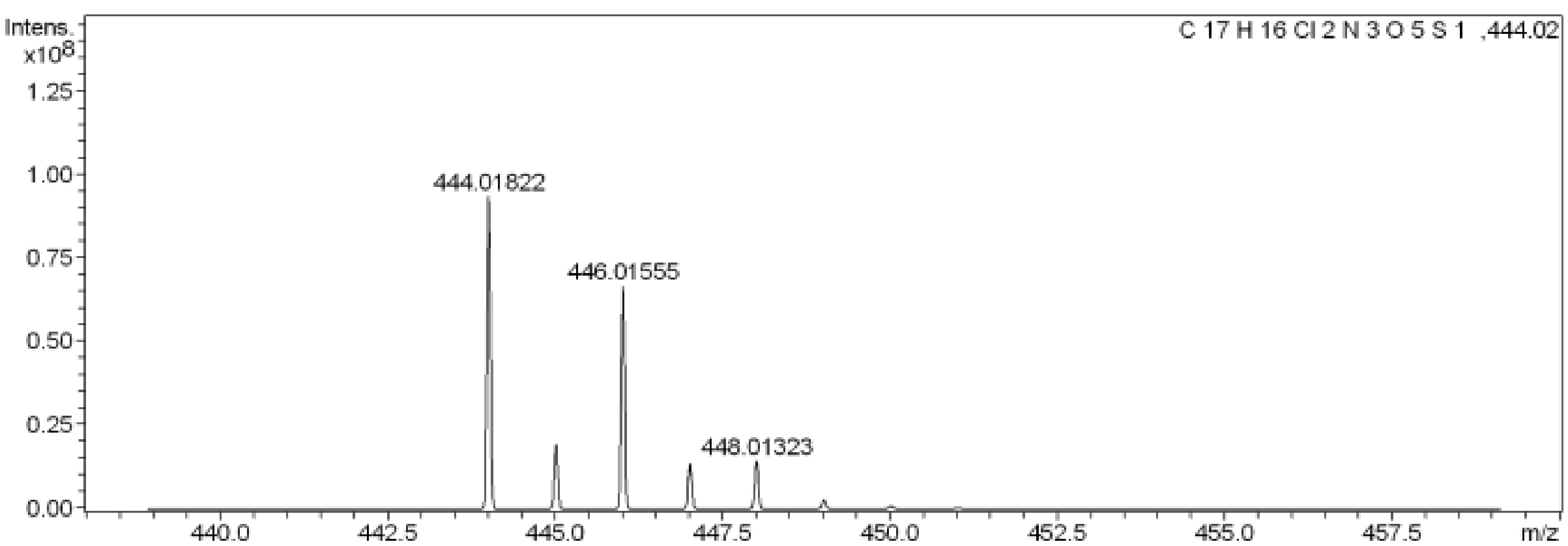


| Sum Formula | Sigma | m/z | Err [ppm] | Mean Err [ppm] | Err [mDa] | rdb | N Rule | e⁻ |
|---|---|---|---|---|---|---|---|---|
| C 17 H 16 Cl 2 N 3 O 5 S 1 | 0.005 | 444.01822 | 4.75 | 3.92 | 2.11 | 10.50 | ok | even |

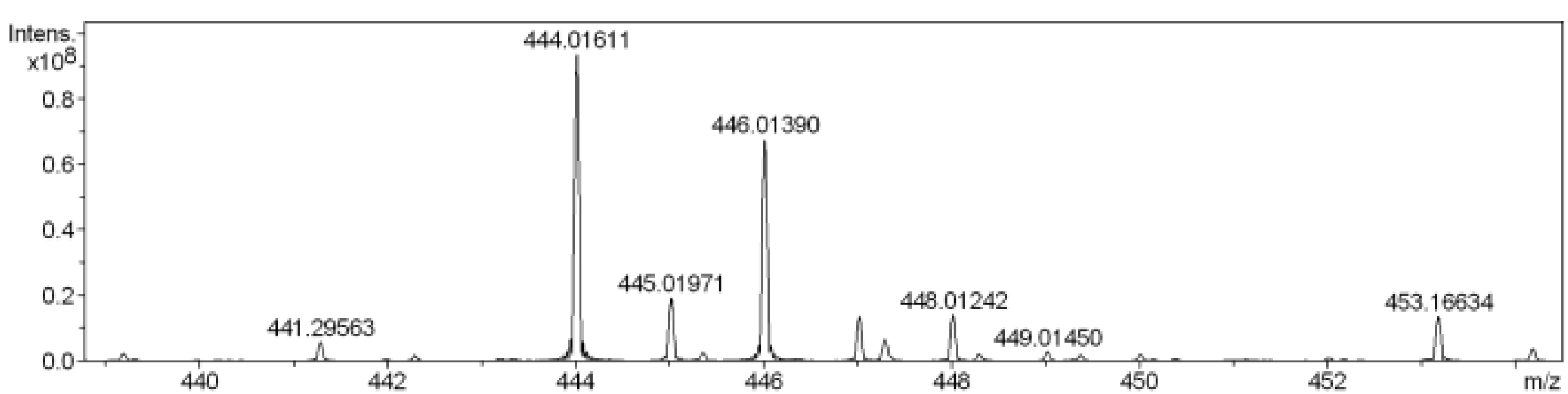


Fig.S118. HRMS spectrum of compound 12j

**Crystal data and structure refinement for compound 11f**

**exp_3182_auto**

**Table 1 Crystal data and structure refinement for exp_3182_auto.**

| | |
|---|---|
| Identification code | exp_3182_auto |
| Empirical formula | $C_{18}H_{19}BrN_2O_3S$ |
| Formula weight | 423.32 |
| Temperature/K | 293(2) |
| Crystal system | monoclinic |
| Space group | $P2_1/n$ |
| a/Å | 6.9075(3) |
| b/Å | 21.2837(8) |
| c/Å | 13.0726(7) |
| α/° | 90 |
| β/° | 102.818(5) |
| γ/° | 90 |
| Volume/Å$^3$ | 1874.01(15) |
| Z | 4 |
| ρ$_{calc}$g/cm$^3$ | 1.500 |
| μ/mm$^{-1}$ | 2.324 |
| F(000) | 864.0 |
| Crystal size/mm$^3$ | 0.535 × 0.228 × 0.127 |
| Radiation | Mo Kα (λ = 0.71073) |
| 2Θ range for data collection/° | 6.344 to 58.786 |
| Index ranges | -9 ≤ h ≤ 9, -24 ≤ k ≤ 29, -16 ≤ l ≤ 16 |

| | |
|---|---|
| Reflections collected | 8969 |
| Independent reflections | 4333 [$R_{int}$ = 0.0290, $R_{sigma}$ = 0.0634] |
| Data/restraints/parameters | 4333/0/275 |
| Goodness-of-fit on $F^2$ | 1.003 |
| Final R indexes [I>=2σ (I)] | $R_1$ = 0.0463, $wR_2$ = 0.0904 |
| Final R indexes [all data] | $R_1$ = 0.0932, $wR_2$ = 0.1078 |
| Largest diff. peak/hole / e Å$^{-3}$ | 0.42/-0.62 |

**Table 2 Fractional Atomic Coordinates (×10$^4$) and Equivalent Isotropic Displacement Parameters (Å$^2$×10$^3$) for exp_3182_auto. $U_{eq}$ is defined as 1/3 of the trace of the orthogonalised $U_{IJ}$ tensor.**

| Atom | *x* | *y* | *z* | U(eq) |
|---|---|---|---|---|
| Br1 | 9169.1(6) | 9177.0(2) | 3910.8(3) | 66.96(17) |
| S1 | 8596.5(11) | 6630.1(4) | 5613.0(7) | 48.6(2) |
| O1 | 3794(3) | 8279.8(10) | 6656.8(19) | 54.6(6) |
| O3 | 10535(3) | 6426.2(11) | 5538(2) | 76.0(8) |
| C9 | 7161(4) | 7532.9(13) | 6648(2) | 35.5(7) |
| C4 | 5076(4) | 8478.7(14) | 6056(2) | 39.8(7) |
| C3 | 4580(5) | 9010.7(15) | 5445(3) | 49.3(9) |
| N2 | 8925(3) | 7188.2(11) | 6492(2) | 41.4(6) |
| C2 | 5792(5) | 9216.0(15) | 4808(3) | 51.0(9) |
| C5 | 6797(4) | 8137.1(13) | 6025(2) | 34.6(7) |
| O2 | 7254(3) | 6884.6(10) | 4714.9(17) | 57.5(6) |
| C6 | 8028(4) | 8356.1(14) | 5401(2) | 39.6(7) |
| C10 | 10229(5) | 7070(2) | 7545(3) | 60.6(11) |
| C8 | 5788(6) | 7720.8(19) | 8183(3) | 61.7(10) |

Table 2 Fractional Atomic Coordinates (×10⁴) and Equivalent Isotropic Displacement Parameters (Å²×10³) for exp_3182_auto. $U_{eq}$ is defined as 1/3 of the trace of the orthogonalised $U_{IJ}$ tensor.

| Atom | *x* | *y* | *z* | U(eq) |
|---|---|---|---|---|
| C11 | 8835(6) | 7137.2(19) | 8259(3) | 60.3(10) |
| N1 | 7580(4) | 7657.8(11) | 7780(2) | 45.8(6) |
| C1 | 7521(4) | 8891.5(14) | 4802(2) | 43.4(7) |
| C13 | 5352(5) | 5928.6(15) | 5780(3) | 49.7(8) |
| C16 | 7561(6) | 5051.3(16) | 7092(3) | 65.0(10) |
| C14 | 4449(5) | 5422.0(17) | 6139(3) | 58.8(9) |
| C12 | 7396(4) | 5991.8(14) | 6071(2) | 43.4(7) |
| C15 | 5519(6) | 4974.8(16) | 6794(3) | 58.1(9) |
| C17 | 8495(5) | 5544.3(15) | 6726(3) | 56.4(9) |
| C7 | 4609(6) | 8291.7(19) | 7780(3) | 61.9(10) |
| C18 | 4493(7) | 4422.8(18) | 7168(4) | 89.1(14) |

Table 3 Anisotropic Displacement Parameters (Å²×10³) for exp_3182_auto. The Anisotropic displacement factor exponent takes the form: $-2\square^2[h^2a^{*2}U_{11}+2hka^*b^*U_{12}+\ldots]$.

| Atom | $U_{11}$ | $U_{22}$ | $U_{33}$ | $U_{23}$ | $U_{13}$ | $U_{12}$ |
|---|---|---|---|---|---|---|
| Br1 | 84.1(3) | 60.2(3) | 60.2(3) | 17.58(19) | 23.6(2) | -13.72(19) |
| S1 | 55.4(5) | 43.5(5) | 54.7(6) | 12.2(4) | 28.7(4) | 16.6(4) |
| O1 | 49.0(12) | 52.7(14) | 67.5(17) | -0.2(12) | 24.8(12) | 4.2(11) |
| O3 | 70.1(15) | 67.4(17) | 108(2) | 22.0(15) | 58.1(15) | 28.1(13) |
| C9 | 34.9(15) | 32.5(16) | 38.6(19) | 4.2(13) | 7.3(13) | -0.1(13) |
| C4 | 41.9(15) | 35.7(17) | 42.2(19) | -2.2(14) | 10.0(14) | 1.2(14) |
| C3 | 57(2) | 36.6(18) | 52(2) | -5.4(16) | 6.5(17) | 14.7(16) |

Table 3 Anisotropic Displacement Parameters (Å²×10³) for exp_3182_auto. The Anisotropic displacement factor exponent takes the form: -2π²[h²a*²$U_{11}$+2hka*b*$U_{12}$+...].

| Atom | $U_{11}$ | $U_{22}$ | $U_{33}$ | $U_{23}$ | $U_{13}$ | $U_{12}$ |
|---|---|---|---|---|---|---|
| N2 | 34.5(12) | 37.4(14) | 52.2(17) | 11.7(12) | 9.7(12) | 5.2(11) |
| C2 | 74(2) | 30.9(17) | 44(2) | 3.2(15) | 4.3(18) | 9.7(17) |
| C5 | 37.1(14) | 30.3(15) | 34.6(17) | -0.4(13) | 4.2(13) | -0.5(13) |
| O2 | 84.0(15) | 50.4(14) | 41.3(14) | 11.7(11) | 21.1(12) | 18.5(12) |
| C6 | 41.9(16) | 35.8(17) | 40.2(19) | 2.1(14) | 7.1(14) | 0.4(14) |
| C10 | 43.5(19) | 53(2) | 75(3) | 11(2) | -10(2) | 3.1(19) |
| C8 | 86(3) | 56(2) | 53(3) | 7(2) | 36(2) | 1(2) |
| C11 | 73(3) | 58(2) | 43(2) | 7.5(19) | -1(2) | -5(2) |
| N1 | 58.3(15) | 41.1(15) | 36.5(16) | 5.8(12) | 7.7(13) | -0.2(13) |
| C1 | 58.4(18) | 34.9(16) | 37.1(19) | 1.5(14) | 10.9(15) | -6.7(15) |
| C13 | 57.7(19) | 45(2) | 47(2) | -0.8(15) | 12.8(16) | 12.7(16) |
| C16 | 91(3) | 38(2) | 62(3) | 12.0(18) | 10(2) | 9(2) |
| C14 | 61(2) | 52(2) | 67(3) | -9.9(19) | 19.4(19) | -2.8(18) |
| C12 | 54.9(18) | 35.2(16) | 43.0(19) | 0.1(14) | 16.9(15) | 10.4(15) |
| C15 | 90(3) | 38.6(19) | 53(2) | -8.4(17) | 31(2) | -4.9(19) |
| C17 | 62(2) | 41.9(19) | 63(2) | 7.8(18) | 7.9(18) | 9.4(17) |
| C7 | 84(3) | 54(2) | 57(3) | -1.8(19) | 37(2) | 1(2) |
| C18 | 132(4) | 50(2) | 95(4) | -5(2) | 46(3) | -23(3) |

Table 4 Bond Lengths for exp_3182_auto.

| Atom | Atom | Length/Å | Atom | Atom | Length/Å |
|---|---|---|---|---|---|
| Br1 | C1 | 1.900(3) | C2 | C1 | 1.381(4) |

Table 4 Bond Lengths for exp_3182_auto.

| Atom | Atom | Length/Å | Atom | Atom | Length/Å |
|---|---|---|---|---|---|
| S1 | O3 | 1.432(2) | C5 | C6 | 1.383(4) |
| S1 | N2 | 1.633(3) | C6 | C1 | 1.383(4) |
| S1 | O2 | 1.431(2) | C10 | C11 | 1.489(5) |
| S1 | C12 | 1.764(3) | C8 | N1 | 1.455(4) |
| O1 | C4 | 1.374(3) | C8 | C7 | 1.492(5) |
| O1 | C7 | 1.451(4) | C11 | N1 | 1.459(4) |
| C9 | N2 | 1.474(3) | C13 | C14 | 1.379(4) |
| C9 | C5 | 1.513(4) | C13 | C12 | 1.385(4) |
| C9 | N1 | 1.468(4) | C16 | C15 | 1.387(5) |
| C4 | C3 | 1.384(4) | C16 | C17 | 1.372(5) |
| C4 | C5 | 1.402(4) | C14 | C15 | 1.380(5) |
| C3 | C2 | 1.376(5) | C12 | C17 | 1.388(4) |
| N2 | C10 | 1.490(4) | C15 | C18 | 1.509(5) |

Table 5 Bond Angles for exp_3182_auto.

| Atom | Atom | Atom | Angle/° | Atom | Atom | Atom | Angle/° |
|---|---|---|---|---|---|---|---|
| O3 | S1 | N2 | 106.41(15) | C5 | C6 | C1 | 120.0(3) |
| O3 | S1 | C12 | 107.87(14) | C11 | C10 | N2 | 102.7(3) |
| N2 | S1 | C12 | 108.89(14) | N1 | C8 | C7 | 112.9(3) |
| O2 | S1 | O3 | 120.30(15) | N1 | C11 | C10 | 102.1(3) |
| O2 | S1 | N2 | 105.39(13) | C8 | N1 | C9 | 112.8(3) |
| O2 | S1 | C12 | 107.58(15) | C8 | N1 | C11 | 112.8(3) |
| C4 | O1 | C7 | 114.7(3) | C11 | N1 | C9 | 105.1(3) |

Table 5 Bond Angles for exp_3182_auto.

| Atom | Atom | Atom | Angle/° | Atom | Atom | Atom | Angle/° |
|---|---|---|---|---|---|---|---|
| N2 | C9 | C5 | 113.1(2) | C2 | C1 | Br1 | 118.7(2) |
| N1 | C9 | N2 | 104.2(2) | C2 | C1 | C6 | 121.3(3) |
| N1 | C9 | C5 | 111.1(2) | C6 | C1 | Br1 | 120.0(2) |
| O1 | C4 | C3 | 118.2(3) | C14 | C13 | C12 | 119.5(3) |
| O1 | C4 | C5 | 121.0(3) | C17 | C16 | C15 | 121.3(3) |
| C3 | C4 | C5 | 120.7(3) | C13 | C14 | C15 | 122.0(3) |
| C2 | C3 | C4 | 120.2(3) | C13 | C12 | S1 | 120.6(2) |
| C9 | N2 | S1 | 117.99(18) | C13 | C12 | C17 | 119.2(3) |
| C9 | N2 | C10 | 107.7(3) | C17 | C12 | S1 | 120.3(3) |
| C10 | N2 | S1 | 119.3(2) | C16 | C15 | C18 | 121.5(4) |
| C3 | C2 | C1 | 119.2(3) | C14 | C15 | C16 | 117.7(3) |
| C4 | C5 | C9 | 117.8(3) | C14 | C15 | C18 | 120.8(4) |
| C6 | C5 | C9 | 123.6(2) | C16 | C17 | C12 | 120.3(3) |
| C6 | C5 | C4 | 118.6(3) | O1 | C7 | C8 | 113.7(3) |

Table 6 Torsion Angles for exp_3182_auto.

| A | B | C | D | Angle/° | A | B | C | D | Angle/° |
|---|---|---|---|---|---|---|---|---|---|
| S1 | N2 | C10 | C11 | -117.6(3) | C5 | C4 | C3 | C2 | -0.9(5) |
| S1 | C12 | C17 | C16 | 179.3(3) | C5 | C6 | C1 | Br1 | -178.0(2) |
| O1 | C4 | C3 | C2 | -178.1(3) | C5 | C6 | C1 | C2 | -0.2(5) |
| O1 | C4 | C5 | C9 | 1.8(4) | O2 | S1 | N2 | C9 | 44.8(2) |
| O1 | C4 | C5 | C6 | 179.5(3) | O2 | S1 | N2 | C10 | 178.7(2) |
| O3 | S1 | N2 | C9 | 173.6(2) | O2 | S1 | C12 | C13 | -18.6(3) |

Table 6 Torsion Angles for exp_3182_auto.

| A | B | C | D | Angle/˚ | A | B | C | D | Angle/˚ |
|---|---|---|---|---|---|---|---|---|---|
| O3 | S1 | N2 | C10 | -52.5(3) | O2 | S1 | C12 | C17 | 161.2(3) |
| O3 | S1 | C12 | C13 | -149.8(3) | C10 | C11 | N1 | C9 | 42.8(3) |
| O3 | S1 | C12 | C17 | 30.0(3) | C10 | C11 | N1 | C8 | 166.1(3) |
| C9 | N2 | C10 | C11 | 20.5(4) | N1 | C9 | N2 | S1 | 143.86(19) |
| C9 | C5 | C6 | C1 | 175.7(3) | N1 | C9 | N2 | C10 | 5.2(3) |
| C4 | O1 | C7 | C8 | -84.1(4) | N1 | C9 | C5 | C4 | -67.7(3) |
| C4 | C3 | C2 | C1 | -1.2(5) | N1 | C9 | C5 | C6 | 114.7(3) |
| C4 | C5 | C6 | C1 | -1.8(4) | N1 | C8 | C7 | O1 | 65.2(5) |
| C3 | C4 | C5 | C9 | -175.3(3) | C13 | C14 | C15 | C16 | 0.4(5) |
| C3 | C4 | C5 | C6 | 2.4(4) | C13 | C14 | C15 | C18 | -179.5(3) |
| C3 | C2 | C1 | Br1 | 179.6(2) | C13 | C12 | C17 | C16 | -0.9(5) |
| C3 | C2 | C1 | C6 | 1.8(5) | C14 | C13 | C12 | S1 | 179.3(3) |
| N2 | S1 | C12 | C13 | 95.1(3) | C14 | C13 | C12 | C17 | -0.4(5) |
| N2 | S1 | C12 | C17 | -85.1(3) | C12 | S1 | N2 | C9 | -70.4(2) |
| N2 | C9 | C5 | C4 | 175.4(2) | C12 | S1 | N2 | C10 | 63.5(3) |
| N2 | C9 | C5 | C6 | -2.1(4) | C12 | C13 | C14 | C15 | 0.7(5) |
| N2 | C9 | N1 | C8 | -153.0(3) | C15 | C16 | C17 | C12 | 2.1(6) |
| N2 | C9 | N1 | C11 | -29.8(3) | C17 | C16 | C15 | C14 | -1.8(5) |
| N2 | C10 | C11 | N1 | -38.2(4) | C17 | C16 | C15 | C18 | 178.1(4) |
| C5 | C9 | N2 | S1 | -95.3(3) | C7 | O1 | C4 | C3 | -116.9(3) |
| C5 | C9 | N2 | C10 | 126.0(3) | C7 | O1 | C4 | C5 | 65.9(4) |
| C5 | C9 | N1 | C8 | 84.9(3) | C7 | C8 | N1 | C9 | -67.2(4) |
| C5 | C9 | N1 | C11 | -151.9(3) | C7 | C8 | N1 | C11 | 174.0(3) |

Table 7 Hydrogen Atom Coordinates (Å×10⁴) and Isotropic Displacement Parameters (Å²×10³) for exp_3182_auto.

| Atom | *x* | *y* | *z* | U(eq) |
|---|---|---|---|---|
| H13 | 4593.72 | 6225.74 | 5345.82 | 60 |
| H16 | 8311.11 | 4762.76 | 7548.67 | 78 |
| H14 | 3076.64 | 5380.65 | 5934.42 | 71 |
| H17 | 9869.87 | 5579 | 6918.29 | 68 |
| H18A | 4935.29 | 4040.57 | 6903.37 | 134 |
| H18B | 3083.32 | 4463.52 | 6917.35 | 134 |
| H18C | 4807.72 | 4413.2 | 7920.74 | 134 |
| H6 | 9190(40) | 8151(13) | 5380(20) | 43(8) |
| H3 | 3390(40) | 9244(14) | 5470(20) | 52(9) |
| H2 | 5410(40) | 9555(16) | 4390(20) | 57(10) |
| H7A | 5330(40) | 8685(17) | 7970(30) | 66(11) |
| H11A | 8000(40) | 6733(16) | 8300(20) | 58(9) |
| H11B | 9430(40) | 7255(16) | 8920(30) | 60(11) |
| H10A | 10880(40) | 6668(17) | 7560(30) | 64(10) |
| H10B | 11230(40) | 7362(15) | 7690(20) | 52(10) |
| H7B | 3390(50) | 8323(18) | 8130(30) | 92(13) |
| H8A | 6030(50) | 7707(19) | 8980(30) | 99(14) |
| H8B | 4920(50) | 7361(17) | 8020(30) | 69(11) |
| H9 | 5960(30) | 7273(12) | 6434(19) | 27(7) |
